# A Comprehensive Review on Filled Carbon Nanotubes: Synthesis, Properties and Applications

**DOI:** 10.1021/acs.chemrev.5c00219

**Published:** 2026-02-04

**Authors:** Stefania Sandoval, Gil Gonçalves, Jorge Pérez Barrio, Marianna V. Kharlamova, Gerard Tobías-Rossell

**Affiliations:** † Institut de Ciència de Materials de Barcelona (ICMAB-CSIC), Campus UAB, 08193 Bellaterra, Barcelona, Spain; ‡ TEMA − Centre for Mechanical Technology and Automation, Department of Mechanical Engineering, University of Aveiro, 3810-193 Aveiro, Portugal; § LASI − Intelligent Systems Associate Laboratory, 4800-058 Guimarães, Portugal; ∥ Faculty of Physics, University of Vienna, Strudlhofgasse 4, 1090 Vienna, Austria

## Abstract

Carbon nanotubes (CNTs) have emerged as one of the most exciting families of carbon nanomaterials. Their hollow tubular architecture, with a nanometric inner cavity, not only defines their distinct physical and chemical behavior but also enables the encapsulation of a wide range of materials, including inorganic and organic compounds. This encapsulation capability allows CNTs to function as nanocontainers, protective hosts, and confined reaction vessels, leading to novel hybrid materials with tailored optical, electronic, catalytic, and mechanical properties. In this review, we provide a comprehensive overview of the methodologies employed for filling CNTs, including *in situ* and *ex situ* approaches. We critically examined the diverse range of materials encapsulated within CNTs, highlighting how confinement at the nanoscale influences their chemical reactivity, phase stability, and emergent quantum phenomena. Special attention is given to the wide range of applications of filled CNTs in addressing societal challenges. These include biomedicine, catalysis, energy storage, gas separation, filtration membranes, sensing technologies, and nanoelectronics. Beyond revisiting the current state-of-the-art, this review offers a critical discussion of future directions and challenges in this field.

## Graphical abstract

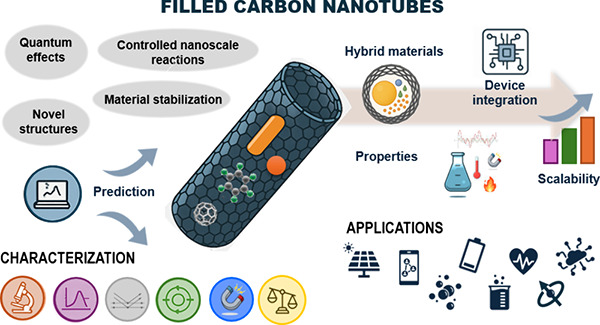

## 1 Introduction

Carbon nanotubes (CNTs) are an allotropic form of nanocarbon that has gained attention from the scientific community. They are cylinders with one (single-walled carbon nanotubes, SWCNTs), two (double-walled carbon nanotubes, DWCNTs), or more (multiwalled carbon nanotubes, MWCNTs) concentric layers of rolled carbon atomic sheets. If no defects are present within the layers, a perfectly seamless material composed of a hexagonal lattice is obtained. Although early observations of tubular carbon nanostructures were made several decades earlier,^1^ a pioneering report by Sumio Iijima in 1991 ^2^ drew the attention of the scientific community to this new class of synthetic carbon nanomaterials, which presented extraordinary properties unlike any other carbon material reported before. More than 30 years later, CNTs are produced worldwide in kiloton quantities and can be found in several commercialized materials (turbines, vessels, sporting goods, etc.).^3,4^

Several synthetic routes have been established for the preparation of CNTs,^5–7^ with chemical vapor deposition (CVD) being the preferred method for large-scale synthesis of CNTs. Other available methods for CNTs’ production include laser ablation (LA) and electric arc discharge (AD). Regardless of the method used for their synthesis, as-made CNT samples can contain several impurities, such as catalyst particles, amorphous material, or graphitic particles. Attempts to remove these impurities have been made using different methods, such as oxidation by air,^8^ steam,^9^ thermal treatment,^10^ sonication, chemical acid treatments,^11^ microfiltration, and centrifugation.^12^ However, none of these methods are universal, as each presents its own intrinsic advantages and disadvantages. The most convenient purification strategy depends on the type of impurities present in the sample and the desired structural characteristics of the final material. This is because the purification treatment might also lead to the introduction of functional groups on the external walls, shorten the length of the CNTs, and/or open their ends.

CNTs not only act as directing agents for synthesis and protection against external damage, but the resulting hybrids also usually exhibit enhanced optical and electronic properties.^13^ The preparation of nanowires (NWs),^14^ nanorings,^15^ nanoribbons,^16–18^ nanotubes (NTs),^19^ or one-dimensional (1D) atomic chains,^20–22^ among many other encapsulated nanostructures, is being intensely studied; the final structure of the guest is determined not only by the chemical nature of the bulk material but also by the characteristics of the CNTs^20^ and the reaction conditions.^23^ Several reviews have been published in the literature that focus on the filling of CNTs.^24–32^ Initial reports emphasized the encapsulation of inorganic compounds, most likely because of the technical difficulties in characterizing organic structures once confined within CNTs. Recently, Kharlamova et al. reviewed the effect of the encapsulation of materials on the electronic properties of the resulting hybrid,^33^ and Teng et al. presented an analysis that provides an overview of filled CNTs with special emphasis on selected applications, namely, energy storage, electronics, sensors, and catalysis.^6^ Filled CNTs have also been discussed in reviews focused on inorganic NTs^34,35^ and within the broader family of 1D van der Waals (vdW) heterostructures.^36–38^ 1D vdW heterostructures is a more general term that includes both endohedral and external coaxial coating of CNTs and other tubular structures.^39^ The aim of this review is to provide a comprehensive and up-to-date state-of-the-art in this rapidly evolving research field. We discuss different filling strategies, review a wide range of guest materials, analyze how encapsulation leads to novel structures and properties, and provide examples of the broad spectrum of applications of the resulting hybrids.

## 2 Mechanisms of Encapsulation and Confinement Effects

The encapsulation of foreign species within CNTs cavities occurs through different mechanisms that rely on aspects such as capillarity, vapor pressure, and interfacial interactions. Both the physical and chemical properties of the filler are crucial aspects to consider. The state of the guest species during encapsulation is determined by the methodology employed during the synthesis. The forces driving the migration of the material, the morphology of the host, and the stability of the intermediates and newly formed hybrids also play major roles. Additional parameters, such as viscosity and crystal-NT interaction energies, should be considered when selecting the material to be encapsulated (Table 1).

**Table 1:** Mechanisms and Main Aspects Driving the Confinement of Foreign Materials within CNTs

Mechanism	Driving force	Driven/deposited specie	Main considerations
***In situ*** **encapsulation**	Catalyst. Energy promotes the growth and interaction between the reactants.	Molecule/crystalline structure	• It requires harsh reaction conditions.

**Capillary action**	Minimization of surface energy. Capillary forces (Young–Laplace equation).	Molecule	• This depends on the wettability of the CNT inner walls and the surface tension.
			• Open ended hosts are required.

**Vapor diffusion**	Diffusion (concentration gradients and pressure).	Ion/molecule	• This requires precise control of T and vapor pressure.
	Adsorption (vdW interactions).		• Open ended hosts are required.

**Liquid-phase diffusion**	Intermolecular interactions between the host, guest, and vehicle (solvent).	Ion/molecule	• This depends on the affinity of the solvent for the host and filling agent. Open ended hosts are required.
	Chemical processes (e.g., precipitation and reduction) occur within the CNT’s cavity.		• Size matching between the guest and the inner cavities of CNTs is required.

**EC deposition**	Applied electrical potential.	Ion/molecule	• The host CNTs must be conductive.

*In situ* encapsulation involves filling during the synthesis of CNTs. This process requires the presence of a carbon source and specific precursors that are treated under conditions that promote the simultaneous growth of tubular nanostructures and guest materials. A driving force able to induce the transformation is required, namely, an energy source (such as temperature (*T*) or an electric discharge) or an agent that lowers the activation energy of the reaction to increase the growth/filling rate (catalyst). In most cases, the process proceeds via the encapsulation of the catalyst itself, which therefore acts as both the driving force and guest material.

Capillary action is one of the most commonly used mechanisms for filling CNTs. According to the Young–Laplace equation, any liquid with a surface tension below 200 mN m^–1^ should spontaneously diffuse into the CNT’s cavity, whereas for higher surface tension values, filling would only be possible if a suitable pressure difference at the interface is present.^40^ Capillary action involves the exposure of open ended CNTs to materials in its liquid phase. Therefore, materials that are liquid under normal conditions (including solutions and suspensions) or those molten by an energetic driving force (such as *T*) can spontaneously fill CNTs by capillary forces. When the material to be encapsulated within the host CNTs is dissolved in a solvent, a liquid-phase diffusion mechanism primarily governs the filling process. Because the intermolecular interactions between the host, guest, and solvent significantly affect the mechanism of incorporation, the latter can also play a driving role, either favoring or slowing down the encapsulation.^41^ Yudasaka et al. proposed two different mechanisms for the incorporation of buckminsterfullerenes (C_60_) within SWCNTs in liquid phases.^42^ The authors established that C_60_ enters the hosts via direct deposition within their cavities when ethanol was used as solvent (nanoextraction), while a thin layer of C_60_ molecules was formed on the external surface of the CNTs, prior to confinement, when a C_60_-toluene saturated solution was used during the process (nanocondensation) (Figure 1). One may take advantage of the affinity of certain species for both the host and solvent to promote the release of other systems within the CNTs. Thus, discrete molecules such as C_60_ have been employed to drive the displacement of low-affinity species from the inner surface of CNTs,^43^ which confirms the important role of interactions, such as vdW forces in the encapsulation process.^44^

**Figure 1:**
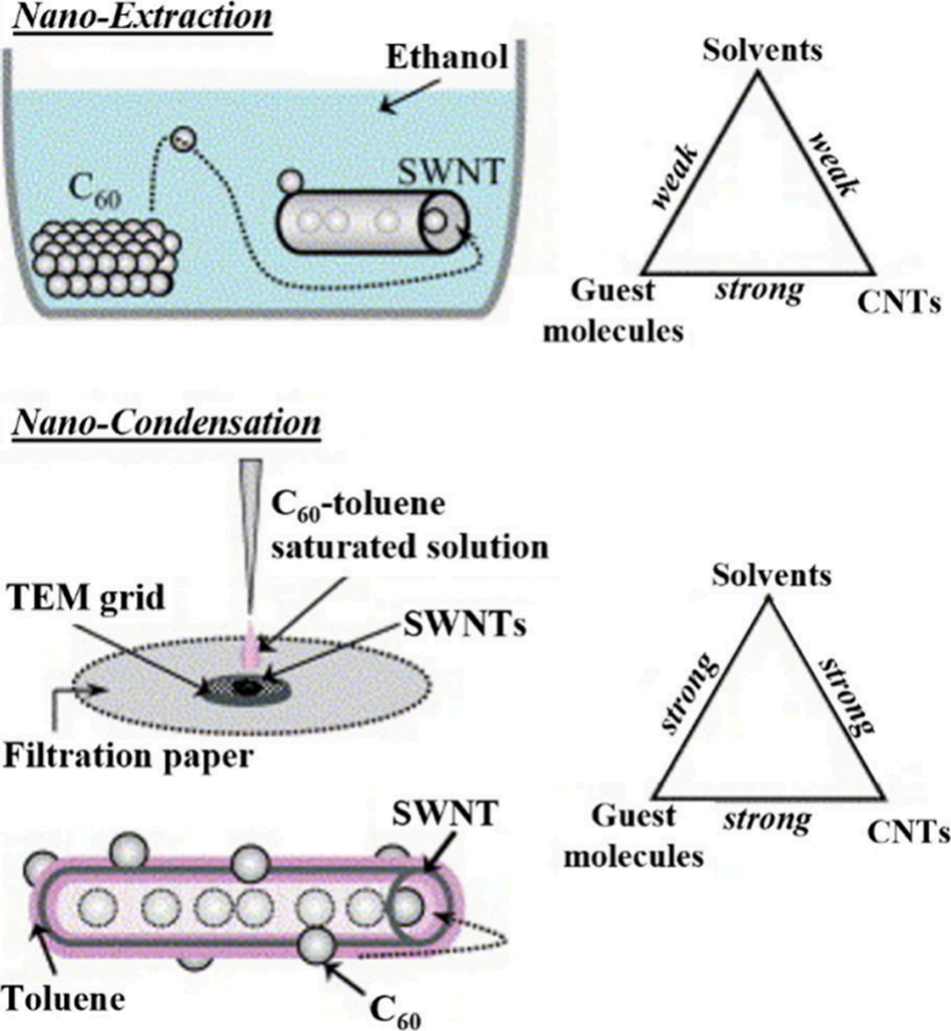
Schematic representation of nanoextraction and nanocondensation approaches. The mechanism of C_60_ approaching and encapsulation within SWCNTs depends closely on the solvent employed as the vehicle. Reproduced with permission from ref 42. Copyright 2003, Elsevier.

Materials that are easily vaporized, have a high surface tension, or are gases under normal conditions can be encapsulated by exploiting vapor diffusion. Concentration gradients drive the mechanism, in which once the species enter the CNT’s cavities, adsorption and close interactions between the gas-phase molecules and the host occur. *T* gradients can favor the encapsulation of sublimated materials, which tend to recrystallize when they diffuse into cooler regions. Because the equilibrium between the matter states is closely related to the pressure and *T* of the system, these parameters can be controlled carefully. In some cases, other substances that promote the vaporization and diffusion of the material into the CNT’s cavities can be used to fill them.

Finally, electrochemical (EC) deposition occurs via the application of an electrical potential, which drives the encapsulation of species dissolved in an electrolyte inside CNTs (which act as the electrode material). Nie et al. studied the role of redox potential on the final filling yield of different metal iodides.^45^ They reported the influence of the physical properties of inorganic guests on the filling rate of CNTs. Moreover, the chemical reactivity of the potential guests plays a major role in endohedral functionalization.

Energetic factors may also explain some counterintuitive results during the filling process.^46^ The spontaneity of the encapsulation process depends on the stability of the former precursors, host, potential guests, and the formed hybrid. Aspects such as the energy necessary to promote melting or vaporization, breaking intermolecular interactions between the solvent and filler, and the establishment of new connections, such as vdW forces with the CNTs surface, may be considered to propose a coherent filling mechanism. One of the most representative examples is the encapsulation of water in hydrophobic CNTs. It is expected that a great energy consumption will break the intermolecular water–water H-bonds, thus decreasing both the entropy and enthalpy of the process. Despite the energy requirement being partially compensated by the stabilization induced by the formation of vdW interactions between water and the host, one might expect that preparing H_2_O@CNTs is not straightforward. In contrast, the process is spontaneous, as demonstrated by both experimental reports and theoretical models,^47^ which, among other aspects, consider translational and rotational entropy, affected by, for instance, the CNTs dimensions or the viscosity of the confined liquid.

## 3 Quantum Confinement (QC) in Filled CNTs

It has already been demonstrated that the confinement of compounds within the inner cavity of CNTs can alter not only the structure^48^ and hence the properties of the filling material, but also the properties of the hosting CNT (for example, confinement effects or mechanical behaviors).^17,49–51^ Atomic interactions between the atoms/molecules of the guest material can also be altered by encapsulation, resulting in significant variations in the properties of the confined and unconfined (bulk or solution) states.^33,52^

Two initial aspects must be considered to evaluate the effects of QC when analyzing a system formed by foreign species located inside carbon-based tubular nanostructures. The first includes intrinsic confinement that occurs within the structure of the host. CNTs are 1D systems, ideally formed by concentrically rolling up one or more graphene layers. Aspects such as the electronic properties and thermal conductivity are ruled by the diameter, number of walls, chirality of the system, curvature, or the presence of topological defects, such as the introduction of pentagons or heteroatoms within the *sp* ^*2*^ honeycomb lattice,^53^ which significantly affect the mobility of the electrons (e^–^) within the NT walls. In CNTs, the delocalized electrons can be confined either around the circumference that forms the cylinder-like structure or along the axial direction, which is limited by the CNTs length.^54^ Therefore, modification of CNTs dimensions enables tuning of their electronic behavior. Fluorescent hosts, for instance, can be prepared by shortening the SWCNTs with *sp* ^3^ defects. A blueshift (to the lowest exciton energy) has been predicted for ultrashort CNTs, whose length approaches the exciton Bohr radius. The energy scales with length as Δ*E*_11_ ∼ *L* ^–1/2^ for both semiconducting and metallic SWCNTs.^55^

Understanding the intrinsic confinement of CNTs is crucial for explaining the mechanisms of interaction and encapsulation of foreign species within the CNTs’ cavity. Topological defects can induce changes in the reactivity of CNTs walls. By electronic or substitutional doping, high reactivity and high surface area adsorption sites can be formed, thus creating suitable environments to establish intermolecular interactions with the approaching molecules as a preliminary step to the encapsulation. The intermolecular distances between the adsorber and adsorbate can also be enhanced by controlling the environmental conditions. Pressure-induced confinement within SWCNTs can contribute to reducing intermolecular distances and enhancing resonance effects, thereby promoting adsorption. Once encapsulated, high pressure can induce sintering of the encapsulated system as well.^56^

The second aspect involves the confinement of foreign materials within a narrow cavity. The confinement process depends on the interactions established between the approaching species and the CNTs surface (both external and internal) and on the chemical and physical properties and how the space restriction modifies these properties. There are multiple reports on how confinement can induce structural modifications in both organic and inorganic materials.^48^ Examples include the formation of new systems, otherwise unstable or unreported phases in bulk.^57–59^

Confinement alters the structure, binding, vibrational modes, and electronic configuration of the encapsulated system and the way in which both guest and host interact. QC can be used to tune the characteristics of the filled system, thereby improving their properties and favoring their applications in different fields. While the diameter of CNTs can be tuned to create selective functional sieves, specific gas molecules can be adsorbed and separated from gas mixtures by using SWCNTs with specific chiralities.^60^ Among other applications, CNTs improve the catalytic behavior of confined systems due to narrowing of the interactions of the reactants and charge transfer between the host and the catalyst that stabilizes the system, favors the formation of intermediates or ensure the reusability of the hybrid.^61^

### 3.1 Effects of the Nanoconfinement in Both Structure and Properties of the Filler

There are four main aspects that may be considered to evaluate how the encapsulation within the cavity of a CNT can alter a guest entity, namely, variations in the structure and morphology, physical and chemical properties, chemical reactivity, and transport properties.^62^ As mentioned above, the encapsulation of foreign molecules within CNTs is limited by their tight 1D inner surface. Therefore, CNTs can accommodate discrete molecules or more complex crystalline structures with dimensions smaller than their diameter. Once encapsulated, both the shape of the host and the interaction with its surface play major roles in the configuration adopted by the guest entities. Numerous reports have described unique and unprecedented crystalline structures (polymorphs) grown within CNTs.^63^ When the dimensions of the guest materials surpass the diameter of the CNTs internal cavities, they can undergo structural compression and deformation, which may lead to the creation of new nanosystems that are frequently unstable in their bulk form.^52,64–66^ The strain induced by the curvature of the CNTs walls that closely interact with the confined system, either by the formation of covalent bonds or by intermolecular forces, along with the deformation caused by the limited available space are responsible for substantial modifications of the electronic and optical properties of the encapsulated material.

One of the most widely studied effects of nanoconfinement is the variation in the band structure of semiconducting and insulating systems, which depends on parameters such as lattice structure, symmetry, and binding characteristics. As discussed, encapsulation significantly affects these parameters. Furthermore, once the material is grown within CNTs, the electrons are confined in directions perpendicular to the long axis, which means that the diameter of the host is crucial for describing its electronic properties. Confinement induces quantization of the energy levels, which, in the case of CNTs, is inversely proportional to the square of their diameter. Consequently, the electronic properties of the system can be precisely tuned using CNTs with disparate dimensions. Filling narrow CNTs with transition metal chalcogenides (TMCs)^67–69^ or carbon allotropes has led to the formation of 1D or quasi-1D structures with band structures that significantly differ from their bulk 2D and 3D counterparts.^70^ Milligan et al. described the encapsulation of single quasi-1D vdW chains of Sb_2_Se_3_ within SWCNTs. Theoretical approaches indicated electron transfer from the tubular host to the inorganic guest, and a blue-shift of the near-infrared (NIR) bandgap of Sb_2_Se_3_, which appeared around 600 nm, was observed in the Raman spectrum.^71^ GeX_2_ (X = S, Se) crystals have also been confined within narrow carbon-based tubular nanostructures. After encapsulation, GeX_2_ adopted 1D configurations with tetrahedral connectivity, which is CNTs size dependent and that revealed tunable semiconducting properties through composition and dimension engineering.^68^

The spin-polarized states of the materials also undergo significant modifications after encapsulation within CNTs. The spin polarization of confined ferromagnetic materials, for instance, is strongly affected by the shape adopted by the crystals (shape anisotropy), which are frequently solid and continuous NWs in which the magnetic moments align along the axis parallel to the CNT, minimizing the magnetostatic energy. Shape anisotropy is size dependent and is favored by large length-to-diameter ratios of the crystals, which also enhances the spin polarization along the CNTs axis.

Confinement also induces novel spin ordering, which provokes magnetic behavior in samples that are non-magnetic in bulk. This phenomenon can be explained in terms of the restricted interaction of the magnetic moments of the atoms with their two nearest neighbors in a 1D system (Ising Model Analogy), which induces a long-range magnetic order that depends on the *T*, crystalline structure of the filler, and diameter of the CNTs. Spin polarization is also induced by the interaction between confined crystals and the host. The magnitude of this interaction and its effect on the magnetic behavior of the material depend on aspects such as the curvature of the CNTs, which enhances the spin–orbit coupling in carbon atoms, the chirality of the CNTs, and the proximity of the encapsulated atoms to the CNTs surface. Thus, CNTs have been widely used to modify or induce the magnetic properties of various systems, owing to the possibility of obtaining tunable materials with potential applications in fields such as spintronics and biomedicine. Jo et al. used spin polarized ab initio calculations to evaluate the effect of encapsulation on the magnetic properties of Fe, Co, and Ni NWs.^72^ Although the transition metals confined inside (5, 5) SWCNTs did not undergo variations in the M-M interactions compared with free-standing NWs, significant charge transfer and, therefore, binding energies, were calculated for the M–C bond. ferromagnetic atoms to the C shell.^72^ In agreement with other reports, encapsulation induced a reduction in the magnetic moment of the NWs, which was more pronounced in the case of Fe. More recently, the growth of 1D magnetic MX_3_ single chains (M = Cr, V; X = Cl, Br and I) was reported.^73^ In the case of confined metal halides (MXs), the stabilization of the system and magnetic phase transitions are driven by charge transfer from the carbonaceous host to the MX chains.

## 4 Theory and Simulation Tools for Filled CNTs

Computational methods have provided useful insights into the encapsulation of various species within the cavities of CNTs. Simulation tools are often employed to decipher the confinement mechanisms of foreign species inside CNTs, considering different aspects, such as the affinity of both host and guest entities, their interactions, and the stability of the resulting hybrid. Theoretical calculations also enable the exploration of the properties of emerging nanostructures.

Simulations can be used to determine the energy variation that occurs when guest molecules approach and are confined to the hosting CNTs. For this purpose, different aspects, such as the environment, physical state, and chemical and physical affinities between the reactants, should be considered. Once the foreign species are encapsulated, the energy of interaction and the structural and morphological aspects of the emerging hybrid enable the determination of the stability of the system and its physicochemical properties, namely, electronic structure, mechanical properties, and thermal conductivity, among others.

Computational methods include *ab initio* calculations (density functional theory (DFT) being the most widely used), Quantum Monte Carlo (QMC), Molecular Dynamics (MD) simulations, and Finite Element Method (FEM). Owing to the discrepancy in the theoretical base used to predict the characteristics of the hybrids formed during the filling process in every methodology, the information provided by each approach is different and can be complementary (multiscale modeling). Moreover, theoretical approaches are frequently used to confirm the structural features and properties when simulations are compared with the performed characterization, namely, spectroscopic and microscopic techniques, X-ray diffraction (XRD), magnetic measurements, or mechanical properties, to name a few.

Table 2 summarizes the aspects analyzed and the information provided by the main theoretical approaches applied in the research on filling CNTs. The advantages, disadvantages, and applications of the methodologies are described and some relevant references are included.

**Table 2:** Main Theoretical Approaches Employed for the Research on Filling CNTs

Theoretical approach	Provided insights	Advantages	Disadvantages	Application	References
**DFT and other ab initio methods**	• Adsorption energies.	• High accuracy at the atomic/molecular levels.	• Computationally intensive, limiting the size and time scale of the systems that can be studied.	• Structural characterization, energetics and electronic profiles.	^74–86^
	• Electron densities.	• Provides a high level of accuracy for chemical bonding and electronic properties.		• Energy generation, power conversion, solar cells, batteries.	^87–89^
	• Binding energies.			• Sensors.	^90^
	• Electronic properties.			• Electronic devices: Spintronics, nanoelectronics.	^91–94^
	• Stability of the nanostructure.			• Catalysis.	^76^
	• Bond lengths.			• Gas storage and separation: Energy storage.	^95–97^
	• Lattice parameters.			• Pollutant removal.	^98^
	• Charge transfer.			• Biomedicine: Drug delivery and sensors.	^99^
**QMC**	• Adsorption isotherms.	• Enables efficient calculation of equilibrium properties.	• Does not provide information about the dynamic or time-dependent processes.	• Adsorption of liquids.	^100^
	• Uptake of adsorbed molecules.	• Does not require knowing forces and trajectories.		• Gas storage and separation: Nuclear waste disposal, hydrogen storage.	^101–107^
		• Well-suited for GC ensembles.		• Magnetic storage.	^108^
**MD simulation**	• Filling mechanism.	• Useful for analyzing time-dependent dynamic processes.	• Not as accurate as DFT owing to the empirical force fields-based method employed for calculations.	• Nanofluidics: Study of the encapsulation of liquids.	^46^,^109^,^110^
	• Thermodynamic properties (ΔG, enthalpy, entropy).		• Does not describe electronic properties.	• Study of the encapsulation of crystals and single entities.	^49^,^111–115^
	• New crystalline structures and configurations.				
	• Transport properties.			• Composite synthesis.	^116^
	• Dynamics of crystal growth:			• Gas storage and separation: Energy storage, pollutant removal, nuclear waste disposal.	^103^,^106^,^117–121^
	• Buckling behavior.			• Electronic devices.	^122^
				• Biomedicine: Drug transport, drug delivery, protein adsorption.	^44^,^114^,^123–125^
				• Nanoreactors.	^111^
**FEM**	• Mechanical properties of CNTs.	• Enables analyzing complex geometries and boundary conditions.	• Not an atomistic or dynamic method, so it is not typically used to simulate the physical process of filling.	• Synthesis of reinforced nanocomposites.	^126^,^127^
		• Application in large-scale systems.	• Requires linking to MM to define material properties at the nanoscale.		
**ML**	• Accelerating MD predictions.	• Reduces costs.	• The accuracy depends on the quality and quantity of the existing data.	• Electronic devices.	^128^,^129^
	• Electronic, and mechanical properties of filled CNTs.	• Can handle large data sets and find complex, non-linear relationships.		• Thermal transport.	
	• Enables finding optimal candidates for filling.				

### 4.1 Density Functional Theory (DFT) and Other *Ab Initio* Methods

DFT is a quantum mechanical approach (*ab initio* quantum chemistry method) that enables the calculation of the electronic structure and energetics of multiple-electron systems at the atomic level. Owing to the first-principles nature of the approach, it is based on physical constants to solve the Schrödinger equation without considering empirical parameters or experimental approaches. DFT methods allow the accurate determination of chemical properties; however, they are time-consuming and require complex computational systems. Consequently, their use is limited to non-complex systems, such as discrete molecules or nanostructures with limited numbers of atoms and short-lapse-time dynamic processes. Since the first reports describing the band structure of CNTs,^130^ *ab initio* approaches have been employed to predict the properties of these graphitic-based systems. In the field of filling CNTs, they are usually employed to describe the interactions of the material to be encapsulated within the CNTs, providing information on the adsorption energy, emerging bonds, and charge transfer between the guest and host.^97^ Based on the newly formed nanostructures, DFT modeling predicts the acquired electronic properties and potential applications. Variations in the morphology, crystalline structure, and deformation of the host CNTs can also be predicted using DFT.

DFT calculations have been used to assess the crystal structure of inorganic halides^131^ and TMCs^14,18,132,133^ encapsulated within CNTs, the stability of monoelemental nanostructures,^15,20,74^ and other inorganic systems within CNTs, and very often, are parallelly performed along with experimental research to confirm the characteristics of the grown inorganic nanostructure, as determined, for instance by means of electron microscopy or spectroscopic assessment.^15,18^ *Ab initio* methods are useful tools to understand the adsorption process of pure molecules^60,134–136^ and mixtures of gases, which is particularly useful to develop functional materials for pollutant removal,^98^ sensors,^90^ and energy storage.^95–97^

Owing to the limitations of DFT in processing complex hybrid systems, other *ab initio* methods have been used to simulate the interactions between foreign species and CNTs. These include a series of approaches known as “post-Hartree-Fock (HF)” methods that improve the solution of the wave equation by including other aspects that affect the electron movement (besides the field induced by adjacent electrons), such as electron repulsions. Post-HF methods include the Møller–Plesset Perturbation Theory (MPn), Configuration Interaction (CI) methods or the Coupled Cluster (CC) Theory.^137^

In dynamic systems, where strong and fast transformations occur, such as bond formation and breaking, *ab initio* methods using an approximation different from HF are particularly useful. These approaches are known as Multireference Methods,^138^ and have recently been used to analyze complex electronic structures and reactions, for instance, during catalytic processes^139^ or when CNTs are used as nanoreactors.

### 4.2 Quantum Monte Carlo (QMC) Method

As in the case of DFT and other calculation approaches described above, the QMC method is an *ab initio* approach that allows the prediction of the behavior and properties of physical systems. Unlike other methods that solve the Schrödinger equation based on HF theory, QMC consists of a statistics-based approximation that uses random sampling techniques to evaluate integrals. QMC calculations enable accurately solving more complex systems compared with other first-principle approaches, being suitable for the analysis at the nanoscale.^140^

Grand Canonical Monte Carlo (GCMC) simulations are typically employed to model the adsorption processes and the interactions between the adsorbates and host CNTs during encapsulation. Several reports describing the confinement of gas^101–107^ and liquid^100^ phase species take advantage of this approach to calculate the adsorption isotherms and understand the adsorption–desorption equilibrium of the system under different pressure and *T*.

### 4.3 Molecular Dynamics (MD) Simulations

MD simulations use classical physics to model the behavior of single entities, such as atoms or molecules. By solving the equations of motion of Newton, the MD approach simulates the dynamics of the movement and interactions of the species over time. Empirical potential energy functions were employed to determine the forces of these interactions. In non-static systems or processes, such as the encapsulation of molecules within CNTs, MD enables the modeling of how foreign species approach and enter the CNT and the energy required during encapsulation. The *T* of the system, concentration of the reactants, and applied pressure can be considered to determine the thermodynamic aspects, namely, Gibbs free energy (Δ*G*), entropy, and enthalpy, thus providing information on the energy required for the process to occur.

MD can be combined with *ab initio* methods (*ab initio* molecular dynamics (AIMD)) to record valuable information on the encapsulation process of guest molecules. Thus, AIMD enables the determination of dynamic processes over a wide *T* ranges while accurately elucidating the structural features and mechanisms involved in the confinement process.

### 4.4 Finite Element Method (FEM)

The FEM is a numerical technique that allows the modeling of complex and continuous systems by solving partial differential equations, considering that the system is formed by a finite number of discrete domains. In the field of CNTs filling, it has been used to simulate the electric,^141^ thermal, and mechanical properties of the emerging hybrids using molecular mechanics (MM), which enables the calculation of parameters such as Young’s modulus, stress, strain, or deformations induced either to the host or the filler. The FEM is particularly useful for predicting the properties of composites formed by filled CNTs. However, this approach has important limitations, as it does not allow for molecular-level analysis of processes. As consequence, it is usually employed as a complementary approach to predict the macroscopic behavior of the composites, in order to improve their properties.^142^

Despite the scarcity of reports on simulation-filled CNT systems using FEM, this approach has tremendous potential for understanding the properties of composites formed by these heterostructures. FEM provides certain advantages over other theoretical approaches. These include the ability of FEM to incorporate the analysis of multiple parameters, such as the geometry or ratio of components, and the simulation process requires relatively short periods of time.

### 4.5 Machine Learning (ML)

ML has emerged as a valuable artificial intelligence (AI) tool that can accelerate research on functional material design. ML takes advantage of a set of algorithms able to learn patterns and trends from data that can be recorded from available reports on experimental research or even from predictive models such as MD, DFT or QMC.^143^ The accuracy of ML models depends on the quality of the algorithms designed for recovering the data set, namely their ability to find, store, properly process and learn from a large volume of information, which may be reliable.^144^

The ability of ML algorithms to acquire and analyze such enormous amounts of information places this AI tool at the forefront of nanotechnology research. ML offers compelling advantages over the available predictive methodologies, being able to efficiently develop time- and resource-consuming tasks, identify complex patterns and trends, and continuously “learn” from the already performed research (both experimental and theoretical) to understand the behavior of systems, role of parameters, and physical and properties of entities involved in the processes. This confers ML the possibility to design functional materials, propose optimized protocols for synthesis, and predict their electronic and mechanical properties.^144^ In recent years, multiple articles have been published on using ML for developing new functional systems by exploiting the properties of CNTs,^145^ however, despite its demonstrated potential to contribute to the field, few reports have been devoted to using this tool to study the filling process. The available reports consist in the analysis of the encapsulation of fullerenes and their role in the modulation of the thermal transport properties of the system.

For ease of discussion and understanding the role of theoretical approaches on the advances in the field of encapsulation of foreign species within the cavity of CNTs, here, we have incorporated a variety of examples of articles describing the use of theory and simulation approaches for the research in this area.^128^

## 5 Methodologies for Filling CNTs

Soon after Iijima’s seminal paper on the preparation of MWCNTs, extensive efforts were devoted to studying the structural features of this carbon allotrope. Pristine CNTs can undergo different chemical transformations, providing room for novel and unprecedented properties to be discovered. These transformations can occur on the surface of CNTs (exohedral functionalization, coating), within the CNT lattice (doping), or inside their cavities (endohedral filling). The original interest in the preparation of filled CNTs, also known as hybrid CNTs, was to use them as templates for the preparation of nanostructured materials. However, in recent years, efforts have been devoted to filling CNTs with different organic and inorganic species to modify the chemical and/or physical properties of both the host and encapsulated material.

The first reports on the filling of SWCNTs date back to 1998. Smith et al.^146^ reported the *in situ* containment of fullerene molecules in purified CNTs produced by pulsed laser vaporization (PLV), as demonstrated by high-resolution transmission electron microscopy (HRTEM). They also observed that some of these C_60_ molecules were assembled in a linear fashion with similar center-to-center distances, thus resembling a nanoscopic “peapod”, a term that is currently used to identify CNTs filled with fullerenes. Using a completely different approach, Green et al.^147^ reported the first deliberate attempt to fill CNTs using a wet chemistry technique, wherein a saturated solution of RuCl_3_ was mixed with open-ended SWCNTs. The final encapsulated material was identified as Ru (M), as confirmed by electron micrographs and energy-dispersive X-ray (EDX) spectroscopy analysis. Whereas initial efforts were focused on filling MWCNTs, most reports in modern literature are related to the filling of SWCNTs, as their structural characteristics resemble a 1D nanocontainer more realistically than MWCNTs. Nevertheless, filling SWCNTs is not straightforward because their diameters are smaller than those of MWCNTs, and they are prone to forming bundles, which makes the filling process even more difficult. The available methodologies for filling CNTs can be divided into two main groups according to the synthetic approach, as summarized in Figure 2.

**Figure 2:**
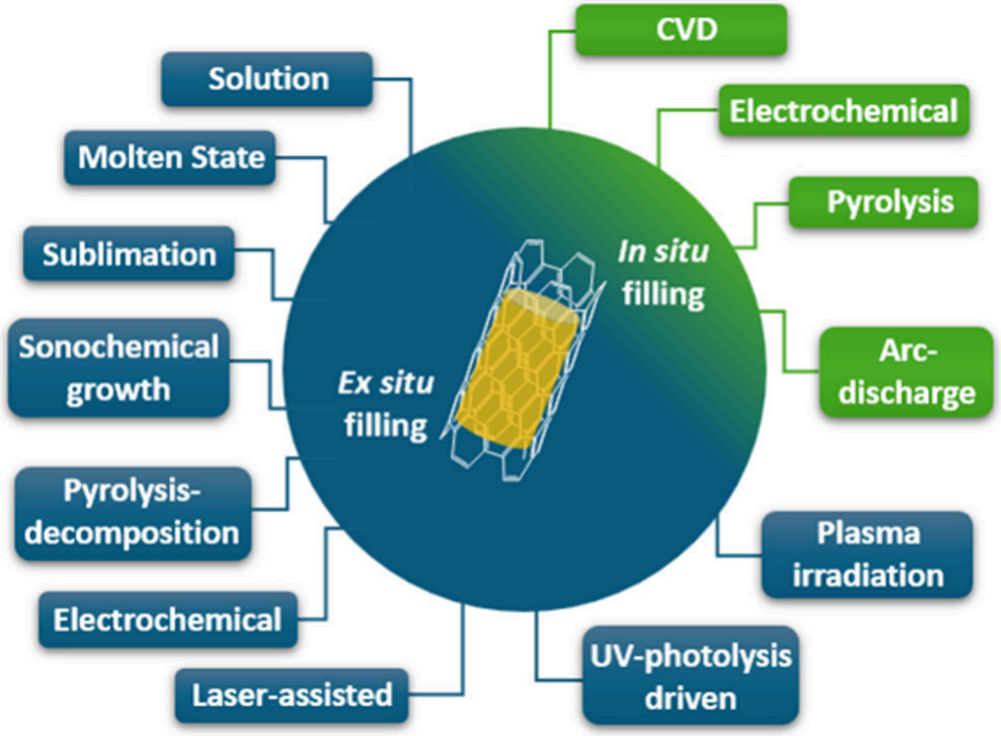
Main strategies employed for the encapsulation of guest species into the inner cavities of CNTs. Foreign materials can be incorporated into CNTs simultaneously during their synthesis (*in situ* filling) or in a subsequent step (*ex situ* filling).

Some considerations have been established for selecting the methodology employed for encapsulating foreign materials within the inner cavity of CNTs. The nature of the material to be encapsulated plays a significant role. Each approach has its advantages and disadvantages, significantly impacting the number of steps and time required for filling, resulting filling yield, and nature, quality, and long-term stability of the obtained hybrid.

### 5.1 *In Situ* Filling

Although this methodology is rarely used nowadays, *in situ* filling represents a pioneering route for the endohedral functionalization of CNTs. Its main advantage is that the filling process occurs during CNTs synthesis; therefore, both CNT growth and encapsulation of the chosen payload are carried out in a single step. Unlike other synthetic approaches, in most *in situ* methodologies, the encapsulated material plays a double role: acting as a catalyst for the formation of the CNTs, which usually grow as closed-ended rolled-up graphene sheets around the inorganic precursor, and simultaneously serving as the filler, remaining confined within the CNTs. Because both the filling material and CNTs grow simultaneously, these protocols usually result in a high compatibility between the guest and host, leading to a hybrid system with excellent stability. Therefore, *in situ* approaches were considered suitable alternatives to promote the formation of hybrid systems in which the CNTs stabilize and protect guest entities susceptible to degradation or transformation, including those whose structure, formed at the nanoscale, is unusual under ambient conditions. Unfortunately, this method is limited to a few materials, usually those that are compatible with strong reaction conditions, such as high *T* or significant variations in the EC potential of the system. Therefore, these approaches have mainly been employed for the encapsulation of compounds involved in the synthesis of CNTs. It is worth noting that the as-produced CNTs might contain catalyst particles entrapped within graphitic shells or other compounds employed during their synthesis. These are generally regarded as impurities that need to be removed, depending on the targeted application. For instance, the presence of catalytic particles can dominate the EC response of a material.^148,149^

One of the major challenges of in situ approaches is the lack of control over the characteristics of the synthesized materials. The quality of the CNTs depends on the technique employed for their synthesis. For instance, some CVD-based approaches lead to CNTs with a wide diameter distribution and the formation of side products such as amorphous carbon and catalyst residues external to the CNTs. These should be removed through subsequent purification steps. The homogeneity of the sample is an important issue as well. These protocols usually produce mixtures of empty and filled CNTs, with relatively low overall filling yields compared to their *ex situ* counterparts. However, when filled CNTs are formed, the confined materials are relatively stable and highly pure, and usually consist of a long crystal that extends along the inner cavity of the CNT, even reaching microns in length.

The synthesis conditions certainly determine the morphology and composition of the encapsulated compounds. In general, AD and CVD syntheses that include ferromagnetic metals (Fe, Co, and Ni) to promote CNTs growth lead to CNTs either filled with highly crystalline and long metal NWs^150^ or with metal NPs.^151^ Otherwise, when using their metal alloys (FeCo, NiCo, or NiY), the formation of single-crystal alloys within the cavities of the CNTs is observed. Transition and rare-earth metals catalyze the formation of metal carbide filled CNTs,^152^ while a transition metal oxide catalyst used in CVD synthesis, such as V_2_O_5_, was directly confined within the CNTs or stabilized as their metal carbide derivative.^153^

It has been reported that the production of filled CNTs by the AD method can be favored by introducing covalent species such as sulfur. Demoncy et al. evaluated the role of S, initially present as an impurity (∼0.25%) in the graphitic electrode, which in some cases was incorporated inside the CNTs along with the transition metal, forming the corresponding M–S bond. Sulfur enhances the catalytic activity of metals such as Co or Ni, and favors the graphitization of carbon materials. Moreover, its affinity for both metals and carbon promotes the encapsulation of S inside the CNTs in the molten state and the stabilization of the resulting hybrid.^152^

Among others, Pd nanoparticles (NPs) were encapsulated *in situ* into CNTs using a simplified AD method in solution.^154^ CVD has also been used for the *in situ* filling of CNTs with iron using different precursors, including ferrocene (FeCp_2_), acetylferrocene (acFeCp_2_), and iron nitrate.^155^ Analogously, the pyrolysis of dimethyl sulfide over a Co/MgO catalyst resulted in the formation of Co_9_S_8_ NPs, nanorods (NRs), and NWs, which were encapsulated within CNTs.^156,157^

In addition to AD and CVD, an EC approach has been reported for the simultaneous synthesis of CNTs and their filling with metallic Sn. Here, the authors took advantage of the intercalating capacity of Li (from a LiCl molten salt) to induce the exfoliation of cathodically polarized graphite in a system containing SnCl_2_. Thus, while graphite layers were released from the electrodes, Sb was simultaneously deposited and encapsulated within the inner surfaces of the newly formed tubular nanostructures. Although the protocol seemed to be very successful in terms of filling yields, the authors found the presence of O_2_ in the determined areas, apparently due to the partial oxidation of Sb. XRD analysis revealed the presence of SnO, SnO_2_, and occasionally Li_2_SnO_3_.^158^

### 5.2 *Ex Situ* Filling

The first report on filled CNTs was a theoretical study published in 1992.^159^ One year later, the *ex situ* filling of MWCNTs was experimentally achieved,^160–164^ thus paving the way for a more detailed study of the inclusion of guest molecules and compounds into the CNTs’ cavities. In the case of *ex situ* methodologies, a pretreatment of CNTs to open their ends might be required to allow the diffusion of the guest material into their inner cavity. *Ex situ* filling is used more frequently than the *in situ* approach and allows the encapsulation of a large number of compounds. Among the wide range of developed *ex situ* approximations, solution, molten state, and sublimation methods have taken the lead and are discussed in greater detail.

#### 5.2.1 Solution Filling

The solution-filling method involves mixing a high concentration of the material to be encapsulated with CNTs in a suitable solvent. The versatility of solution-phase methods offers the possibility of including several guest molecules in the hollow cavities of CNTs. This method represents one of the main strategies currently being explored for filling CNTs. The filling process normally occurs at mild *T* and in open air or non-oxidative saturated atmospheres, and is a suitable alternative for filling the inner cavity of CNTs with materials that can decompose under harsh reaction conditions, such as high *T* treatments. Owing to the solvation of the molecules entering the CNTs, the efficiency of the encapsulation is reduced; therefore, filling yields below 50% are usually achieved using this method. The filling yield decreases with the diameter of the CNTs used; thus, filling SWCNTs or DWCNTs using this method is less efficient than filling MWCNTs.

The filling of MWCNTs using wet chemistry methods was first reported by Tsang in 1994 using a Ni­(NO_3_)_2_ solution under reflux conditions in the presence of concentrated HNO_3_.^156^ The proposed approach has the advantage that both the opening and filling of the CNTs occur simultaneously under these conditions. The main drawback is that the use of a strong oxidizing acid limits its application to a few compounds only. Instead, a two-step procedure (opening/filling) has been widely employed, in which the ends of the CNTs are initially opened using HNO_3_ or another reagent that removes the ends. In 1998, Sloan et al. reported this approach for the first time using RuCl_3_ as the filling agent, which was reduced to Ru (M) under the synthetic conditions.^147^ Currently, this two-step procedure is widely used for the encapsulation of various materials, including organic and inorganic compounds.

As mentioned above (see Section 2), filling CNTs in the liquid phase is strongly governed by capillary forces. To be successfully loaded within the CNTs’ cavities, the solution containing the foreign material should effectively wet the inner surface of the CNTs. A high compatibility between the vehicle solvent, filling agent (solute), and hosting platform (often hydrophobic) is therefore required, which leads to strong interactions between the reactants. Thus, the selection of solvent plays a crucial role in the formation of new CNT hybrids. Aspects such as polarity, solvation degree of the solute, concentration,^42^ and pH^165^ determine the filling yield and mechanism of encapsulation, and may also play a role in their containment and/or release. In general, high filling yields are observed when the filling agent is dissolved at high concentrations in non-polar solvents with low surface tension. Finally, a careful selection of the hosting platform should be made because the dimensions and structural configuration of the filler material can also affect its diffusion into the CNTs cavities.

Solution filling may also promote the release of previously encapsulated molecules from CNTs interiors. As a result, it can serve as an active driving force to stabilize or remove the encapsulated guests,^41^ which is useful in controlled-release systems. An additional modification of the wet-filling protocol includes the elimination of the solvent by sublimation, which ensures a good distribution of clusters and size control along the cavities of the CNTs.^166^ This will lead to establish strong interactions between the reactants.

#### 5.2.2 Molten State Filling

In this method, the material to be encapsulated diffuses into the hosting cavity of the CNTs in the molten state via the capillary forces. Thermally stable and non-reactive filling agents with congruent melting behavior and high wettability under working conditions are required. Consequently, this approach is limited to inorganic materials, such as metals, alloys, or stable inorganic salts, which can withstand high *T* conditions. The filling process can be expressed in terms of the Laplace equation, which correlates the pressure of the system with the surface tension (γ) of the molten compound and the inner dimensions of the nanotubes, expressed in terms of their radius (*R*):

$$\Delta P=\frac{2\gamma \cos\theta }{R}$$

Moreover, their melting points should be compatible with the thermal stability of CNTs to avoid structural damage. Since the filling process generally occurs at high *T* (above the melting point of the filler), the prepared mixture of CNTs and the selected guest should be maintained under a non-oxidative atmosphere (generally keeping the sample under vacuum or an inert gas, e.g., Ar), to avoid undesired decomposition or transformation.^167^ To improve the quality of the resulting material, the synthetic parameters can be modified. In particular, slow annealing and cooling rates favor the controlled diffusion of the guest and the formation of highly ordered crystals within the CNTs, respectively. Using this method, higher filling rates than those achieved using solution methods are typically observed. Foreign materials usually grow as long and continuous systems with stable morphologies, such as NWs or NTs, protected from oxidation and decomposition owing to confinement within CNTs’ cavities. Consequently, this is probably the most widely used approach for filling SWCNTs with all kinds of materials, apart from peapods.

Initial studies using the molten-phase (MPH) filling method led to the formation of inorganic NWs within the cavities of MWCNTs, starting with molten metals and their compounds. Pioneering attempts have included the encapsulation of Y_3_C,^168^ Ni,^169^ or Bi_2_O_5_.^163^ In 1998, Grigorian et al.^170^ reported the intercalation of charged polyiodine chains into the interstitial channels of SWCNT bundles. Two years later, the same authors reported the encapsulation of iodine (as a source material) into SWCNTs by MPH filling.^112^ Afterward, Sloan et al. reported the use of this method for the encapsulation of a wide range of materials into SWCNTs, including AgCl, AgBr, KCl, and UCl_4_.^171^

#### 5.2.3 Sublimation Filling

This method involves filling the CNTs by heating the system to the sublimation *T* of the guest species. This approach, which can also be considered a gas-phase filling protocol, allows achieving filling yields of up to 100% without the presence of solvent molecules in the CNT’s cavities, but is limited to a small range of materials, such as I_2_, whose sublimation *T* falls within the range of the thermal stability of CNTs. CNTs may be located in a cooler area of the reaction system, inducing the diffusion and further condensation of the material within their cavities. This process occurs at elevated *T* and strongly depends on the pressure of the system. As in the case of the MPH approach, carefully controlling the reaction conditions guarantees the ordered crystallization of the sublimed compound. Both the length of the confined nanostructure and the filling yield can be controlled by tuning the treatment time, thus leading to highly stable and homogeneous structures, usually consisting of 1D chains or long NWs.

Although the examples in the literature for species successfully encapsulated using this method are scarce, sublimation filling is important because it is still the current preferred method for filling CNTs with fullerenes (formation of “nanopeapods”), with the first reports appearing more than 20 years ago.^172^ Smith et al.^173^ reported on the preparation of SWCNTs with C_60_ chains encapsulated through transport in the vapor phase. In another pioneering study, Hirahara et al. encapsulated 1D metallofullerenes inside SWCNTs [(Gd@C_82_)_*n*_@SWCNTs], with the filled crystals adopting a regular intermolecular distance.^172^ In all cases, the arrangement of nanopeapods was examined using HRTEM. Sublimation filling to encapsulate I_2_ molecules into CNTs has also been well documented.^74,174–176^

#### 5.2.4 Other *Ex Situ* Filling Strategies

Extensive efforts have been devoted to develop efficient approaches for filling CNTs. In all cases, incorporation of the filler was driven by an external force that provoked transformations that improved the reactivity or compatibility between the involved species. The filling yield, quality, and stability of confined compounds are determined by the reaction conditions and precursor characteristics. Examples include the synthesis via pyrolysis-decomposition of a metal precursor, often leading to the formation of metal and metal oxide (M_*x*_O_*y*_) NPs within the inner cavities of the CNTs. This approach can sometimes involve the encapsulation of the metal precursor (or a mixture of metals) under wet and/or high-pressure (hydrothermal synthesis) conditions. The formation of NPs occurs under high *T* treatment in the absence of a reducing agent, and the reaction conditions ensure high compatibility and stability of the filled materials. Examples include the formation of M_*x*_O_*y*_@CNTs using a variety of metal–organic salts, the encapsulation of Fe, Co, and Ni^177^ using dicyandiamide, metal nitrates, or Pt NPs via the decomposition of Pt­(C_5_H_7_O_2_)_2_,^178^ or FeCo and FeNi mixtures.^179,180^ In the case of Pt@SWCNTs, the hybrids were also used as precursors to confine PtI_2_ and PtS_2_, whose formation is not straightforward.^181^

Environmentally friendly approaches, including both liquid- and solid-phase treatments, have been proposed, and they are claimed to have some advantages over traditional methods. These include working under soft synthetic conditions (room temperature, RT), fast reaction times, and high filling yields or selectivity toward the formation of certain structures or morphologies. This is the case of the sonochemical technique reported by Nowak et al., which led to the encapsulation of ternary chalcohalide (SbSI, SbSeI) NWs within MWCNTs, using monoelemental precursors like Sb, S or Se and I;^182,183^ or the almost exclusive formation of tubular MX within MWCNTs, promoted by their laser irradiation in the presence of bulk PbI_2_, which was also tested for ZnI_2_.^19^ Plasma ion irradiation has been also reported to assist the filling of CNTs with Cs metal.^91^ Beside promoting the diffusion of the foreign species inside the NTs, plasma energy improves their compatibility by inducing functionalization of the hosting platform, which not only increase wetting during the filling, but also strengthen the interactions of the filler with the inner surface of the CNTs, thus contributing to obtain systems with large stability.

In a recent study, Mittal et al. reported the simultaneous opening and filling of SWCNTs using UV photolysis, which has been proposed as a good alternative for filling small-diameter hosts at RT.^184^ This single-step methodology, which occurs at RT, takes advantage of the ability of UV light to dissociate certain liquid solvents, in this case CHCl_3_, with the subsequent release of free radicals that promote the opening of CNT tips. The open tubes were subsequently filled with MoCl_5_ and I_2,_ which are highly soluble in the solvent employed during the treatment.^19,184^ Other methodologies include EC deposition to fill CNTs with metal alloys.^185,186^ Using microcurrent electrochemistry, Fu et al. effectively prepared CNTs that were highly filled with Li, Na and K (Ms).^187^ As it has been mentioned above, to select a suitable encapsulation approach, different aspects must be considered. Table 3 shows a comparative analysis of the most common approaches used for the encapsulation of foreign materials within CNTs, considering aspects such as the mechanism, driving process, compatibility of reactants, stability, and quality of the resulting hybrid structures.

**Table 3:** Summary of the Characteristics of the *In Situ* and *Ex Situ* Approaches Most Commonly Used for Filling CNTs

Filling approach	Synthetic highlights	Mechanism	Compatibility between host and filler	Stability & Quality	Filling yield	Control of the resulting hybrid
*In situ filling*						
**CVD, AD, EC, pyrolysis**	• Single-step approach.	Simultaneous growth of the guest and host.	High. Strong interactions between the guest and host.	High. The guest is properly isolated and protected from degradation. Pristine CNTs. are obtained.	Can be high, depending on the growth conditions.	Poor, depending on the growth conditions.
	• Limited to alloys. monoelemental systems, and metal oxides.					
*Ex situ filling*						
**Solution**	Allows confining sensitive species.	Diffusion of the guest (or its precursors) assisted by a solvent.	High. Versatile for various materials. It requires high affinity between the guest, solvent and host.	• High stability. When reactions occur inside the NT, S&Q depend on the properties of the newly formed guest.	Variable, depending on the affinity of the reactants and the reaction efficiency. Sensitive to the host diameter.	Variable. Good over composition and morphology. Poor for phase and length.
				• Low to moderate crystallinity of the filler.		
**Molten state**	• Solvent-free method.	Capillary filling.	High. Limited to low-surface tension liquids.	Variable, depending on the properties of the filler. It requires free oxygen conditions to preserve the integrity of the CNTs.	Can be high.	Guest morphology is limited to the tubular structure. It can be controlled by tuning the synthesis conditions.
	• Limited to filling agents with low melting points.					
**Sublimation**	• Limited to filling agents with low sublimation points.	Diffusion of the guest in the vapor phase.	High. For thermally stable guests.	High for thermally stable guests. It requires free-oxygen conditions to preserve the integrity of the CNTs.	Can be high.	High. Good over composition and morphology.
**UV-Photolysis driven**	• Occurs at RT.	Photochemical reaction of the guest precursor and/or photo-oxidation of the CNT tips.	High. Depending on the solubility of the precursor and its photochemical reactivity.	• High stability due to RT processing. Non-equilibrium structures formed inside the host.	Low to Moderate, depending on the efficiency of the photo-opening and photolysis reaction.	Low. Limited to irradiation time and precursor type.
	• Limited to photoreactive fillers/precursors.			• Low to moderate crystallinity of the filler.		
	• Simultaneous tips opening and filling.					
**Sonochemical growth**	• Fast.	Capillary wetting enhanced by acoustic cavitation induced by high *T* and pressure.	Hydrodynamic forces overcome poor wetting, enabling compatibility with a wider range of solutions.	Low to moderate crystallinity of the filler. Formation of short and granular deposits.	Moderate to High owing to strong driving forces.	Poor owing to difficult control of conditions (time and sonication power).
	• Single-step method.					
	• Simultaneous surface modification and filling.					
**EC**	• Mild operating T conditions.	Electrodeposition.	Variable-It depends on the ability of the guest to electrodeposit inside the host.	High for stable phases formed during the deposition.	Variable. Depending of the deposition parameters.	High. Good over deposition rate, composition, and loading.
	• Requires conductive CNTs.					
**Laser-assisted**	• Ultra fast.	High *T* induced by laser irradiation.	High.	High. The guest is properly isolated and protected from degradation.	High. Depending on laser fluences.	High. Selective for the formation of hollow tubular nanostructures.
	• Requires high fluences and vacuum conditions.					
**Plasma-assisted**	• Solvent-free.	Decomposition of precursor assisted by plasma irradiation.	High. Owing to wetting improvement by induced functionalization.	• Moderate. Stable and pure crystal NWs.	Variable for direct filling.	Good for the growth of single-crystal NWs. Limited to plasma composition, power and time.
	• Requires high *T* and vacuum conditions.			• Precise control over synthetic conditions is required to prevent CNTs damage.	High for subsequent functionalization/doping	

## 6 Characterization of Filled CNTs

Proper characterization of the filled CNTs is crucial for determining the properties of the emerging hybrids. In most currently available non-destructive characterization methods, an external stimulus, such as light, is used to evaluate the characteristics of the material. CNTs are relatively stable against these stimuli; therefore, the selection of the evaluation method should consider the properties and stability of the filling agent. Different fillers, such as metals, metal oxides, and organic compounds, require distinct analytical approaches, each with specific limitations and advantages. To determine how the filling material modifies the properties of the host, different aspects need to be analyzed, namely, the structure and composition of the filler, morphology and filling yield, and finally, the chemical and physical properties of the system.

Electron microscopy (EM) is the most widely used characterization tool for confirming the encapsulation of foreign materials within CNTs. Both transmission electron microscopy (TEM) and scanning electron microscopy (SEM) allow visual inspection of the sample, quantification of the filling yield, and assessment of the dimensions of the CNTs and morphology of the guests. Moreover, Selected Area Electron Diffraction (SAED) provides atomic-level information on the crystal structure of the confined compounds. Modern microscopes with high spatial resolution (nm to sub-nm scale) enable the determination of the structure, crystallinity, atomic location, and presence of defects. One particular advantage of this technique is the ease of coupling of microscopes with other complementary tools for simultaneous spectroscopic characterization. Thus, using EDX, one can take advantage of the X-rays emitted from the sample to determine its chemical composition. Spatial maps showing the atomic distribution of the filler can also be built. Finally, by measuring the energy lost by the electrons that interact with the sample, Electron Energy Loss Spectroscopy (EELS) can be performed. In addition to elemental composition determination, EELS provides information about the chemical bonding, oxidation states, and electronic properties, making it a powerful tool for predicting the properties and further applications of the analyzed materials.

Spectroscopic techniques enable the analysis of the electronic, optical and vibrational properties of filled CNTs.^188^ Raman spectroscopy and Fourier Transform Infrared (FTIR) spectroscopy take advantage of the molecular vibrations induced by the interactions of the sample with specific wavelength incident light. While FTIR is particularly useful for analyzing organic and polymeric fillers, providing information about the surface functionalization of the hosting CNTs, Raman spectroscopy allows the determination of modifications to the electronic environment induced by encapsulation. Therefore, shifts in the frequencies of the characteristic Raman bands can be associated with electronic transitions (from metallic to semiconducting), charge transfer, doping, or strain induced by encapsulation. Moreover, the electronic band modification of semiconducting CNTs can be studied using Optical Absorption Spectroscopy (OAS), because encapsulation can induce shifts in the peaks generated by electronic transitions between the valence and conduction bands, which occur in both the NIR and the Visible/NIR region (Ultraviolet–Visible–Near-Infrared (UV–vis-NIR) Absorption Spectroscopy). Finally, photoluminescence excitation (PLE) mapping is a powerful technique for characterizing CNTs when photoluminescent compounds are employed.^13,189^ Other complementary techniques include X-ray Photoelectron Spectroscopy (XPS) for the elemental, binding, and electronic assessment of the sample, Thermogravimetric Analysis (TGA) for the quantification of the filler and sample purity, and magnetic measurements such as SQUID or magnetometry to determine the filling yield and the magnetic behavior of the hybrids when these present magnetic response.

Advanced approaches involving real-time monitoring techniques are increasingly recognized as essential for enhancing the precision, uniformity, and reproducibility of CNT-filling processes. These methods enable direct observation or indirect detection of changes during encapsulation, allowing researchers to fine-tune the process parameters and ensure better control over the quality and consistency of the resulting nanostructures. *In situ* TEM has emerged as one of the most powerful tools for this purpose. This enables the real-time visualization of capillary-driven filling and structural transformations at the atomic or nanoscale level.^190^ This technique allows direct observation of how filler materials enter the CNT’s cavity, interact with the inner walls, and respond to changes in *T*, pressure, or chemical environment.^191^ Such dynamic visualization provides critical insights into the kinetics of the filling process, possible interface formation, and morphological evolution, which are difficult to access via post-synthesis analyses. In addition to *in situ* TEM, quartz crystal microbalance (QCM) methods have been employed to monitor mass uptake during vapor- or gas-phase filling.^192^ Moreover, QCM can be employed to study the adsorption kinetics and saturation behavior of CNTs. However, one of the limitations of QCM is its sensitivity to external environmental factors such as *T*, pressure, and humidity. These variables can cause significant frequency fluctuations, potentially affecting the accuracy and reproducibility of measurements.

Synchrotron X-ray based techniques have emerged as powerful tools offering localized and high-resolution characterization, owing to the access to bright, tunable and coherent energy sources. These complementary techniques, which include X-ray Absorption Spectroscopy (XAS), synchrotron X-ray Diffraction (SXRD), Small-Angle X-ray Scattering (SAXS), Wide-Angle X-ray Scattering (WAXS) and X-ray Fluorescence (XRF) mapping, allow the characterization of the crystal and electronic structure, chemical environment, composition, size, and elemental distribution, taking advantage of the benefits offered by synchrotron light, which provides a superior signal-to-noise ratio, orientation mapping, and fast data acquisition. Synchrotron-based techniques require a low amount of sample and are ideal for the assessment of properties at the nanoscale, which is challenging with laboratory-based facilities. X-ray Magnetic Circular Dichroism (XMCD) measurements, for instance, overcome the limitations of bulk magnetic measurements of SQUID, enabling the quantitative determination of the magnetic moments of the confined system. XMCD is a selective-to-element technique that clearly identifies whether the magnetic signal originates from the encapsulated system, the host, or other species present in the sample. Additional information can be provided by XMCD, including the effect of the host–guest interaction on the magnetic properties, Curie temperature, and coercivity of the magnetic phase.^193^ Synchrotron based techniques also enable for *in situ* and operando experiments. Along with the acquisition of a large amount of experimental data, real-time monitoring of the transformations that the sample undergoes under external stimuli, such as heating or different environments, facilitates the assessment of different properties and mechanisms, including thermal stability, phase transitions, surface behavior, and reaction kinetics.

### 6.1 Challenges in the Characterization of Filled CNTs

The accurate characterization of filled CNTs remains complex and challenging. Primary difficulties stem from the nanoscale dimensions of the material, the broad variety of filling materials, the influence of environmental and processing conditions, and the limitations intrinsic to most standard characterization methods.

As mentioned above, for the initial screening of filled CNTs, conventional imaging techniques such as TEM and SEM are frequently employed. Nevertheless, owing to the low atomic number contrast between the carbon walls and many encapsulated materials, particularly organic compounds or light elements, it is often difficult to confirm the presence of the filler. High resolution Scanning Transmission Electron Microscopy (HRSTEM) offers improved spatial resolution and can reveal the structure of crystalline fillers; however, interpretation is still complicated by factors such as beam-induced damage, sample instability, and orientation-related artifacts.^194^ In the case of EELS, high-vacuum conditions and continuous exposure to a high energy e-beam (usually 200- 300 kV energy) are required for a high signal-to-noise ratio. Consequently, foreign species under the beam can undergo melting, vaporization, or even decomposition, leading to misinterpretation of the morphology and composition. EELS is also sensitive to compositional overlap, owing to ionization edges of different elements can occur at similar energies, thus limiting the proper detection if coincident species are simultaneously present in the sample.^195,196^

Sample preparation is a critical factor that might significantly affect the characterization by imaging techniques. Owing to the ultrathin samples required to allow the transmission of electrons through the sample in TEM, the preparation methods involve the dispersion of the sample in different solvents using processing tools such as ultrasonication or stirring, drop-casting or drying, which may inadvertently damage the sample, remove or redistribute the encapsulated material, potentially leading to inaccurate results. Special attention should be paid to processing CNTs filled with species sensitive to oxidation or highly reactive species that can interact with the solvent or require storage in an oxygen-free environment.

Spectroscopic techniques, such as Raman spectroscopy,^84^ which are often employed as indirect methods to support evidence of encapsulation, especially in the case of metallic or magnetic fillers, also present significant limitations that should be considered. Raman analysis from the filler is frequently affected by the dominant signals from the CNT walls, while in case of PLE mapping, the interpretation of the results must be approached with attention, as the CNTs walls can significantly modulate the intrinsic photoluminescence (PL) of the encapsulated molecules.^197^ One particular challenge for the PL evaluation of CNTs is the invisibility of metallic specimens, therefore being useful only for the characterization of electronically differentiated filled CNTs (e.g., semiconducting SWCNTs). Moreover, if the encapsulated material induces quenching, the PL signal can be suppressed, which occurs in the presence of highly polar species or metals.^189^ Several factors can influence the observed signals, including the CNT diameter and chirality, the presence of defects or contaminants, and the orientation and interaction of the host molecules within the CNT’s cavity.

Quantifying the filling efficiency, which involves determining the volume or mass fraction of the material successfully encapsulated within the CNT’s cavity, remains challenging. The heterogeneity in the degree of filling among individual CNTs is a major issue. Variability in tube length, diameter, contaminants, and wall structure often leads to non-uniform encapsulation. Therefore, a statistically significant analysis across a large number of CNTs is required to reach reliable conclusions, making the process time-consuming and technically demanding. Observations from individual CNTs, although informative, may not be representative of the overall sample without statistical validation of the results.

Although TGA and inductively coupled plasma mass spectrometry (ICP-MS) can quantitatively determine the filler content, they are destructive and do not provide spatial information. Therefore, the presence of an external material on the CNTs’ sidewalls or other contaminants, such as residual catalysts used during the synthesis of the CNTs, can significantly affect the results. In the case of TGA, the accurate quantification of the filling yield also depends on the properties of the filler and the overall stability of the sample. Transformations occurring during the combustion of the sample and possible side products resulting from interactions of the species present might lead to the formation of thermally stable compounds, such as metal carbides, which contribute to the final residue. Given these limitations, no single technique can provide a complete analysis of the filled CNTs. Instead, a combination of complementary methods is typically required to obtain an accurate and reliable characterization.

Despite the benefits offered by synchrotron light, experimental and operational challenges remain for characterizing filled CNTs. The main issue is the limited access to the highly complex conditions required to perform these experiments. Extensive planning and, in general, the application and approval of a scientific proposal are required to access synchrotron beamtime (often constrained) in different public facilities. Moreover, owing to their high costs, these techniques are far from routine laboratory practices. The technical challenges during the execution of the experiments include complex preparation, specialized experimental setups, and difficulties in analyzing individual specimens, therefore, most experiments are based on bulk powdered samples. Otherwise, the analysis of the acquired data is not straightforward. Saturation and self-absorption artifacts usually result from analyzing the low-energy edges of elements such as C (NEXAFS), making quantification challenging. In some cases, complex theoretical models and fittings should be applied to decipher the obtained spectra.^198^ The characteristics of the sample may also affect its response to the incident radiation. Crystal size heterogeneity or localized strain within the CNTs’ cavities can induce for instance broadening of the diffraction peaks owing to the contribution of multiple environments. Finally, to avoid beam-induced damage to the sample by the high-flux high-energy synchrotron source, the conditions employed to perform the experiments must be carefully selected. Localized heating may alter the molecular states and even induce undesired transformations, vaporization, and decomposition, especially when *in situ* measurements are performed.

Table 4 summarizes the characterization techniques that provide useful information for evaluating the properties of filled CNTs.

**Table 4:** Summary of the Most Common Techniques Used for the Characterization of Filled CNTs

Characterization Technique	Principle	Provided information	Advantages	Limitations
**TEM HRTEM**	Interaction of the sample with an accelerated e-beam. The image is created using e^–^ transmitted through the sample.	Direct:	• It has high spatial resolution (nm to sub-nm scale).	• It requires extremely thin samples
		• Size and morphology of both the guest and host.	• It requires low amount of sample.	• The e-beam can damage the sample.
		• Crystal structure (imaging and ED).	• It can be coupled with spectroscopic techniques to provide compositional information. (EDX, EELS).	• It is sensitive to contamination.
		• Composition.		
		Indirect:		
		• Filling yield (calculated from visual inspection).		
**SEM/FESEM**	Interaction of the sample with an e-beam. The image is created using secondary and backscattered e^–^.	• Surface morphology.	• It requires a low amount of sample.	• It has lower resolution than TEM.
		• Topography.	• Its relatively easy sample preparation.	• There are lacks in structural assessment.
			• It allows the determination of composition when coupled with EDX.	
**EDX**	X-rays emitted from the sample after interaction with e^–^ provide elemental composition information.	• Composition.	• It provides elemental composition.	• It has limited sensitivity to light elements.
		• Semiquantitative elemental analysis.	• It is relatively fast.	
		• Elemental distribution (mapping).		
**EELS**	It measures the energy lost by electrons as they pass through a thin sample, corresponding to electronic transitions.	Elemental identification.	• It has high spatial resolution (down to atomic scale).	• It requires extremely thin samples
		• Chemical bonding and oxidation states: Fine structure in core-loss spectra (e.g., C K-edge, N K-edge) reveals hybridization (*sp* ^2^, *sp* ^3^), bonding environment, and oxidation states of elements.	• It is sensitive to light elements (e.g., C, N, O, B, Li), which are often difficult for EDS.	• Sample preparation is critical and can introduce artifacts.
		• Electronic structure: Analysis of plasmon excitations (low-loss region) provides information on band structure, optical properties, and charge carrier density.	• It provides detailed chemical bonding and electronic structure information.	• Interpretation of complex spectra can be challenging.
		• Specialized equipment is required (spectrometer, monochromator).	• It can be combined with TEM/STEM for correlative imaging and spectroscopy.	• It can be susceptible to beam damage for certain materials.
				• Spatial distribution: Elemental and chemical state mapping with atomic resolution.
**FTIR**	It measures the absorption of IR radiation by molecular vibrations.	• Functional groups on CNT surface.	• It is non-destructive.	• It provides bulk information.
		• It can detect some organic or inorganic filler materials with characteristic IR absorption bands.	• It is relatively simple and fast.	• It is less sensitive to the CNT backbone itself than Raman.
		• Guest−host chemical interactions.		
**Raman Spectroscopy**	Inelastic scattering of monochromatic light by molecular vibrations.	• Crystal lattice.	• It is non-destructive.	• It provides less direct information about the filler, which should be Raman active.
		• Electronic interaction between the filler and CNTs.	• It is relatively fast.	
		• Composition if the filler is Raman active.	• It is sensitive to structural changes and defects.	
**PLE mapping**	It measures the light emission resulting from the excitation-relaxation process of an electron–hole pair (exciton).	• Electronic structure.	• It is non-destructive.	• It is limited to semiconducting samples.
		• Chirality.	• It is relatively fast.	• It requires highly dispersed samples.
		• Electronic impact of the filler.	• It is highly sensitive to electronic environment.	• Poor solid-state performance.
			• Direct readout of interaction.	
**XRD**	The X-rays diffracted by the sample produce a diffraction pattern.	• Crystalline structure of the material.	• It is non-destructive.	• It requires a crystalline material.
		• Lattice parameters and interlayer spacing.	• Sample preparation is not required.	
**XPS**	The kinetic energy of the photoelectrons ejected by X-ray irradiation is measured.	• Surface elemental composition and chemical states (e.g., oxidation states) of the filler and CNTs.	• It is surface sensitive (top few nm).	• It requires delicate measurement conditions (UHV).
			• It provides chemical state information.	• Bulk composition determination.
**TGA**	*T*-dependent weight variation.	• Filling yield.	• It is quantitative.	• It provides bulk information.
		• Purity and homogeneity of the sample.	• It is relatively simple and fast.	• Destructive.
		• Thermal stability.		
**QCM/QCM with Dissipation Monitoring (QCM-D)**	It measures changes in the resonant frequency and/or dissipation of a piezoelectric quartz crystal as mass adsorbs onto or desorbs from its surface.	• Mass uptake: Measurement of the mass of filler materials or other molecules adsorbing onto or being released from CNTs deposited on the crystal.	• Real-time measurement: Dynamic monitoring of surface processes.	• The sample need to be immobilized on the crystal surface.
		• Adsorption kinetics: Rate and mechanism of filler integration or surface functionalization.	• High sensitivity: Nanogram-level mass detection.	• Analysis of results can be complex.
		• Viscoelastic properties (QCM-D).	• Operation in various environments.	• It Can be sensitive to non-mass related changes (e.g., *T*, density of surrounding medium).
		• Film thickness/density.	• Label-free detection: Does not require tags for detection.	
**Magnetic Measurements (e.g., SQUID Magnetometry)**	Response of a material under an external magnetic field.	• Filling yield (magnetic materials).	• It is highly sensitive to magnetic materials.	It is limited to magnetic materials.
		• Magnetic behavior.	• It is quantitative to magnetic filler.	
**Synchrotron-based X-ray Techniques**	Response of a material under highly intense, tunable, and coherent X-rays from a synchrotron source.	• SAXS: Size, shape, and distribution of filler and CNT bundles in the nanoscale range.	• High flux/brightness: Enables rapid data acquisition.	• It requires access to complex facilities.
		• WAXS: Crystallinity and lattice parameters of CNTs and filler.	• It requires low amount of sample.	• The data analysis can be complex and time-consuming.
		• XAS: Electronic and local atomic structure (coordination, oxidation state) of specific elements in the filler and at the interface.	• Tunable energy: Allows element-specific probing of the sample.	• High operational costs.
		• XMCD: Element-specific magnetic properties.	• High spatial/temporal resolution.	
		• X-ray Tomography: 3D imaging of the structure and distribution of the filler within CNT networks.	• Penetrative: Can probe buried interfaces and bulk structures of materials.	
			• Enables for in situ and operando experiments.	
***In Situ*** **Characterization Techniques (e.g.**, ***in Situ*** **TEM**, ***in situ*** **Raman**, ***in situ*** **XRD)**	Real-time observation of materials under controlled external stimuli (T, pressure, gas environment, electrical/magnetic field, mechanical stress and chemical reaction).	• Dynamic processes: How the filler enters CNTs, phase transitions of the filler, and changes in the CNT structure due to filling or external stimuli.	• Direct evidence of dynamic processes.	• It requires specialized sample holders and environmental cells for the measurements.
		• Stability: Real-time monitoring of the thermal, chemical, or mechanical stability of the sample.	• Structure–property relationships under relevant conditions are established.	• It requires complex and extensive data acquisition.
		• Reaction mechanisms: Understanding the formation or transformation of the filler inside CNTs.	• It minimizes artifacts from ex situ measurements.	
		• Performance correlation: Direct correlation between structural changes and functional properties (e.g., electrical conductivity under strain).		

## 7 Encapsulation of Different Materials into the Inner Cavity of CNTs

Since the publication of the first reports on the filling of CNTs, approximately 30 years ago, hundreds of manuscripts on the encapsulation of a variety of species (atoms, molecules, and extended structures) in the hollow cavity of CNTs have been reported in the literature. The tremendous interest in the properties and applications of CNTs (empty and filled) has led to an almost exponential increase in the number of publications in the past few years, highlighting the importance of finding new compounds that can successfully enter the CNT cavity to further expand their range of applications. As mentioned before, the initial interest in the synthesis of the so-called “hybrid CNTs” was to use the inner cavity of the carbon nanomaterial to confine different species in a quasi-1D space, creating novel and unprecedented structures.^48^ This has attracted the attention of chemists, materials scientists, and physicists, interested in the design and synthesis of novel hybrid materials with unprecedented properties.

Currently, the main efforts in the preparation of CNT-encapsulated materials are directed toward the modification of the chemical and physical properties of both the CNT and the guest species (confinement effect) in comparison with the pristine starting materials, with special interest in exploring their synergistic properties. Furthermore, structural and chemical modifications of the encapsulated material can be achieved through chemical (e.g., catalytic) or physical (e.g., electron beam) reactions. The present discussion is structured according to the chemical nature of the guest species. It includes descriptions of *inorganic materials*, namely metals, monoelemental nanostructures, alloys, salts and oxides, carbon allotropes (fullerenes and derivatives, graphene, diamonds, and carbon NWs), *organic and organometallic compounds*, which include biomolecules (drugs, DNA and RNA, proteins, nucleic acids), dyes, chromophores, polymers, and other types of organic materials, *gases* and *liquids*, including organic solvents, ionic liquids (ILs), and water. Table 5 provides a comparative overview of different types of guest materials encapsulated within CNTs, highlighting their typical filling approaches, stability, areas of application along with key considerations.

**Table 5:** Comparative Analysis of the Various Types of Guest Materials Confined within CNTs

Guest	Typical Filling Approach	Guest Material Stability	Application Area	Main Considerations
**Metals**	*In situ* (AD, CVD, EC, plasma).	Moderate to high	Catalysis, electronics.	Encapsulation enhances oxidation resistance and electrical properties.
	*Ex situ* (liquid-phase, EC).			
**Metal oxides**	*In situ* (EC), ex situ (MPH, liquid-phase, pyrolysis).	High	Energy storage, sensors.	Provide excellent thermal and chemical stability within CNTs.
**Salts**	*Ex situ* (MPH, sublimation, liquid-phase, sonochemical, UV-photolysis).	Moderate	Biomedical, EC.	Environmental factors influence stability; encapsulation aids protection/stability.
**Organic Molecules/polymers**	*Ex situ* (MPH. Sublimation, liquid-phase, EC).	Low to moderate	Drug delivery, photonics.	Encapsulation improves stability; compatibility depends on functionalization.
**Alloys**	*In situ*, *Ex situ* (EC).	Moderate to high	Magnetic, electronic devices.	Composition tuning allows for application-specific performance.
**Magnetic Materials**	*In situ*.	Moderate	MRI contrast, data storage.	CNTs protect against oxidation, enhancing durability in applications.
	*Ex situ* (MPH, Sublimation).			
**Fullerenes**	*Ex situ* (sublimation, liquid-phase).	High	Nanoelectronic, photovoltaic.	Encapsulation leads to unique electronic properties and stability.

### 7.1 Inorganic Materials

#### 7.1.1 Metals and Monoelemental Structures

Metal-filled CNTs are one of the most studied families of filled CNTs. Since the first report by Iijima in 1993,^162^ many researchers have successfully filled the inner cavities of CNTs with a wide range of metallic crystals. The number of metals successfully encapsulated in the interior of CNTs is quite extensive, with the initial efforts mainly devoted to the preparation of metal NWs with interest in their new electronic and magnetic properties.^75,199^ Filling the cavities of both SWCNTs and MWCNTs with monoelemental nanostructures has attracted attention in the past few years. A large variety of p-block elements, including metals, semimetals, and nonmetallic elements (XIII-XVI groups), have been successfully encapsulated inside CNTs. Here, we describe in detail the different elements that have been confined using both *in situ* and *ex situ* filling.

##### 7.1.1.1 *In Situ* Filling Approach

*In situ* filling can occur if the element to be encapsulated is present during the synthesis of CNTs. As mentioned before, AD and CVD are the two most important synthetic methods reported for the preparation of CNTs. In both cases, the formation of CNTs might require the presence of a metal-based catalyst that, in addition to acting as a catalyst for growth, can simultaneously enter the inner cavity of the growing nanomaterial, thus leading to the formation of metal-filled CNTs. Several metals such as Y,^160,168^ Mn,^200^ Bi,^201^ Ag,^202^ Ni,^169^ Cu^203^ and Sn^204^ have been encapsulated *in situ* by using the AD method. Subsequently, the synthesis of metallic carbides using the AD,^164,205–209^ CVD,^210–213^ or pyrolysis^214^ has been frequently reported.^29^ In two different reports,^199,215^ Louiseau et al. investigated the filling of CNTs by the AD method with a large variety of elements from group B (II–VI), reporting the successful encapsulation of Se, S, Sb, Ge, Cr, Ni, Dy, Yb, Gd, Fe and Co in their monoelemental forms. The metal employed during the synthesis determined the structure of the guest species, and the presence of metallic carbides was often observed.^152,168^ Because the growth of the CNTs and filling occurs simultaneously, when partial encapsulation results from the process, as in the case of Ni- or Co-promoted reactions, non-regular CNT diameters are obtained.^169,216^

Different variants of the CVD method have also been employed for the *in situ* synthesis of metal-filled CNTs.^217–224^ Gao et al.^225^ used the LaNi_2_ alloy as catalyst precursor to fill CNTs with Ni NWs through a CVD method at low temperature (550 °C). The grown NWs crystallized in a Face-Centered Cubic (fcc) structure, as indicated by TEM, SAED, and XRD.

Rao et al. synthesized metal-filled CNTs by pyrolyzing different metallocenes (MCp_*x*_s) in the gas phase at high *T*.^226^ Recently, additional precursors such as Raney-Ni catalyst,^227^ FeCp_2_,^108,228–231^ cobaltocene (CoCp_2_),^150,232^ acFeCp_2_, iron­(II) phthalocyanine (FePc),^233^ organogermanium precursors,^234^ Cu­(acac)_2_,^235^ and iron nitrate were used under different synthetic conditions (*T*, gas) to produce metal-filled CNTs with variable filling yields and structural characteristics.^155,236,237^ Figure 3 a) shows a long and continuous Co NW crystallized inside MWCNTs, prepared by a CVD approach using cobaltocene (CoCp_2_) as precursor.

**Figure 3:**
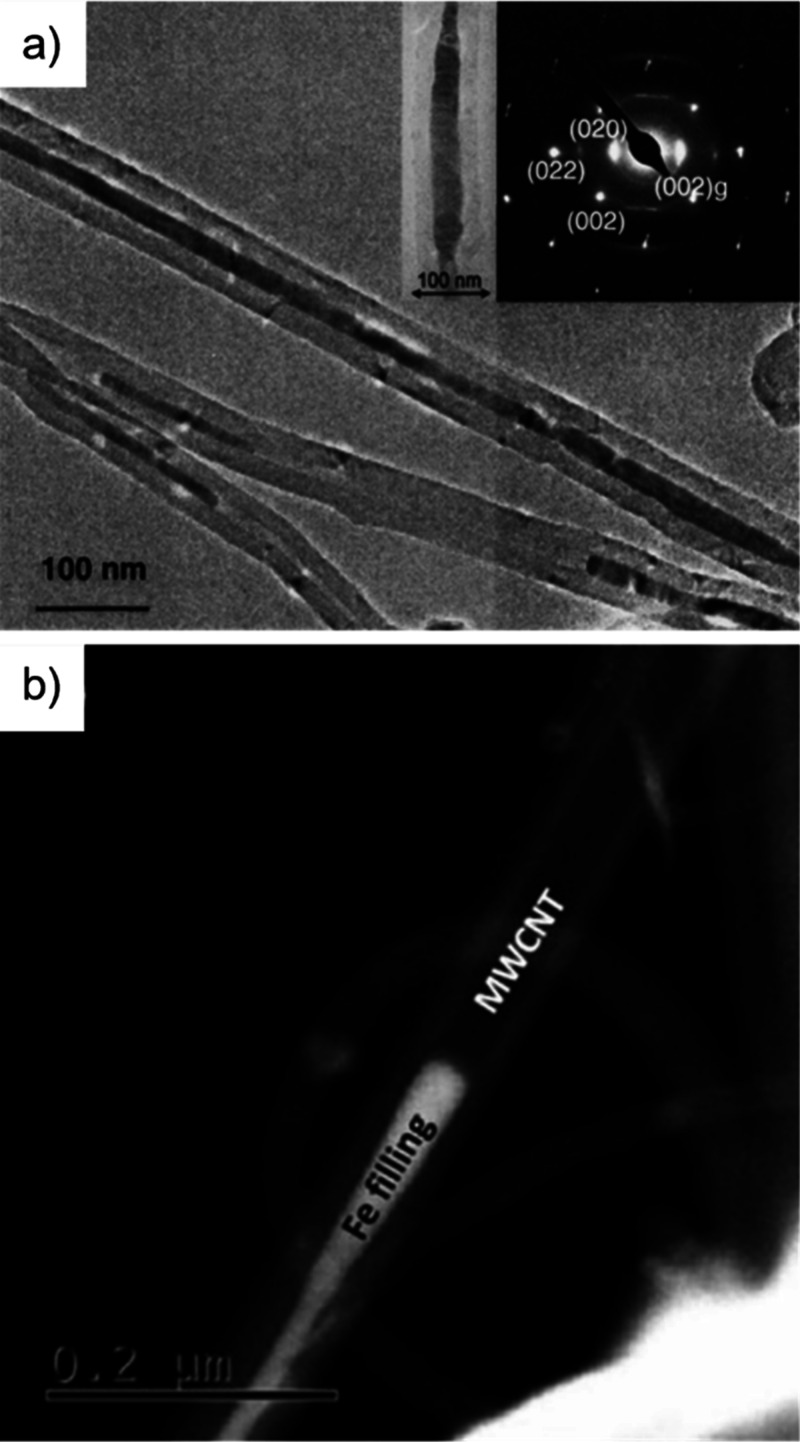
Metal-filled CNTs. a) Long and continuous Co NW crystallized inside MWCNTs by a CVD approach using CoCp_2_ as precursor.^150^ b) STEM-ADF image showing Fe-filling in a P and N codoped MWCNT, synthesized using an aerosol-CVD process.^224^ Panel a) is reproduced with permission from ref 150. Copyright 2017, Elsevier. Panel b) is reproduced with permission from ref 224. Copyright 2004, Elsevier.

Both the metal source and reaction parameters play a crucial role determining in the characteristics of the resulting metal–carbon heterostructures. For example, Boi et al. reported on the Cl-assisted CVD synthesis of ferromagnetic highly filled cm-scale buckypapers of horizontally aligned MWCNTs. In this study, Fe_3_C@MWCNTs were prepared using dichlorobenzene as the carbon source. Interestingly, they were able to improve the filling ratio and control the alignment of CNTs by tuning the evaporation *T* of the precursor and the vapor flow rate within the CVD system.^210^ According to the authors, the protocol takes advantage of the chlorine radicals generated from the process, which decreases the growth rate of the metal-based NWs within the interior of the CNTs, with the flow vapor rate playing an important role in the stabilization of the fcc and α-Fe γ-Fe phases at the nanoscale.^230^

Sengupta et al. explored Ni (Salen) as a catalyst source for the CVD synthesis of Ni-filled CNTs.^151^ In this case, metallic NPs were located at the tips of the CNTs, indicating a tip growth mechanism with a continuous and well-ordered filling. HRTEM performed on the filled samples indicated a high selectivity toward the filling of CNTs versus the external decoration. Liu et al. reported a simple catalytic method for synthesizing CNTs filled with long Co NWs.^238^ The compound Co­(CO_3_)­NO was used as a precursor and subjected to thermal decomposition to produce metallic cobalt particles encapsulated within the CNTs, with CO as the source of carbon. *In situ* filling of Fe by the reduction of Fe_2_O_3_ was obtained in the presence of H_2_, N_2_, and cyclohexane, the latter being employed as a carbon source upon catalytic decomposition.^239^ NWs, NRs, and NPs were produced using this method, and the presence of Fe was confirmed using HRTEM and EELS.

Grobert et al. tuned the properties of magnetic Fe@CNTs by changing the synthesis conditions.^240^ The magnetic characterization of Fe@CNTs revealed that their coercivity could be systematically tuned by varying the pyrolysis *T*, whereas the saturation magnetization could be significantly enhanced by optimizing the sublimation *T*. The same group also reported an *in situ* approach to encapsulate Fe into P and N codoped MWCNTs as it can be seen in the STEM-ADF of Figure 3 b).^224,241^ During the aerosol-CVD process, triphenyl phosphine, FeCp_2_ and benzylamine were used as precursors. The presence of N induced the formation of an intermediate alloy phase that subsequently decomposed to form the Fe filled CNTs containing the heteroatoms embedded within the carbon honeycomb lattice of the CNTs walls while the P was detected within the host cavity.^224^

A similar approach has also been explored for the encapsulation of Ni^242^ and Fe derivatives, including iron carbides,^243,244^ within CNTs.^245^ Notably, α-Fe and Fe_3_C can be prepared using either *in situ* ^240,246–249^ or *ex situ* methods.^250^ Importantly, the characteristics and magnetic behavior of nanomaterials are closely related to the synthesis approach.^251–258^ Recently, a molten salt electrolytic process was employed to synthesize Sn@CNTs.^158,259^ The process consists in the deposition of Sn and Li from a LiCl electrolyte containing small quantities of SnCl_2_ by electrolysis between two graphite electrodes and the subsequent release of graphitic layers from the graphite to form highly filled MWCNTs.

Various approaches that involve the preparation of metal precursors in the liquid phase, followed by pyrolysis of their mixture with a carbon source, have recently gained attention. In the presence of heteroatom sources, mostly N, these methods may also lead to the formation of M@doped-CNTs with heteroatoms.^260–262^ In some cases, these approaches involve using compounds such as metal–organic frameworks (MOFs), which can act as bimetallic sources and also contain p-block atoms (such as N), which are further incorporated within the conjugated walls of the CNTs..^263,264^ Using this technique and 1 nm Keggin-type polyoxometalate (POM) clusters, H_3_PW_12_O_40_ and H_3_PMo_12_O_40_, the anisotropic growth of both W_2_C and Mo_2_C within SWCNTs was achieved.^76^ Further annealing of W_2_C@SWCNTs in H_2_/Ar and the injection of O_2_ led to the formation of β-W@SWCNTs. Figure 4 presents a detailed electron microscopy analysis of the different steps required for the synthesis of β-W in the interior of SWCNTs.^265^ Other examples include the synthesis of CNTs filled with other metal carbides (MCs), such as Fe_3_C,^249^ and Fe_3_C.^266^

**Figure 4:**
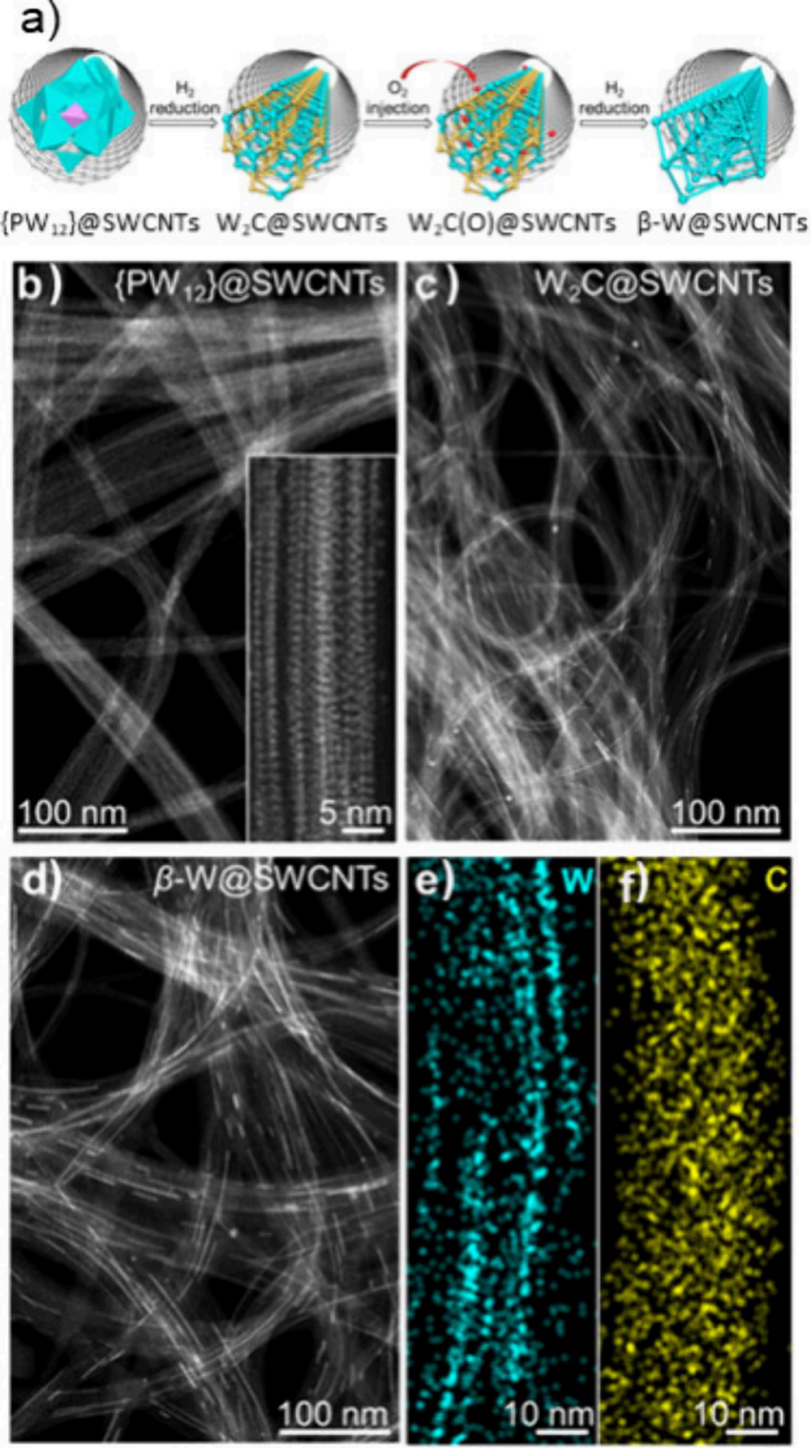
SWCNTs filled with β-W. a) Schematic representation of the synthesis of W_2_C@SWCNTs by encapsulation of the H_3_PW_12_O_40_ POM cluster. Subsequent reduction/oxidation cycles led to the formation of β-W@SWCNTs. b–d) HAADF-STEM images confirming the encapsulation of the cluster and W_2_C. Elemental mapping (EDX) showing the e) W and f) C distributions.^265^ Reproduced with permission from ref 265. Copyright 2025, American Chemical Society.

##### 7.1.1.2 *Ex Situ* Filling Approach

The *ex situ* filling approach is another commonly employed strategy for encapsulating monoelements into CNTs. In the case of metals, Pb and Eu can be directly confined by inducing their sublimation and subsequent crystallization within the NT cavities.^162,267^ The filling of CNTs with metals can also be achieved by the previous incorporation of a metal precursor into their hollow cavity and further reduction by chemical or thermal treatment.^268–274^ Chen et al. reported the synthesis of M@MWCNTs hybrids using AgNO_3_, Pd­(NO_3_)_2_ and AuCl_3_ as precursors. After encapsulation, the guest salts were heated in a stream of H_2_, leading to the reduction to form metallic Ag, Pd and Au, respectively.^275^ Other methods for the reduction of Ag and Au precursors to their metallic form inside the CNTs have also been reported. These include using different reducing agents,^276^ annealing under mild conditions^277^ or irradiation with ^60^Co γ-rays.^278^ Figure 5 a) shows a HRTEM image of a CNT filled with small spherical Ag crystals prepared by a two-step wet method. The authors opened the CNTs with HNO_3_ and subsequently stirred them overnight in the presence of AgNO_3_. To obtain Ag@CNTs, the separated and clean filled CNTs were calcined at 250 °C.

**Figure 5:**
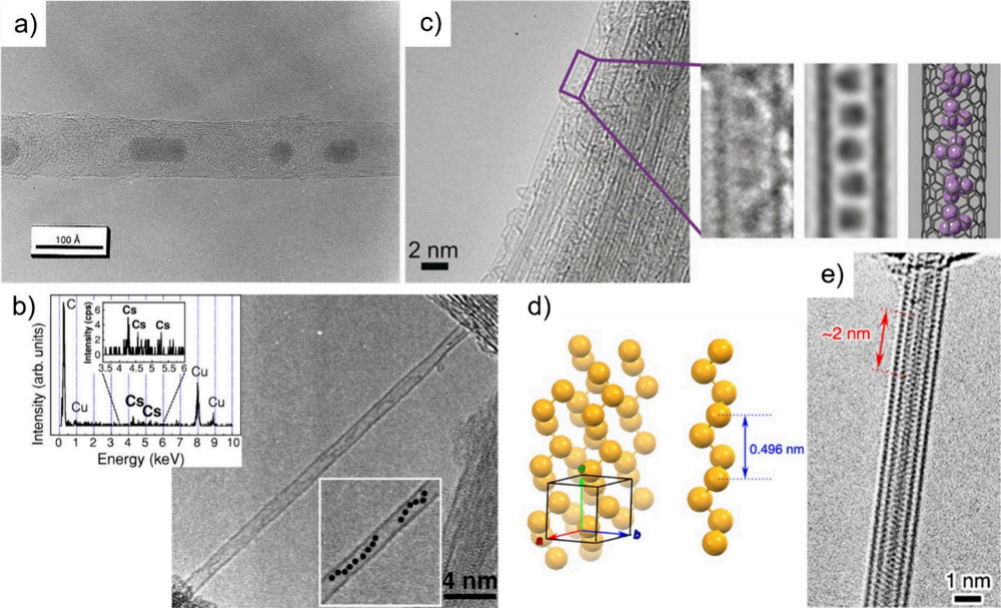
CNTs filled with monoelemental systems using *ex situ* approaches. a) HRTEM image of a CNT filled with small spherical Ag crystals.^277^ b) Cs-filled CNTs synthesized using a plasma ion irradiation technique.^91^ c) HRTEM image of As_4_ molecules contained inside a SWCNT. The magnification of the highlighted area, its corresponding HRTEM simulation and the structural model are shown on the right.^77^ d) Structural model of the double-helical nanostructure formed after encapsulation of Se inside CNTs through a vapor-phase approach, corresponding to the HRTEM shown in e).^81^ Panel a) is reproduced with permission from ref 277. Copyright 1996, American Chemical Society. Panel b) is reproduced with permission from ref 91. Copyright 2003, American Physical Society. Panel c) is reproduced with permission from ref 77. Copyright 2018, John Wiley and Sons. Panels d) and e) are reproduced with permission from ref 81. Copyright 2013, American Chemical Society.

This approach was also explored for filling SWCNTs and DWCNTs with metals. Sloan et al. demonstrated for the first time the possibility of filling AD SWCNTs using wet chemistry techniques.^147^ The SWCNTs were solution-filled with RuCl_3_ and then reduced to Ru metal. Because this method involves encapsulation and subsequent reactions inside CNTs, it can be useful not only for filling metal crystallites^279^ but also for transforming of the precursors into other species, such as metal oxides^275,280^ and metal sulfides.^281^ Another remarkable approach includes the formation of metallic NWs by initially filling metallofullerenes within SWCNTs and their subsequent coalescence, leading to the formation of internal SWCNTs with a metal core, resulting in M@DWCNTs.^282–284^

Electrodeposition has also demonstrated to be useful for the encapsulation of metals within CNTs. Ni and Co were encapsulated into vertically aligned CNTs previously synthesized by the CVD method,^285–288^ while Fe,^289^ Li, Na, and K^187^ were confined into CNTs forming electrode sheets from a microcurrent system. These approaches induce selective loading of materials within the cavities of CNTs. Using an alternative methodology, the bombardment of SWCNTs with accelerated Cs ions (plasma ion irradiation) led to the confinement of Cs within the CNTs (Figure 5 b)).^91^ Template-assisted synthesis using alumina has been widely reported.^247^ This involves the preparation of CNTs in the selected template and subsequent filling using different means. Aligned CNTs filled with Co^286,290^ or Ni^291^ have also been obtained using similar template-based approaches.

A multistep synthesis has mainly been employed for the encapsulation of group XIV elements into CNTs.^292^ It was reported that the gas phase and thermal decomposition of Sn, Ge, and Pb precursors respectively, followed by thermal annealing under a reducing atmosphere (Ar/H_2_) promoted the formation of both, pure metallic NWs (up to 1 μm length) and NPs inside CNTs. Notably, when the solution-phase approach was followed, the reaction parameters played an important role in the filling yield and morphology of the guest material. Shorter treatment times resulted in the formation of small NPs (ca. 50 nm), whereas the growth of NWs was favored when the reaction time was increased.

Other monoelemental nanostructures have also been encapsulated using this approach. Hart et al. polymerized As, P and Sb inside SWCNTs.^77,78,293^ Arsenic adopted different configurations, and the authors reported, for the first time, the presence of highly reactive chains of As_4_ (Figure 5 c)) single-stranded zigzag chains, or double-stranded zigzag ladders confined within CNTs. In addition, the presence of As at the tips was found to act as a corking agent, allowing the isolation of new sensitive structures that would otherwise react rapidly in the presence of O_2_.^77^ I_2_,^112,174,175,280,294–297^ and chalcogen element-based structures, namely, containing S90,^298–300^ and Se,^299,301^ were filled using the vaporization method. In the case of S, the material adopted the structure of chains inside the CNTs, while double helices of Se^81^ were observed after encapsulation (Figure 5 d–e)) taking advantage of its relatively low melting point (449.5 °C). Te, on the other hand, has been incorporated within DWCNTs and SWCNTs’ cavities by means of MPH^302^ and sublimation^20^ syntheses, respectively. In 2020, Qin et al. used a physical vapor transport (PVT) approach to synthesize Te NWs within SWCNTs, with the formation of a single chain or few chain-based nanostructures being limited by the inner diameter of the CNTs,^22^ thus confirming the usefulness of the rational selection for the tailored synthesis of new nanostructures at the nanoscale. Theoretical studies have predicted the effects of encapsulation on the electronic and transport properties of Te chains. While compressive strain induces transitions between direct and indirect band gaps in Te single atomic chains, encapsulation of Te within CNTs induces direct band gaps, with the band alignment being closely linked to the diameter of the CNT.^303^

Research on the synthesis of P in its three most common allotropic forms, namely, white, black, and red P, has been widely reported. Intense work in the field has led to the synthesis of structures with lower dimensionality, which differ from those of 3D crystalline architectures. This has resulted in a library of P-based structures, including polymeric tubes, P-based cages, and 2D forms of black P. The use of CNTs as growth-directing agents has been the most widely used strategy for the formation of 1D structures. The advantage lies in the demonstrated capacity of the host CNTs to protect the material from oxidation. It also diminishes the damage induced during the characterization of the resulting hybrid, as in the case of exposure to an electron beam when employing high-resolution microscopic techniques. CNTs may also be useful as nanoreactors, allowing the P polymerization, thus leading to phase transformations, for instance, from white to red P. By exploring a variety of synthetic techniques, which range from solution and MPHs to vapor transport, it has been possible to encapsulate phosphorus chains (C–P),^304^ square columnar (SC-P),^83^ and ring-shaped P (r-P) within SWCNTs.^15^ In 2017, Hart et al. reported the formation of single and stable P_4_ molecules located within the cavities of 0.81 nm hosts (average diameter) after encapsulation of white P (Figure 6).^15^

**Figure 6:**
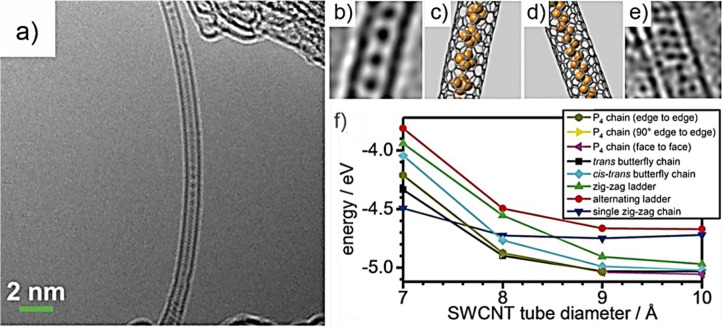
Encapsulation of P within CNTs. a) TEM image of a SWCNTs filled with a P-based nanostructure. b–c) HRTEM image and simulated structure of P_4_ units. d–e) Their polymerized forms confined within SWCNTs. f) Energies of the predicted chains as a function of the SWCNT diameter.^15^ Reproduced with permission from ref 15. Copyright 2017, John Wiley and Sons.

Zigzag chains resulting from the polymerization of elemental P were also observed. The authors used DFT calculations to predict the configurations that could be grown inside SWCNTs. Within a 0.7–1.0 nm range, theoretical studies have concluded that the P_4_ form represents the most stable configuration in CNTs with larger diameters, while decreasing the CNTs radius might favor the growth of zigzag polymeric chains (Figure 6 f)).

Subsequent experiments confirmed the presence of 1D C–P within the DWCNTs.^66^ Similar to the results observed for SWCNTs, the structure adopted by P was closely linked to the inner diameter of the host, including linear monoatomic chains (*d* = 0.8–1 nm), double (P_8_P_2_)_*n*_ chains (*d* = 1.3–1.74 nm), and triple (P_8_P_2_)_*n*_ chains (1.6–2.9 nm).^305^ The encapsulation of P within CNTs with narrow diameters is not limited to the formation of ring or chain structures. In 2020, the formation of a SC-P structure confined within 1 nm SWCNTs was reported (Figure 7 a–b)).^83^

**Figure 7:**
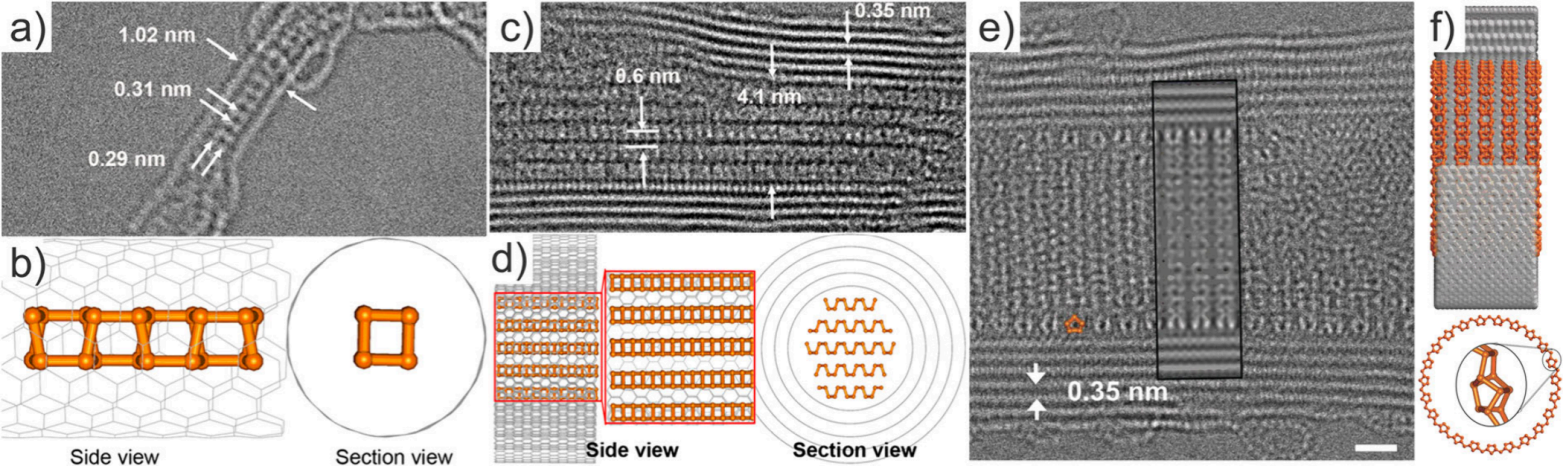
Encapsulation of P within CNTs. The structure adopted by the filler closely depends on the host diameter. Negative spherical aberration image and structural models of a–b) a SC-P@SWCNT c–d) a ZR-P@MWCNT^83^ and e–f) a r-P@MWCNT.^306^ Panels a–d) are reproduced with permission from ref 83. Copyright 2020, American Chemical Society. Panels e–f) are reproduced with permission from ref 306. Copyright 2017, John Wiley and Sons.

More recently, Vorfolomeeva et al. optimized the encapsulation protocol by modifying parameters such as the form of P (white and red), the amount of the guest precursor, and the conditions of synthesis.^307^ MWCNTs with an inner diameter of 4.1 nm have also been used as hosts for the encapsulation of P, which for instance adopts the shape of zigzag nanoribbons (ZR-P); being the most stable structure within CNTs with diameters larger than 1.4 nm (Figure 7 c–d)).^83^

Vaporization led to the formation of r-P-filled MWCNTs^306^ and SWCNTs^308^ through the incorporation of red P, which sublimed phase diffused into the cavities of the CNTs, reaching filling yields of up to 60%. Elemental P adopted a more complex structure, as determined by the negative spherical aberration imaging (NCSI) technique, compared with P encapsulated in the single-walled counterparts, which consisted in alternating P_8_ – P_2_ subunits (helical coils). Theoretical calculations supported the formation of P rings composed of 230 atoms (for a 5.90 nm CNT diameter), analogous to the experimental findings in MWCNTs with diameters ranging between 5 and 8 nm (Figure 7 e–f)). The hollow cavities of the CNTs allow the formation of multiple layers of rings with variable diameters and, therefore, a variable number of P atoms, separated from each other by a 6.4 Å distance.^306^ Using a solution approach, Li et al. encapsulated polyphosphides (PP), obtained by dissolving red P in dimethyl sulfoxide (DMSO) within MWCNTs.^309^

In addition to high-resolution microscopy imaging, alternative characterization tools were used to confirm the presence of various P-based nanostructures encapsulated within CNTs. Figure 8 shows the experimental and theoretical Raman spectra computed using the Placzek approximation for white P filled within 1.6–2.9 nm SWCNTs. Although there is no perfect agreement between the experimentally recorded data and the predicted spectra, the results suggest a combination of the nanostructures that might be grown inside the CNT mold with the characteristic vibration modes for P–P appearing within the 300–400 cm^–1^ range. The signals can be associated with vibrations in different nanostructures, namely, orthogonal stretching in the chain direction, pentagonal cages, or to the shared P–P bond in P2. Other visible peaks can be associated with wag-like modes along the backbone (ca. 190–200 cm^–1^), oscillations of the P8 cage (220–294 cm^–1^), or P–P stretching of the cross-linking bonds between the chains (251 cm^–1^).

**Figure 8:**
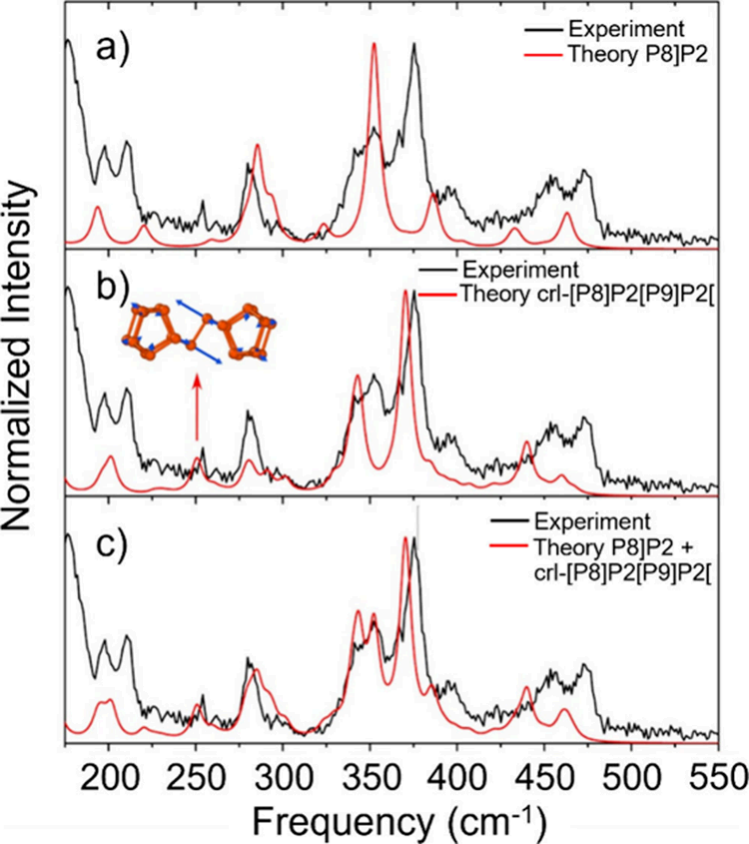
Experimental and theoretical Raman spectra of different phosphorus structures computed using the Placzek approximation. The characteristic P–P signals appearing within the 300–400 cm^–1^ range correspond to orthogonal stretching in the chain direction, to pentagonal cages, or to the shared P–P bond in P2 vibration of the inorganic nanostructures encapsulated within CNTs.^305^ Reproduced with permission from ref 305. Copyright 2022, American Chemical Society.

Research on the guest–host interactions is crucial for understanding the filling mechanisms, which contributes to the development of new synthetic strategies. Special interest has been paid to the incorporation of Li within CNTs as novel energy storage systems because the resulting hybrid has been advocated as a promising candidate for improving the performance against the current benchmark. Therefore, Wei et al. described the mechanisms of encapsulation of Li by means of *in situ* TEM. They observed that the carbon walls play an active role in the filling process, providing geometric/mechanical constraints and electron/ion transport channels, which alter the Li growth patterns, leading to the formation of unusual Li structures, such as polycrystalline NWs and free-standing 2D ultrathin (1–2 nm) Li membranes. First-principles-based calculations revealed that N/O doping might contribute to filling and stabilizing Li inside the carbon nanostructure owing to energy minimization associated with the formation of low-energy Li–C interfaces.^89^ Table 6 summarizes the main methods reported in the literature for synthesizing CNTs filled with metals, monoelemental nanostructures, and alloys.

**Table 6:** Main Approaches Reported for Filling CNTs with Metals, Monoelemental Nanostructures, and Alloys

Method	Method	Species	Reference
***in situ***	AD	Ti, Cr, Fe, Co, Ni, Cu, Zu, Mo, Pd, Sn, Ta, W, Gd, Dy, Yb, Y, Mn, Bi, Ag, Se, S, Sb, Ge, Sm	^169^,^199–204^,^215^,^310^
		LaC_2_, GdC_2_, Mn_*x*_C_*y*_, TaC and CeC_2_, Y_*x*_C_*y*_, TiC	^160^,^164^,^168^,^205–209^,^310^
	CVD-Pyrolysis	Co, In, Cu, Ni, Fe, Ge, B	^108^,^150^,^151^,^210^,^217^,^218^,^220–223^,^225–230^,^232^,^233^,^235^,^236^,^238–240^,^245^,^247^,^248^,^250^,^251^,^255–257^,^286^,^290^,^291^,^311^,^312^
		Fe_3_C, TiC	^210–213^,^243^,^244^,^246^,^251^,^252^,^255^,^256^,^258^
		FeCo, NiFe, NiFeCo, NiZr, NiCo	^246^,^311–321^
	Molten salt electrochemistry	Sn	^158^,^259^
	Liquid phase-Pyrolysis	Ni, Co, Fe, β-W	^260–265^
		FeNi, FeCo, Fe_3_C, W_2_C, Mo_2_C, Fe_2_C, CoFe/NiFe, RuCo_2_Ni, CoNi, FeCoNiMnCr	^76^,^88^,^179^,^180^,^249^,^266^,^322–327^
***ex situ***	MPH	Pb, Sn, Se, S, Sb, Te	^78^,^162^,^292^,^299^,^302^,^328–331^
		SnSb	^332^
	Liquid phase-reduction	Ru, Co, Fe, Te, Ag, Pd, Ge, Pb, Au, Mo, Zn, black P, white P, Cu, Pt	^15^,^147^,^268–271^,^274^,^275^,^277^,^279^,^280^,^292^,^309^,^333–335^
		NiFe	^335^,^336^
	Pyrolysis-decomposition	Pt, Fe/Fe_3_C	^178^,^321^,^337^
	Vapor-phase	Se, Te, Fe, Co, As, S, P, I_2_	^20^,^66^,^77^,^78^,^81^,^83^,^90^,^112^,^174–176^,^272^,^280^,^294–298^,^300^,^301^,^304^,^306^,^338^
		Mo_2_C	^272^
	UV-photolysis driven	I_2_, MoCl_2_	^184^
	Electrodeposition	Ni, Co, Li, Na, K, Fe	^187^,^285–289^
		Ni_*x*_Fe_*y*_, CoNi	^185^,^186^,^339^
	Plasma-ion irradiation	Cs	^91^

#### 7.1.2 Alloys

The insertion of alloys within the inner cavities of CNTs has also attracted interest.^312,319,332^ The confinement of these structures within CNTs opens a new window for exploiting their physical and chemical properties at the nanoscale.^326,340^ CVD is the most common approach for synthesizing CNT-alloy hybrids. Special attention was directed toward the formation of Fe–Ni@CNTs and the resulting chemical composition and morphology.^180,313,341^ Lv et al. dissolved a mixture of FeCp_2_ and nickelocene (NiCp_2_) in different carbon sources (C_6_H_6–*x*_Cl_*x*_ (*x* = 0–3)), which act as precursors for the formation of CNTs. After annealing the solution at a high *T* under a reducing atmosphere, long and continuous NWs were observed encapsulated along the inner cavities of the CNTs,^314^ with the filling yield depending on the nature of the carbon precursor. XRD was employed to identify the structure of the guest material, confirming the presence of fcc-structured FeNi alloys. Other alloys, including FeCo, FeNi, CoNi, FeCoNi, and Ni/ternary Zr, have also been filled within CNTs using CVD.^246,315–317,342^ Fe–Co ferromagnetic NWs with enhanced coercivity were synthesized within the cavities of aligned MWCNTs by the decomposition of a FeCp_2_/CoCp_2_ mixture using thermal CVD.^318^ Additional examples of the filling of CNTs with different alloys are listed in Table 6.

EC deposition and plasma-hydrogen-induced demixing have also been explored for the controlled synthesis of permalloy-filled CNTs^185^ and vertically aligned CNTs filled with Pd–Co.^343^ The pyrolysis of nitrate-based precursors and NiCo-mesoporous silica NPs as catalysts and metal sources^311^ allowed the preparation of NiCo-filled MWCNTs.^326^ An environmentally friendly approach was used for the encapsulation of the high-entropy alloy FeCoNiMnCr with application in non-enzymatic glucose sensing.^327^ Another method involves the encapsulation of NiCo alloy NPs within CNTs by pyrolysis of a Co/Ni-coordinated macrocyclic ligand (Cyclam) with ZIF-67. Additionally, the authors were able to include N atoms within the honeycomb lattice of the host (CNT walls) by adding melamine.^88^

#### 7.1.3 Salts

As mentioned before, Tsang et al. reported in 1994 the filling of an inorganic salt into the cavities of CNTs.^156^ Subsequently, different approaches have been implemented for filling CNTs with a wide variety of metal salts. Sloan et al. reported the filling of SWCNTs with RuCl_3_ ^147^ and studied the structure of several compounds,^344,345^ paving the way for the preparation of a wide library of materials in this category.^346,347^ Reports on filling CNTs with MX, where X = F, Cl, Br, I, not only include the encapsulation of transition metal compounds (M = Ag, Fe, Mn, Zr, Co, Cu, Ni or Zn among others),^171,280,295,297,348–360^ but also the formation of inner structures of alkali,^361–364^ alkaline earth^365^ and p-block halide crystals,^19,45,330,366–371^ as well as TMCs,^18,68,133,330,372–378^ halide-chalcogenides,^183^ or mixtures of halides and halide-chalcogenides.^166,171,362,379,380^ Molten-phase capillary wetting is the most commonly used approach, but solution techniques,^381^ CVD,^380,382^ sonochemical growth,^182,183,383^ electron-beam irradiation, pyrolysis,^378,384^ chemical vapor transport (CVT),^360^ and laser-assisted synthesis^19^ have also been employed for this purpose. Some studies have shown that both the filling yield and crystallinity of the salt depend on the synthesis conditions. Examples of the endohedral functionalization of CNTs with MX are shown in Figure 9.

**Figure 9:**
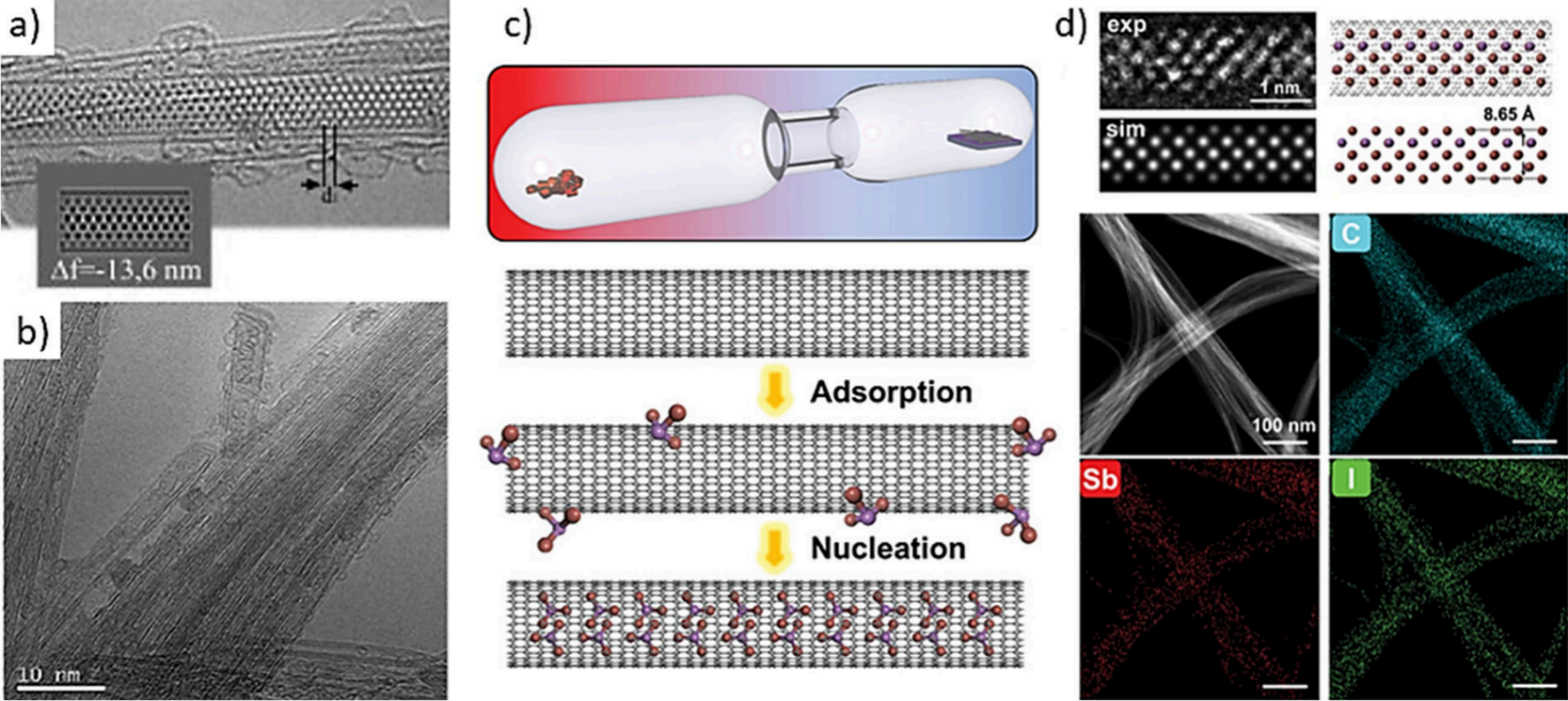
Different MX encapsulated inside CNTs. HRTEM images of a) 3DCuI,^353^ and b) GdCl_3_ crystals.^385^ c) Schematic representation and proposed mechanism for the encapsulation of SbI_3_ within SWCNTs by sublimation. The process involves adsorption of SbI_3_ molecules onto the external surface of the SWCNTs, followed by nucleation and crystallization upon cooling, leading to the formation of 1D SbI_3_ chains. d) HAADF-STEM experimental and simulated images and structural models of SbI_3_@SWCNTs. EDX elemental-mapping confirmed the atomic distribution of C, Sb, and I.^371^ Panel a) is reproduced with permission from ref 353. Copyright 2012, Elsevier. Panel b) is reproduced with permission from ref 385. Copyright 2021, AIP Publishing. Panels c–d) are reproduced with permission from ref 371. Copyright 2024, John Wiley and Sons.

In the case of materials that are highly unstable under thermal treatment or even under environmental conditions, for instance, due to their propensity for decomposition, oxidation or dehydration, a two-step protocol was proposed. To prepare FeF_3_@DWCNTs, Doubtsof et al. developed a new synthetic approach, which involved the preliminary filling of the CNTs with the iodized iron precursor, FeI_3_, using the MPH approach, followed by fluorination using gaseous F_2_. By controlling the reaction conditions, the authors avoided undesired side reactions, such as the external fluorination of the CNTs. This approach allowed the endohedral and controlled formation of hydration-free FeF_3_. Moreover, encapsulation within the CNTs may provide extra protection against decomposition or transformations, which often hinder the application of the material in fields such as batteries or catalysis.^359^

1D crystals of lanthanide halides confined within CNTs have been synthesized, taking advantage of the low surface tension and melting points (<1000 °C) of the materials.^23,57,385–393^ When LaI_3_ is confined in SWCNTs, the encapsulated material adopts the bulk structure of the precursor, with the atomic distribution undergoing important variations.^115^ The different crystalline structures observed correspond to LaI_3_, with one-third of the iodine positions unoccupied, and the presence of individual I.^391,394^ Xu et al. annealed mixtures of arc-synthesized SWCNTs and selected halides under non-oxidant conditions to introduce polyhedral chains of La, Nd, Sm, Eu, Gd, Tb, and Yb chlorides into their inner cavities. Diverse crystalline structures were observed in the samples. The crystallization process was highly influenced by the dimensions of the host. Additionally, the success of the encapsulation process depends on the characteristics of the filling agent. GdCl_3_ has received special attention in this regard. It has been shown that DWCNTs filled with GdCl_3_ (Figure 9 b)) undergo a superparamagnetic transition that can be correlated with phonon hardening at low *T*.^390^ On the other hand, the encapsulation of GdI_3_ into CNTs was found to lead to the reduction of the lanthanide metal and induce disorder in the initial GdI_3_-type structure. The magnetic response of the material was not compromised, retaining a strong paramagnetic response and an effective magnetic moment of ∼6 μB.^395^ Although TEM is widely employed for the characterization of both the crystal structure and filling yield in samples of filled CNTs, TGA provides a bulk quantitative assessment and has been used to determine the filling yield of a variety of MX.^388^ The key parameters for conducting TGA on carbon nanomaterials have recently been examined to ensure reliable and reproducible measurements of TGA data.^396^

The guest halides can adopt different configurations, ranging from NWs, which is the most commonly reported structure, to NPs, nanoclusters, NTs, or nanosnakes.^23^ The newly formed nanostructures depend on factors such as the bulk structure of the salt, size of the host, *T* of treatment, and method of filling.^19,23,397^ In some cases, encapsulation within the hollow cavities of CNTs may induce significant variations in the structural arrangement of the guest. When vdW solids are encapsulated in CNTs, the formation of a single layered nanotube (SLNT) of the MX has been observed.^36^ The resulting structure was a “1D tubular vdW heterostructure”.^37^ Several compounds with this type of structure, including PbI_2_,^368^ ZnI_2_,^19,23^ BiI_3_,^367^ BiCl_3_,^398^ CeCl_3_,^23^ CeI_3_,^23^ TbCl_3_,^23^ GdCl_3_,^57^ SmCl_3_,^57^ CrI_3,_ ^399^ and LuX_3_ (X= Cl, Br, I)^400^ have been encapsulated into CNTs. Among them, PbI_2_@MWCNTs were the first identified SLNTs grown within the CNT’s cavities in 2013 (Figure 10).^368^

**Figure 10:**
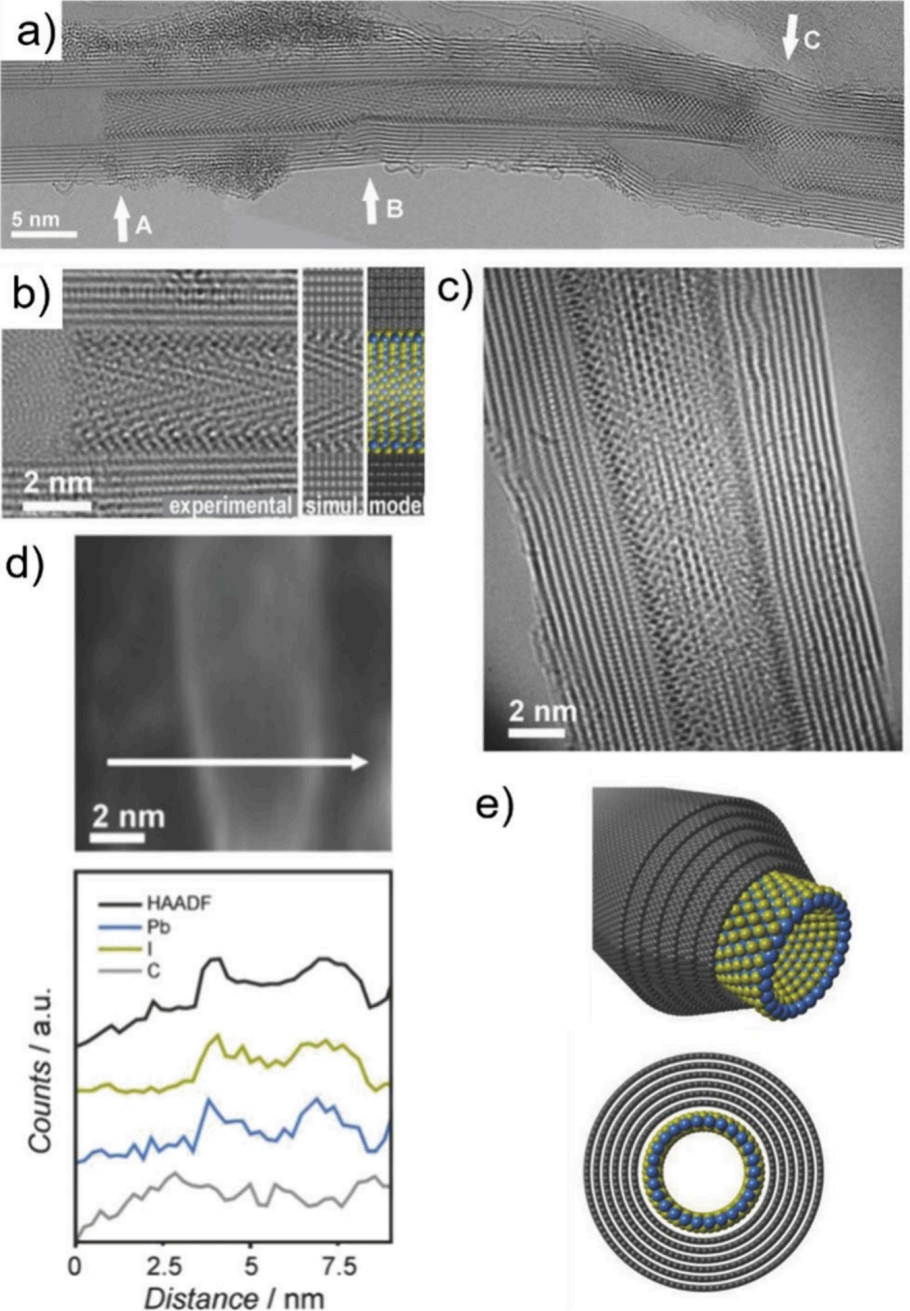
PbI_2_ NTs encapsulated within MWCNTs. The heterostructure was prepared by the MPH approach. a) Aberration-corrected HRTEM image of a single-layered PbI_2_ NT. b) Details of the HRTEM image (area A) with its corresponding simulation and model (cross section along the main axis). c) Aberration-corrected HRTEM image of another single-layered PbI_2_ NT. d) STEM-EDS line profiles confirming the presence of a PbI_2_ NT. e) Schematic representation of the grown single-layered PbI_2_ tubular materials (cyan and green spheres representing Pb and I atoms, respectively) within the inner cavities of a MWCNT (gray spheres). A cross-section across the main axis of the 1D tubular vdW heterostructure is also included for clarity.^368^ Reproduced with permission from ref 368. Copyright 2013, John Wiley and Sons.

Remarkably, an enhanced formation of such heterostructures has been reported using laser-assisted filling (∼94% of filled CNTs contained PbI_2_ NTs; 80 mJ·cm^–2^ fluence and 1000 pulses)^19^ compared to conventional thermal annealing (21.7% of filled CNTs contained PbI_2_ NTs). 1D tubular vdW heterostructures in which SWCNTs serve as the inner core for the growth of inorganic NTs on their exterior, have also received considerable attention.^36^ In this case, BN has been largely investigated,^39,401^ but other materials are also being studied.^402–404^

The characteristics of eutectic melting compounds have been exploited to fill CNTs with species that cannot be encapsulated because of their high melting points. Mixtures such as (KCl)_*x*_(UCl_4_)^405^ and AgCl–AgBr^171^ led to the formation of continuous NWs, the nature of which was related to the initial composition of the mixture. Using this approach, metastable 1D AgCl_1–*x*_I_*x*_ structures were grown inside the SWCNTs.^406^ The crystalline structure, which coexisted with metallic Ag-filled inner areas, consisted of a 1D “tunnel” containing Cl and I atoms distributed along the structure derived from wurzite AgI. This is probably the first report on a ternary phase encapsulated in CNTs. Hsu et al. annealed decapped CNTs in the presence of K and further treated the sample under solvothermal conditions, using CCl_4_ as solvent. Microscopic characterization showed the presence of KCl crystals embedded within the walls of the MWCNTs, thus increasing the original *d*-spacing between the walls, as well as a series of structural disruptions in the C layers. Nevertheless, KCl was not detected within the hollow cavities of the CNTs.^407^

Filling CNTs with Cu halides (CuX) has been both theoretically explored and synthesized via *ex situ* approaches.^131,408^ High loading efficiencies were achieved when the capillary technique was used to encapsulate CuI. Spectroscopic analyses of CuX@CNTs (X = I, Br) revealed interactions between the fillers and CNTs that affected the electronic structure of the salts.^297,409–411^ Gas-phase synthesis has been employed to fill CNTs with CuX, such as CuCl@SWCNTs, which are of interest for their optical properties.^354,412^ Fedotov et al. annealed metallic and semiconductive SWCNTs in the presence of gas-phase CuCl under different experimental conditions. Metallic CNTs were found to be more susceptible to filling using this method.^354^ CuCl functionalization induces the formation of n-doped hybrids, which consequently results in the suppression of optical absorption transitions that are characteristic of the CNTs. Mn,^413^ Cd,^414^ Zn,^415^ or Sn halides^416^ filled into the cavities of CNTs also induce charge transfer and hole doping from the CNTs to the guest, thus increasing their semiconducting character or suppressing their metallic properties.

Sub-nm halide perovskites were successfully encapsulated within SWCNTs using a MPH approach.^417^ The confinement of CsPbBr_3_ and CsSnI_3_ resulted in the formation of three ABX_3_ perovskite archetypes and a perovskite-like lamellar NW, which were sterically stabilized within the SWCNTs. Confinement was predicted to induce various conformations, vacancies, and electronic states that may play a role in the charge transport and optoelectronic properties of this family of materials, which have attracted interest owing to their applications in photovoltaics and optoelectronics. Figure 11 shows microscopic analysis of halide perovskites encapsulated within SWCNTs. HAADF imaging a) along with EELS elemental map **(b–c)**) confirm the presence of a lamellar cesium tin iodide formed within a ∼1.6 nm diameter SWCNT. Two theoretical models were developed, corresponding to the [Cs_4_Sn_4_I_14_]_2_ and the Cs_4_Sn_4_I_12_ (with vacancies created by removing I atoms). Other structures such as the CsSn^II^I_3_ -perovskite-like polymeric system **e)** were observed by HRTEM.

**Figure 11:**
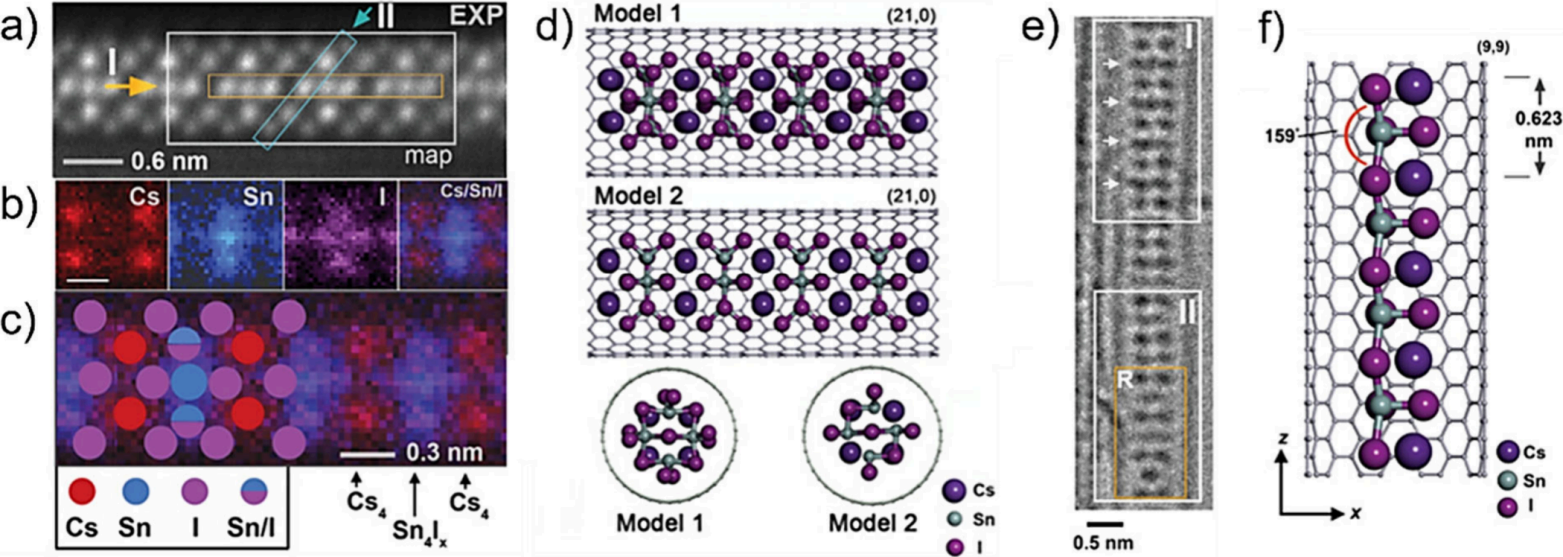
Halide perovskite structures encapsulated within SWCNTs. a) HAADF image along with the corresponding b) EELS elemental map of a lamellar cesium tin iodide formed within a ∼1.6 nm diameter SWCNT. c) An overlaid motif was added to indicate the positions of Cs, Sn, and I. d) Two optimized models are presented for the obtained perovskite. Model 1 corresponds to the [Cs_4_Sn_4_I_14_]_2_ structure, whereas Model 2 includes the presence of vacancies created by removing I to give Cs_4_Sn_4_I_12_. e) HRTEM image of a CsSn^II^I_3_-perovskite-like polymeric structure derived from Pm3m CsSnI_3_ encapsulated within a SWCNT. The material adopts a SnI_2_-based bilayered structure where SnI_2_ (arrowed) alternates with Cs/I single-atom columns (region I). A rotated region R was observed in II. f) Enlarged view of the DFT-optimized CsSnI_3_ bilayer structure with the calculated repeat and longitudinal I–Sn–I angle indicated.^417^ Reproduced with permission from ref 417. Copyright 2014, Springer Nature.

Faulques et al. reported the formation of novel Cs_2_Mo_6_Br_14_@SWCNTs using a melting-phase approach. The authors studied the encapsulation of inorganic clusters within SWCNTs of different diameters, evaluating the packaging configuration adopted by the guest molecules, which formed either ordered cluster arrays or polymerized into continuous [Mo_2_Br_6_]_*x*_ chains inside the narrower tubular hosts.^418^ *In situ* approaches have been attempted to encapsulate TMCs,^156,157,384^ particularly metal sulfides, due to their potential applications in batteries. In 2011 Su et al. reported a one-step CVD method that used dimethyl sulfide as both the carbon and sulfur sources. The authors used a stainless-steel substrate as both the support and promoter (catalyst) of the pyrolytic reaction, which led to the formation of Fe/Ni sulfide-filled CNTs.^419^ Ni­(NO_3_)_2_ and Ni NPs were recently used as Ni precursors in a CVD system in which H_2_ was bubbled in a solution of thiophene, thus leading to the simultaneous graphitization of carbon and filling of the emerging tubular nanostructures with crystalline Ni_3_S_2_.^420^ AD^421^ and microwave-plasma-enhanced CVD^422^ allowed the growth of crystalline GaN NWs inside CNTs in the presence of N_2_ and methane, the latter of which served as the carbon source.

Various sulfides have been encapsulated using an *ex situ* approach. CdS crystals and AuS can be achieved by annealing CdO and AuCl_3_ filled CNTs, previously prepared by a wet chemical approach, under flowing H_2_S.^275^ Recently, FeS NPs have been grown into CNTs by employing Fe and S as filling precursors.^259^ Other approaches involve one-step synthesis using FeCp_2_ in the presence of NaN_3_ under supercritical CS_2_ ^423^ or the CVD method to encapsulate long Co_9_S_8_ NWs using a dimethyl sulfide precursor.^424^ Costa et al. investigated the stability of sulfide materials encapsulated in and expelled from MWCNT capsules.^24^ A high density of electrical current was employed to induce the abrupt release of Zn_0.92_Ga_0.08_S from the interior of CNTs (Figure 12). This event did not alter the chemical identities of either the expelled or the encapsulated materials. After 30 days of air exposure, significant differences in reactivity were observed. The expelled chalcogenide particles were found to be chemically more unstable than the material protected by the carbon shell. The same group also investigated the oxidation process of the encapsulated sulfide (Zn_0.92_Ga_0.08_S).^425^

**Figure 12:**
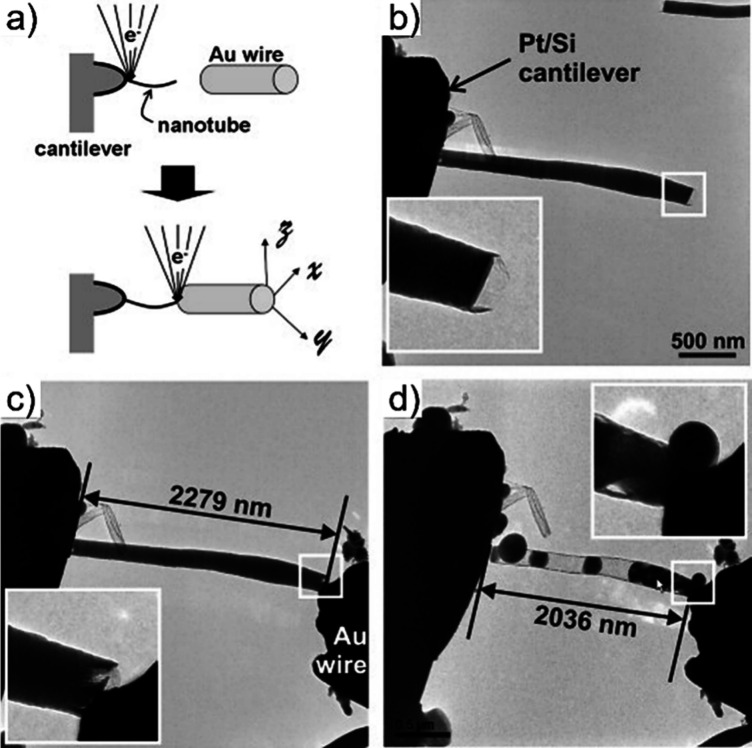
Stability studies of sulfide materials encapsulated in and expelled from MWCNT capsules. a) Diagram of the procedure followed to weld the CNT to both electrodes (wire and cantilever) with a converged electron beam (e^–^), thereby obtaining a doubly clamped configuration. b) A welded Zn_0.92_Ga_0.08_S@CNT was suspended from an electrically conductive force-sensing cantilever. Inset: Details of the freestanding end. c) Structure in (b) after being fixed to the Au wire (sample substrate). Inset: Details of the welded end. d) The structure in (c) after being subjected to a high-density electrical current flow and subsequent release of the filling material. Inset: Details of a ruptured end.^24^ Reproduced with permission from ref 24. Copyright 2011, Elsevier.

Metal nitrates have frequently been filled into the cavities of CNTs, with *ex situ* methods being the most widely explored. The chosen methodology is determined by the physicochemical properties of the salt. Whereas solution filling has been employed in most cases,^275^ other compounds, such as AgNO_3_, can also be successfully encapsulated using the MPH approach.^426^ As mentioned in the next section, nitrates are often encapsulated into the hosting CNTs as precursors to prepare oxide-filled CNTs.

Special interest has been directed toward the synthesis of hybrids formed by low-dimensional chalcogenide-based materials encapsulated within CNTs. Theoretical studies on filling with tellurides^427^ and using the MPH capillary wetting technique for their encapsulation inside CNTs are widely documented.^14,372,375,428–431^ After encapsulation, CNTs and inorganic guests undergo modifications in their electronic properties, atomic coordination and morphology,^432^ leading to the formation of nanostructures that are unstable in their bulk form.^62,377,431^ Reports include the modification of the band structure of SWCNTs due to the presence of MnTe_2_ inside their inner cavities,^428^ which was similarly observed in HgTe@SWCNTs by Sloan et al. HgTe presented a novel structure after filling, with Hg adopting a trigonal planar geometry.^433,434^ The experimental results are in agreement with the DFT calculations, which predict the distortion of the Hg–Te angles induced by encapsulation within narrower SWCNTs.^132^ Furthermore, the structural variations of the guest can be associated with the electrical modifications observed in the nanomaterial. In a more recent study, HgTe NWs were encapsulated within very narrow (*d* < 1 nm) metallic and semiconducting SWCNTs by the diffusion of melted inorganic material into the host. After filling, the formation of a zigzag and monoatomically thin HgTe nanostructure was favored, regardless of the NT chirality. Optical absorption spectroscopy (OAS) allows the investigation of charge transfer in filled SWCNTs. Figure 13 a) shows the UV–Vis–NIR spectra of both unfilled and HgTe filled semiconducting (blue lines) and metallic (red lines) SWCNTs. In case of the semiconducting samples, the observed absorption lines in the regions 645–650 nm and 1120–1130 nm (S22 and S11 transitions) were associated with a (7,6) chirality, while the absorption lines located within the 400–500 nm range correspond to vibrations characteristic of metallic CNTs (M11 excitonic transitions). The confined species significantly alter the optical and electronic properties of the host. Electron transfer from the HgTe NWs to the host, suggested by the downshifts observed in the high-frequency modes in the Raman spectra, showed enhanced phonon–electron coupling for metallic SWCNTs and resulted in enhanced fluorescence of some filled semiconducting SWCNTs, such as (7,5) and (9,4).^435^

**Figure 13:**
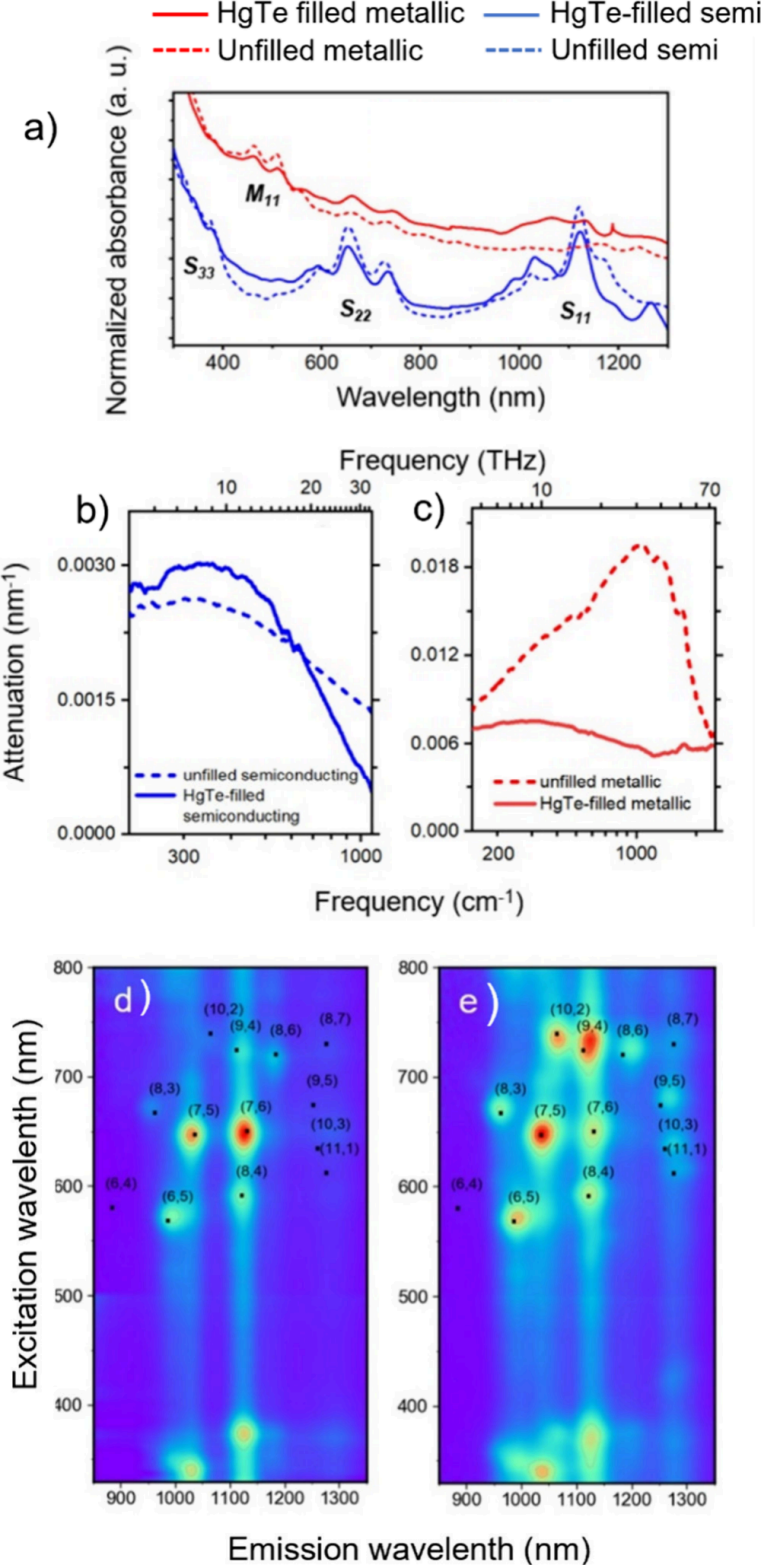
Modification of the photoelectronic properties of HgTe-filled chiral SWCNTs. a) UV–vis-NIR absorption of pristine and HgTe-filled SWCNT solutions enable discerning between metallic (red lines) and semiconducting (blue lines) hosts owing to the presence of characteristic absorbance lines. b–c) RT- IR attenuation for semiconducting and metallic SWCNTs. Dotted curves correspond to pristine SWCNTs included as reference. d–e) PLE maps of the pristine and HgTe-filled SWCNTs, respectively.^435^ Reproduced with permission from ref 435. Copyright 2022, American Chemical Society.

Figure 13 d–e) shows the PL spectra of the pristine and HgTe-filled SWCNTs. Unlike the negligible variations observed for the equilibrium charge carrier density in semiconducting SWCNTs, their metallic counterparts undergo an effective decrease in the free carrier density when filled with HgTe NWs, as established by the reduction in the intensity of the signal appearing in the far-infrared (FIR) attenuation spectra, along with the observed red shift (see Figure 13 b–c)).

In 2013, Giusca et al. encapsulated GeTe, a material of interest for memory devices, within SWCNTs with diameters of less than 1.3 nm. Important variations in the structural characteristics of the filler were reported, ranging from an amorphous to a crystalline phase under the influence of the TEM electron beam, and adopting a rocksalt structure once encapsulated. A decrease in the melting point of GeTe was also observed in this study. Phase-changes at the nanoscale are promising for future applications in phase switching because of the reduction in writing/erasing currents.^436^

Further examples of the modification of the structure and electronic properties by encapsulation of TMCs, include the formation of two types of tetrahedral 1D chains of GeX_2_ (X = S or Se), whose connectivity and stability closely depend on the diameter of the hosting tubes^68^ or the expansion of the CNTs’ band gap upon being filled with SnSe.^437^ SnSe adopts an orthorhombic *Pnma* form in bulk, but it can grow with a Fm3m configuration when it crystallizes inside SWCNTs with diameters less than 1.4 nm. The packing of the atoms corresponds to SnSe 2 × 2 bilayers, which structure can be distorted depending on the diameter of the host. Other unprecedented SnSe structures were grown within the cavities of SWCNTs when going from narrower to medium diameter CNTs (0.7–1.3 nm) using a vapor-phase approach. Linear dipole single atomic chains **a)**, zigzag **b)**, 2 × 1 chains **c)**, 2 × 2 cubic **d)**, and a new structure with 6-fold symmetry (MoSe-like SnSe structure) **e)** were observed (Figure 14).^58^

**Figure 14:**
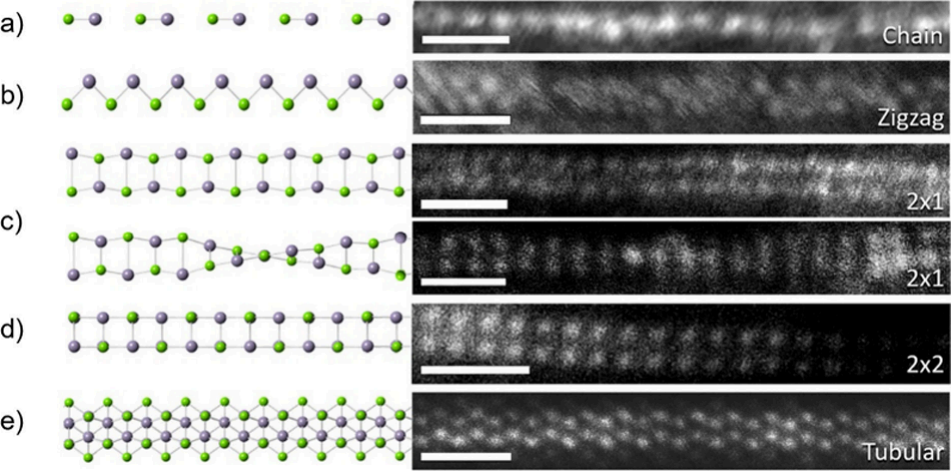
SnSe NWs encapsulated inside SWCNTs. ADF-STEM images of the observed forms of SnSe NWs ordered from narrow to medium diameters of the host, along with their structural models. a) Linear dipole chain, b) zigzag, c) 2 × 1, d) 2 × 2 cubic, and e) MoSe-like SnSe structure. Scale bars are ∼1 nm.^58^ Reproduced with permission from ref 58. Copyright 2019, American Chemical Society.

By combining DFT studies and Raman spectroscopy, Faulques et al. monitored the variations induced in SnSe when confined within SWCNTs.^438^ In agreement with the theoretical calculations, additional vibrational bands appeared in the low-frequency Raman region of the SnSe-filled SWCNTs. A decrease in the signal-to-noise ratio of the G band, accompanied by a blue shift, was observed, along with an increase in the D band. These results suggest an increased disorder within the conjugated carbon lattice induced by the encapsulated compound. The specific modes that appeared after the confinement of SnSe were closely dependent on the structure adopted by the crystals, with specific modes for 2 × 2 structures (151 cm^–1^ and 185 cm^–1^) or their hexagonal counterparts (235 cm^–1^).

Despite a large number of materials that can be filled inside CNTs using the MPH technique, the synthesis of continuous 1D nanostructures of high-melting point materials is still problematic. Taking advantage of the documented nanoreactor characteristics of CNTs, Eliseev et al. developed a new approach to synthesize 1D TMCs nanocrystals within SWCNTs, structures that had not been confined inside the CNTs by conventional impregnation methods. By encapsulating low-melting point precursors and their subsequent reactions, the authors were able to grow MS, MTe and MSe (M = Cd, Zn, Pb) inside the tubular carbon-based hosts,^439^ thus contributing toward the formation of new nanohybrids with unprecedented nanostructures.

#### 7.1.4 Oxides

The first examples of CNTs filled with metal oxides resulted from strategies employed to open the tips of the CNTs.^156,162^ Additional examples involve the encapsulation of oxides during the synthesis of the CNTs.^440–442^ Single- and multistep protocols that employed solution filling in aqueous media of non-oxidized metal precursors and subsequent calcination under oxidizing conditions were used to prepare a variety of M_*x*_O_*y*_@CNTs (M = Sn, Zn, Ni, Sm, Nd, Co, Pd, Cd, Fe, Y, La, Ce, Pr, Eu, V, U, and Nb).^275,443–450^ Other approaches include the use of acidic solutions to introduce transition metals^451^ or lanthanide oxides^452^ into CNTs or a wet impregnation approach assisted by sonication to increase the solubility of the metal oxide to be filled.^453,454^ The use of mixtures of metal nitrates or acetates in the case of Mn^455^ and a variety of other reactants^456^ has been explored to encapsulate mixed metal oxides, namely FeBiO_3_, NiCo_2_O_4_, LaCrO_3_, MgCeO_3_, LaFeO_3_, and CoFe_2_O_4_ into the cavities of CNTs (Figure 15 a)).^457^ Filling with concentrated solutions of bimetallic acids, metal iodides, and organometallic compounds led to the formation of crystalline structures inside CNTs. The oxidation of the guest is required to prepare the corresponding metal oxides, and consequently, lower filling yields are usually obtained. Controlling the pH of the solution allowed the formation of metallic oxychloride/oxide mixtures. Some examples include the direct encapsulation of metal oxides by solution filling, melting, or vapor-phase techniques. Several resulting hybrid structures have been reported, including simultaneous filling and external coaxial coating **b)**,^458,459^ filling **(c–d))** and decoration with NPs, and interlayer filling between the walls of the CNTs.^460^

**Figure 15:**
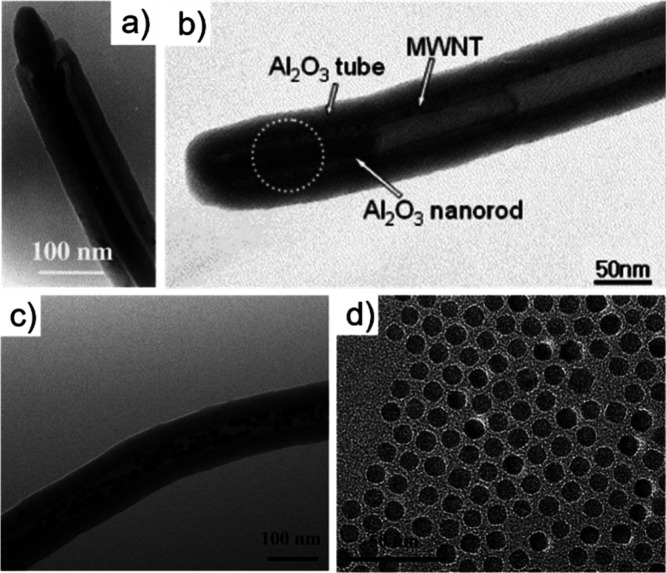
Examples of metal oxides filled within CNTs. TEM images of a) CoFe_2_O_4_@MWCNTs prepared by filling the host with a mixture of nitrates of the corresponding metals.^457^ b) Al_2_O_3_ filling (rods) and coating of MWCNT.^459^ c) MWCNT filled with iron oxide. The formation of magnetic NPs d) is favored over other types of inner nanostructures.^461^ Panel a) is reproduced with permission from ref 457. Copyright 2004, Elsevier. Panel b) is reproduced with permission from ref 459. Copyright 2003, Elsevier. Panels c–d) are reproduced with permission from ref 461. Copyright 2013, Royal Society of Chemistry.

Wilson et al. reported the heterogeneous encapsulation of a Gd^3+^-cluster,^462^ and later, using an analogous protocol, they prepared BiOCl/Bi_2_O_3_@US-tubes.^463^ The MPH filling of materials with low-surface tension induces the formation of p-block metals^464–466^ and transition metal oxide structures, such as V_2_O_5_ ^467^ MoO_3_ ^449^ or Re_*y*_O_*x*_ NPs.^468^ The reduction of Mo can be achieved easily by annealing the sample under H_2_ at 500 °C.^469^ Air-sensitive halides can be encapsulated by melting under inert conditions and subsequently calcined, as mentioned before.

Special efforts have been directed toward the encapsulation of magnetic and magnetoelectric species inside CNTs.^451,460,470^ Several reports have described the encapsulation of different iron oxide nanostructures, exploring a wide range of applications in different fields, such as biomedicine,^471^ water purification,^472^ evaporation,^473^ and energy storage.^445,474,475^ The most commonly used strategy consists in filling CNTs with non-oxidized Fe precursors, followed by annealing under controlled conditions.^291,294^ MWCNTs filled with magnetic NPs are formed *in situ* during the synthesis of CNTs,^461^ and as it has been already mentioned previously, in most cases, these guest nanostructures are usually considered undesired impurities that need to be removed depending on the targeted applications. The desired filling with continuous NRs has also been reported.^476^ Using a simple protocol, Korneva et al. successfully filled CNTs with Fe_3_O_4_ NPs (magnetite, 10 nm) contained in commercially available ferrofluids. This technique allowed a higher loading of the F_3_O_4_ NPs inside the CNTs. Unfortunately, the weak magnetic signal is the main drawback of the application of the synthesized hybrids.^477^

One particular advantage of CNTs as hosts is the encapsulation of POMs within their cavities. POMs are inorganic anionic clusters composed of early transition metal oxides with potential applications in catalysis and as molecular magnets. In a recent study, Cui et al. employed a liquid-phase approach to confine Keggin-type POM (PMo_12_) within SWCNTs. The electron- acceptor character of PMo_12_ induces a substantial narrowing of the SWCNTs’ band gap, which facilitates the absorption of light by the CNTs, while the isolation of the cluster enhances its use as a photothermal agent, which is otherwise restricted by its high solubility in water.^473^

### 7.2 Carbon Allotropes

Carbon allotropes are undoubtedly the family of materials with the greatest impact on filled CNTs. In 1998, C_60_ was the first example of a macromolecule successfully inserted into a CNT,^146,478^ and its presence was subsequently confirmed by UV–vis analysis.^479^ Although this discovery was accidental and took place while synthesizing CNTs using a PLV method (Figure 16 a)), it led to countless reports and studies^480,481^ on CNTs synthesized by different routes containing fullerenes.^482,483^ Hereafter, a variety of reports have been published on the encapsulation of carbon nanostructures inside CNTs, which include carbon NWs, graphene nanoribbons (GNRs), and *sp* ^3^ compounds such as adamantane (Figure 16 c)).

**Figure 16:**
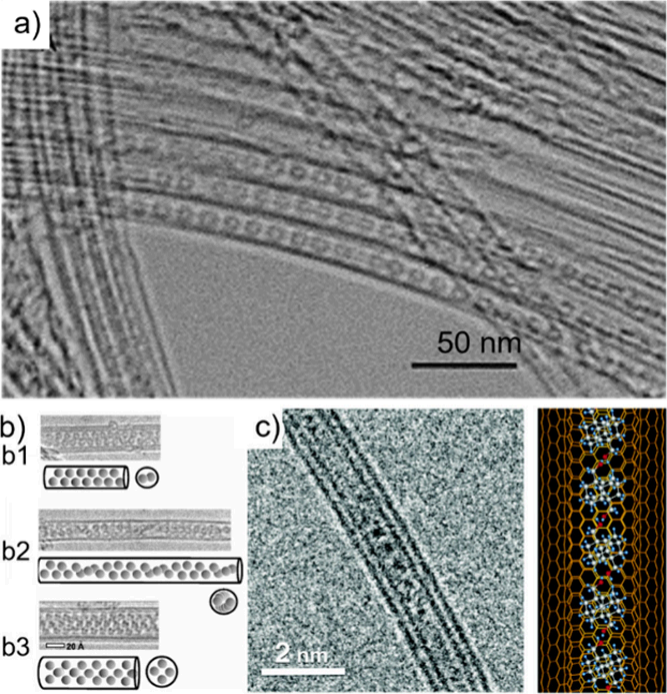
Encapsulation of fullerenes within CNTs: Formation of “nanopeapods”. a) “Nanopeapods” observed in a sample of SWCNTs synthesized by a pulsed laser deposition method^479^ and b) HRTEM micrographs and schematic representation of b1) C_60_@DWNTs zigzag phase; b2) C_60_@SWCNT chiral phase; b2) two-molecule layer phases.^484^ c) TEM image and the corresponding scheme of functionalized adamantine filled inside a DWCNT.^485^ Panel a) is reproduced with permission from ref 479. Copyright 1999. Elsevier. Panel b) is reproduced with permission from ref 484. Copyright 2004, American Physical Society. Panel c) is reproduced with permission from ref 485. Copyright 2018, Royal Society of Chemistry.

#### 7.2.1 Fullerenes

Sublimation filling is the most widely used and efficient method for the formation of “nanopeapods”, a term coined for the structure resulting from the encapsulation of fullerenes within CNTs. The vaporization of fullerenes is usually obtained via thermal treatment above 400 °C, pulsed laser deposition^146^ or plasma-ion irradiation.^486,487^ Afterward, the filler migrates in its vapor phase to the CNTs and enters into their cavities by capillarity, thus forming highly ordered arrays, which can occupy up to 100% of the inner surface of the hosting nanostructures (Figure 17 a)).^488–490^

**Figure 17:**
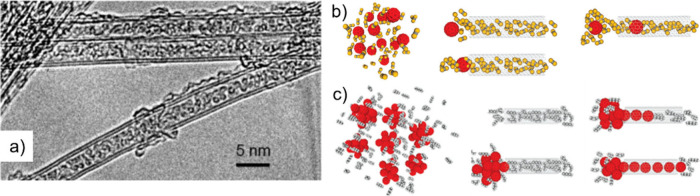
Adsorption dynamics of C_60_ into CNTs. a) TEM image of DWCNTs filled with C_60_ by a vapor-phase reaction. C_60_ adopt a multilayer configuration due to the diameter (2.5–3 nm) of the hosting tubes.^490^ The spontaneous encapsulation of C_60_ in liquid phase within CNTs has been explained via the approach of b) single molecules or c) molecular clusters, which nature is mainly ruled by the characteristics of the solution (concentration and solubility of the solute in the solvent).^491^ Panel a) is reproduced with permission from ref 490. Copyright 2007, Elsevier. Panels b–c) are reproduced with permission from ref 491. Copyright 2010, American Chemical Society.

Taking advantage of the ability of fullerene derivatives to modify their electronic properties,^492,493^ externally functionalized fullerenes,^43,494–497^ endohedral metallofullerenes,^82,498–509^ azafullerenes^510–514^ and many other buckyballs have been reported to be encapsulated into CNTs. However, the formation of peapods containing these species requires adopting synthetic approaches that do not affect the stability of the guest molecule.^42,490,515–519^ Liquid-phase synthesis has emerged as an alternative for this purpose.^520^ Aspects such as the chemical nature of the solvent, the transport dynamics in solution, and the three-component interaction between CNTs, buckyballs, and solvent might play a role in the encapsulation process.^42,519^ Since the affinity with the host and the ability of the solvent molecules to enter into their cavities might hinder the formation of fullerene arrays inside the CNTs, their size, shape, and solvation properties should be considered. In general, this approach requires careful selection of a solvent that favors the migration of fullerenes toward the host tubes. In solution, buckyballs can approach CNTs either as discrete molecules or as agglomerates. When the number of solvent molecules largely exceeds that of the solute, the solute will be highly solvated and will approach the CNT as a single entity. In this case, the solvent can also enter into the host and, if such molecules are located between two C_60_, get trapped inside, thus diminishing the area available for their encapsulation. Therefore, to successfully compete with the solvent, the concentration of C_60_ in the solution should be substantially increased. Conversely, when a solvent in which C_60_ is poorly soluble is employed, molecular clusters can be formed. C_60_ would then approach to the CNTs as complex arrangements able to block (or at least to limit) the encapsulation of solvent molecules inside the CNTs, thus increasing the C_60_ filling rate (Figure 17 b–c)).^491^

Treatment under supercritical conditions (at RT or mild heating) was proposed by Khlobystov et al. to efficiently encapsulate C_60_ within CNTs using CO_2_.^517^ This methodology was proposed to counter the negative effect of the solvent used as a vehicle to transport the buckyballs within the interior of the CNTs in a liquid solution. Unlike conventional solvents (under normal conditions), supercritical CO_2_ (scCO_2_) provides higher diffusivity to C_60_ but also favors their precipitation (due to their diminished solubility in scCO_2_). Molecular simulations suggest that the encapsulation of C_60_ using scCO_2_ occurs via the formation of nanoclusters formed by eight particles ((C_60_)_8_) that approach the CNTs ends and spontaneously enter their cavities one by one. According to the calculations, when larger aggregates are formed, the encapsulation requires longer periods, and the extra molecules tend to deposit onto the external surface of the CNTs.^113^ The reaction, which experimentally proceeded between 30 and 50 °C, yielded up to 70% and emerged as an alternative for the encapsulation of *T*-sensitive molecules.^517^ Other supercritical fluids, including ethanol, methanol, and toluene, have been reported to successfully promote the encapsulation of fullerenes and metallofullerenes (C_60_, C_70_, C_78_, C_84_, Gd@C_82_, Y@C_82_, Er@C_82,_ or La@C_82_) within SWCNTs. In these cases, the *T* used for the reaction corresponded to the critical temperature (*T*_*c*_) of the solvents. After 5 min under 30 MPa, the authors estimated filling yields of up to 90%.^521^ It is beyond the scope of this review to provide a detailed description of all examples available in the literature regarding the filling of CNTs with fullerenes. For this, the reader is directed toward some excellent available reviews.^488,489^

The encapsulation of fullerenes within CNTs is, in most cases, a spontaneous and irreversible process that can occur when the difference in diameter between the hosting CNTs and guest molecules is greater than 0.6 nm. This is due to favorable vdW forces between the two structures and a perfect geometrical match, providing the perfect environment for a successful encapsulation.^36^ In this way, and depending on the diameter of the host CNT, a number of different conformations for the C_60_ molecules are available, including zigzag, double helix, two-molecule layer (Figure 16 b))^484,522^ or dimers.^523,524^ Most of the reports correspond to the encapsulation into SWCNTs with inner diameters of ca. 1.3–1.9 nm. However, the encapsulation into narrower CNTs cannot be discarded, since theoretical studies indicate that C_60_ can be filled into CNTs down to a 6.4 Å diameter,^525^ but smaller particles (C_20_ and C_28_) are also susceptible to be encapsulated into CNTs of different inner diameters.^522^ It is noteworthy mentioning that, under exposure to an energy source, such as a high electron flux in TEM or high-temperature annealing (up to 1200 °C),^526–528^ these structures undergo rearrangements and coalesce, eventually resulting in the formation of a continuous NT within the encapsulating host. Therefore, DWCNTs result from this treatment.^478,482^ This new inner “carbon wall” differs from the external cylinder since it could consist of five-, six-, seven- or eight-membered rings (depending on the nature of the former fullerene molecule) and the stability of the new nanostructure strongly depends on its coalescence mechanism.^529^ When fullerenes are confined within CNTs with larger diameters, irregularly arranged clusters can also be formed.^173,530^

Both the filling rate and configuration adopted by fullerenes within CNTs greatly depend on the chemical nature of the guest molecules. In the same way in which functionalized fullerenes can tune the properties of the CNTs; the encapsulation process can be affected by the functional groups attached onto their surface, or the groups encaged within their cavities, which are able to influence the way in which the molecules accommodate inside the hosting tubes.^531^ Attaching non-planar functional groups onto the fullerene surface, for instance, limits the intermolecular close packing, and controlling the length and nature of the attached groups allows the directed formation of ordered molecular chains with a defined distance between the fullerene cages.^532^

Nanopeapods formed by encapsulating fullerene derivatives into CNTs can also undergo transformations owing to coalescence. 1D confinement can modify the polymerization process, as in the case of fullerene epoxides, which, when filled into SWCNTs and heated at 260 °C, react to form linear polymeric structures, unlike the branched polymer resulting from the reaction of free C_60_O. This demonstrates that CNTs are useful not only as templates, but also as directing agents to synthesize new nanostructures.^533–535^

#### 7.2.2 1D Arrays

Zhao et al. reported the presence of carbon NWs, consisting of 1D linear chains of more than 100 carbon atoms inside the cavities of MWCNTs.^536^ These new carbon-based species, carbon NWs are composed of both *sp* ^3^ and *sp* ^*2*^ bonds, and the hybrid material results from a hydrogen-modified AD method for the production of CNTs. Their synthesis using high-*T* treatments has also been reported.^537^ As in the case of carbon NWs, the encapsulation of polyynes, linear arrays of carbon atoms with *sp* bonding, into SWCNTs or DWCNTs^538^ was found to be very useful to stabilize these carbon chains, that otherwise are difficult to isolate due to their high susceptibility to polymerization.^86^ Polyynes (C_10_H_2_) can be prepared by the laser irradiation of graphite particles. The C_10_H_2_@CNTs hybrids were obtained by heating a mixture of the precursors under vacuum at mild *T* ^539–541^ or by atmospheric AD.^86,542,543^ Using different synthetic approaches, linear carbon chains of diverse lengths have been achieved. For example, Shi et al. were able to confine long linear carbon chains, up to six thousand contiguous *sp* hybridized carbon atoms, within DWCNTs, using high *T* and high vacuum treatment.^93,538,544^ More recently, the stability and charge transfer was investigated for linear carbon chains encapsulated in SWCNTs.^87,545^

#### 7.2.3 Graphene Nanoribbons (GNRs)

GNRs stand out among graphene derivatives and related materials because of their remarkable properties. These nanostructures consist of a single-layered *sp* ^*2*^ carbon network with a quasi-1D morphology, forming strips with a high length-to-width ratio. The emerging interest in these materials arises from their well-known transport properties (from the graphene-like structure), QC induced by their narrow lateral dimensions, leading to tunable gaps with interest in the field of nanoelectronics, and their high surface area, allowing them to undergo functionalization or be loaded with drugs or biomolecules, thus expanding their possible applications in the biomedical field.^546^ Depending on their structural configuration (armchair or zigzag), which also determines their optoelectronic properties,^547^ GNRs can be extraordinarily reactive, also tending to form aggregates due to self-stacking.^546^ As an alternative for stabilizing these nanostructures, both theoretical^111^ and experimental reports have explored the encapsulation process of GNRs within the narrow cavities of SWCNTs. Tazylin et al. reported the preparation of H terminated GNRs by vapor-phase filling of the CNTs with polycyclic aromatic hydrocarbons, namely, coronene or perylene, and the subsequent thermal fusion of the guest molecules at *T* ranging between 350 and 530 °C, with the best results obtained when the system was annealed at 400 °C. The dimensions of the GNRs were limited by the inner surface of the CNTs, acquiring either helical or twisted configurations (Figure 18 a)).^94^ One additional report included the use of dicoronylene as a precursor for the synthesis of similar hybrids.^548^ The encapsulation and subsequent polymerization of coronene to form GNRs induced strain to the CNTs’ structure, as confirmed by the red-shift and widening of the absorption peaks of the sample.^549^ This behavior is diameter-dependent, going from 18 to 8 meV for CNTs of 1.3 to 1.9 nm (Figure 18 b–c)). Chernov et al. evaluated the PL of GNRs@SWCNTs synthesized by encapsulating coronene using the vapor-phase filling and a low *T* approach (polymerization occurring below 450 °C), the latter leading to the formation of less ordered and shorter ribbons. Similar to previous studies, the absorption spectra of both the high *T* and low *T*-treated samples underwent a red shift compared to the pristine CNTs. The bandgap modification was closely dependent on the geometry of the tubes. When using semiconducting tubes, the empty sample emitted with higher intensity in the case of (11,9) and (14,6) species, while the GNRs@SWCNTs prepared at low and high *T* showed the brightest emission areas in the case of the (14,6) and (13,8), respectively, thus suggesting that the distortions occurring to the material by the encapsulation of nan-oribons are largely geometry dependent (Figure 18 d)).^17^

**Figure 18:**
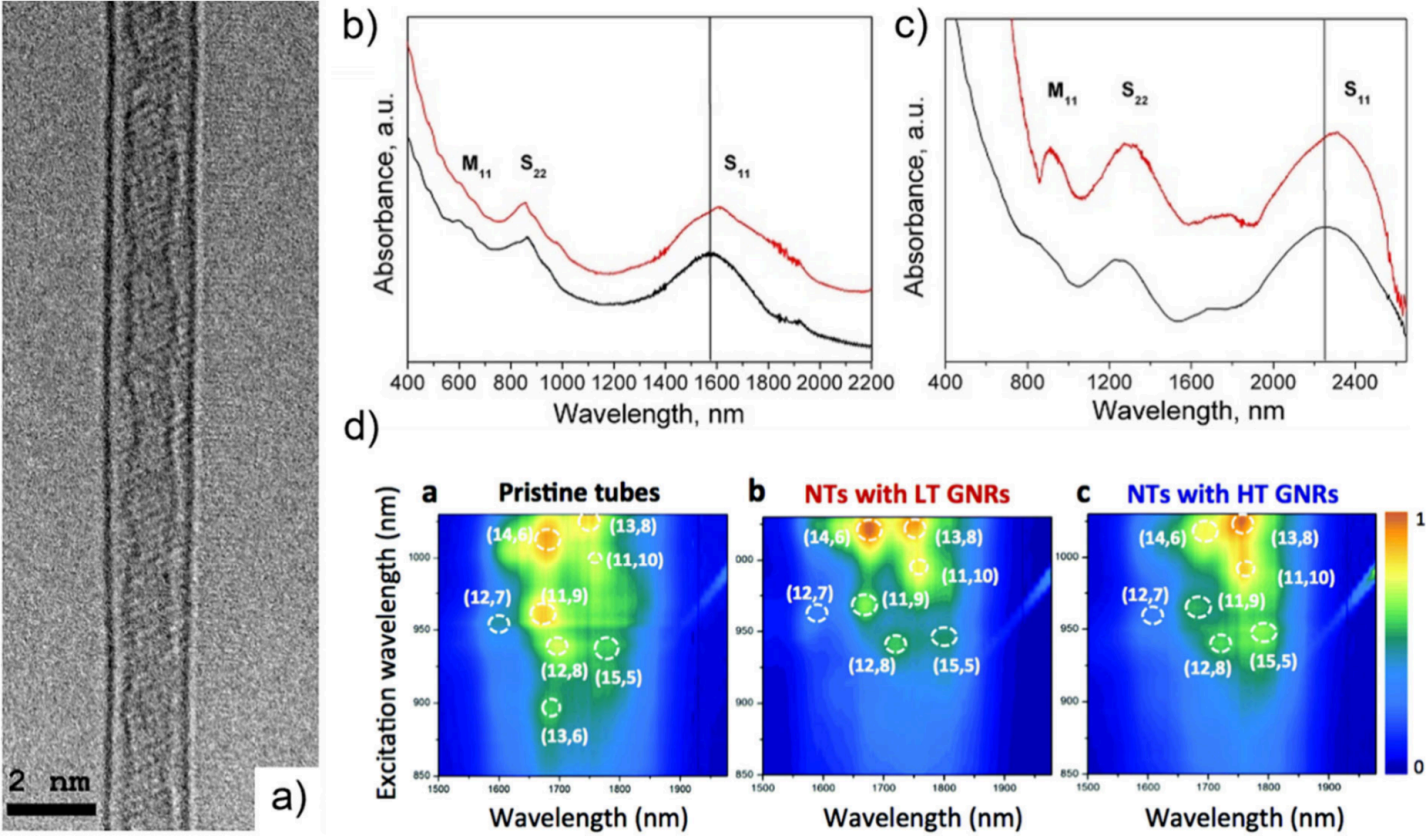
GNR encapsulated within CNTs. a) TEM image of a GNR@SWCNT. The sample was prepared by filling the CNTs with vaporized coronene.^94^ Diameter-dependent red shifts were observed in the absorption bands after confinement of GNRs in b) 1.3 nm and c)­1.9 nm SWCNTs. The red continuous lines correspond to the GNRs@SWCNTs. The spectra of empty SWCNTs are included as references (black continuous lines).^549^ d) PL contour plots of pristine SWCNTs, low *T*-grown GNRs@SWCNTs (LT GNRs), and high *T*-grown GNRs@SWCNTs (HT GNRs).^17^ Panel a) is reproduced with permission from ref 94. Copyright 2011, American Chemical Society. Panels b–c) are reproduced with permission from ref 549. Copyright 2013, American Chemical Society. Panel d) is reproduced with permission from ref 17. Copyright 2018, Royal Society of Chemistry.

Other approaches include the encapsulation of three rows of consecutive hexagonal rings formed after exposure to an 80 kV electron beam of SWCNTs and C_80_ functionalized with a sulfur-containing organic moiety (to provide heteroatoms able to stabilize the edges of the nanoribbon),^535^ or to high *T* treatment of SWCNTs in the presence of FeCp_2_.^550^ Finally, Cadena et al. proposed a liquid-phase approach followed by annealing to induce the formation of nanoribbons inside CNTs.^551^ For this purpose, the authors pretreated the CNTs to clean, open their tips, degas, and further sonicated them in the presence of 1,2,4-trichlorobenzene. After encapsulation of the aromatic moieties, the sample was annealed up to 1100 °C, thus inducing the formation of several tens of nm nanoribbons confined within CNTs.

#### 7.2.4 *sp* ^3^-Hybridized Nanostructures

Although in a lesser proportion, diamond-like structures were also encapsulated within the CNTs. Adamantane is a building block of the diamonoid family, which includes cage-like *sp* ^3^ hybridized carbon nanostructures such as diamantine, triamantane, and tetramantane. They have outstanding properties, including a high band gap and high thermal stability, and can be functionalized to expand their applications. The encapsulation of adamantane within both SWCNTs and MWCNTs has been reported to occur under mild conditions (190 °C), with the filling rate depending on the CNT diameter. Unlike fullerenes, ordered arrangements of adamantane molecules were not observed. They become static after encapsulation, which is favored in CNTs with diameters greater than 1.2 nm. Thermal treatment of the hybrid can also induce transformation of the guest molecules, leading to the release of adamantane, which again depends on the dimensions of the host.^552^ The formation of ordered C linear arrays was then reported after annealing DWCNTs filled with adamantane at 300 °C.^553^ The authors found that the encapsulation of adamantane within (7,7) CNTs consisted in an endothermic process with a wider energy gain when CNTs narrower than 0.8 nm were employed. In fact, adamantane molecules were not observed in the HRTEM images of CNTs with this diameter and below. Otherwise, the 1D arrays were formed by molecules separated by ∼6.2 Å with a 0.16 eV binding energy. The diameter of the host also ruled the number of encapsulated adamantane linear arrays, with 2 and 3 of them located inside ∼1.4 and ∼1.8 nm, respectively. Similar behavior was observed after encapsulation of diamantane-4,9-dicarboxylic acid (Figure 19 a–b)).^554^ Functionalized adamantane molecules, namely 1-adamantanemethanol and 1-bromoadamantane, were also vaporized and filled into CNTs. Shifts induced in the spectra (that is Raman G-mode and optical absorption) due to charge transfer produced by the migration of bromine atoms from the diamonoid were observed in the CNTs filled with 1-bromoadamantane.^555^

**Figure 19:**
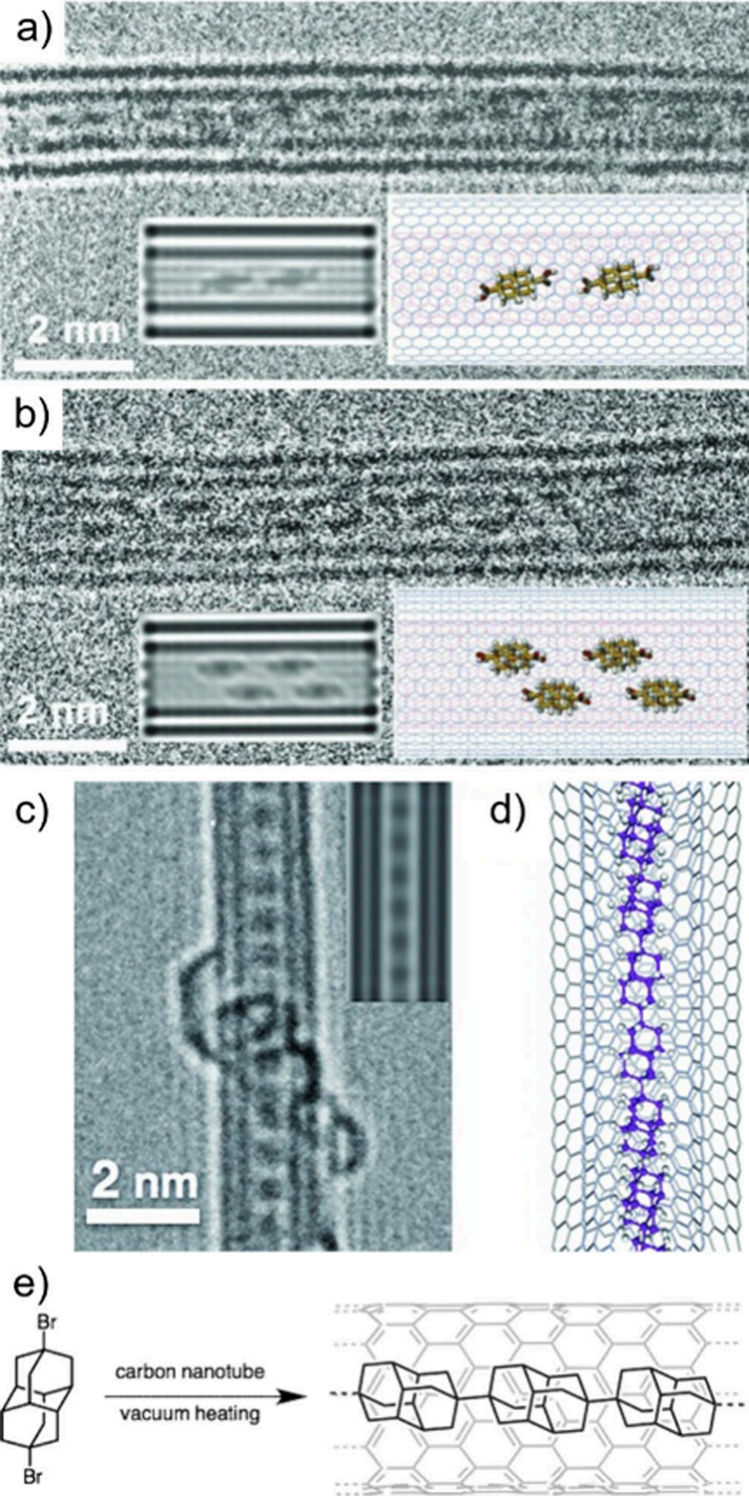
DWCNTs filled with linear diamond-like arrays. HRTEM images (along with the theoretical simulations) of a) 1 linear array and b) 2 linear arrays encapsulated within a ∼1 nm and a ∼1.3 nm inner diameter host.^554^ c) HRTEM and d) simulated image of a diamantane nanodiamond polymer confined in a DWCNT. e) Schematic representation of the formation of diamond-based linear arrays by encapsulation of 4,9-dibromodiamantane within CNTs.^556^ Panels a–b) are reproduced with permission from ref 554. Copyright 2013, John Wiley and Sons. Panels c–e) are reproduced with permission from ref 556. Copyright 2015, John Wiley and Sons.

Nakanishi et al. reported a novel approach for the formation of linear chains of nanodiamonds using CNTs as templates. The authors sublimed apical-bridgehead-halogenated diamantane (4,9-dibromodiamantane) inside SWCNTs and DWCNTs (Figure 19 c–e)). The thermal removal of bromine atoms and subsequent formation of *sp* ^3^ C–C bonds led to the formation of diamantane-linear structures.

The functionalization of adamantane increases the filling yield, which is otherwise unfavorable because of the low affinity of the molecules for the inner walls of the CNTs. A spontaneous alignment of these molecules due to the formation of H bonding induces the formation of DWCNTs that are densely filled with a 1D network of 1,6-bis­(hydroxymethyl)­diamantane (Figure 16 c)).^485^

### 7.3 Organic and Organometallic Compounds

Doping plays a key role in modulating of the electronic properties of CNTs. Both *n*-type and *p*-type doping are of interest for the application of CNTs in molecular electronics. Among many other strategies, the introduction of nucleophilic molecules in semiconducting-type CNTs can lead to controllable *n*-type doping, as previously demonstrated for fullerenes.^493^ The simplicity of the encapsulation process compared to other approaches, along with the possible mass production, makes this strategy promising for obtaining CNT-based field-effect transistors (FETs), as it takes advantage of the high carrier mobility of the CNTs. By vapor-phase techniques, SWCNTs were endohedrally functionalized with a variety of organic molecules, including anthracene, pentacene, tetrathiafulvalen and tetracyano-p-quinodimethane (TCNQ).^557^ The liquid-phase encapsulation of 1,1′-didodecyl-4,4′-bipyridinium dihexafluorophosphate (viologen) into metallic SWCNTs induced the successful opening of the band gap of the hosting nanostructures, thus leading to semiconducting hybrids.^558^ Meanwhile, a reflux method using dioxane led to the encapsulation of 2,4-bis­[4-(N,N-diphenyl amino–2,6-dihydroxyphenyl] squaraine (DPSQ) and N,N′-bis­(3-pentyl)­perylene-3,4,9,10-bis­(dicarboximide) (PBI). In this approach, the coencapsulation of a dummy molecule (coronene) allowed the control of the amount of n/p dopant filled within the CNTs.^559^ Therefore, the authors were able to easily tune the properties of the host CNTs.

The ionization energy and electron affinity of the guest molecules play important roles in the optical behavior and charge transfer between SWCNTs and organic molecules.^560^ An additional advantage is that the formed hybrid is highly stable under ambient conditions, and the hosting CNT acts as a physical and chemical barrier, preventing the interaction of organic molecules with reactive external species. This can also be extrapolated to the stabilization of sensitive materials, such as π-conjugated polyenes, which have potential applications in photonics owing to their ultrafast optical response. Unfortunately, they are susceptible to degradation under prolonged ambient exposure. β-carotene (Car), usually employed as model for this group of compounds, has been successfully filled into SWCNTs by solution filling and it was confirmed that the encapsulation can suppress the light degradation of the organic guest.^561^

Organometallic compounds are an important class of chemical entities that contain covalent bonds between carbon and a metal. The electronic, magnetic, and catalytic properties of this family of materials make them good candidates for catalytic reactions, medicinal chemistry, and sensors because of their ability to undergo redox reactions. Classic organometallic compounds include carbonyl complexes and organolithium and Grignard reagents. MCp_*x*_ are one of the most important families of organometallic compounds consisting of a metal atom in the +2 oxidation state, connected to two cyclopentadienyl (C_5_H_5_, Cp) rings. As metal- and carbon-containing species, the initial interest in MCp_*x*_s in NT chemistry was directed toward their use as precursors for the growth of CNTs.^562^ A few years later, the first studies on the encapsulation of MCps in CNTs began to appear in the literature. In 2001, Stercel et al. reported the encapsulation of several MCp_*x*_s (M = Fe, Cr, V, Ru, and W) in CNTs,^563^ both from the liquid and vapor phases. HRTEM images revealed a chain-like arrangement of MCp_*x*_ molecules within the tubular structure, whereas EDX confirmed the presence of M atoms inside the host material. n-type doping has also been observed for CNTs filled with MCp_*x*_ s, with FeCp_2_ being the most studied member of this family.^564–566^ A molecular size effect was observed by Li et al. for the encapsulation of the MCp_*x*_ compounds CoCp_2_ and Co­(EtCp)_2_.^567^ TEM, EDX, and UV–vis spectra were used to assess the encapsulation process, while PL spectra showed an induced red shift in the PL emission upon CNT filling.

Pichler et al. observed a selective enhancement of the PL spectra after FeCp_2_ encapsulation.^568^ Additionally, it was reported that the process was strongly dependent on the CNT diameter and chirality, in which PL signals increased 3-fold for (8, 6) and (9, 5) chiral CNTs. The same group has also reported a series of other studies on CNTs filled with FeCp_2_ ^283,569–574^ and other organometallic compounds.^575^ Due to the potential applicability of these types of nanohybrids, a variety of characterization techniques have been used to evaluate their structural and electronic properties. Near edge X-ray absorption fine structure spectroscopy (NEXAFS) allows for the confirmation of filling and determination of the characteristics of the interactions created between the hosting CNTs and the encapsulated species. Figure 20 a–b) shows the C 1s and Fe 2p NEXAFS spectra of FeCp_2_, SWCNTs, FeCp_2_-filled SWCNTs, and an annealed sample at 900 °C.^576^

**Figure 20:**
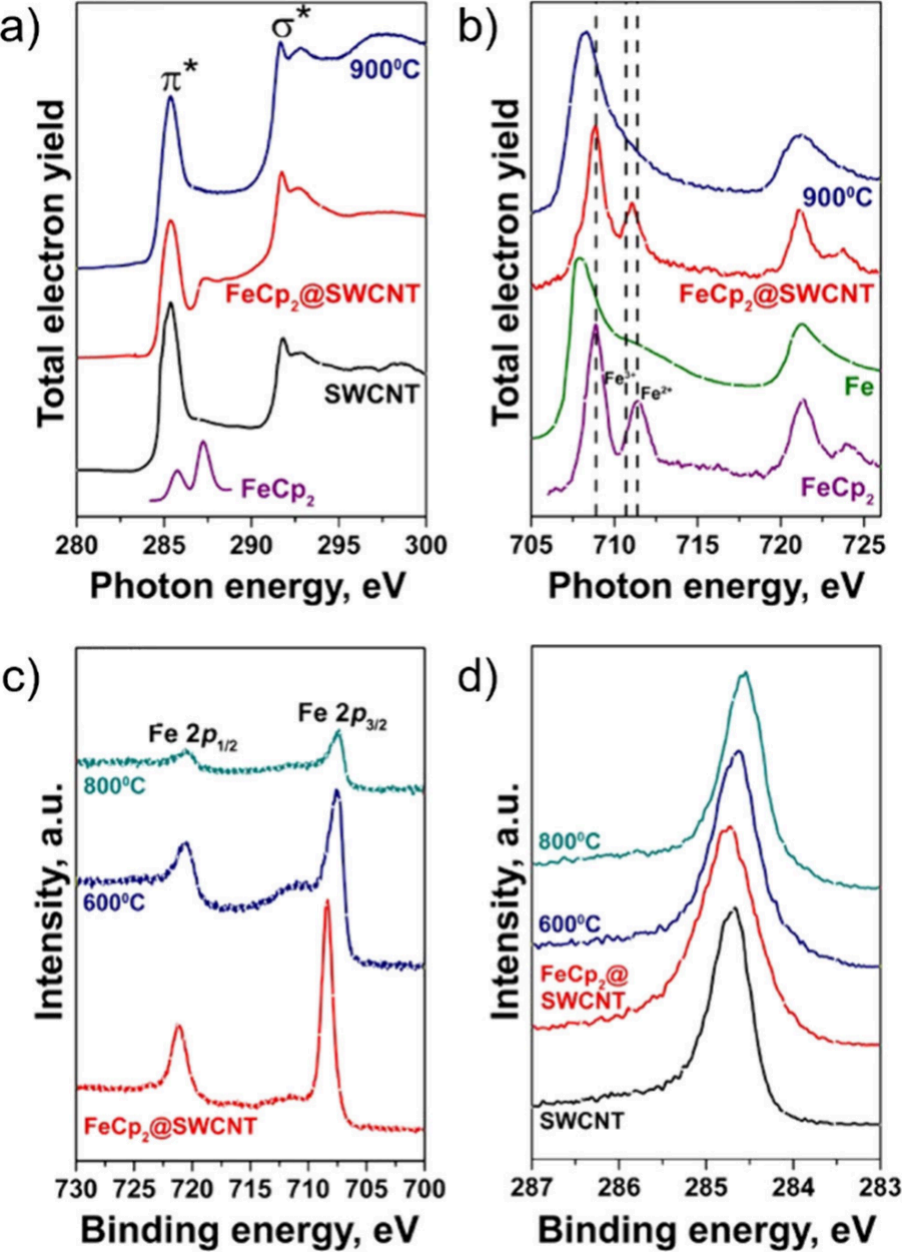
Encapsulation of FeCp_2_ into SWCNTs induces an enhancement of the PL. The a) C 1s, and b) Fe 2p NEXAFS spectra of SWCNTs (black line), FeCp_2_-filled SWCNTs (red line) and a sample annealed at 900 °C for 2 h (blue line), in comparison with the data of pure FeCp_2_. c) Fe 2p and d) C 1s XPS spectra of FeCp_2_-filled SWCNTs and the samples annealed at 600 and 800 °C for 2 h.^576^ Reproduced with permission from ref 576. Copyright 2013, John Wiley and Sons.

By monitoring variations in the photon energy of the elements in the sample, aspects such as the bonding environment, presence of covalent interactions, or variations in the oxidation states can be discerned. In the Fe 2p spectra, slight shifts in the photon energy of the FeCp_2_-filled SWCNTs were observed, which corresponded to changes in the oxidation states of iron +2 and +3. The Fe 2p and C 1s XPS analyses performed on FeCp_2_-filled SWCNTs as well as on samples annealed at 600 and 800 °C for 2 h are registered in Figure 20 c) and d), respectively. As can be seen in the spectra of the analyzed samples, once annealed, the intensity of peaks decreased due to evaporation of Fe from the CNTs. In the case of the C 1s signals, the spectrum of FeCp_2_-filled SWCNTs was shifted toward higher energies, whereas the spectra of the annealed samples were shifted toward lower energies. This testifies to changes in the electronic properties of SWCNTs upon both the filling and annealing processes.^576^

Several theoretical investigations have been conducted on the encapsulation of MCp_*x*_ s in CNTs. For example, Zhao et al. used DFT to study the structural, energetic, and electronic properties of SWCNTs filled with organometallic compounds MCp_2_ (M = Fe, Co, Ni).^79^ The authors found that the stability and charge transfer increased as the adiabatic ionization potential (AIP) of the MCp_*x*_ decreased (CoCp_2_ < NiCp_2_ < FeCp_2_). The same theoretical method was used by Green et al. to investigate the non-covalent interactions between SWCNTs and MCp_*x*_ complexes (M = Fe, Co) as a result of the encapsulation process.^577^ An inverse relationship was observed between the CNT diameter and the binding energy, which depends on the geometrical shape of the MCp_*x*_ with respect to the CNT’s cavity. This diameter-selective encapsulation behavior has been observed by other groups.^567^ Although the initial interest in the encapsulation of MCp_*x*_s and other organometallic species inside CNTs was related to the modification of the electronic properties of the host structure, there are also a few reports dealing with the growth of inner tubes by thermal annealing of MCp_*x*_-filled SWCNTs.

The growth kinetics of SWCNTs inside MCp_*x*_-filled SWCNTs were studied using Raman spectroscopy.^33^ It was shown that the formation consists in two stages: initial growth on a metal carbide catalyst and subsequent growth promoted by a metallic catalyst. Therefore, two growth rates α and β (Figure 21), and activation energies (E_α_ and E_β_ Table 7), which depend on both the diameter of SWCNTs and the chiral angle, were identified.^578^

**Figure 21:**
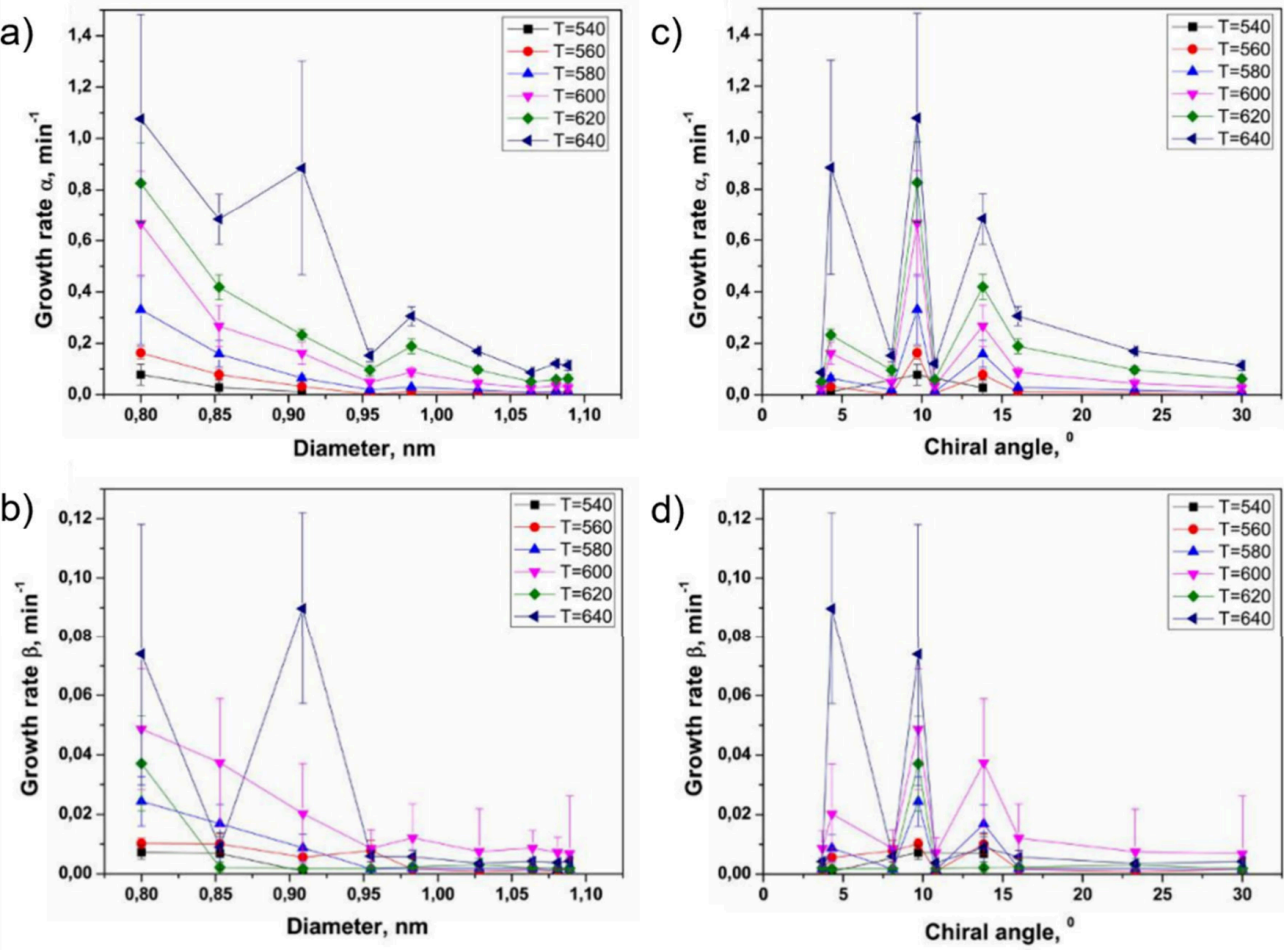
Growth kinetics of SWCNTs inside MCp_*x*_-filled SWCNTs. Dependence of growth rates α and β a–b) on diameter and c–d) chiral angle of grown SWCNTs at different *T*.^578^ Reproduced with permission from ref 578. Copyright 2021, MDPI.

**Table 7:** *E*_α_ and *E*_β_ for the Growth of Individual Chirality SWCNTs Inside Ni­(Cp)_2_, and CoCp_2_-Filled SWCNTs^578^

	Activation energy, eV			
	Precursor			
	NiCp_2_		CoCp_2_	
Inner NT chirality	*E*_α_, eV	*E*_β_, eV	*E*_α_, eV	*E*_β_, eV
(8,8)	2.57 ± 0.16	1.58 ± 0.26	2.64 ± 0.31	0.95 ± 0.52
(12,3)	2.57 ± 0.19	1.74 ± 0.33	2.48 ± 0.09	0.99 ± 0.28
(13,1)	2.36 ± 0.16	1.53 ± 0.21	2.43 ± 0.15	0.77 ± 0.39
(9,6)	2.35 ± 0.11	1.50 ± 0.16	2.43 ± 0.11	0.81 ± 0.33
(10,4)	2.08 ± 0.17	1.49 ± 0.28	2.71 ± 0.13	1.07 ± 0.48
(11,2)	2.30 ± 0.25	1.49 ± 0.33	2.34 ± 0.25	1.31 ± 0.41
(11,1)	1.85 ± 0.21	1.62 ± 0.14	2.28 ± 0.12	1.79 ± 0.85
(9,3)	2.18 ± 0.18	1.91 ± 0.37	1.80 ± 0.25	1.75 ± 0.65
(9,2)	2.17 ± 0.11	1.85 ± 0.62	1.81 ± 0.21	1.63 ± 0.30

The endohedral functionalization of SWCNTs with copper­(II) acetylacetonate (Cu­(acac)_2_) was performed using a nanoextraction approach. Continuous-wave electron paramagnetic resonance (EPR) was used to monitor the encapsulation process. Because EPR measurements allowed the distinction between the encapsulated and non-encapsulated molecules, the stability of Cu­(acac)_2_ inside SWCNTs in different solvents was determined. This technique along with other assessment tools, like polarized Raman scattering, is also useful for determining aspects such as the polar character of the environment, the intermolecular distances and the orientational distribution of the molecules within the CNTs.^579^

Liquid-phase filling of SWCNTs with Cu­(acac)_2_, Pt­(acac)_2_, and a mixture of Cu­(acac)_2_ and Pt­(acac)_2_ has been used for the formation of metal-NW-based devices.^335^ After thermal decomposition (under N_2_) of the immobilized filled CNTs, Cu@SWCNTs, Pt@SWCNTs, and Cu–Pt@SWCNTs hybrids were obtained. These hybrids were subsequently employed for the formation of Cu, Pt, and Cu–Pt NWs, thus demonstrating the versatility of CNTs for the template-assisted synthesis of a variety of inorganic materials.

Spin crossover (SCO) molecules, which undergo variations in their spin states under external stimuli such as *T* or light incidence, have attracted interest in the field of nanoelectronics owing to their ability to act as magnetic switches. However, the low conductivity and relative instability of SCO complexes often hinder their practical application. To overcome these obstacles and potentiate their properties, Villalva et al. encapsulated Fe-based SCO complexes within SWCNTs. The resulting hybrids preserved the magnetic properties of the guest molecules, which underwent spin variations under thermal treatment, with a shift in the SCO transition toward higher *T*. Enhanced properties were observed, including large conductance bistability through the CNTs.^580^

#### 7.3.1 Photoactive Molecules, Chromophores and Luminophores

Charge transport in CNTs is particularly relevant for their use in miniaturized electronic systems. Therefore, tuning the electronic properties of CNTs by external (exohedral) or internal (endohedral) functionalization is an active field of interest. Selecting appropriate molecules to encapsulate in the inner cavity of the CNTs and understanding their effect on their electronic structure are crucial. In recent years, the number of reports on the encapsulation of photoactive molecules, chromophores, and luminophores has increased significantly, indicating the scientific interest in tailoring the electronic structure of CNTs by developing novel host–guest interactions through endohedral functionalization. It is expected that for this type of molecule, the containment effect within the CNT walls will prevent its degradation, which is the main drawback for their application in electronic and photonic devices. In this family of encapsulated organic materials are included photoactive molecules like thiophene and phthalocyanine (Pc) derivatives, chromophore molecules like tetracyanoquinodimethane (TCNQ), tetrakis (dimethylamino)­ethylene (TDAE), squarylium dye, retinal, coronene or Car, and oligomers like α-quaterthiophene (4T), α- quinquethiophene (5T) and α-sexithiophene (6T).^581^ As deeply studied by Forel et al., who encapsulated squaraine dye within semiconducting chirality-sorted SWCNTs, the configuration adopted by the dyes, and therefore the final properties of the nanohybrid, depend on a variety of parameters, mainly related but not limited to the morphology and electronic configuration of the host. These include the CNTs diameter, chirality, and the mutual interactions between the confined molecules. The synthetic approach may also play a relevant role.^582^

Car, an organic pigment in plants and animals belonging to the carotenoid family, is probably the most studied molecule for hybrid optoelectronic devices. Several studies have focused on the confinement of Car into CNTs. Special emphasis was placed on the conformation of the organic molecule within the CNT’s cavity, as this can provide valuable information about the energy transfer mechanism and dictates its optical properties. Yanagi et al.^561^ were pioneers in the encapsulation of Car inside SWCNTs. Although this organic molecule shows a large third-order optical non-linearity,^583^ its practical application is limited by its facile degradation under ambient conditions. Therefore, confinement and protection within the CNT’s cavity can provide an ideal environment for this material for its application. Raman spectroscopy was explored to verify the successful encapsulation in three different types of CNTs: laser vaporization (LV), high-pressure CO (HiPco) and purified HiPco (p-HiPco). Results of Raman spectra of Car and p-HiPco, before and after filling, suggested effective encapsulation on p-HiPco CNTs (with their terminal ends open). Raman spectra of post-processed (after encapsulation) LV CNTs also show several peaks corresponding to the organic molecule, confirming its presence in the internal cavity of the CNTs. The Car content inside the CNTs was approximately 3 wt %, corresponding to a filling rate of about 30%, assuming that Car molecules are arranged in a straight line. Finally, the UV irradiation stability of the isolated versus encapsulated Car was measured, with the absorption band of Car inside the CNT being retained after irradiation. This indicates that the light degradation of Car does not occur when it is encapsulated in the cavity of the NTs. It is worth mentioning that degradation of the Car structure within the CNTs during HRTEM analysis, XRD, and optical absorption spectra were explored to further investigate the structure and stability of Car inside the SWCNTs, and PL to reveal the energy transfer from Car to the CNTs.^584^ The results obtained corroborated the initial idea that encapsulated Car molecules align parallel to the CNT axis and a light-harvesting function is exhibited by photoexcitation energy transfer to the CNTs. Additional Raman experiments were carried out later on by other research groups,^585,586^ and more recently UV–vis absorption spectra were also used to study the confinement effect of Car molecules inside SWCNTs.^587^

Photoactive molecules have also been the subject of intense research in CNT endohedral functionalization, and in this respect, the majority of research has been conducted with two photoactive molecules, namely, oligothiophene and Pc. The first reports came from the preparation of erbium biphthalocyanine (HerPc_2_) confined into CNTs.^588^ Cao et al. used capillarity to fill MWCNTs with HerPc_2_ NWs of up to 10 μm in length, with potential applications in NIR photodetector devices. Schulte et al. described the successful encapsulation of cobalt phthalocyanine (CoPc) inside MWCNTs (please note that although it could be considered a hybrid organometallic compound, we have decided to include it in the organic family for classification purposes).^589,590^ The restriction of the assembly to one or two dimensions for this type of organic molecule is of particular interest because it can affect its electronic structure and, by extension, the optical, magnetic, and transport properties upon confinement. No internal order was observed in the stacking of CoPc within the CNT’s cavities. Recently, X-ray absorption near edge structure (XANES) and Resonant Inelastic X-ray Scattering (RIXS) were employed to probe the electronic structure changes in CoPc as a consequence of CNT encapsulation.^591^ The molecular orientation was found to be non-planar, and a change in molecular symmetry was observed upon encapsulation. DFT modeling can be used to confirm these changes in the electronic and geometrical structures of the guest molecules. The encapsulation of zinc phthalocyanine (ZnPc) in CNTs has also been reported recently,^592^ with behavior similar to that of the cobalt analog. HRTEM and Raman spectroscopy were used to probe the encapsulation efficacy and investigate the confinement effect. A high filling yield was observed in the HRTEM images, whereas a comparison of the Raman spectra for free ZnPc versus encapsulated ZnPc@SWCNTs revealed a rather free-like conformation of the organic molecule within the NT, similar to the bulk phase.

Oligothiophenes, such as 4T and 6T, are conjugated oligomers with semiconducting character. Their optical and electronic properties make them suitable for several applications, especially in optoelectronics. Loi et al.^593^ synthesized visible-light-emitting 6T@SWCNT peapods by the sublimation of 6T in the presence of 1.2–1.5 nm CNTs. The resulting nanohybrids were characterized by HRTEM and Raman spectroscopy, which suggested an interaction between both materials and an increase in the stability of the 6T molecules as a consequence of encapsulation. Similar observations were made by Kalbáč et al.,^594^ who also studied the doping effects using *in situ* Raman spectroelectrochemistry. One year later, Loi et al. observed visible PL with quantum yields of up to 30% for the hybrids obtained by encapsulating 4T, 5T, and 6T@SWCNTs.^595^ DFT calculations were used to investigate the electronic structure of the so-called “peapods” and the bonding mechanism between the tube and the encapsulated species (vdW interactions). Improvements in the encapsulation of these oligomers were achieved by adopting a synthesis strategy alternative to the sublimation technique. By using low *T* nanoextraction under CO_2_ supercritical conditions,^581^ the formation of insoluble oligothiophenes on the exterior of the CNTs was avoided, while similar filling ratios were observed using this approach.

Sauvajol et al. also observed evidence of charge transfer between CNTs and an encapsulated derivative of the conjugated oligomer 4T. TEM was used to study the morphology of the hybrid 4T@SWCNTs, while information about the charge transfer effect was obtained through Raman and UV–visible absorption spectroscopies.^596^ The maximum in the absorption spectra shifted from 400 to 403 nm after encapsulation, a behavior that has already been observed in similar filled compounds. Modifications in the Raman spectra due to encapsulation were also observed, suggesting a certain degree of interaction between the walls of the CNTs and the organic oligomer. Spatially Resolved Electron Energy Loss Spectroscopy (SR-EELS) was used to confirm the presence of S within the CNTs^597^ and the Infrared (IR) spectra of 4T@SWCNTs suggested a significant charge transfer.

A PL quantum yield of 15% was reported by Iijima et al. for coronene encapsulated in SWCNTs.^598^ The 1D molecular packing of the π-conjugated organic molecule when confined inside the CNT differs from the 3D packing observed in the solid state, which confers unique electronic and spectroscopic properties to the encapsulated material. Energy transfer from coronene molecules to the host CNT structure was suggested when comparing the quantum yield with that of coronene alone (23%). The same research group encapsulated C_60_ molecules with a chromophore retinal attached to SWCNTs and obtained atomically resolved images of individual structural isomers of the chromophore.^599^ The cis/trans isomerization process, which is undergone by the retinal molecules when absorbing light, is responsible for triggering the biological activity of rhodopsin (i.e., the initial step in the vision process); therefore direct observation of the structural isomers involved in the process is important for understanding the activity of these molecules *in vitro* and *in vivo*. Finally, HRTEM images of the individual molecules within the CNT cavities allowed the authors to study the conformational changes and the isomerization process.

The absorption spectrum of semiconducting CNTs limits the optoelectronic properties of these materials. To improve these properties, one option is to modify the diameter of the NT; however, this process is difficult to control. Photosensitization by dye encapsulation may be an alternative method to broaden the light absorption spectrum. In this line, Kataura et al. successfully filled squarylium dye inside SWCNTs.^600^ XRD revealed an off-center position for the dye molecules inside the CNTs, while polarization–resolved optical absorption indicated an alignment of the molecules parallel to the NT axis. The encapsulated dye effectively exhibited photosensitizing behavior, with energy transfer to the CNT. More recently, the synthesis of quaterrylene@SWCNTs, achieved by annealing a mixture of semiconducting CNTs and perylene powder under vacuum, was reported to produce excitation energy transfer between the CNTs and the organic molecules.^601^

#### 7.3.2 Drugs and Biomolecules

After the successful filling of CNTs with a variety of species and due to the interest of the scientific community in the use of CNTs for biomedical applications, it was not long before the filling of CNTs with biomolecules was achieved. To date, a wide array of bioactive materials, including drugs, proteins, nucleic acids, and DNA/RNA, have been successfully incorporated into the hollow cavity of CNTs. Whereas external functionalization is mainly directed toward the modification of the CNT wall to make it biocompatible, the encapsulation of molecules of biological interest into the CNT’s cavities is more related to the protection of the filling species from the external environment under biological conditions. The CNT shell is a protective mono-, bi- or multilayered 1D structure that can be used for the transport of bioactive molecules to the desired location and their protection from the biological milieu. Green et al. reported on the encapsulation of bioactive molecules in CNTs in 1995.^602^ They used HRTEM to study the morphologies of some small proteins, such as cytochrome c3 and β-lactamase I, immobilized inside CNTs, and other organic molecules potentially useful in the biomedical field.^603^ In a later report,^604^ they carried out a more detailed electron microscopy characterization of these proteins along with the stability and catalytic activity of the β-lactamase I protein. It was found that in the case of the proteins, their catalytic activity was retained, and no drastic conformational changes occurred as a consequence of the CNT confinement. The internal surface of the CNTs showed a strong interaction with the enzymes, indicating a favored immobilization process. Several other groups have reported the encapsulation of proteins within CNTs. For example, Kang et al. have reported a series of studies on the encapsulation of proteins in CNTs. Molecular simulations revealed the spontaneous encapsulation of metallothionein SmtA within CNTs with a suitable tube diameter,^605^ where vdW interactions between the protein and CNT played a dominant role in the process. The protein α-helix structure was almost unaffected by the encapsulation process, whereas a conformational change in the protein with a deformation of the protein β-sheets was observed after encapsulation to maximize CNT-protein interactions. MD simulations have been used to understand protein adsorption within CNTs^114^ and to predict the diameter selectivity of proteins in the encapsulation process.^606^ Conformational changes in the protein structure were expected to maximize its interaction with the tubular host structure, which was dependent on the protein size and the CNT diameter. For a given protein, an optimal CNT size was found that maximized the protein-CNT interactions and therefore provided the most effective encapsulation.

DNA and siRNA are other types of molecules that can be used for biotechnological applications combined with different types of nanomaterials, including CNTs.^607,608^ Gao et al. demonstrated the possibility of DNA encapsulation within the hollow cavity of CNTs.^609^ They used simulations to study the spontaneous insertion and confinement of single-stranded DNA composed of eight adenine bases with an uncapped armchair (10, 10) CNTs (length: 2.95 nm; diameter: 1.36 nm) through a combination of hydrophobic and vdW forces. As mentioned before, the CNT dimension play a determinant role, and if a certain critical value is exceeded (especially for the tube diameter), encapsulation is possible through a fast dynamic interaction process. Thereafter, a variety of examples encapsulating DNA molecules into CNTs by thermal treatment^44^ or electrophoresis^610,611^ have been reported. Alshehri et al.^612^ applied a mathematical model to investigate the vdW interaction energy between double-stranded DNA and different armchair tubes. Their results showed that the optimal radius of a CNT, for successfully encapsulating double helix DNA is 12.8 Å. The preferred tube conformation was (19,19), similar to the (20,20) CNT conformation previously predicted by Xue et al.^109^ Kamiya used DFT calculations to investigate the energy and electronic structure of single-stranded DNA encapsulated in SWCNTs,^613^ whereas Wu et al. studied the DNA ejection mechanism from filled CNTs using MD simulations in two different reports.^614,615^ Finally, Mogurampelly et al.^616^ reported the results of the MD simulations for the encapsulation of small interfering RNA (siRNA) within CNTs of various diameters and chiralities.

The encapsulation of therapeutic drugs into the hollow cavity of CNTs represents another field of high interest in pharmaceutical applications. They provide excellent shielding when it is necessary to protect the active compound from the biological environment. Some of these factors include instability and degradation, toxicity, light sensitivity, and interactions with other molecules. Platinum­(II)-based anticancer agents, such as cisplatin (cis-diamminedichloroplatinum, cis-[Pt­(NH_3_)_2_Cl_2_], CDDP), carboplatin (CB), and oxaliplatin (L–OHP), are clear examples. Their undesired side effects and drawbacks have encouraged the scientific community to pursue for novel technologies capable of localized and more effective delivery of these metallodrugs.^617^ Thus, several groups have reported the successful encapsulation of CDDP into CNTs.^618^ Hilder et al.^619^ modeled the interaction of CDDP molecules with CNTs. They found that the minimum CNT radius for CDDP to be successfully encapsulated was 4.785 Å, which corresponds to a (9, 5) CNT, while the maximum uptake was observed for a CNT radius of 5.3 Å, corresponding to a (11,4) CNT. Tripisciano et al.^620^ studied the incorporation of CDDP into SWCNTs by Raman, IR and HRTEM (Figure 22). The EDX spectrum of the CDDP-functionalized annealed SWCNTs confirmed the presence of Pt in the sample (Figure 22 c)). The amount of CDDP encapsulated into the CNTs was estimated using Differential Thermal analysis (DTA), with the results indicating a loading of 21 μg CDDP per 100 μg CNT. The release of the drug under physiological conditions was also evaluated, with 68% of the drug discharged from the CNT in a period between 48 and 72 h. The filling of MWCNTs with CDDP through capillary forces was also investigated by the same group.^621^ In this case, the release was found to be faster (12–48 h) and almost complete (95%), as confirmed by Inductively Coupled Plasma (ICP) analysis. Spherical CDDP clusters located in the MWCNT hollow core were observed by HRTEM, while TGA was used to calculate the amount of CDDP encapsulated, which was found to be lower than that for SWCNTs (13.6 μg CDDP per 100 μg SWCNT). This could be attributed to the larger number of carbon atoms in each individual MWCNT compared to a SWCNT. More recently, Li et al.^622^ also reported the successful encapsulation of CDDP into “capped” and “uncapped” MWCNTs using nanoextraction. A higher drug loading into the CNTs was achieved in comparison with previous reports, with a maximum loading capacity of 62.1 μg CDDP per 100 μg CNT in 40 h.

**Figure 22:**
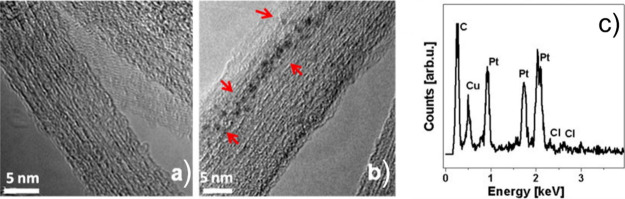
SWCNTs filled with CDDP. TEM images of a) annealed SWCNTs (reference), and b) CDDP-functionalized annealed SWCNTs. In c), the EDX spectrum of the CDDP-functionalized annealed SWCNTs is presented. Signals from Cu (from TEM grid), C (from CNTs) and Pt (from CDDP) are detected.^620^ Reproduced with permission from ref 620. Copyright 2013, Elsevier.

CB, another therapeutic agent that is more water-soluble and has fewer adverse effects than CDDP, has also been used as a filling agent for CNTs. Mönch et al. demonstrated the filling of MWCNTs with CB.^623^ They initially synthesized MWCNTs filled with Fe for biomedical applications and then filled them *ex situ* with CB using a wet chemical technique. HRTEM images demonstrated the successful encapsulation of the drug in the hollow cavities of the CNTs. In a similar report,^624^ the role of *T* on the loading of CB into MWCNTs was investigated, with a maximum loading of 30 wt % at 90 °C. TEM, EDX, XPS, and ICP were used to determine the characteristics of the loaded CNTs, whose structure was retained after encapsulation of the chemotherapeutic drug.^625^ A loading yield of 0.20 mg Pt/mg material was achieved for MWCNTs, with 68% of the drug being released from their interior over a period of 14 days, which demonstrates the capability of filled CNTs to act as molecular containers and nanocarriers. A maximum loading of 43.6% into the internal cavities of PEGylated MWCNTs was recently achieved for L–OHP,^626^ another platinum-based anticancer drug that induces DNA cross-linking and is currently used for colon, ovarian, and lung cancer.

Drugs that are not based on Pt complexes have also been successfully incorporated into the cavities of CNTs. Irinotecan is an anticancer drug that prevents DNA unwinding by inhibiting the enzyme Topoisomerase I. Tripisciano et al.^627^ confined this drug into MWCNTs. The resulting filled CNTs were analyzed by TGA, Raman spectroscopy and IR spectroscopy to determine the successful filling with the drug, its integrity during the filling procedure, and the amount of drug agent entrapped within the cavity. The maximum loading efficiency was approximately 32 wt % was achieved, with an appreciable and fast drug release from the CNTs at pH = 6. Another example of the usefulness of CNTs as nanocarriers is the formation of a “carbon nanobottle”. This can be achieved, for instance, by using fullerenes as “corks” taking advantage of their affinity with the inner cavities of CNTs and the relative spontaneity of their encapsulation.^628,629^ Using this approach, Ren et al. filled SWCNTs with the antitumor agent hexamethylmelamine and subsequently sealed the open tips of the host to form nanocapsules with potential applications in drug delivery.^629^ A partial release of the encapsulated molecules was observed by changing the solvent. Another strategy that has been developed consists in the use of functionalized fullerenes as corks. This allows the triggered release of the encapsulated payload upon decreasing the pH of the solution.^165^ The capacity of CNTs to load gemcitabine (GEM),^125^ which is usually employed as an anticancer agent for lung and pancreatic cancers, has been theoretically studied, as well as other drugs such as lamivudine,^630^ paclitaxel, doxorubicin (DOX)^631^ and Olutasidenib.^632^ As mentioned above, aspects such as length, radius, or even the chirality of the CNTs may play a major role in the encapsulation of foreign organic bioactive molecules within their interior. In the case of the drug carbazochrome, for instance, studies revealed that 8.8 Å diameter SWCNTs with chiral angles of 8° not only favor the encapsulation speed, but also show a narrower interaction between the drug and the CNT.^633^

CNTs can act as reservoirs for the controlled release of NO in biological environments. The presence of this diatomic molecule promotes the endothelial cell migration and proliferation and prevents bacterial infection. Therefore, incorporating it into functional biomaterials may improve its therapeutic effects. In a recent report, Kabirian et al. incorporated S-nitroso-N-acetyl-d-penicillamine (SNAP), a biocompatible NO donor, into functionalized MWCNTs. After loading SNAP, MWCNTs were coated with the polymeric matrix polyethylene glycol-poly-ε-caprolactone (PEG–PCL) and incorporated into 3D printed biodegradable vascular grafts. A significant improvement in the physiological NO release was observed, along with a reduction of the initial burst, previously reported for free SNAP/PEG–PCL.^634^

#### 7.3.3 Polymers and Related Monomeric Molecules

Polymer chemistry has played a pivotal role in the development of CNTs and their applications. The exohedral modification of CNTs with polymers is one of the main strategies for their dispersion. Most research on the combination of polymers and CNTs has focused on the use of the latter to enhance the properties of polymeric materials. Polymers are also widely employed to increase the biocompatibility of CNTs when externally functionalized. When it comes to filling, the majority of reports published so far have dealt with the encapsulation of the monomeric unit of the polymer alongside an initiator, so the polymerization reaction takes place inside the CNTs after encapsulation. Therefore, the final reaction product can be controlled using this approach. Examples in this area include styrene with/without benzoyl peroxide as an initiator to form polystyrene (PS),^635,636^ pyrrole (Py) to form polypyrrole (PPy), N-vinyl carbazole with azobis­(isobutyronitrile) (AIBN) as an initiator to give poly­(*N*-vinyl carbazole),^637^ and aniline or acrylonitrile to form polyaniline (PANi).^638^ Steinmetz et al. took advantage of scCO_2_ to promote the formation of conducting polymers within the inner cavities of CNTs, leading to new materials with potential applications as sensors or electrodes. The authors demonstrated the versatility of this technique by impregnating of CNTs with different monomers, namely, N-vinyl carbazole, Py, or acetylene. In the presence of AIBN and the Ziegler–Natta catalyst and under supercritical conditions, poly­(*N*-vinyl carbazole) and polyacetylene can be formed inside CNTs. In the case of the preparation of PPy, the synthesis of PPy@CNTs was conducted by oxidation in an FeCl_3_ solution.^639,640^

The successful direct encapsulation of polymers into CNTs has rarely been demonstrated. Yarin et al.^641^ reported in 2007 the encapsulation of low-molecular weight polymers like poly­(ethylene oxide) (PEO) and poly-*ε*-caprolactone (PCL). Dilute solutions of relatively low molecular weight polymers (10–100 kDa) were found to diffuse into as-grown MWCNTs of 50–100 nm diameters. TEM images confirmed the successful encapsulation and showed different conformations of the polymer deposits inside the CNTs (peapod, foam, and dispersed bubbles). When the molecular weight of the polymer was increased, the number of encapsulated species decreased up to the point where no filling occurred. Recently, the successful encapsulation of polyarylene ether nitrile inside MWCNTs was reported, and its crystallization behavior was studied by You et al. Crystalline polymers, with enhanced thermal, chemical, and mechanical properties, compared to amorphous polymers, can be used in a wider range of processing conditions, thus expanding the range of applications.^642^ Several studies have been dedicated to simulations of CNTs driven polymer encapsulation. Park et al. studied the electronic structure of a polyacetylene chain interacting with CNTs in different conformations.^643^ DFT calculations revealed a preference for CNTs wider than the (5,5) configuration, with weak attractive interactions between the polymer and CNT surface. More recently, Li et al. investigated the role of chirality, *T*, and radius on the interaction energy of polyethylene (PE) encapsulated in SWCNTs using MM and MD.^116^ The results suggested an influence of all these parameters on the relatively quick filling process, which is favored by the attractive interaction between the CNT and the polymer. Among chiral CNTs, the armchair configuration presents the strongest interaction. A comparison of the filling processes for pristine and modified CNTs was reported by Ling et al.^644^ They studied, using MD, the influence of the polarity of the polymer chain (PE, poly­(vinyl alcohol) (PVA), and poly­(vinyl chloride) (PVC)) on the encapsulation in SWCNTs. Their results revealed that highly polar polymers could not be filled into CNTs.

### 7.4 Liquids

#### 7.4.1 Water

Water usually plays an active role in most chemical reactions, regardless of its expected role as a solvent, vehicle, or reactant. Its confinement at the nanoscale induces modifications not only in the entropy but also in the enthalpy of the system.^645^ The encapsulation of water within local environments is of great interest in biology, geology, and materials science because it can resemble the structure of biological membrane channels. H_2_O-filled CNTs can be used in nanofluidic devices, sensing or separation, desalination, and purification. Despite the hydrophobic nature of CNTs’ channels, it has already been demonstrated that water can be encapsulated in their hollow interior. The surface tension of pure H_2_O is below the 200 mN m^–1^ limit, which makes it possible to fill CNTs by capillary forces.^40^ As in the case of other species that have been successfully filled in CNTs, the confinement of water within the CNT is expected to bring novel and unusual properties, different from those observed in the bulk phase (e.g., phase transitions). The size of the CNT also plays a pivotal role in this respect, considering that the interactions between the pore wall and filling molecules increase as their dimensions approach one another. Because these interactions can be modulated, CNTs can act as suitable models for studying the confinement of liquids, especially water, in nm-sized channels.

Different studies exploring the confinement of water within the cavities of CNTs have been reported.^110,646,647^ Hummer et al.^110^ used MD simulations to probe the continuous filling of non-polar CNT’s cavities with water molecules in a 1D chain-ordered fashion. As a consequence of the curvature-induced static dipole moment, which affects the orientation of the water molecules, a heterogeneous degree of filling along the CNT is predicted.^648^ Since the single-filled chains formed after filling water molecules into the CNTs are magnetically oriented along the tube axis, the application of a homogeneous electric field might shift the equilibrium of the process toward the filled state.^649^ Koga et al.^650^ used computer simulations to study the behavior of water confined inside CNTs, with the formation of novel ice phases upon cooling and application of axial pressures from 50 to 500 MPa. Under these conditions, a continuous transformation of liquid-like water into solid-like water was observed. The existence of a solid–liquid critical point, which is not present in the bulk state, would allow the continuous and direct transformation of liquid matter into a solid.

Striolo et al. published a series of manuscripts^100,651–654^ explaining the water confinement properties and diffusion mechanisms inside CNTs. For example, MD simulations were used to prove that in an infinitely long CNT with a diameter of 1.08 nm, water molecules diffuse through a fast ballistic motion mechanism within the CNT’s cavity.^653^ Long-lasting H-bonds are responsible for this behavior, which are coordinated and not dependent on the filling degree. The transport properties of confined water in heterogeneous CNTs were studied^651^ to understand the effect of hydrophilicity imposed by these heterogeneous sites on the surface of the CNTs. It was found that the diffusion inside the CNTs is faster when the number of confined water molecules is increased, up to a maximum (108 molecules of water within (8,8) SWCNTs decorated with carbonyl groups), where the self-diffusion coefficient starts to decrease. This effect was attributed not only to preferential interactions between water molecules and oxygenated sites but also to the small CNT diameter. Despite further theoretical studies on the behavior of water^655–658^ and their transporting processes^659^ in the interior of CNTs were thoroughly investigated, it was not until 2004 that Naguib et al. reported, for the first time, the *in situ* observation of water inside CNTs.^660^ TEM micrographs along with EELS and EDS indicated the presence of water in closed MWCNTs with 2–5 nm diameter channels treated at different *T* and pressures, with water molecules entering the CNTs through wall defects. Byl et al.^661^ used vibrational spectroscopy to study the confinement of water molecules within CNTs and the effect of their interactions through unusual H bonding. A stacked ring structure with intra- and inter-ring H bonds was observed, with the latter being relatively weak, and giving rise to a distinct OH stretching mode at 3507 cm^–1^, which was directly related to the confinement effect. This observation of different OH configurations for water molecules when confined in the CNT’s cavity was confirmed by Paineau et al.,^662^ who explored MD simulations to determine the vibrational spectra of H_2_O molecules inside narrow CNTs. Wenseleers et al. took advantage of the variations in the vibrational modes of H_2_O@CNTs to propose a quantitative approach to assess the amount of open-ended CNTs (with a known chirality) present in a determined sample.^663^ For this purpose, the authors dispersed both untreated and open-ended AD SWCNTs in a solution of sodium deoxycholate (surfactant) in D_2_O, and then monitored the variations in the RBM peaks by means of Raman spectroscopy, Figure 23.^663,664^

**Figure 23:**
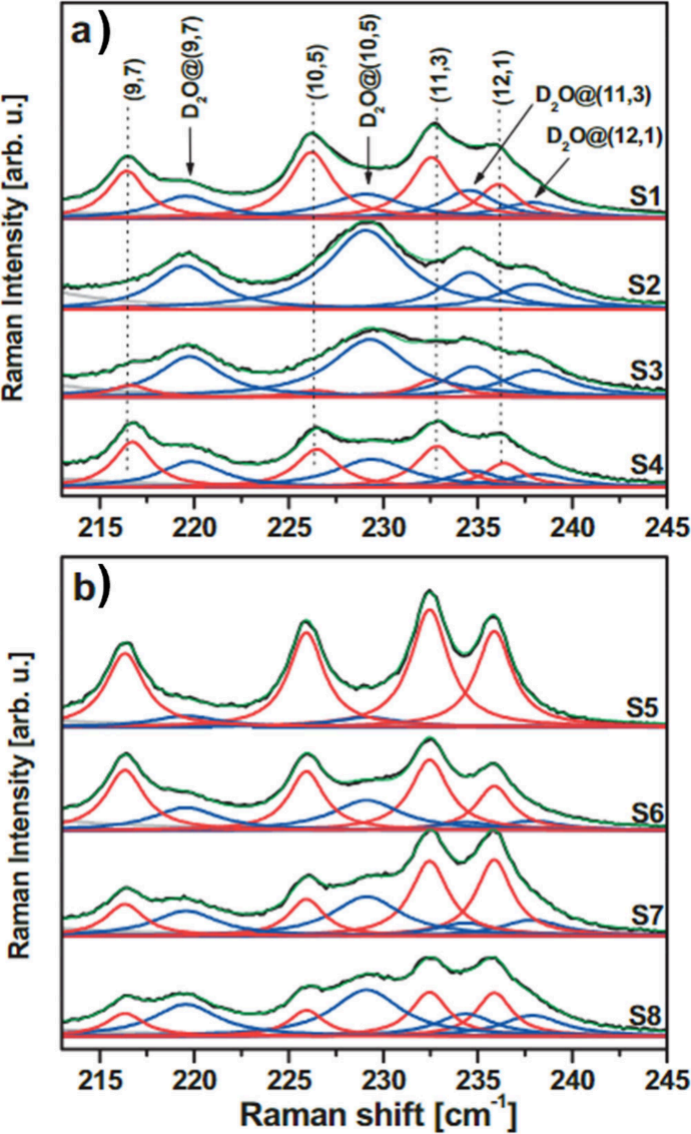
Raman spectroscopy of H_2_O@SWCNTs. Resonant Raman spectra at 785 nm (excitation wavelength) of a) chemically (S1–S4) and b) ultrasonically (S5–S8) treated SWCNTs. In all cases, an increase of the signal denoting the successful filling with water (blue line, shifted with respect to the red fitted spectrum) suggests the increase of the number of open-ended CNTs.^663^ Reproduced with permission from ref 663. Copyright 2007, John Wiley and Sons.

Several experimental methods have been developed to probe the structure and dynamics of water at the nanoscale,^665^ including XRD, neutron diffraction (ND) and X-ray scattering (XRS), X-ray absorption spectroscopy (XAS), IR spectroscopy, TEM, nuclear magnetic resonance (NMR) and hyperspectral PL microscopy.^666^ The information provided by each of these techniques has been crucial for understanding the behavior of water molecules inside the CNTs, with pronounced differences with respect to the bulk state. The confinement effect, hydrophobicity of the CNT channels, and the strong intermolecular interactions between water molecules account for these,^64^ but the nature of the interactions is strongly dependent on the CNT structure (chirality, length, diameter, etc.). The process of filling water molecules within SWCNTs was recently evaluated using XRS. *In situ* XRS measurements were performed while exposing empty CNTs to a saturated atmosphere of water vapor. Aspects such as the powder density in the capillary played a role in the filling time. Simultaneously, the configuration of water molecules within the CNTs strongly depends on their diameter and degree of filling. Thus, for 14 Å CNTs, a layered structure with the highest density of water at the center of the host was detected. During the process, a homogeneous distribution of water molecules was observed for fillings of approximately 5%, whereas a maximum of ca. 15.5% in mass was achieved using this approach.^667^

The singular mechanical^668^ and optoelectronic^669^ properties of SWCNTs can be influenced by confining water molecules within their interiors. Cambré et al. identified significant differences in both the PL and PLE spectra of empty and water-filled sorted SWCNTs. The main observations include red shifts of the spectra after confining water molecules within the CNTs,^666^ along with the formation of wider emission lines owing to the increase in individual extrinsic perturbation. In contrast, empty CNTs presented narrower spectra with well-resolved RBM phonon sidebands for small-diameter tubes. Confined water molecules also exhibit variations in their optical behavior compared to their bulk counterparts. By carrying out *T*-dependent (4.2 K up to RT) PL experiments, the authors observed a shift of the H_2_O@SWCNTs at approximately 150 K, which was attributed to a change in the dipole orientations of the encapsulated water molecules. The experimental results were supported by MD calculations that predicted qualitative changes in the orientational order within the measured *T* range.^670^ Due to the marked differences encountered in the emission spectra, PL measurements emerged as suitable characterization tools for the assessment of both water-filled and empty CNTs with different chiralities. More recently, Li et al. performed ML-MD simulations to evaluate the variations in the thermal transport of CNTs induced by filling them with water molecules.^647^ After encapsulation, the thermal conductivities of the hybrids were reduced by up to 28%, which was closely dependent on the diameter of the CNT, reaching values of 432.5 W m^–1^ K^–1^ for 2148 Å tubes.

#### 7.4.2 Organic Solvents

The encapsulation of organic solvents within CNT’s cavities has also been attempted, although the number of reports is still scarce. Here, we focus on the filling of CNTs with pure solvent molecules without the presence of a second filling agent. For example, Chaban studied the filling of CNTs with acetonitrile (CH_3_CN) because of its interest in supercapacitors.^671^ MD simulations carried out in narrow armchair CNTs, ranging from (5, 5) to (11, 11) with 10 nm lengths, showed that they can be filled with CH_3_CN in less than 100 ps, with the filling rate weakly dependent on the CNT diameter. Few reports have also dealt with the MD of the encapsulation of aromatic solvent molecules, such as benzene (C_6_H_6_) and its derivative cyclohexane (C_6_H_12_), in the hollow cavities of CNTs.^672–674^ Several preferred conformations were observed as a function of the CNT diameter, from a single-file distribution for smaller (7, 7) CNTs to a double-shell conformation in (12, 12) CNTs. The dynamics of the solvent molecules are much slower when confined within CNTs than in the bulk state. The conformation of methanol (CH_3_OH) inside CNTs was also investigated using simulations.^675^ Depending on the CNT diameter, bulk, biwire or monowire modes were observed. This behavior is different from that of water molecules encapsulated in CNTs, probably because of the coexistence of hydrophobic and hydrophilic groups within the methanol molecule.

#### 7.4.3 Ionic Liquids (ILs)

ILs, ionic salt-like materials that exist in the liquid state below 100 °C, are an alternative to classic solvents. They are considered “green” organic solvents, with remarkable properties such as high polarity and ionic conductivity, negligible vapor pressure, and good thermal stability. These properties make ILs the ideal solvents for carrying out chemical reactions in confined spaces, such as CNT’s cavities. The first report of the successful encapsulation of an IL appeared in 2007 by Wu et al.^676^ The confinement effect of MWCNTs in the IL [bmim]­[PF_6_] induced the formation of a polymorphous crystal with a high melting point (200 °C compared to 6 °C for the unconfined IL). Samples were characterized using of HRTEM, XRD, and DSC to confirm the formation of the crystals within the CNT’s cavities. Two years later, Shinohara et al. reported the behavior of the IL [Me_3_NC_2_H_4_OH]­[ZnCl_3_] inside SWCNTs.^677^ The thermal decomposition of the IL in the confined state was higher than that in the bulk state, and the encapsulated material exhibited different morphologies (single-chain, double helix, zigzag tube, random) as a function of the CNT diameter. The electronic properties of the CNTs can also be modulated from *p*-type to *n*-type as a function of the filling ratio.

### 7.5 Gases

The storage of gases by nanostructured carbons is an active field of research, and many different types of carbon materials have been reported for their exceptional properties (graphene, mesoporous carbons, etc.).^678^ The first report on the encapsulation of gas molecules within CNTs appeared in 1997 from Dillon et al.,^679^ who demonstrated that H_2_ molecules could condense into CNT’s cavities. Temperature-programmed desorption (TPD) spectroscopy was used to probe the adsorption of H_2_ molecules via a physisorption mechanism, with an estimated 5–10 wt % maximum storage capacity. There is a strong interest in developing clean energy sources and processes for their production. H_2_ has been the most studied gas for CNT gas storage; however, other gases have also been reported, including greenhouse gases (GHG) such as CO_2_,^680–682^ CH_4_ ^683–685^ and CF_4_;^686^ inert gases like N_2_,^687^ Ar,^688^ and Kr^689^ or hydrocarbons such as C_2_H_2_.^690,691^ Although many early reports claimed very promising results and high gas adsorption capacities, in many cases no distinction was made between the exohedrally and the endohedrally adsorbed molecules. The results suggest that the adsorption of gas molecules in CNTs can occur in three different locations: CNT’s cavities and their external surfaces, and interstitial spaces between CNTs in CNT bundles. The purity of CNTs was not considered in the 90s, when the first reports were published; therefore, a significant contribution of these impurities to the total gas storage capacity could be expected in some of the studies. The presence of defects on the external surface of the CNTs also plays an important role in the nature of the host-gas interaction. Therefore, this will definitely contribute to the overall storage capacity of these materials.^692,693^

## 8 Applications of Encapsulated Materials in CNTs

The ability of CNTs to be filled with small molecules, compounds, and NPs has paved the way for the appearance of new exotic properties that can be explored in many different fields of application.^694,695^ Indeed, fundamental studies using these carbon-based nanostructured hybrids have been extensively performed to explore new electronic,^696^ magnetic,^696^ optical^696,697^ and mechanical^695^ properties. The appearance of new features resulting from the QC of the filling materials within the internal cavity of CNTs and/or the establishment of strong interactions between the filling material and the internal walls of CNTs can reveal the potential for the development of new devices in different areas of application. Next, we present a systematic discussion of the most relevant achievements of filled CNTs in biomedicine, energy storage, nanoreactors, sensors, and catalysis and their respective technological impacts.

### 8.1 Biomedical

CNTs recently celebrated their quarter-century anniversary (CNT25, Tokyo, Japan),^698^ and a relevant part of their history has been marked by extensive research on the development of new biological and biomedical applications.^699^ Indeed, during the past few years, efforts have intensified to fully explore the 1D structural properties and chemical versatility of CNTs^700^ for biomedical purposes in the areas of diagnosis and therapy.^701^ However, many concerns related to CNTs’ asbestos-like structure, which can promote bioaccumulation and consequently increase the carcinogenic risks, have limited CNTs progress in nanomedicine, comparatively to other application fields.^702^ Nowadays, it is well-known that CNTs’ biotoxicity is mainly governed by the presence of impurities, structural properties (diameter, length, and aggregates), and biointerface.^703^ Several approaches are now available to modulate the interfacial chemistry and structural features of CNTs, in order to accurately control their behavior according to the required biological specifications.^704^ Indeed, relevant improvements for the biological use of CNTs have been clearly achieved by developing strategies for purification and shortening their lengths.^705–707^ Additionally, it was found that tailored surface functionalization of CNTs can favor cellular internalization and intracellular circulation^708–710^ and improve the *in vivo* biocompatibility, biodistribution, and bioaccumulation.^711–713^ In fact, the accurate control of CNTs interface combined with short lengths can exhibit long circulation times, low uptake by the reticuloendothelial system, and high tumor accumulation.^699^ Recently, the first *in vivo* trial in non-human primates regarding the biocompatibility of short amino-functionalized CNTs was reported. This study provides extensive insights into the *in vivo* biodistribution, uptake, and processing of ^86^Y-functionalized CNTs ([^86^Y]­f-CNT) using positron emission tomography (PET). The collected data revealed almost complete elimination of the injected activity (>99.8%) from the primate within 3 days. This result demystifies the typical label associated with CNTs for permanent *in vivo* bioaccumulation, showing that properly treated CNTs can have a positive impact on the development of new biomedical applications.

#### 8.1.1 Cancer Therapy

CNTs have been widely explored over the years for the development of biomedical applications, with particular emphasis on oncology.^714^ Great progress has been made using CNTs as multifunctional nanomaterials for detecting cancerous cells and delivering drugs or carrying other therapeutic biomolecules by taking advantage of their internal cavities. The most relevant developments in the endohedral functionalization of CNTs for cancer therapy and bioimaging are summarized in Table 8.

**Table 8:** Summary of Filled CNTs for Biomedical Imaging and Therapy

			Theranostic		
CNTs	Endohedral functionalization	Exohedral functionalization	Therapy	Bioimaging	ref.
**SWCNTs**	Ultrashort SWCNTs (<50 nm) loaded with I_2_.	Covalent functionalization with Serinol amide.	-	• Intracellular molecular imaging by computed tomography (CT) X-ray.	^715^
	SWCNT loaded with CDDP.	Non-covalent functionalization with PEG-5000.	• Drug delivery – *In vitro* cytotoxicity in prostate cancer cells PC3 and DU145.	-	^620^
		Non-covalent functionalization with Pluronic surfactant.	• *In vivo* biodistribution in MDA-MB-231 tumor-bearing mice.	-	^716^
			• *In vivo* therapy in MCF-7 xenografts established in SCID/Beige mice and in MDA-MB-231 human breast cancer patient-derived xenograft (PDX) model.		
		Non-covalent functionalization with Pluronic surfactant.	• Drug delivery – *In vitro* cytotoxicity in MCF-7 and MDA-MB-231 breast cancer cell lines.	-	^618^
		Non-covalent functionalization with Pluronic surfactant.	• Drug delivery remotely triggered by radiofrequency fields – *In vitro* cytotoxicity in human liver cancer cells Hep3B or HepG2.	-	^717^
	SWCNTs filled with Radionuclide Na^125^I.	Covalent functionalization with biantennary carbohydrate (N-acetylglucosamine (GlcNAc)).	-	• *In vivo* tracking in a Balb/C mouse using single-photon emission computed tomography (SPECT).	^718^
	SWCNTs loaded with GdNTs.	Non-covalent functionalization with Pluronic surfactant.	-	• *In vitro* cytotoxicity with J774A.1 mouse macrophages cell line.	^719^
				• *In vivo* biodistribution in a female C57BL/6 mouse by MRI.	
	Ultrashort SWCNTs loaded with BiOCl/Bi_2_O_3_.	Non-covalent functionalization with Pluronic surfactant.	-	• Intracellular labeling of pig MSCs by Computed Tomography (CT).	^463^
	Ultrashort SWCNTs loaded with Gd^3+^ and ^64^Cu^2+^ ions.	-	-	• *In vivo* dual PET/MRI.	^720^
	SWCNTs loaded with Zr^4+^ (ends sealed with fullerenes C_70_).	Covalent functionalization with Cyclo(-RGDfK) peptide or Bombesin.	• *In vitro* cell internalization into human prostate cancer cells PC-3 or CHO cells.	-	^721^
	SWCNTs filled with PbO, BaI_2_ and Kr.	Covalent surface functionalization with different peptides for subcellular trafficking.	-	• *In vitro* bioimaging of HeLa cells by X-ray fluorescence (XRF) mapping.	^689^
	SWCNTs filled with SmCl_3_ and LuCl_3_.	Covalent functionalization with triethylene glycol (TEG) and monoclonal antibody mAb.	-	• *In vitro* cell internalization in EGFR positive cancer cells U87-EGFR+ and CHO cells.	^387^
				• Development of radioisotopes for SPECT.	
	SWCNTs filled with SmCl_3_	Covalent functionalization with TEG and monoclonal antibody mAb.	-	• Immunological *in vitro* profiles of in murine (RAW 264.7 macrophages) and human cells (peripheral blood mononuclear cells, PBMCs).	^722^
				• Immunological impact after *in vivo* administration in C57Bl/6 mice.	
				• Development of radioisotopes for SPECT.	
	SWCNTs loaded with ^64^Cu (ends sealed with fullerenes C_70_).	Noncovalent functionalization with β-d-glucan.	-	• *In vitro* biocompatibility and stability studies with PC-3, Chinese hamster ovary (CHO) and healthy human primary skin (FEK4) by multiphoton fluorescence lifetime imaging with NIR excitation.	^723^
				• *In vivo* Bioimaging in healthy male Wistar rats by PET.	
	SWCNTs loaded with Hexamethylmelamine (HMM).	SWCNTs ends sealed with C_60_ (nanobottles).	• Drug delivery – proof of concept.		^629^
**DWCNTs**	DWCNTs loaded with Chloroquine.	Non-covalent functionalization with Polyethylenimine for plasmid DNA binding.	• Dual gene and drug delivery – *In vitro* biocompatibility tests with HeLa cells.	-	^724^
	DWCNT loaded with Indole (model molecule).	Covalent functionalization with EphB4-binding peptides.	• Drug delivery triggered by NIR irradiation – *In vitro* cytotoxicity of HeLa cells.	-	^725^
	DWCNTs loaded with HMM.	DWCNTs ends sealed with C_60_ (nanobottles).	• Drug delivery – proof of concept.		^629^
**MWCNTs**	MWCNTs filled with Fe.	Preincubation with cationic lipid formulation.	• *In vitro* biodistribution in human bladder cancer cell line EJ28.	-	^726^
		-	• *In vivo* biocompatibility using mouse model.	-	^623^
		-	• *In vivo* laser-induced thermotherapy (LITT) in orthotopic mouse model of human breast cancer.	• *In vivo* MRI imaging orthotopic mouse model of human breast cancer.	^727^
		Covalent functionalization with amine groups and mAb targeting ligand.	• *In vitro* cell biocompatibility with CHO and EAhy926 cell lines.	-	^728^
			• *In vitro* cytotoxicity in lung cancer cell line A451 cells (EGFR^+^) triggered by electromagnetic radiation inducing magnetic fluid hyperthermia.		
		Surface functionalization with Gd^3+^.	• Candidate for magnetic hyperthermia treatment.	• Candidate for MRI contrast agent.	^729^
		Non-covalent functionalization with 1,2-Distearoyl-*sn*-Glycero-3-Phosphoethanolamine-N-(PEG)2000 and targeting ligand FA.	-	• *In vivo* biodistribution and biotoxicity in mice.	^257^
				• *In vivo* bioimaging to mice bearing EMT6 tumor xenografts by thermoacoustic imaging (TAI) and MRI.	
	MWCNTs loaded with CB.	-	• Drug delivery – *In vitro* cytotoxicity in EJ28 cancer cells.	-	^624^
	MWCNTs loaded with Irinotecan.	-	• Drug delivery at different pH values (experimental).	-	^627^
	MWCNTs loaded with Dexamethasone.	Surface functionalization with PPy.	• Drug delivery by electrical stimulation – *In vitro* cytotoxicity in highly aggressively proliferating immortalized (HAPI) cells cultures.	-	^730^
	MWCNTs loaded with CDDP.	MWCNTs ends sealed with Au NPs functionalized with alkanethiols.	• Drug delivery triggered by pH – *In vitro* cytotoxicity in Human mammary gland adenocarcinoma epithelial cells (MCF-7).	-	^622^
		Covalent functionalization with 2,2′-(ethylenedioxy)diethylamine (TEG).	• *In vivo* biodistribution studies in a female BALB/c mice model.	-	^731^
	MWCNTs loaded with paclitaxel (Taxol) and C6-ceramide.	Non-covalent functionalization with amine-terminated PL–PEG.	• Drug delivery triggered by a.c magnetic field – *In vitro* cytotoxicity in pancreatic cancer cells (L3.6).	-	^732^
	MWCNTs filled with Fe_3_O_4_.	Surface decoration with transferrin (Trf), hybrid SiO_2_-coated quantum dots (HQDs) and anticancer drug DOX hydrochloride (DOX).	• *In vitro* cellular uptake and intracellular biodistribution in Hela cells.	• Cancer-targeted optical imaging (fluorescence).	^733^
			• Target Drug delivery – *In vitro* cytotoxicity in Trf negative HEK293 cells.		
			• Drug delivery triggered by magnetic field – *In vitro* cytotoxicity studies with Hela cells.		
	MWCNTs loaded with L–OHP.	Covalent functionalization with PEG 600.	• Drug delivery – *In vitro* cytotoxicity in human colon cancer cells HT29.	-	^626^
	MWCNTs loaded with GEM.	Covalent functionalization with FA followed by carboxylation, acylation and amidation.	• Drug delivery – *In vitro* cytotoxicity in human breast cancer cells MCF-7.	-	^734^
	MWCNT loaded with platinum(IV) prodrug (PtBz).	Covalent functionalization with TEG. Derivatization with mitochondrial-targeting fluorescent rhodamine-110 or non-targeting fluorescein.	• Drug delivery – *In vitro* cytotoxicity with A2780 ovarian carcinoma cells.	• Intracellular biodistribution in MCF-7 cells by fluorescence.	^735^
	MWCNT and carbon nanohorns loaded with ^6^LiI and EuCl_3_.	Covalent functionalization with amine groups.	LiNCT (^6^LiI) in rat osteosarcoma UMR-106 cells.	• Confocal imaging in HeLa cells (EuCl_3_).	^736^

The internal cavity of CNTs was first explored in cancer therapy for developing novel drug delivery systems. The ability of CNTs to encapsulate anticancer drugs allows researchers to overcome some limitations of “free” drugs *in vivo*, such as improving the formulation of poorly water-soluble drugs, allowing targeted delivery, enabling the codelivery of several drugs simultaneously, and controlling the drug delivery profile at the tumors with or without external stimuli. The potential of CNTs for the encapsulation and controlled release of different cancer therapeutic drugs was initially predicted by MD simulation studies.^699^ Some experimental parameters have been identified as crucial for the development of reliable simulations for the filling of CNTs with therapeutic payloads, such as the interactions between CNTs and drugs (vdW forces and electrostatic interactions) and the dimensions of CNTs, with particular relevance to diameter. MD simulation studies have already been performed to investigate the propensities of CNTs to self-insert peptides,^124,737,738^ RNA/DNA^44,609,739^ and small drug molecules (for instance, CDDP,^99,123,619^ GEM,^125,740^ DOX,^631,741^ Temozolomide,^742^ Paclitaxel^631^ and Topotecan^743^).

CDDP is one of the most studied cancer drugs for developing of CNT-based drug delivery systems (Table 8). The cytotoxicity of SWCNTs loaded with CDDP has already been studied for non-stimulated *in vitro* drug delivery with prostate cancer cells PC3 and DU145,^620^ and remotely *in vitro* triggered drug delivery by radiofrequency with human liver cancer cells Hep3B or HepG2.^717^ Recently, *in vivo* studies with CDDP-filled SWCNTs were conducted, and their biodistribution in MDA-MB-231 tumor-bearing mice was assessed. Moreover, their therapeutic efficiency was evaluated in mice bearing human breast cancer xenografts.^716^ The authors reported that CDDP@SWCNTs demonstrated higher efficacy in suppressing tumor growth than free CDDP in both xenograft models. Drug-filled CNTs enabled safe transport and accumulation at the therapeutic site, providing gradual delivery of therapeutic doses and consequently minimizing the risks of side effects.

MWCNTs have also been explored for the controlled release of CDDP to improve its therapeutic efficiency. Li et al. developed CDDP-loaded MWCNTs with a drug delivery profile triggered by the pH of the medium.^622^ Indeed, an enhanced *in vitro* cytotoxicity of CDDP in human mammary gland adenocarcinoma epithelial cells (MCF-7) was reported by the controlled release of CDDP from the inner cavity of MWCNTs. Additionally, the *in vivo* biodistribution of CDDP-filled MWCNTs in a female BALB/c mouse model was assessed (Figure 24).^731^ Preclinical studies confirmed that filled MWCNTs were safe and efficient for the targeted delivery of CDDP. Other examples of MWCNTs filled with CB, Irinotecan, Dexamethasone, Paclitaxel, L–OHP, and GEM for controlled cancer drug delivery are summarized in Table 8.

**Figure 24:**
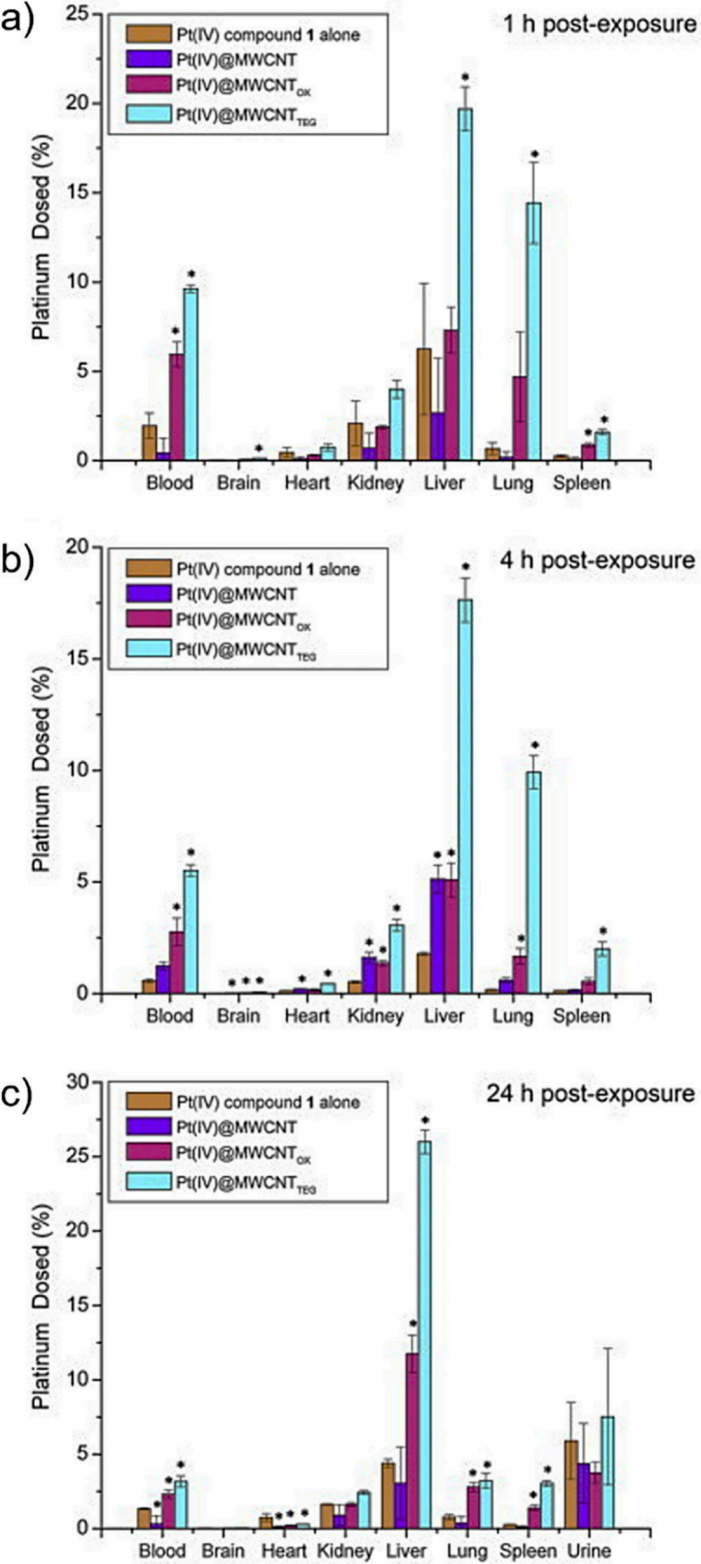
*In vivo* biodistribution of CDDP-filled MWCNTs in a female BALB/c mouse model. Tissue distribution of compound **1** alone (depicted in panel as inset), Pt­(IV)@MWCNT, Pt­(IV)@MWCNT_OX_ (MWCNT_OX_ = acid-oxidized MWCNT), and Pt­(IV)@MWCNT_TEG_ (MWCNT_TEG_ = amino-functionalized MWCNT) in female mice at a) 1 h post-exposure, b) 4 h post-exposure, and c) 24 h post-exposure. Data are presented as mean ± SD (*n* = 3). *Significantly different from compound **1** alone (*P* < 0.05).^731^ Reproduced with permission from ref 731. Copyright 2014, Elsevier.

Research on filled CNTs for cancer therapy has not been restricted to drug delivery systems, although the development of alternative therapeutic approaches reported in the literature is limited (Table 8). One example reported by Ding et al. showed the production of new theranostic agents based on MWCNTs filled with Fe for *in vivo* laser-induced thermotherapy and simultaneous magnetic resonance bioimaging (MRBI).^727^ The authors reported the significant tumor regression in an orthotopic mice model of human breast cancer treated with Fe-filled MWCNTs, due to their ability to generate thermoablative *T* by NIR laser irradiation (cancer cell death by hyperthermia occurs at *T* ranging from 40 to 42 °C).^744^ Fe-filled MWCNTs were also investigated as T2-weighted contrast agents for *in vivo* MRBI.^631^ More recently, Marega et al. reported the development of cetuximab (mAb) targeted Fe-filled CNTs for the therapy of epidermal growth factor receptor (EGFR)-overexpressing cell lines (EGFR^+^) using magnetic fluid hyperthermia.^728^ The *in vitro* cytotoxicity studies showed higher cancer cell death in lung cancer cell line A431 (EGFR^+^) compared to EAhy926, proving the specificity of Fe-filled MWCNTs conferred by the targeting ligand (Figure 25). The ability of Fe-filled MWCNTs to perform selective magnetic filtration of EGFR^+^ cells from a mixed population of healthy cell lines in a short period of time (10 min) was demonstrated.

**Figure 25:**
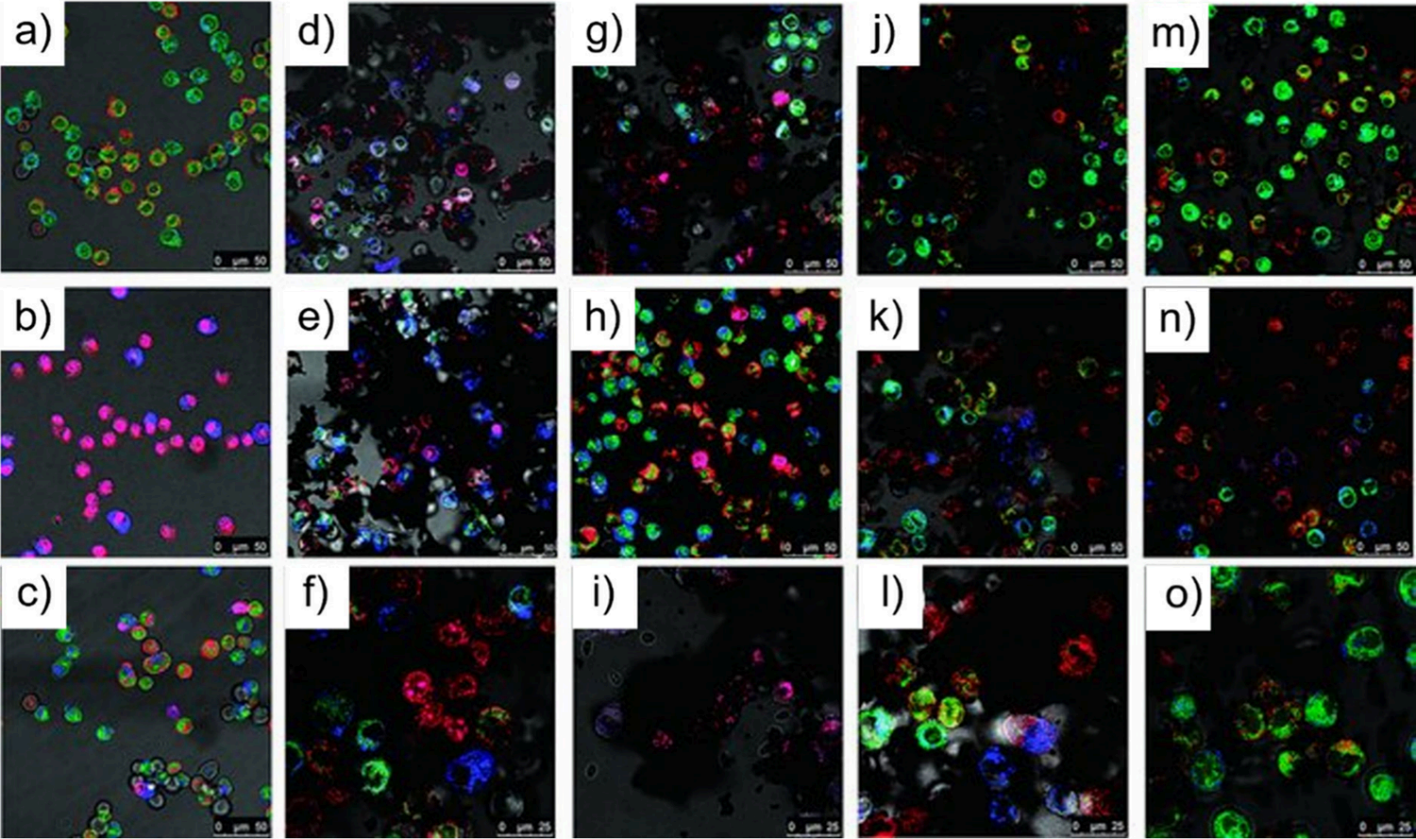
Cetuximab (mAb) targeted Fe-filled CNTs have been proposed for cancer therapy using magnetic fluid hyperthermia. Confocal microscopy images obtained after staining with ethidium bromide and acridine orange mixtures containing A431 cells (red) and Eahy926 cells (blue) a) without any magnetic treatment, b) after 10 min at 70 °C, c) after 20 min of ultrasonication, d–i) after addition of Fe@MWCNTs-NHCO-Ab and exposure to the electromagnetic irradiation, and j–o) after exposure with only Fe@MWCNTs-NHCO-Ab alone in the absence of any irradiation.^728^ Reproduced with permission from ref 728. Copyright 2013, John Wiley and Sons.

The radiotherapeutic efficacy of ^153^Sm@SWCNT and ^153^Sm@MWCNT nanocapsules was investigated *in vivo* using an experimental B16F10-Luc melanoma lung metastatic tumor model (Figure 26 a)).^745^ The term “nanocapsule” refers to closed-ended filled CNTs, thus preventing leakage of the encapsulated cargo.^57,167,746^ It was revealed that a fewer number of melanoma nodules were present in ^153^Sm@CNT treated mice (Figure 26 b)). Figure 26 c) shows the average tumor size (photons s^–1^) of untreated and treated mice with ^153^Sm@SWCNT or ^153^Sm@MWCNT. Histological examination showed that the ^153^Sm@CNT treated mice lung sections had a smaller number of melanoma nodule colonies. Several strategies have been developed to functionalize the walls of CNTs while preserving the encapsulated radionuclides. Therapeutic studies with ^153^Sm@CNT were also performed with CNTs bearing amine groups and using a monoclonal antibody (mAb) targeting the EGFR. Significant tumor growth reduction was induced by both treatments of ^153^Sm@MWCNTs functionalized with or without the antibody after a single intravenous injection. Although EGFR targeting showed no improvement in therapeutic efficacy, reduced splenic toxicity and normal hematological profiles were obtained for both functionalized derivatives.^747^ To complete the study, ^153^Sm@MWCNTs were also functionalized through an optimized (∼1 h) arylation reaction and evaluated using not only lung but also brain tumors. As in previous studies, no leakage of the internal radioactive material into the bloodstream was observed. In the treated mice, the highest uptake was detected in the lungs, followed by the liver and spleen. Presence of tumors in brain or lung did not increase percentage accumulation of ^153^SmCl_3_@MWCNTs-NH_2_ in the respective organs, suggesting the absence of an enhanced permeation and retention effect.^748^ More recently, the use of nanocapsules for lithium neutron capture therapy (LiNCT) has also been proposed.^736^

**Figure 26:**
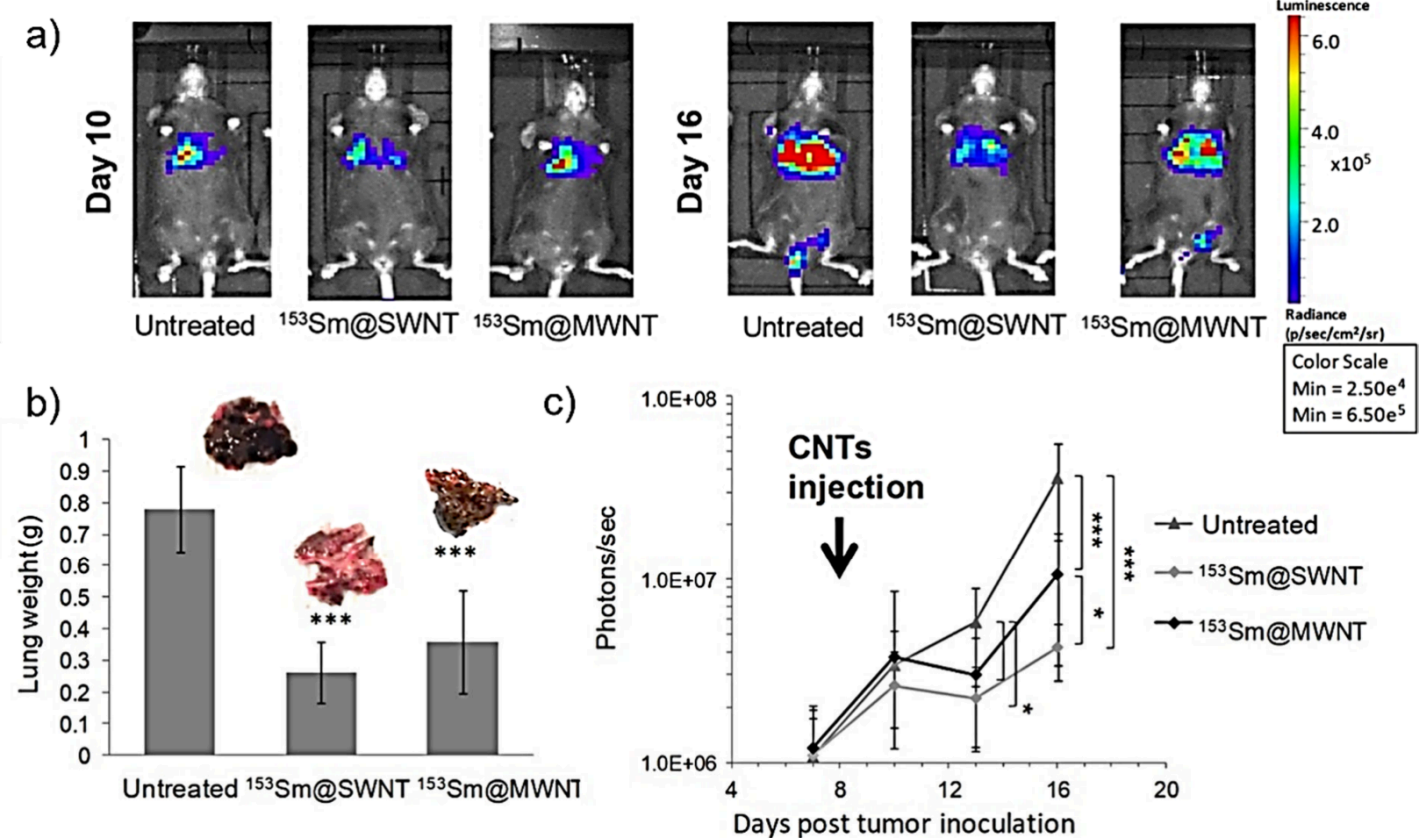
Radiotherapeutic efficacy of ^153^Sm@SWCNT and ^153^Sm@MWCNT nanocapsules. a) Whole-body bioluminescence images, b) average lung weights and average c) tumor size (photons s^–1^) for initial, ^153^Sm@SWCNT, and ^153^Sm@MWCNT treated mice.^745^ Reproduced with permission from ref 745. Copyright 2020, American Chemical Society.

#### 8.1.2 Bioimaging Agents

Beyond cancer therapy, CNTs also offer the possibility of being filled in their hollow cavity with materials with high bioimaging relevance for the development of more accurate contrast agents, to monitor different types of subcellular structures, cells, and tissues.^701^ Significant progress has been made in recent years using SWCNTs for the development of new “out of the box” strategies for bioimaging (Table 8).

In 2005, Wilson et al. demonstrated the ability of short SWCNTs (with high surface area values, 1180 m^2^ g^–1^) for the physisorption of radionuclides (^125^I^–^) from aqueous solutions.^749^ Two years later, the same group explored the potential of iodine-filled CNTs for CT. Iodine was filled into short SWCNTs, and the external surface was functionalized with serinolamine groups to improve water dispersibility. The CT images obtained with the hybrids I_2_-filled SWCNTs (ca. 10% iodine by weight from XPS) was twice more opaque than non-filled SWCNTs.^715^

Spinato et al. reported the successful preparation of carbon nanocapsules by encapsulating of radioactivable MX, SmCl_3_ and LuCl_3_, on steam-purified SWCNTs sealed by thermal treatment. The external surface of the SWCNTs nanocapsules was functionalized with mAb antibodies to induce specific targeting of EGFR. *In vitro* fluorescence imaging showed the ability of these targeted nanocapsules to preferentially internalize into U87-EGFR^+^ cells, overexpressing EGFR, in comparison to CHO cells employed as control.^387^ Serpell et al. demonstrated the ability of these carbon nanocapsules to hermetically seal toxic elements and even a gas (krypton) for X-ray fluorescence bioimaging.^689^ The external surface of nanocapsules was decorated with specific peptides for mapping the subcellular organelles.

Carbon nanocapsules have also been used for *in vivo* imaging. In 2010, Hong et al. reported on the hermetic sealing of radionuclides within the cavities of SWCNTs. In the first study, Na^125^I was filled into SWCNTs and functionalized with biantennary carbohydrates, resulting in highly efficient *in vivo* radioprobes.^718^ Specific tissue accumulation (lung) coupled with high *in vivo* stability prevented leakage of radionuclide to high-affinity organs (thyroid/stomach) or excretion, and resulted in ultrasensitive imaging and delivery of unprecedented radiodose density.

Pascu et al. significantly expanded the bioimaging capabilities of filled CNTs. Hybrid materials based on supramolecularly assembled SWCNTs were generated for PET, magnetic resonance imaging (MRI), and fluorescence imaging. The all-in-one imaging probe allowed quantitative imaging from subcellular resolution to whole-tissue regions. *In vivo* uptake in Wistar rats revealed rapid accumulation of radioactivity in the lungs and myocardium. Furthermore, such materials are fully traceable in cells by multiphoton fluorescence lifetime imaging with NIR excitation.^750^

Gadolinium-filled CNTs, also known as gadonanotubes (GdNTs), have been widely explored as high-performance T1MRI contrast agents.^751^ The high-resolution images provided by Gd complexes are considered important for efficient clinical diagnosis.^752^ Nevertheless, current clinical Gd-based complexes present poor selective delivery, water solubility, bioaccumulation and significant toxicity.^753^ The risks of secondary effects of Gd complexes can be considerably reduced by encapsulating them into CNTs. Remarkably, filling ultrashort SWCNTs with Gd­(acac)_3_.2H_2_O, Gd­(hfac)_3_.2H_2_O and Gd­(thd)_3_ (hfac = hexafluoroacetylacetone, thd = tetramethylheptanedione) results in extremely high T1-weighted relaxivities (>100 mm^–1^ s^–1^).^754^ The confinement effects imposed by the CNTs walls on Gd^3+^ ion sites promote a large hydration number. Therefore, the significant decrease in the distances between Gd–H results in a high proton relaxivity.^755^ Wilson et al. reported, for the first time, the ability of GdNTs to internalize J774A.1 murine macrophage cells for improved *in vitro* biomapping. Cell proliferation MTS assays showed the ability of GdNTs for cell internalization without cytotoxic effects, for concentrations of Gd lower than 28 μM.^719^ The potential of the GdNTs was further analyzed as *in vivo* bioimaging agents using C57BL/6 mouse models. Short SWCNTs were also explored for bimodal *in vivo* bioimaging (PET and MRI) on tumor-free athymic nude mice, by filling them with Gd and copper-64.^720^

Iron-filled CNTs have also been explored for *in vitro* and *in vivo* MRI. However, because iron-filled CNTs can also be employed for therapeutic applications using hyperthermia treatment,^727,728,733^ thus acting as theranostic agents, these hybrids have already been discussed in the previous section.

#### 8.1.3 Nanothermometry

Klingeler et al. pioneered the application of filled CNTs as nanothermometers for determining *T* at the cellular level.^756^ For this purpose, MWCNTs were filled with CuI, which presents a *T* dependence of NMR frequency and relaxation time. The nuclear spin–lattice relaxation of ^127^I was explored as a sensitive *T* parameter, which can provide a *T* accuracy of up to 2 °C. Besides, the authors stated the need for new *T*-dependent NMR filling materials, with enhanced *T* accuracy of up to 0.1 °C.

#### 8.1.4 Multifunctional Scaffolds

The singular features of CNTs have been the subject of intense research as active nanomaterials for the assembly of three-dimensional (3D) scaffolds with applications in tissue engineering and regenerative medicine.^244^ Only a few examples of filled CNTs can be found as part of multifunctional scaffolds. To the best of our knowledge, the first report dates back to 2010 and consists of poly­(lactic-*co*-glycolic acid) (PLGA) scaffolds reinforced with Gd-filled CNTs. In this study, GdNTs were mainly used to monitor the *in vivo* fate of CNTs during the *in vivo* biodegradation of the polymer matrix by MRI.^757,758^ An increase in the MRI intensity of the scaffold’s surrounding tissue was observed after 3 weeks of in vivo implantation, suggesting the release of GdNTs resulting from the biodegradation of the PLGA scaffold. Histological studies showed the formation of connective fibrous tissue around multifunctional GdNTs/PLGA scaffolds and some mild inflammatory signals.^758^ Wilson et al. reported the use of an external magnetic field to stimulate GdNT-labeled mesenchymal stem cells (MSCs) to improve the retention of transplants during cellular cardiomyoplasty.^759^ *In vivo* tests using porcine models showed that an external magnet increased the retention of Gd-labeled MSCs by 3 orders of magnitude compared with Lu-labeled cells (Figure 27). Despite being a proof-of-concept study, this work opens up a strategy to explore GdNTs as magnetic T1-weighted NPs to improve implanted cell retention.

**Figure 27:**
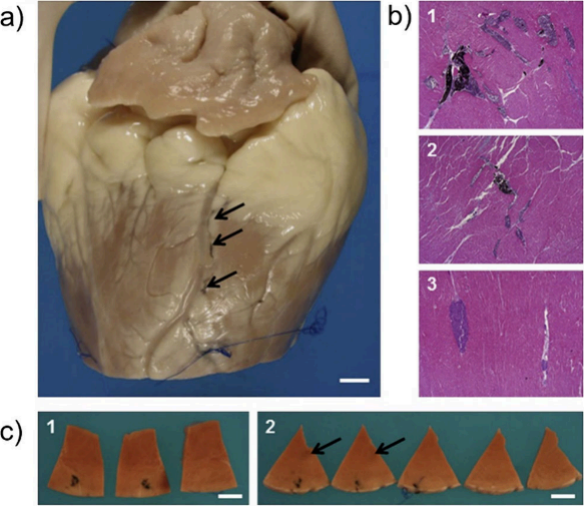
Histopathological analysis of ex vivo perfusion study. a) Photograph of the perfused heart after formalin fixation. Arrows indicate venous drainage of GdNT-labeled MSCs above Site 2. Scale bar = 1 cm. b) Hematoxylin and eosin staining of injection sites 1, 2, and 3, illustrating the presence of the darkly colored GdNT-labeled MSCs. × 4 Magnification. c) Various levels of tissue segments containing injection sites 1 and 2. The arrows denote an area of grayish discoloration, suggestive of disseminated GdNT-labeled MSCs. Scale bar = 1 cm.^759^ Reproduced with permission from ref 759. Copyright 2014, Elsevier.

### 8.2 Energy Storage

CNTs have been widely explored for energy storage applications over the past few decades.^760^ The peculiar structure of CNTs provides unique properties to active materials confined within their cavities, boosting their performance for energy storage and conversion applications. Their high electrical conductivity, large surface area, and mechanical, chemical, and EC stability can have an extraordinary impact on the development of new hybrid materials that combine the benefits of both filler and host.^761^ CNTs provide the opportunity to work with compounds unstable under working conditions, as in the case of 2,6-naphthoquinone,^762^ which has demonstrated potential as an electrode for Na and lithium-ion batteries (LIBs), and whose main drawback is its high solubility in organic solvents commonly used as electrolytes. Once encapsulated, CNTs not only provide protective barrier to avoid dissolution or degradation, but also contribute to improving aspects such as electrical conductivity and thermal stability. The rigid shell-like character of CNTs also enables the stabilization of the confined material. Under the operation conditions, the electrochemically active systems often undergo structure and volume variations that may limit their performance by inducing mechanical stress and strain consequently leading to degradation and capacity fade.^763^ Structure modification of CNTs has been frequently reported to enhance the EC performance. N is the most widely used heteroatom to tune the electronic structure of CNTs, even so, other reports include B, P, or S, or a mixture of the heteroatoms (codoping) within their conjugated skeleton. Owing to its electron-donor character, N induces n-type conductivity, thus improving the storage capacity of the system. Moreover, N can contribute to enhance the adsorption and intercalation of the incoming molecules. In case of supercapacitors, N-doping induces an Electronic Double-Layer Capacitor behavior (EDLC). Its advantages include a higher hydrophilicity induced by the introduction of N-based groups, an improved electrolyte adsorption or favoring the charge accumulation and energy density. Similar to N, other heteroatoms provide substantial improvements to the EC system. The modification induced to the structure of the hosting CNTs and therefore the acquired properties depend on the size, electronic structure and affinity of the heteroatom with the C lattice. Table 9 shows the most commonly employed heteroatoms incorporated within the structure of CNTs for their applications in energy storage.

**Table 9:** Effects on Using Doped CNTs as Hosting Nanostructures for Energy Storage Applications

Dopant(s)	Induced modification	Effects on Battery Performance	Effects on Supercapacitor Performance
**Nitrogen (N)**	*n-type* doping.	• Increases conductivity.	• Favors pseudocapacitance.
		• Enhances electron transport and rate capability.	• Improves Wettability and electrolyte adsorption enhancing EDLC.
		• Improves ion kinetics: Defects lower the energy barrier (Li-ion diffusion).	• Increases Conductivity.
		• Enhances structural stability to mitigate volume expansion.	• Enhances electron transport and rate capability.
		• Enhances polysulfide trapping, improving life cycle.	
**Boron (B)**	*p-type* doping.	• Increases electrical conductivity due to high hole concentration.	• Favors pseudocapacitance.
		• Enhances ion adsorption.	• Enhances ion adsorption thus contributing to EDLC.
		• Minimizes lattice distortion due to size similarities with carbon.	• Increases electrical conductivity.
**Phosphorus (P)**	*n-type* doping and lattice strain.	• Increases electronic conductivity.	• Favors pseudocapacitance.
		• Increases reactive sites due to lattice distortion and defects favoring ion storage.	• Increases porosity and interlayer spacing improving electrolyte accessibility and diffusion.
		• Favors electrolyte infiltration by improving surface area owing to strain.	• Improves conductivity.
**Sulfur (S)**	*n-type* doping and lattice strain (often at the edges).	• Improves conductivity.	• Favors pseudocapacitance through redox reactions.
		• Favors polysulfide anchoring, therefore suppressing the shuttle effect.	• Increases surface activity.
		• Improves wettability and ion transport.	• Improves wettability, ion adsorption and EDLC.
			• Improves conductivity.

Finally, by combining different heteroatoms and strategically locating them inside the *sp* ^*2*^ network, a complex and tunable structure can be created. Authors have taken advantage of the disparate properties of the elements to induce synergistic effects that favor the EC reactions within the CNTs’ cavities. B–N codoping, for instance, creates a complex and balanced electronic landscape composed by *n*- and *p*-type conduction active sites that favor ion adsorption and diffusion. The structural stability of the system is reinforced by the creation of strong covalent bonds and the charge distribution effectively promotes the redox reactions thus boosting the specific capacitance of the system. These materials have therefore demonstrated to be suitable as electrodes for applications in supercapacitors.^764,765^ On the other side, N–S codoped CNTs have been reported as potential electrodes for battery applications. The electronic network, formed by N- and S- based sites are particularly akin and establish strong physical and chemical interactions with polysulfides, thus minimizing the shuttle effect and leading to superior cycle stability and capacity retention.^766^ Other examples include improved charge distributions, presence of more active sites and efficient catalytic activity in metal–air batteries containing N–S codoped hosts.^767,768^

Next, we present in detail some approaches already explored using filled CNTs for the development of new hybrid materials for energy-storage applications.

#### 8.2.1 Supercapacitors

Initial studies on the EC response of close-ended filled SWCNTs revealed that EC opening occurs upon the application of either a sufficiently oxidizing or reducing electrode potential.^346^ Later, an improvement in the EC properties of the electroactive material MnO_2_ was observed after being inserted into the hollow cavities of MWCNTs.^769^ The results demonstrated a significant enhancement in specific capacitance, reaching 289.2 F g^–1^ for the hybrid materials, five times higher than that of pure MWCNTs. Additionally, the MnO_2_/MWCNTs hybrid used as the supercapacitor material showed superior cycling stability in the potential range of 0–1.0 V and retention of 96% of the initial capacitance, even over 200 cycles. The authors concluded that MnO_2_/MWCNTs supercapacitors had improved structural stability and a long-life cycle. Recently, Zhang et al. reported that FeX_*n*_ (X = O, C, and P) filled N-doped CNT-based supercapacitors exhibited a specific capacitance of 392.0 F g^–1^ at a current density of 0.5 A g^–1^. The hybrids were easily and economically synthesized, with specific surface areas of up to 566.1 m^2^ g^–1^. The protective character of the hosting CNTs plays an important role in the negligible capacity loss after 50000 cycles.^770^

Solution phase voltammetry was used to study the cyclic EC performance of different POM-filled SWCNTs (POM@SWCNTs).^771^ The filling process, which was self-driven by electrostatic forces between the POMs and SWCNTs, was performed in an aqueous solution at RT (Figure 28).

**Figure 28:**
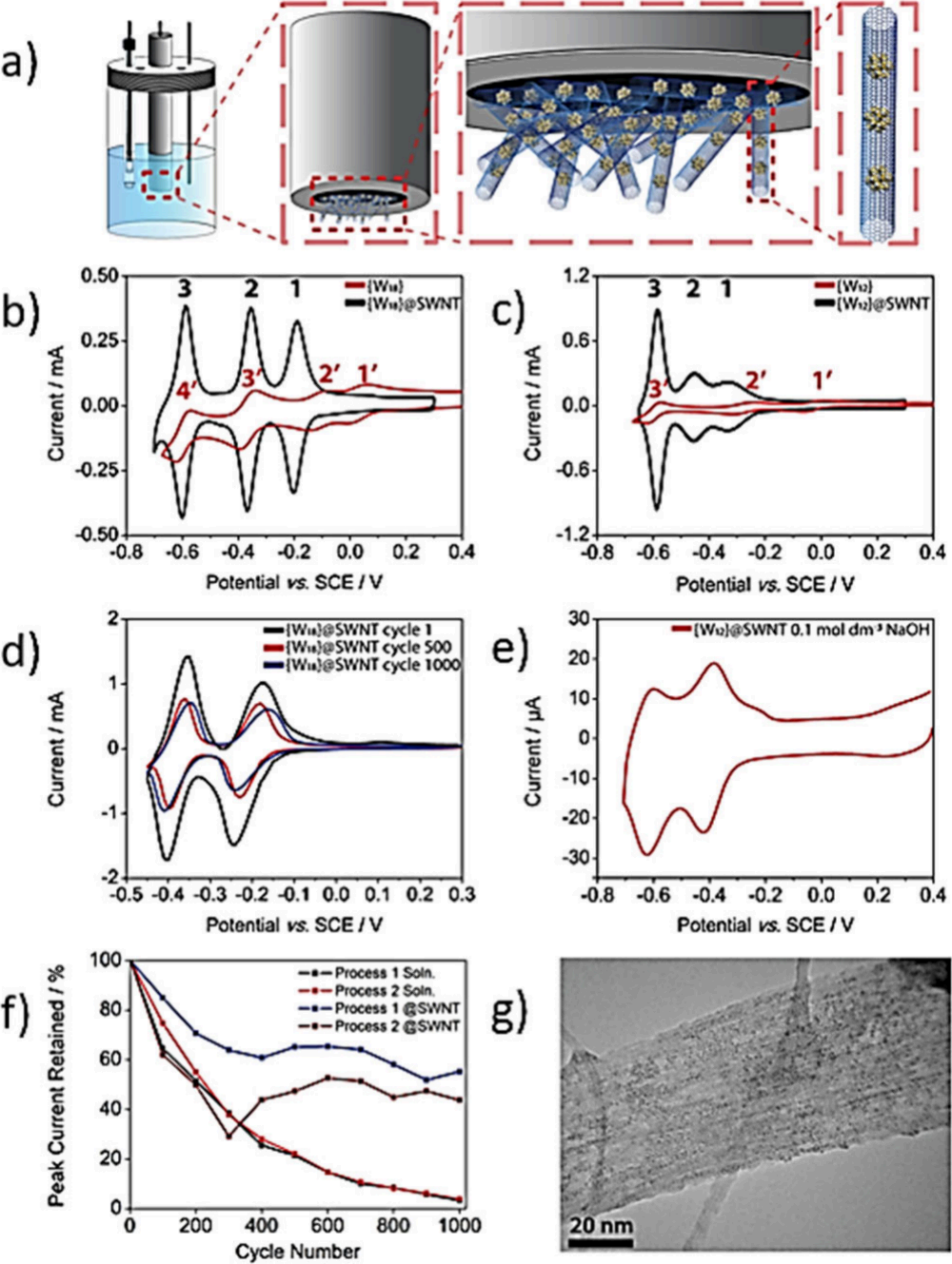
EC performance and electron microscopy of POM filled SWCNTs. a) Schematic representation of an EC cell. The working electrode was a glassy electrode. b, c) CVs of {W_18_} and {W_12_} (red lines) and immobilized {W_18_}@SWCNT and {W_12_}@SWCNT films (black lines). The reduction and oxidation peaks are indicated in the figure. d) 1st (black), 500th (red), and 1000th (blue) CVs of the immobilized {W_18_}@SWCNT. Scan rate of 100 mV s^–1^ in aqueous H_2_SO_4_ (1.0 mol dm^–3^). e) CV of the immobilized {W_12_}@SWCNT at 100 mV s^–1^ in aqueous NaOH (0.1 mol dm^–3^). f) Peak current for {W_18_} in solution (black and red plots) and encapsulated inside SWCNTs (blue and brown plots) plotted versus the cycle number. Process 1 and Process 2 are attributed to the 1st and 2nd reductions of the POM, respectively. g) The TEM micrograph of the bundle of SWCNTs in the {W_18_}@SWCNT sample after 1000 EC cycles.^771^ Reproduced with permission from ref 771. Copyright 2019, John Wiley and Sons.

The spontaneous filling is stable and irreversible, facilitating the efficient scalable production of densely filled POM@SWCNTs hybrid materials. The cyclic voltammograms (CVs) of {W_12_} and {W_18_} (red lines in Figure 28 a–c)) are consistent with the expected CVs of these materials.^771^ The CVs after filling the SWCNTs with the POM and immobilizing the POM@SWCNTs on a glassy carbon electrode (black lines in Figure 28 b) and c) show that the encapsulated POMs maintain their EC activity. The first two peaks in the encapsulated {W_12_} were shifted negatively by approximately 0.2 eV, and the separation of the oxidation and reduction peaks (Δ*E*_p_) decreased to only 10 mV at a scan rate of 100 mV s^–1^. The strongly reduced Δ*E*_p_ is very close to Δ*E*_p_ = 0 in a fully reversible surface-confined redox couple. With increasing scan rate, the peak currents for the oxidation and reduction of {W_18_}@SWCNT and {W_12_}@SWCNT increased linearly. This linearity confirms that the POMs are homogeneously connected to the glassy carbon electrode. This is plausible if the POMs are uniformly packed inside the SWCNTs. The integrated charge between the first redox peaks in the CVs of {W_18_}@SWCNT and {W_12_}@SWCNT yielded surface concentrations (Γ) of 33 and 19 nmol cm^–2^, respectively. These values imply that 80–90% of the encapsulated POMs inside the SWCNTs on the glassy carbon electrodes (GCE) are electrochemically addressable. With increasing scan rate, Δ*E*_p_ only increases slightly and Γ remains unchanged, indicating an efficient and rapid electron transfer between the electrodes and the POMs. Repeated cycling up to 1000 times showed that while free {W_18_} loses its activity completely, the decline in charge capacity of {W_18_}@SWCNT initially tapers off and stabilizes at around 58% of the initial value (Figure 28 d), f)). The stabilization of POM in {W_18_}@SWCNT is attributed to maintained encapsulation. The TEM micrograph of {W_18_}@SWCNT after 1000 cycles (Figure 28 g)) shows that the majority of the POM remains encapsulated (Figure 28 e)).

Electrodes for supercapacitors were also developed by encapsulating chromium oxide into highly conductive SWCNTs.^772^ The resulting hybrid had a desirable combination of pseudocapacitance and conductivity for high-performance EC supercapacitors. In a recent study,^773^ Sn-filled MWCNTs suitable for EC experiments were reported. Figure 29 shows the HRTEM data of the filled MWCNTs before and after the EC experiments.

**Figure 29:**
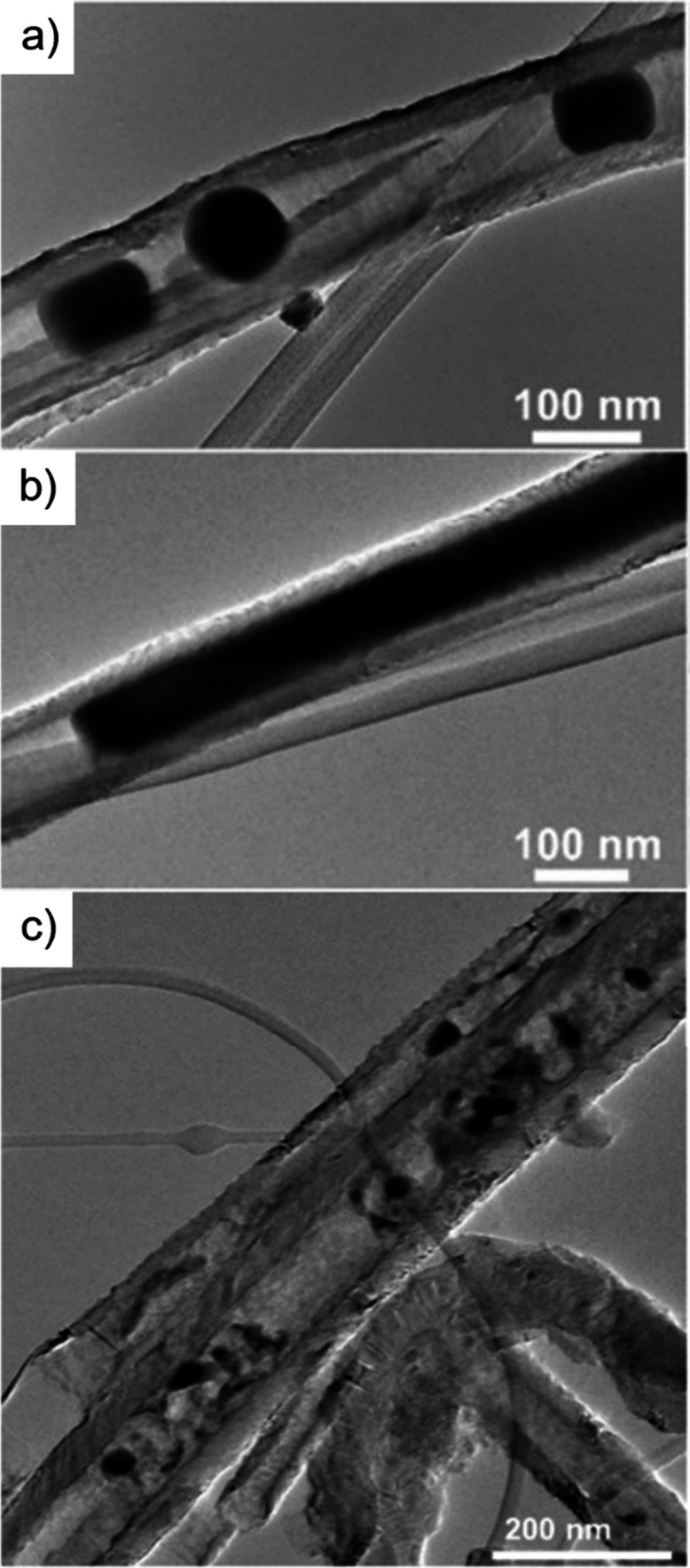
Microscopic evaluation of Sn-filled MWCNTs. HRTEM images of a, b) Sn-filled MWCNTs and c) after being galvanostatically cycled (10 cycles).^773^ Reproduced with permission from ref 773. Copyright 2020, MDPI.

Gao et al. developed a technique that involves the etching of Ni–Co Prussian blue, used as precursor to selectively encapsulate NiSe_2_ and CoSe_2_ NPs within CNTs.^774^ The approach prevents the accumulation of Ni/Co within the host and leads to 1D hybrids with specific capacity up to 375% higher than that obtained with materials prepared by conventional filling protocols. The authors built electrodes that reached a specific capacity of 105.1 mAh g^–1^ at a 1 A g^–1^ current density and asymmetric supercapacitor devices with energy densities of up to 46.3 Wh kg^–1^ at 801 W kg^–1^. After 5000 charge–discharge cycles at 10 A g^–1^, the electrode showed 83% capacity retention.

#### 8.2.2 Lithium, Sodium and Potassium Ion Batteries

##### 8.2.2.1 Anode Material for Lithium, Sodium and Potassium Ion Batteries

In recent years, filled CNTs have been explored as electrodes for LIBs. CNTs filled with metal sulfides and oxides are the most promising candidates for application as anode materials in LIBs.^447,775^ The number and nature of materials proposed for this purpose are constantly increasing. Most reports suggest improved EC performance. Aspects such as the morphological control of the crystals, stability, protection against external degradation agents,^776^ and suppression of the polysulfide shuttle effect^777^ are highlighted.

The first evidence of the EC behavior and lithiation mechanisms of carbon hybrid materials was reported in 2013 by Su et al.^778^ The authors used Co_9_S_8_/Co filled CNTs as a model for the *in situ* investigation of the Li storage mechanism using TEM. The lithiation of Co_9_S_8_/Co-filled CNTs with open ends resulted in a 94.2% axial elongation of the filler. In contrast, the closed-end Co_9_S_8_/Co-filled CNTs exhibited a preferential radial expansion of 32.4% upon lithiation. Notably, the closed-end CNTs demonstrated greater dimensional stability during lithiation-delithiation cycles, which could enhance the EC Li storage performance of these hybrid materials. In open-ended CNTs, the filler was observed to extrude following lithiation; however, the formation of a graphitic shell maintained the structural integrity of the extruded filler throughout the repeated cycles.

Hybrid SnO_2_@CNTs, prepared by a wet chemical filling method, were also explored as anode materials for LIBs.^779^ The hybrid electrode, containing 65 wt % of SnO_2_, showed a high reversible capacity of 627.8 mAh g^–1^ after 50 cycles at a current density of 0.2 mA cm^–2^. Additionally, when the current density was increased to 1.0, 2.0, and 4.0 mA cm^–2^, the hybrid electrodes still exhibited reversible capacities of 563, 507, and 380 mAh g^–1^, respectively. In another study, Sn NP-filled MWCNTs, fabricated by AD, were proposed for LIB anodes.^780^ The hybrid Sn@CNTs electrode showed an initial specific capacity of 850 mAh g^–1^ at a constant current density of 100 mA g^–1^. After three charging cycles, the specific capacity decreased and stabilized at approximately 600 mAh g^–1^. The decrease in specific capacity was less abrupt for the hybrid Sn@CNTs electrode than for the Sn NPs electrode as the number of cycles increased. In 2016, Yu et al. reported the reaction of sublimated sulfur with FeCp_2_ for the growth of FeS NPs inside the internal cavities of CNTs. FeS@CNT showed a reversible charge capacity of 851.2 mAh g^–1^ in the first cycle and a constant charge capacity of 670 mAh g^–1^ after 65 cycles. Dynamic lithiation analysis of FeS@CNT hybrids by TEM showed that CNTs played an important role in restricting the volume expansion of the filler material and providing fast transport pathways for Li^+^ ions. (Figure 30).^281^

**Figure 30:**
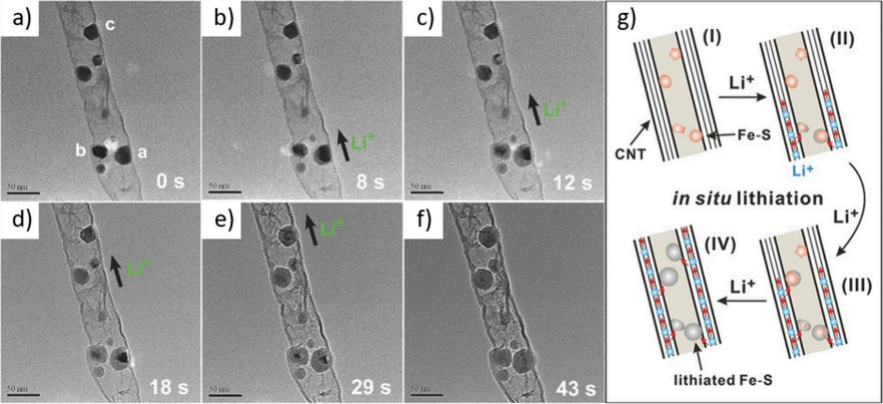
Dynamic lithiation analysis of Fe–S@CNT hybrids. Snapshot series of the in situ lithiation of Fe–S@CNT with densely filled Fe–S NPs. a) CNT with a dense filling of Fe–S NPs. b) Lithiation of particle “a” begins. c–e) Sequential lithiation of different Fe–S NPs along the CNT. b–e) Arrows in the images indicate the Li transport direction and starting lithiation sites. f) TEM image of Fe–S@CNT after full lithiation. g) Schematic of the lithiation process of Fe–S@CNT.^281^ Reproduced with permission from ref 281. Copyright 2016, MDPI.

Silicon has the highest theoretical Li storage capacity of 4200 mAh g^–1^. Nevertheless, its low-dimensional stability during the charge/discharge process promotes fast degradation of its performance as an anode material in lithium batteries. Yu et al. reported the lithiation of Si NPs filled into CNTs prepared by CVD.^781^ The authors observed that the transformation of Si NPs to Li_*x*_Si upon lithiation can promote a volume expansion of ∼180%. However, the confinement of Si NPs within CNTs could limit the expansion during the lithiation reaction to only ∼14% without breaking the tubular structure of CNTs (Figure 31). Furthermore, it was observed that the lithiation process of Si NPs was significantly improved owing to the high electronic and ionic conductivity of the CNTs. The experimental results revealed that Si@CNTs exhibited a high reversible capacity of 1684.1 mAh g^–1^ at 100 mA g^–1^, 1200 mAh g^–1^ at 500 mAh g^–1^, and 614.6 mAh g^–1^ at 2000 mA g^–1^.

**Figure 31:**
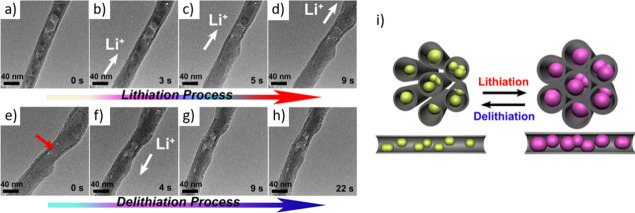
Dynamic structural changes in Si NP-filled CNT under EC lithiation/delithiation. a–d) Lithiation of Si NP-filled CNT. The arrows in images b–d) indicate the Li^+^ transport direction and the site where Li_*x*_Si began to form during lithiation. e–h) Delithiation of the same Si NP-filled CNT. The arrow in image f) shows the Li^+^ transport direction during delithiation. i) Illustration of the lithiation/delithiation of Si NP-filled CNTs.^781^ Reproduced with permission from ref 781. Copyright 2015, American Chemical Society.

More recently, within the race to overcome the limitations due to the severe volume expansion of Si, MWCNTs were used as templates for the growth of SiO_2_ NTs in their outer shell (using a sol–gel approach), thus creating a hollow structure capable of accommodating a large number of Li^+^ species. A carbon coating was then used to cover the surface of the SiO_2_ NTs that contributed to mitigate drawbacks related to the inherent low conductivity and low initial Coulombic efficiency of the SiO_2_-based structure.^782^

Red P-filled SWCNTs were also explored in LIB.^308,783^ Figure 32 shows the long-term cycling of composite nanomaterials at 5 A g^–1^ and the dependence of voltage on specific capacity curves measured at the 65th cycle at a current density of 0.1 A g^–1^. A specific capacity of 1545 mAh g^–1^ at a current density of 0.1 A g^–1^ was observed for the filled SWCNTs. The encapsulated P showed a higher reaction rate and a ∼7% loss of initial capacity after 1000 cycles at 5 A g^–1^.

**Figure 32:**
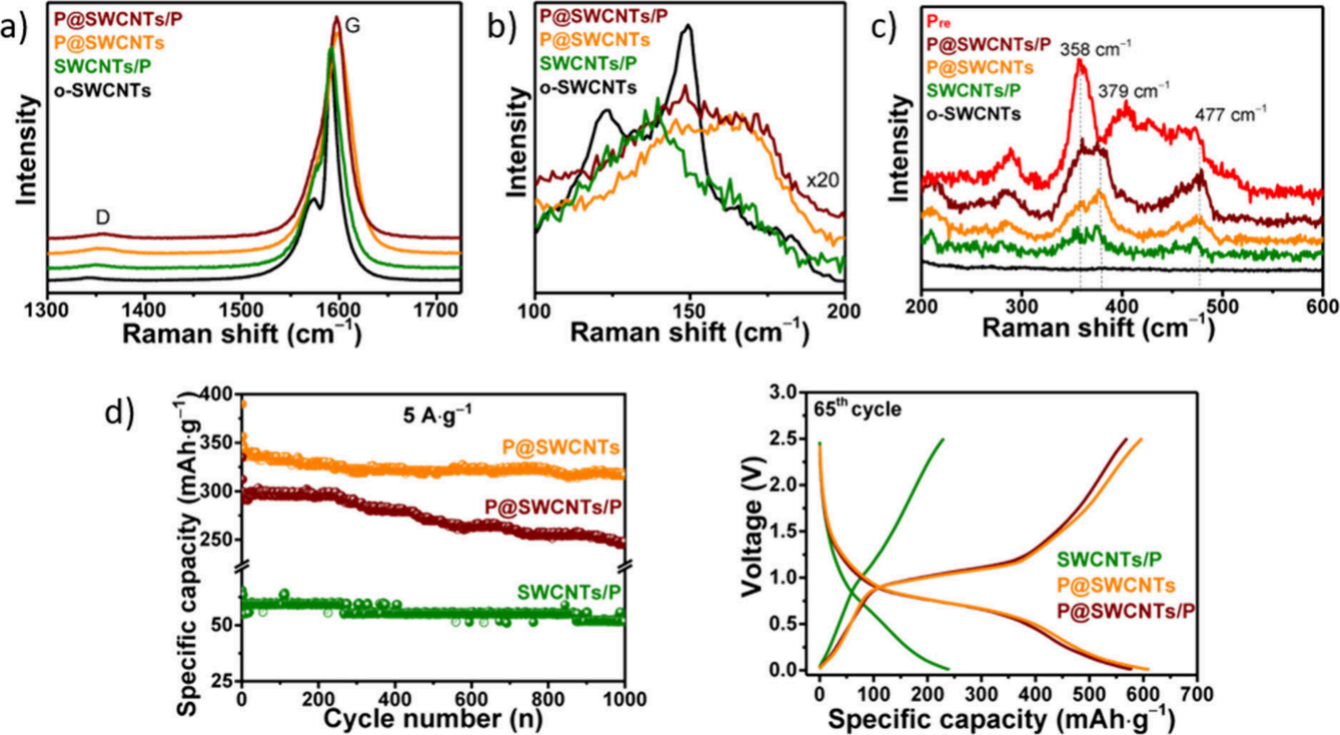
a–c) Raman spectra of the pristine SWCNTs, red P deposited on outer SWCNT surface, cleaned P-filled SWCNTs, filled SWCNTs with P on the outer surface. d) Long-term cycling of composite nanomaterials at 5 A g^–1^, and the dependence of voltage on specific capacity curves measured at 65th cycle at a current density of 0.1 A g^–1^.^783^ Reproduced with permission from ref 783. Copyright 2022, MDPI.

The low abundance and high extraction costs of Li have led researchers to consider new alternatives for ion-based batteries that incorporate more abundant alkali metals, such as Na, K, and Ca. Owing to the inability of Na to form stable intercalation compounds with graphite and the low redox potential of K, which pose scientific challenges when exploring the introduction of these metals as anode materials, CNTs have recently been proposed as attractive alternatives to replace graphite. This would potentially solve stability problems and might also improve performance owing to their large surface area and electrical conductivity.

As in the case of LIBs, CNTs filled with red P have been proposed as potential anode materials for sodium-ion batteries (NIBs).^784^ Denoted as RP-filled carbon nanocages (RP@CNCs), they allow an 85.3% loading of red P and exhibit a high reversible capacity (1363 mA h g^–1^ at a current density of 100 mA g^–1^ after 150 cycles) and a long-term cycling performance. Using another approach, Xu et al. prepared ternary FeS@TiO_2_@CNTs, which increased the channels and active sites for Na transport. As a consequence, an improved rate capability (up to 390 mAh g^–1^) and cycling performance was obtained when using the material as anode for NIBs.^785^

The performance of the prepared hybrids can be enhanced by incorporating N atoms within the conjugated lattice (N-doping). Several examples including the encapsulation of CoO^786^ and Bi^787^ have shown superior metal storage due to the synergetic effect of both, the encapsulation within the cavities of the CNTs and their modification by N-doping.

##### 8.2.2.2 Cathode Material for Lithium-Ion Batteries (LIBs)

Sulfur has a high potential for application as a cathode for LIBs because this element has the highest theoretical specific capacity value of 1673 mAh g^–1^. Nevertheless, some critical factors, such as poor electrical conductivity, dissolution of Li polysulfides, and large volume changes during different cycles, have limited their application as commercial cathodes for Li batteries. A viable alternative for exploring S as an electrode material is its confinement in the inner cavity of CNTs. Therefore, there is a continuous quest to improve the encapsulation of elemental S within CNTs is currently in progress. A variety of new Raman signals appear after filling CNTs with S, which can be used to monitor the stability and EC behavior of the filled CNTs.^788^ A chemical surface enhanced Raman spectroscopy effect was observed upon S encapsulation within narrow CNTs (*d* = 0.89 nm), as a consequence of charge transfer induced by the presence of chiral chains of S within the cavities of the host.^789^ Tonkikh et al. encapsulated S chains within small diameter SWCNTs and evaluated the spectroscopic response of transparent cells prepared from the S@SWCNTs composites.^300^ *In situ* Raman and UV–vis spectra, during short-term EC charging, showed both modifications in the intensity and shifts up to 10 cm^–1^ in the vibrational bands of the sulfur chains. The reversibility of the reactions was confirmed upon charge removal, demonstrating recyclability and chemical stability.

The *in situ* lithiation effect and mechanism in open-ended S@CNTs have already been investigated *in situ* by TEM.^790^ The lithiation reaction with S starts from the ends of CNTs and propagates along the length, promoting the expansion of the material through the empty cavities of the CNTs (Figure 33 a–c)). It was observed that the lithiation of S near the conductive walls of CNTs occurred at the same rate as that in the core of the filler material, which suggests the presence of diverse electron pathways at the Li_2_S/S interface.

**Figure 33:**
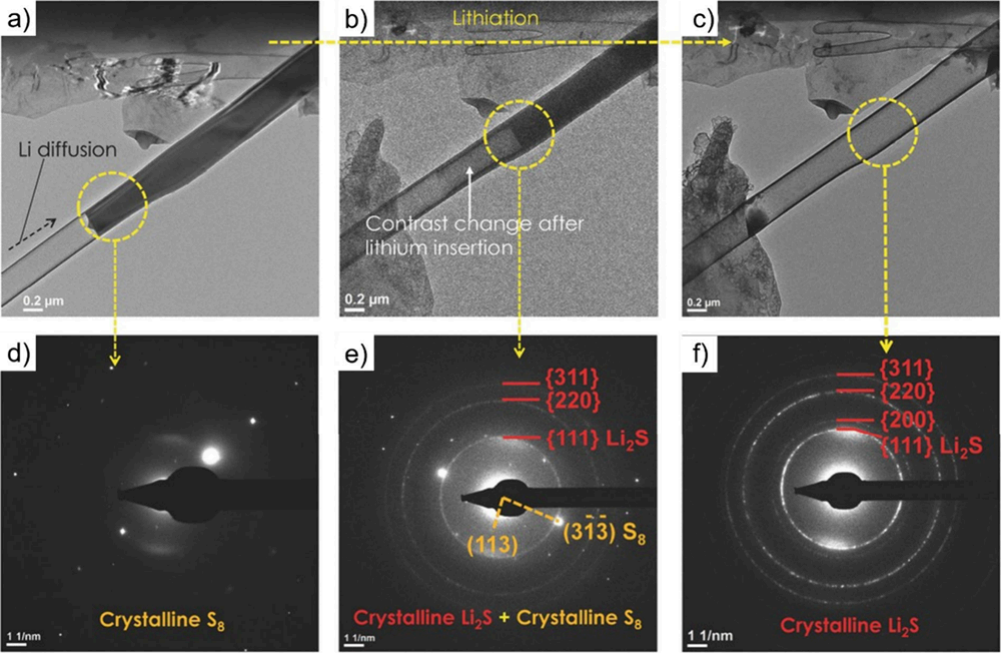
Selected images of the S@CNTs sample evolution during a typical lithiation process. a–c) TEM images captured during the lithiation of S nanoconfined in a CNT reaction vessel and d–f) their corresponding EDP patterns; panels a) and d) correspond to the sample before the initiation and panels c) and f) after the completion of the EC reaction. The darker area appearing at the bottom left corner of the CNT in panel c) is a contaminant.^790^ Reproduced with permission from ref 790. Copyright 2015, John Wiley and Sons.

Reported alternatives include the sonically assisted capillary method using THF, which is a solvent with low surface tension, and reaches up to 36.2% S loading inside the CNTs cavities.^791^ A remarkable study by Jin et al. proposed filling large CNTs with small-diameter CNTs and a high amount of S (85.2 wt %), to overcome the critical limitations reported for pure S cathodes (Figure 34).^792^ The hybrid S-CNTs@CNT electrode showed a large discharge capacity of 1633 mAh g^–1^, similar to the theoretical value predicted for pure sulfur (1673 mAh g^–1^), which proves the effectiveness of the strategy developed. The assembled hybrid electrode showed high discharge capacity values of ∼1146, 1121, and 954 mAh g^–1^ after 150 cycles at large current rates of 1, 2, and 5 C, respectively. The obtained results highlight the potential of the proposed hybrid S-CNTs@CNT electrodes for designing new cathode architectures for lithium–sulfur batteries.

**Figure 34:**
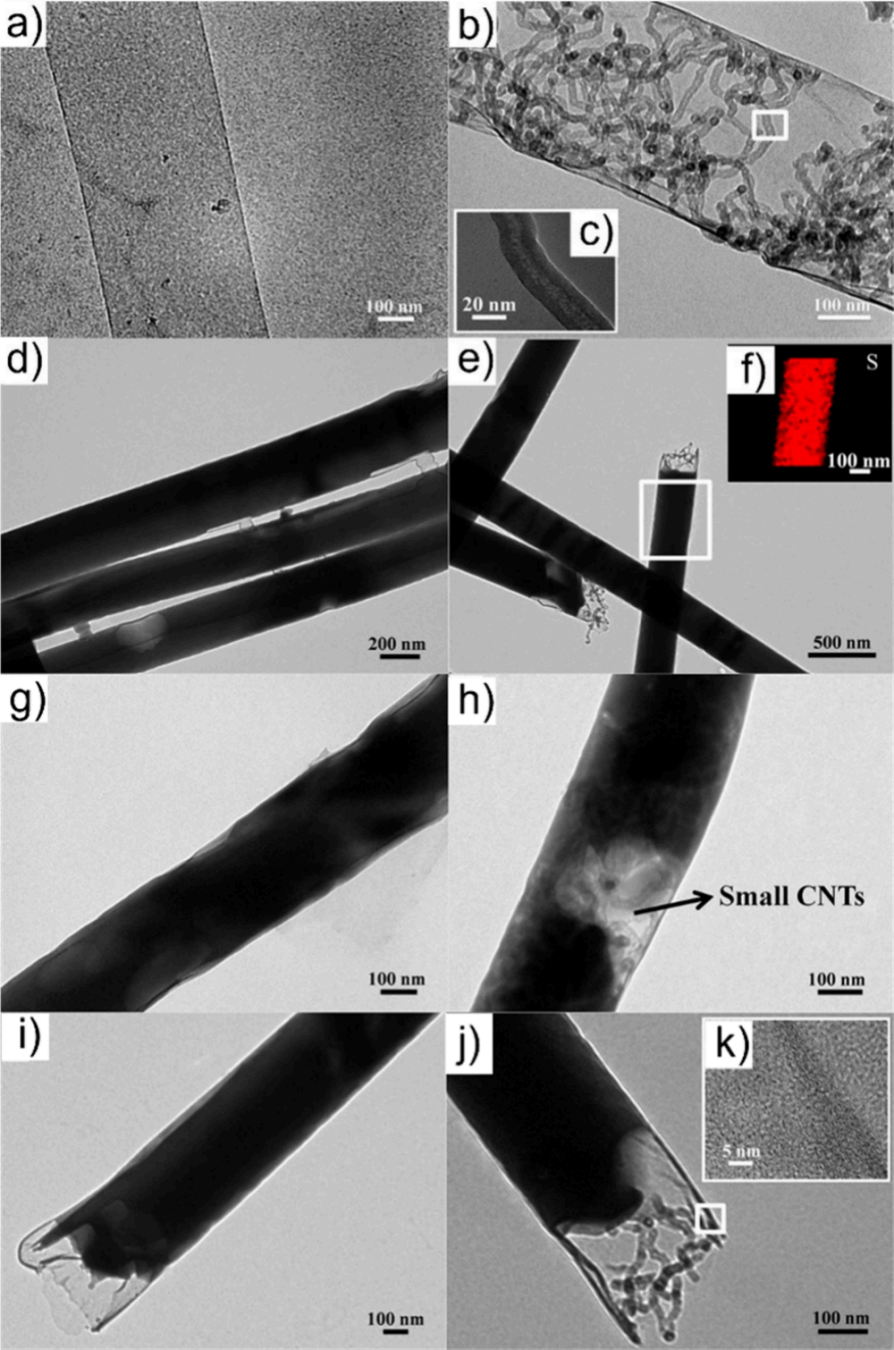
Large diameter CNTs have been filled with small-diameter CNTs and a high amount of S to prepare alternative S based electrodes. TEM images of various products: a) bare CNT, b) CNTs@CNT, c) filled small-diameter CNT, d) S@CNT, and e) S-CNTs@ CNT. f) Elemental mapping image of encapsulated S. TEM images showing the body sections: g) S@CNT and h) S-CNTs@CNT. TEM images showing the tip sections: i) S@CNT and j) S-CNTs@CNT. k) HRTEM image showing the large-diameter CNT overlayer.^792^ Reproduced with permission from ref 792. Copyright 2016, American Chemical Society.

More recently, a highly conductive interconnection framework consisting of on Co-filled N-doped CNTs grown on the surface of reduced graphene oxide was proposed as an enhanced performance cathode for lithium–sulfur batteries.^321^ This hybrid favors both electron transport and the retention of polysulfide species, whose redox kinetics are accelerated by the presence of Co NPs, thus leading to a capacity of 819.5 mAh g^–1^, maintained even after 500 cycles.

CNTs filled with Pd NPs, prepared by solution filling, have been reported as cathode materials for LIBs.^793^ The authors compared the cycling stability of CNTs filled or coated with Pd NPs. A significant improvement of 35% in the discharge cycling performance was observed for Pd@CNTs compared with Pd-coated CNTs at a limited capacity of 500 mAh g^–1^ and a current density of 250 mA g^–1^. This difference was attributed to the enhanced stability of the electrolyte when encapsulated inside the CNTs, which prevented the formation of Li_2_CO_3_.

### 8.3 Gas Storage and Separation

The high concentration of atmospheric contaminants and the continuous research to develop new sustainable energy sources, alternative to fossil fuels, have increased the demand for the fabrication of new solid systems able to efficiently capture and store gas molecules. Gas capture finds application in other practical areas including catalysis, gas separation, sensing or water purification.

Owing to their morphological and chemical features, CNTs have emerged as promising candidates in this field. While their uniform hollow cavity and high surface area confer CNTs a high potential for the diffusion and accumulation of a large variety of gases,^794^ the conjugated structure of their walls can be functionalized either to improve the gas capture mechanism or to increase the interaction with the encapsulated molecules, therefore stabilizing the emerging system. When chiral CNTs are employed, the selective encapsulation of specific gas molecules can be induced, the material being therefore ideal to be used in separation processes.^60^

Gas molecules are confined within the CNTs’ cavity via adsorption mechanisms, which may include physical (physisorption) or chemical (chemisorption) interactions between the host surface and the encapsulated entities. In order to thermodynamically understand the confinement of gas molecules within the cavity of SWCNTs, Bahmanzadgan et al. simulated the adsorption of different gas molecules using Monte Carlo and DFT calculations.^60^ The analysis of the physisorption behavior of O_2_, N_2_, CO_2_, H_2_ and CH_4_ molecules on SWCNT (14,14) surfaces indicated that negligible interactions between the host wall and the adsorbate occur at large separations. When the molecules approach to the CNT, vdW forces induce an energy fall that stabilizes the system. However, physisorption is affected by short-range repulsion forces (breaking the equilibrium) that are less pronounced in electrophilic molecules, due to their stabilization by the interaction with the *sp* ^*2*^ cloud of the conjugated honeycomb structure of the CNT walls.

The adsorption process is exothermic, and is favored by the increase of pressure and widely influenced by the tube diameter and electronic configuration of the encapsulated molecule. Calculations revealed that lower heat is released as molecules are adsorbed within SWCNTs with larger diameters (isosteric heat), as consequence of the weaker vdW forces between the host and the adsorbate. The authors corroborated that the isosteric heats and energy-distribution profiles were different when both O_2_ and N_2_ were confined.^60^ This is particularly useful and pave the way toward the development of functional systems that enable the controlled and highly selective separation of different species in their gas phase.

#### 8.3.1 Hydrogen Storage

Over the past few years, most studies on CNTs for gas storage have focused on understanding their ability to adsorb hydrogen, which is significantly relevant for the development of new materials for energy applications.^795^ A large number of reports consist of theoretical studies that have analyzed the influence of the conditions and the variety of hosting CNTs as suitable storage entities.^60,107,136,796–800^ According to previous studies, H_2_ molecules can approach the surface of CNTs parallel to the C–C bond, perpendicular or parallel to the C ring, being able to bind either the outer or the inner surface of the tubular structure, or occupying the spaces between bundles. The adsorption of H_2_ molecules is greatly ruled by the functionalities attached to the CNTs surface, the curvature and hence by the diameter of the CNTs. Parameters such as *T* and intertube spacing also play a role in the adsorption capacity of the hosting tubes.^801^

The H_2_ adsorption mechanism involves both physisorption and chemisorption processes, which contribution depends on the morphology, purity, crystallinity or degree of modification of the CNTs. When H_2_ is physically adsorbed, the *T* of the system and the pressure of the flowing H_2_ greatly affect the formation of vdW interactions between the diatomic molecules and the walls of the host.^795^ In case of chemisorption, the formation of covalent bonds (hydrogenation) is chirality and diameter dependent, being favored in case of thinner CNTs and armchair systems.^796^ Two studies, reported in 1997 showed, for the first time, the ability of SWCNTs to store considerable quantities of hydrogen.^679,802,803^ Two years later, a theoretical study based on DFT was performed to estimate the hydrogen adsorption capacity of SWCNTs. The authors considered two important experimental parameters: the different operating pressure and *T* regimes. Although the theoretical analysis anticipated a very favorable adsorption of hydrogen by SWCNTs, gravimetric storage densities of SWCNTs revealed a much lower hydrogen storage capacity than the values previously reported in the literature.^96^ Indeed, the theoretical and experimental results reported over the years for hydrogen storage capacity of CNTs are controversial. Several experimental parameters, such as the high morphological diversity of CNTs, the presence of contaminants (catalyst NPs and amorphous carbon), the nature of the interaction between hydrogen and the host nanomaterials, and several possible regimes of pressure and *T*, can contribute significantly to the disparity observed between theoretical and experimental results.^804^

In 2007, Nikitin et al. studied the influence of the SWCNTs diameter on hydrogen chemisorption. The authors reported that the diameter values of SWCNTs around 2.0 nm maximize the formation of CNT-hydrogen complexes, with hydrogen storage capacity that reaches more than 7 wt % and showing high stability at RT.^805^ Another preponderant factor that can contribute to the discrepancy observed between molecular simulations and experimental results for hydrogen storage in CNTs is the formation of microstructures. The characteristic ability of CNTs to form bundles allows the possibility to obtain 3D structures with high interstitial nanoporosity, which is energetically favorable for hydrogen storage.^806^ Assfour et al. studied CNTs with three and four different orientations of tube axes (Figure 35), which showed outstanding hydrogen storage ability on their external walls, with total uptake amounts up to 19.0 wt % at 77 K and 5.5 wt % at 300 K.^807^

**Figure 35:**
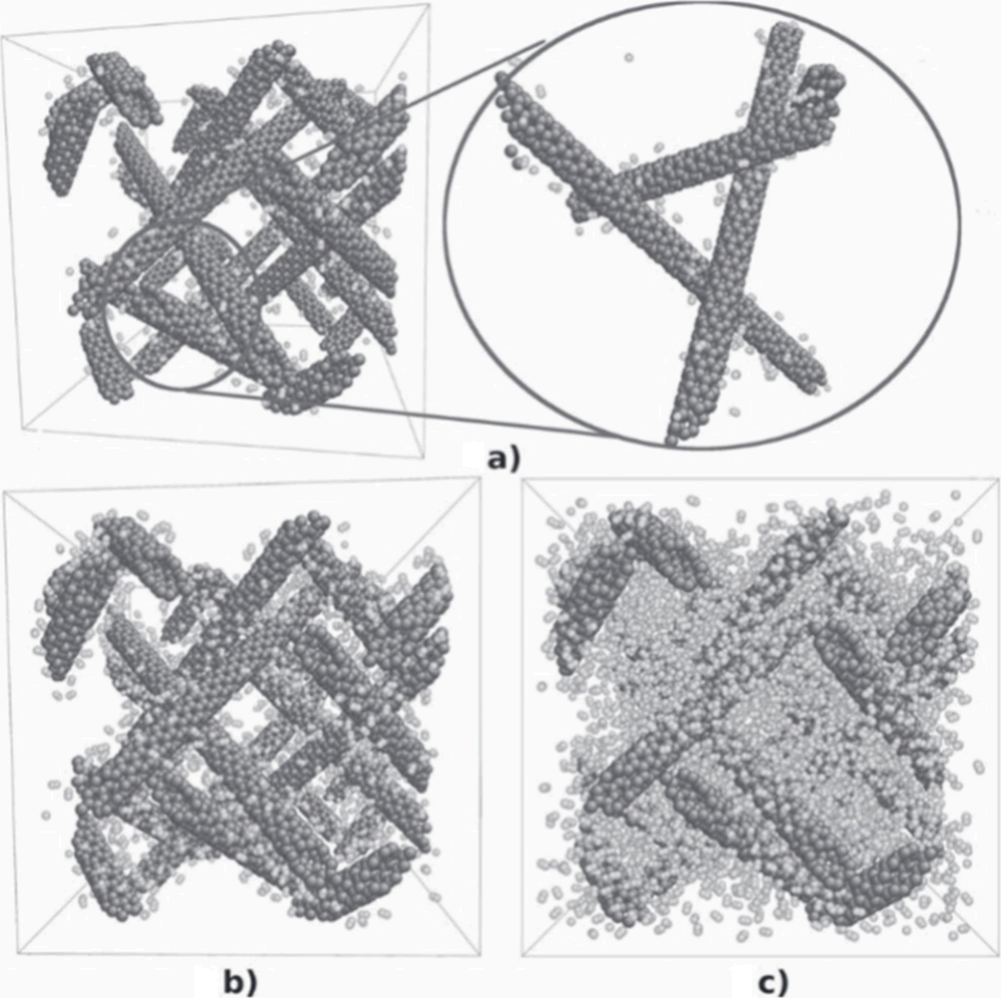
Ability to store H_2_ within packed CNTs. + Σ packing of (6,0) tubes with H_2_ molecules adsorbed at a) 0.1 bar, b) 1.0 bar and c) 100 bar.^807^ Reproduced with permission from ref 807. Copyright 2011, John Wiley and Sons.

The development of CNT hybrids with catalytic metal NPs for the dissociation of H_2_ into atomic H has demonstrated to improve their hydrogen storage capacity. Reports include decoration of MWCNTs with Mg,^808^ Al,^797^ Pt,^809^ Y,^799,800^ Ca, Co, Fe, Ni and Pd NPs.^810,811^ the latter demonstrating the highest contribution to retain H_2_ molecules. Yoo et al. proposed the synthesis of CNTs/Pd for improved hydrogen storage applications. Furthermore, they induced the formation of structural defects in the CNTs by an oxidative process with a lanthanum catalyst to form more anchoring sites for hydrogen. However, in the best-case scenario (1 atm and 573 K), only 1.0 wt % of hydrogen was stored by the prepared hybrid materials.^812^ More recently, Vellingiri et al. studied the H_2_ adsorption/desorption regime of SnO_2_-functionalized MWCNT hybrids that demonstrated high hydrogen storage capacity, reaching up to 2.63 wt % of hydrogenation in only 30 min. Raman spectra of the hydrogenated samples (treated under 5 bar H_2_ at 100 °C) confirmed that SnO_2_ present on the MWCNTs surface favored dissociation of H_2_ molecules that were subsequently adsorbed onto the defect sites of the CNTs walls.^813^ Other examples include CNTs modified with ZnO,^814,815^ Ni,^816^ Pd,^817^ Mg,^803^ or the hexagonal h-BN system.^818^

Doping the structure of CNTs with heteroatoms is another strategy that has been explored to improve their hydrogen storage capacity. Theoretical calculations showed that the incorporation of N, P, S, and B into the carbon network can act as activation centers for highly efficient hydrogenation of CNTs.^819^ Recently, it was shown that the doping of carbon-ring-based molecular complexes with alkali metal ions (Li^+^, Na^+^, and K^+^) can enhance the storage capacity up to 11.21–13.95 and 10.42–13.24 wt %, respectively, thus providing an interesting approach for the use of CNTs as molecular templates for hydrogen storage.^795,820–822^ MWCNTs encapsulating Ni crystals were also proposed as suitable materials for hydrogen uptake.^823^ At moderate conditions of *T*, the system showed hydrogen storage capacity of 0.298 wt %, which was directly proportional to the H_2_ pressure.

Hydrogen production from natural gas is one of the most promising alternatives for clean energy production. However, one of the main drawbacks of this approach is the presence of other gas-phase molecules such as CO and CH_4_. Using first-principle DFT, Cheng et al. demonstrated the potential ability of porous CNTs for the selective adsorption of hydrogen to GHGs, namely, O_2_, CO_2_, CO, N_2_ and CH_4_.^136^

#### 8.3.2 Methane Storage

Owing to a superior thermal efficiency and lower production of GHGs, natural gas constitutes a cleaner alternative to other fuels to produce energy.^824^ The advantage of CNTs as nanovessels for natural gas storage lies in their ability to confine a large amount of CH_4_ molecules under moderate conditions, compared with similar tubular platforms, such as BN,^95^ or other sequestration approaches currently available, such as liquefaction.^825^

Simulations performed according to the armchair arrangement (m, m) of SWCNTs for CH_4_ adsorption indicate that the (15, 15) SWCNT with a vdW gap of Δ = 0.8 nm was the optimal adsorbent at RT.^826^ It was reported that under supercritical conditions, CH_4_ diffusion within SWCNTs can be improved.^827^ The authors observed that at supercritical *T*, the diffusion coefficient at high pressure reaches a plateau independently of the CNT size. Furthermore, they found that for SWCNTs with diameters larger than 2 nm, capillary condensation occurs when the *T* is sufficiently low, followed by layer-by-layer adsorption of gas molecules on the CNT surface.

Structural modifications of the CNTs walls also contribute to improve their adsorption capacity.^828^ DFT studies showed that decoration of SWCNTs with Ca alters the CH_4_ adsorption regime, due to the active site role adopted by the added metal impurities.^134^ More recently, post-synthesis treatments of pristine SWCNTs using HNO_3_ enabled increasing the specific surface area and pore volume of the CNTs, significantly increasing the adsorbate capture. At low *T* and pressures between 0 and 4 MPa, the adsorption capacity of the modified CNTs reached values as twice as the CH_4_ uptake of the pristine sample (26.15 mg g^–1^ and 13.62 mg g^–1^, respectively).^828^

#### 8.3.3 CO_2_ Capture

CO_2_ is the main product resulting of combustion of fossil fuels. Along with other GHGs it contributes drastically to global warming, negatively affecting the environment. A variety of reports describe the advantages of using CNTs for the selective capture and sequestration of CO_2_.^829,830^ In 2003, Cinke et al. performed both experimental and theoretical studies to understand the adsorption process of CO_2_ within SWCNTs.^831^ The authors observed that increasing the *T* of the system decreased the adsorptive capacity of the CNTs, which suggested that the creation of intermolecular physical interactions rule the adsorption process. Most publications register pretreatments to modify the surface and reactivity of the adsorbers, in order to improve their ability to physisorb CO_2_ molecules. Examples include the formation of mixed matrix membranes integrating CNTs with poly­(ether sulfone) for CO_2_ removal from biogas,^832^ modification of MWCNTs with 3-aminopropyl-triethoxysilane (APTS),^830,833,834^ or enhancing the fixation of these molecules at ambient pressure by grafting MWCNTs with chitosan.^835^ Owing to their affinity with the electrophilic C of the CO_2_ molecule, modification with N groups significantly enhances the ability of CNTs to retain the adsorbate.^836,837^ Particular attention has been paid to amino-modified CNTs.^834,838,839^ As consequence of the strong N–C interaction, the CO_2_ adsorption–desorption process in the presence of N-doped CNTs was greatly modified, undergoing up to 503% increase of the desorption rate compared with the unmodified host.^836^

#### 8.3.4 Other Gases

In 1997, a methodology for the efficient storage of Ar within the internal cavities of CNTs with diameters ranging from 20 to 150 nm was reported. The protocol consisted in isostatic pressing of the carbon nanomaterial under Ar at 170 MPa. The procedure, which lasted 48 h, also involved annealing of the system at 650 °C.^840^ X-ray mapping analysis confirmed the preferential storage of Ar (red colored areas) within the inner cavity instead of external superficial adsorption (Figure 36 b), d), e)). After this discovery, which dates from the preliminary tests performed with H_2_, many other studies were conducted in order to investigate the ability of CNTs to store a wide range of gases,^841^ including NO_2_,^97,842–846^ SO_2_,^105^ H_2_S,^98,105^ O_2_,^97,841^ NH_3_,^97,843^ N_2_,^97,101,841^ H_2_O,^97^ Ar,^840^ Kr,^689^ and Xe.^106,806,847,848^

**Figure 36:**
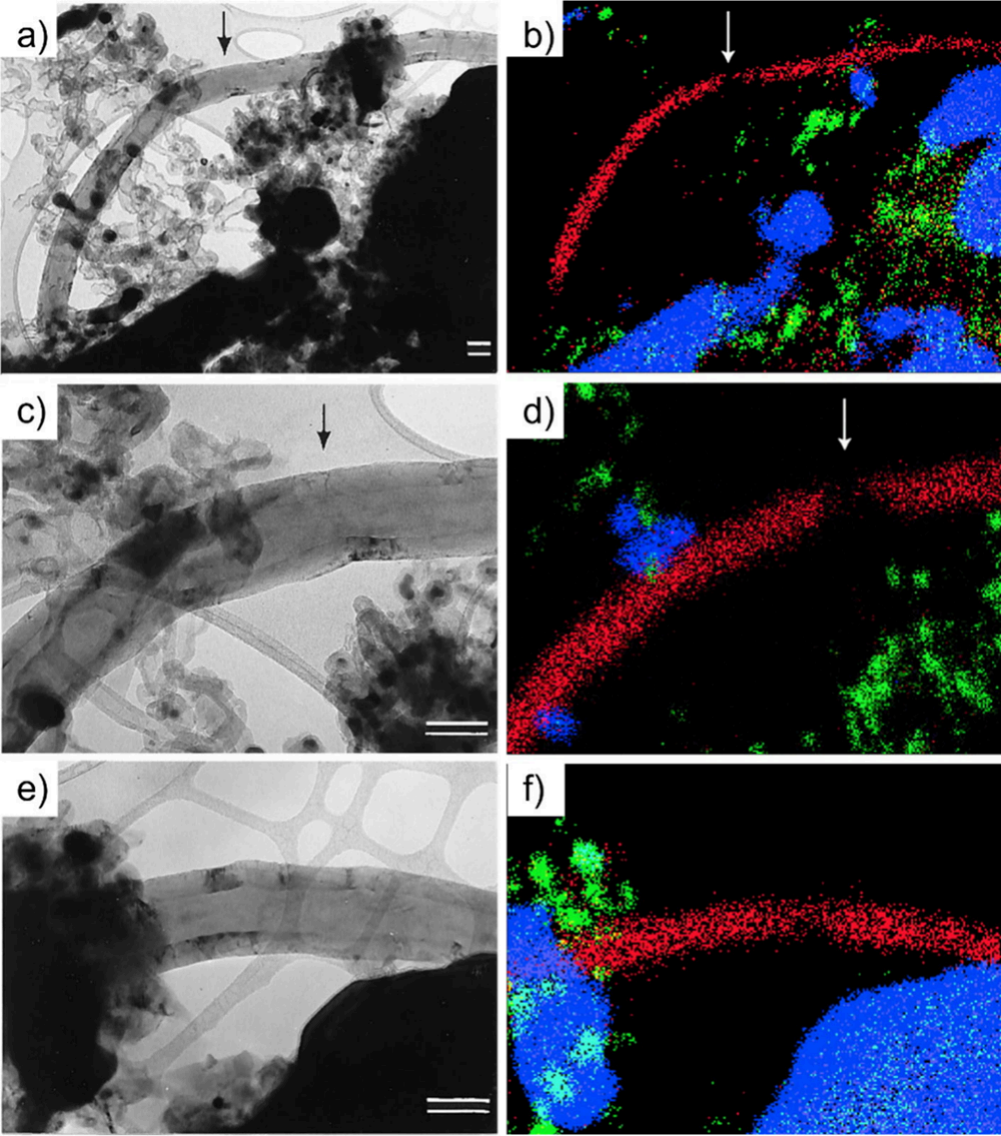
TEM images and X-ray maps of a typical Ar-filled CNT that winds its way through dense material. The C tube was clearly hollow, with an outer diameter of 140 nm and an inner bore diameter of 60 nm. a), c), and e) show TEM images, whereas b), d), and f) display the corresponding X-ray map images. In the latter panels, the Kα X-ray emissions from Ar, Fe, and Zn are shown in red, green, and blue, respectively. The arrows in the images indicate discontinuities in the tubes. The scale bars on a), c), and e) represent 100 nm.^840^ Reproduced with permission from ref 840. Copyright 1997, The American Association for the Advancement of Science.

Fujiwara et al. reported the influence of the structural features of SWCNTs on the adsorption of N_2_ and O_2_ gases. When closed-ended SWCNTs were employed, both gases were adsorbed only at the interstitial sites between the packed CNTs.^841^ However, open-ended SWCNTs underwent an initial adsorption of gases into the inner cavity of SWCNTs and posteriorly on the external interstitial sites. Indeed, some controversies remain regarding the preferential sites for the adsorption of gases by SWCNTs, as discussed earlier for hydrogen. Some authors reported that adsorption of NO_2_, O_2_, NH_3_, N_2_, CO_2_, CH_4_, H_2_O, Ar, and Xe occurs preferentially on the SWCNTs bundle interstitial and groove sites, instead of into individual CNTs.^97,806,847^

The adsorption results cannot be simply extrapolated to all types of CNTs, since their adsorption ability is directly linked to their electronic structure and the type of gas under study.^806^ Recently, theoretical calculations with an analytical model and DFT were used to predict the optimal types of CNTs that can encapsulate each of the noble gas atoms (He, Ne, Ar, Kr, and Xe).^135^ The study showed that CNTs with radii ranging between 2.98–4.20 Å (chiral indices (5,4), (6,4), (9,1), (6,6), and (9,3)) can efficiently encapsulate He, Ne, Ar, Kr, and Xe, respectively. Furthermore, preferential endohedral adsorption of all noble gases on the different types of CNTs over exohedral adsorption was concluded.

CNTs can be chemically modified to increase their adsorption capacities. Gosh et al. synthesized metal-filled CNTs with enhanced CO_2_ adsorption compared to their non-filled counterparts.^849^ The adsorption capacity depended on the material previously encapsulated within the CNTs’ cavities and was significantly improved by nitrogen-doping in the following order: Fe/Fe_3_C@NCNTs > Co@NCNTs > Ni@NCNTs.

#### 8.3.5 Gas Separation

Soon after the initial reports on CNTs, their potential for gas separation was considered. The encapsulation of foreign species within the CNT’s cavity involves a close surface interaction, so understanding the processes involved in the gas capture and separation is crucial to take advantage of the ability of the CNTs to confine or restrict the insertion of specific molecules. Enhanced mechanisms have been reported when using CNTs to separate gases, demonstrating improved adsorption capacities against other capture agents, such as activated carbons or zeolites, under the same conditions.^850^ In terms of dimensionality, CNTs can be used for size exclusion, acting as molecular sieves, enabling the smooth passage of small molecules through their channels, while species larger than the inner CNT diameter remain external to the cavities. Once confined, the passage rate of the molecules also varies in function of their size, structure, and shape, therefore favoring the separation. Although the selectivity of CNTs toward certain gas molecules can be controlled by tuning the dimensions of the adsorbers, other aspects such as the physicochemical properties of the CNTs, the interaction between the molecules that constitute the gas mixtures, and the molecular density should also be considered. CNTs offer low-resistance pathway for diatomic molecules and are able to establish both physical and chemical interactions with the adsorbates, thus enabling selective adsorption/desorption equilibria with a variety of gas molecules. Ideally, pristine CNTs have a smooth interior surface that minimizes the drag and friction, allowing the ultrafast transport of gas molecules. Their surface can be modified either by functionalization, chemical, or electrical doping to improve these interactions, consequently facilitating the selective transport of molecules through the cavities. In this way, strong chemical or physical affinity with selected species, such as CO_2_ over other entities (CH_4_ or N_2_) can occur.^851^

Most studies on gas separation using CNTs are theoretical and describe the approaching, surface adsorption, and transport mechanisms of gas mixtures within different types of CNTs. These consider dimension, surface functionalization and conditions of reaction to predict how the diverse chemical and physical interactions established between the CNTs’ surface and foreign gas molecules induce the selective capture and retention of certain species. Studies have been mainly focused on the encapsulation and separation of noble gases, such as Xe/Kr,^104^ the isolation of CO_2_ from CH_4_,^103,852^ H_2_,^853^ N_2,_ ^118,854^ H_2_O,^855,856^ or the separation of CH_4_/H_2_,^853^ H_2_O/O_2_,^856^ and H_2_O/H_2_ mixtures.

Already in 1995, Takaba et al. carried out MD simulation studies to evaluate the possibility to use CNTs as molecular sieves, selectively adsorb and separate benzene, alkylated benzenes and alkylated naphthalenes. The authors highlighted the flexibility of the CNTs to encapsulate isomers with slight size differences, such as, *p*-, *m*-, and *o*-xylene. During the incorporation of these molecules, CNTs underwent different pore width variations (up to 0.15 nm for the larger molecule, *o*-xylene), which suggests that even if they are thought to be small, steric and binding configurations might play a role in the guest–host interaction and molecules diffusion inside the cavities. This was the case of 2,6-dimethylnaphtalene in which the trans-arrangement of the methyl group facilitated the uptake of the molecule into the CNTs, in contrast with its *cis*-arranged counterpart (2,7-dimethilnaphtalene), which demonstrated a prohibited probability to enter into the host owing to the colliding of the −CH_3_ group with the CNT wall.^121^ In a further theoretical work, the diffusion of three binary molecular mixtures, namely, methane/*n*-butane, methane/isobutane, and methane/ethane inside SWCNTs with diameters ranged between 0.80 and 1.65 nm was analyzed using classical MD simulations.^857^ The authors calculated parameters such as flux, density profile, and diffusion coefficient/mobility of molecules located near open-ended SWCNTs. As expected, the adsorption and diffusion of the molecules was largely dependent on the investigated diameters. The entrance of *n*-butane and isobutane into 0.7–1.1 nm SWCNTs was blocked owing to their dimensions, while these molecules, along with methane were able to diffuse into larger tubular systems. Since methane diffused alone inside the smaller CNTs, a careful diameter selection can be used to allow the separation of both methane/*n*-butane and methane/isobutane mixtures. Otherwise, despite 1.1–1.5 nm SWCNTs, allowed the incorporation of *n*-butane and isobutane molecules, their contact with the host was restricted by the available space and curvature of the walls. Finally, in case of 1.5–2.3 nm CNTs, the incorporation and migration of the binary mixtures through the CNTs’ cavities strongly depended on the interaction of the molecules with the CNT inner walls. This interaction was also affected by their geometry, with significant effects in the adsorption energies of the individual molecules. The linearity of *n*-butane, for instance, favored its approach and location close to wider CNT walls, in contrast with the bulkier isobutane molecule which stood in the center of the cavity.

Zhou et al. used DFT, GCM and MD simulations to evaluate the suitability of Li doped CNTs for the ultrahighly efficient separation of CO_2_ and N_2_.^858^ While inside CNTs Li atoms diffuse rapidly and reach their more stable configuration when located at the bridge site p along the CNT axis (Figure 37), the proposed system showed a higher selectivity toward the CO_2_ uptake against its undoped counterpart, as a function of Li intercalation concentration, reaching CO_2_/N_2_ ratios up to 5 at ambient pressure. Moreover, the presence of Li dopant does not alter the arrangement of gas molecules within the CNTs’ cavities and significantly decreases the influence of the host diameter in gas separation.

**Figure 37:**
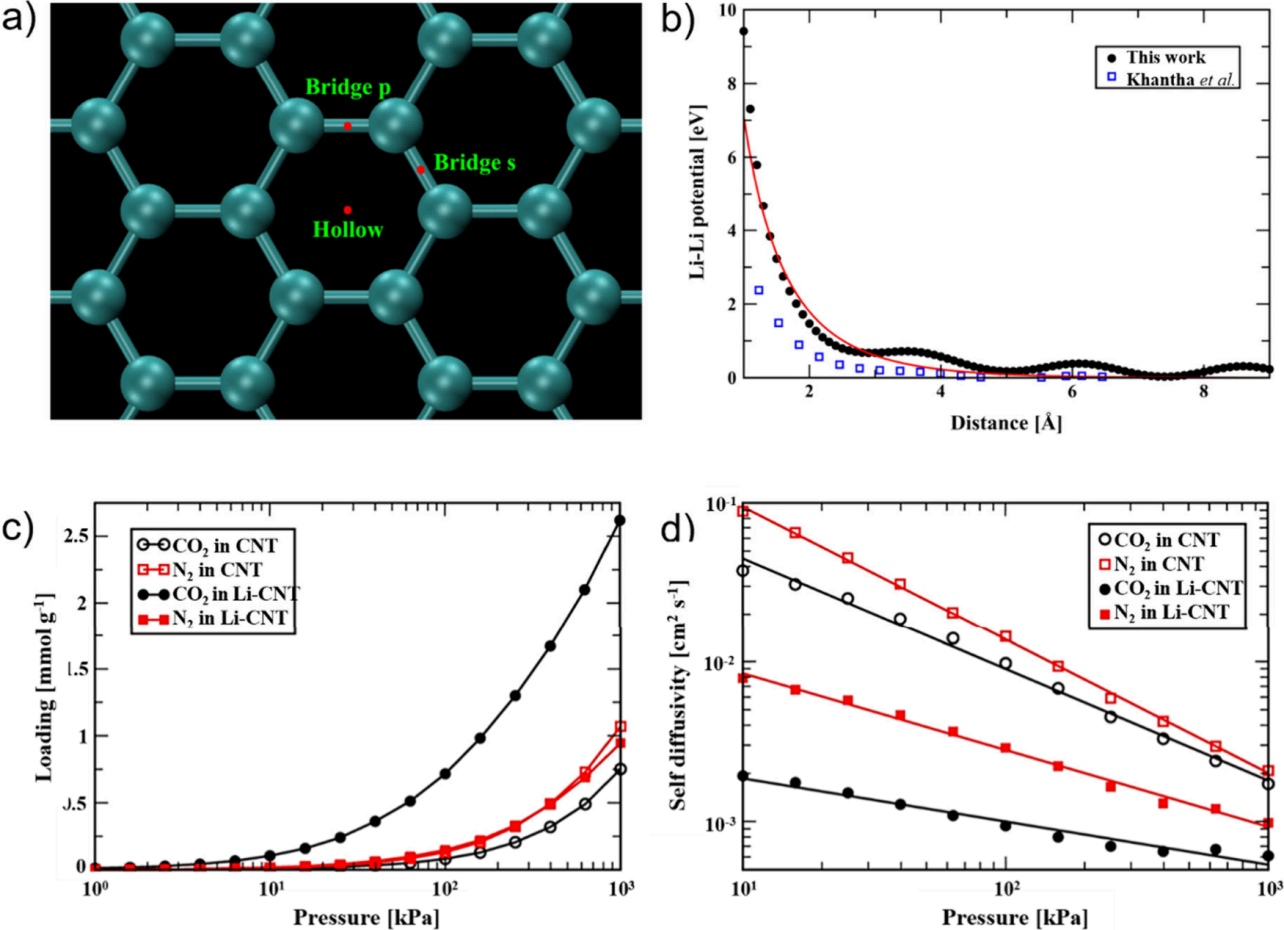
Theoretical calculations suggested that Li dopants contribute to the efficient separation of CO_2_ and N_2_. a) Li atoms reached their more stable configuration when located at the bridge site p. b) The binding energy of Li atoms above two different bridge sites of (8, 8) CNT.^858^ The authors compared results with calculation data from Khantha et al.^859^ Reproduced with permission from ref 858. Copyright 2015, American Chemical Society.

In case of more complex molecules, their size difference plays an important role in the separation. Monemtabary et al. studied the thermodynamics of the adsorption and the ability of MWCNTs to separate CH_4_ molecules from other gas species such as H_2_ and CO_2_ present in syngas.^860^ In agreement with theoretical studies,^117,120^ while high pressures favored the capture and sequestration of the guest, low *T* improved the adsorption capacity of the CNTs (up to 5.44 mmol g^–1^ of CH_4_ at *T* = 283.15 K and P = 40 bar). The employed tubular hosts were able to establish stronger intermolecular interactions and therefore encapsulate a superior amount of CH_4_ compared with H_2_.^824,860^

In a recent study, Nashed et al. investigated the use of MWCNTs in CO_2_ separation from natural gas.^861^ This is particularly relevant since the increasing demand for natural gas has stemmed the interest in alternative sources, often rich in other gases such as CO_2_, with limited benefits owing to corrosion issues or environmental concerns. The hydrate-based separation method takes advantage of the formation of stable gas hydrates, which consist of hydrogen-bonded water cages, to selectively accommodate gas molecules. The formation of solid clathrate crystals of one gas under controlled pressure and *T* conditions enhances the molecular separation. The crystallized gas can then be recovered by a posterior dissociation. In this work, the authors demonstrated the advantages of incorporating CNTs during the formation of gas hydrates in CH_4_/CO_2_ mixtures, highlighting their ability to accelerate the kinetics of the process. Pristine, hydroxylated (OH-MWCNTs) and carboxylated (COOH-MWCNTs) CNTs were tested to separate 70:30 CH_4_/CO_2_ mixtures, the latter acting as more efficient kinetic promoters, which provided a CO_2_ separation factor of 2.9–3.4. Up to 65% CO_2_ was further recovered.

CNTs emerged as promising platforms that are usually integrated within polymeric systems to form mix matrix membranes (MMMs) for their further application in natural gas upgrading, air separation, hydrogen purification and storage, selective gas sensing, biogas upgrading or GHGs capture. The main challenge of using polymeric systems for gas separation consists in the decrease of selectivity as the rate of gas flow increases, and it is described by means of the Robeson Upper Bound (RUB) plot, usually employed to describe the performance limit of these membranes. Owing to their higher selectivity and permeability,^119^ compared to the diffusive transport in the polymeric matrix, incorporating CNTs enables overcoming this issue.

CNTs provide the system an increased thermal, and chemical stabilities, and enhanced mechanical strength, thus reducing plasticization and increasing the cycle of life of the membrane.^862^ The dispersibility of the CNTs filler within the polymeric matrices is usually enhanced by both covalent and non-covalent functionalization of the tubular nanostructures, which can also improve the adsorption of the adsorbate within the composite.^863^ MMMs have been prepared by embedding CNTs within different matrices, including MOF,^864^ polyimide,^865^ and poly­(ether-*block*-amide).^866,867^ By incorporating cyclodextrin modified MWCNTs within a polyimide membrane, Sanip et al. were able to enhance the selectivity toward CO_2_/CH_4_ gas up to 100% compared with the neat polymer membrane, significantly reducing the energy consumed during the process.^865^ More recently, a MMM consisting of amino-functionalized CNTs embedded within PDMS demonstrated enhanced efficiency for ethanol recovery from cellulose-based biomass. Before the integration within the polymeric matrix, the NH_2_-MWCNTs were modified by coupling the amino group with 3-glycidyloxypropyl trimethoxy (KH560) through a ring-opening reaction (K-MWCNTs). The attached linker provided the MMM higher hydrophobicity, therefore reducing the adsorption of water and improving the selectivity toward C_2_H_5_OH molecules during the pervaporation process.^868^ As consequence of the incorporation of the K-MWCNTs, the MMM presented superior performance (separation factor of 10.4) with permeate flux of 982.1 g m^–2^ h^–1^ compared with pure PDMS, which reached a separation factor of 9.1 and a permeate flux of 652.7 g m^–2^ h^–1^. Relevant studies carried out on the application of CNTs in gas capture, storage and separation are included in Table 10.

**Table 10:** Relevant Studies Performed to Evaluate the Ability of CNTs to Act as Molecular Sieves for Gas Storage and Separation^a^

Gas	Hosting platform	Application	Adsorption mechanism	References
**H**_**2**_	• SWCNTs.	• Fuel Cells.	• Physisorption: Weak vdW forces.	^101^,^679^,^802^,^805^,^809–814^,^816–818^,^821^,^823^,^869^
	• Pt, Pd, Ca, Co, Fe, Ni NPs supported MWCNTs.	• Gas separation.	• Chemisorption: On functionalized or doped CNTs.	
	• SnO_2_, ZnO, and hBN-modified MWCNTs.			
	• Ni, B, N and alkali-doped MWCNTs.			
**CH**_**4**_	N-doped SWCNTs.	Natural gas storage.	Physisorption: vdW forces.	^683–685^,^825^,^828^,^860^,^870^
**CF**_**4**_	SWCNTs.	Greenhouse gas capture.	Physisorption: vdW forces.	^686^
**CO**_**2**_	SWCNTs.	Carbon dioxide capture.	Physisorption: vdW forces.	^101^,^680–682^,^831^,^830^,^836^,^838^,^871^
	• N–O-codoped SWCNTs.			
**NH**_**3**_	SWCNTs.	• Gas sensing.	Chemisorption & Physisorption.	^843^
		• Gas storage.		
**NO**_**2**_	• SWCNTs, DWCNTs and MWCNTs.	• Gas sensing.	Chemisorption: Electron transfer.	^843^,^844^,^872^
	• C dots-decorated SWCNTs.	• Environmental monitoring.		
**CO**	SWCNTs.	• Gas sensing.	Chemisorption & Physisorption.	^101^
		• Environmental monitoring.		
**C**_**2**_ **H**_**5**_ **OH**	NH_2_-functionalized MWCNTs.	Gas separation.	Physisorption: vdW forces.	^868^
**Noble gases**	SWCNTs.	Gas separation.	Physisorption: vdW forces.	^688^,^689^,^840^
**CH**_**4**_ **/H**_**2**_	• SWCNTs.	Pure H_2_ production from fuel cells and other industrial processes.	Physisorption.	^853^,^873^,^874^
	• Functionalized MWCNTs.			
	• Vertically aligned CNTs.			
**CO**_**2**_ **/CH**_**4**_	Pristine SWCNTs and MWCNTs. Functionalized-MWCNTs	Natural gas purification.	Physisorption: vdW forces.	^103^,^852^,^865–867^
**CO**_**2**_ **/H**_**2**_	• SWCNTs.	CO_2_ capture, pure H_2_ production.	Physisorption: vdW forces.	^875^,^876^
	• Pd-modified MWCNTs.			
**CO**_**2**_ **/N**_**2**_	• N, O, or NH2-functionalized MWCNTs and SWCNTs.	Removal of CO_2_ from flue gas.	Physisorption: vdW forces.	^118^,^866^,^867^,^877^
	• Vertically aligned CNTs.		Chemical/Electrostatic selectivity.	
**H**_**2**_ **O/CO**_**2**_	Vertically aligned CNTs.	Natural gas dehydration.	• Physisorption: vdW forces.	^855^,^856^,^873^
			• Diffusivity/Hydrophobicity (Ultra-Fast Transport).	
**H**_**2**_ **O/O**_**2**_	Vertically aligned CNTs.	• Dehumidification.	• Physisorption: vdW forces.	^856^
		• Gas purification.	• Diffusivity/Hydrophobicity (Ultra-Fast Transport).	
**N**_**2**_ **/O**_**2**_	SWCNTs.	Air separation.	Physisorption.	^60^
**H**_**2**_ **/N**_**2**_	Pd-modified MWCNTs.	Pure H_2_ production.	Physisorption.	^876^
**Xe/Kr**	SWCNTs.	Recovery of noble gases from nuclear fuel reprocessing off-gases.	• Physisorption and vdW force difference. Polarizability differences favor the separation.	^104^

a The table includes the main gases stored within CNTs along with the application and adsorption mechanism.

### 8.4 Nanoreactors

The nanoconfinement of chemical reactions of individual molecules and atoms can provide a fundamental understanding of reaction pathways and consequently allow fine control over the final products. Several nanocontainers have been explored over the years, including metal–organic frameworks, protein pores and channels, CNTs, microporous silica, zeolites, dendrimers, and polymer templates, to manipulate molecules and ions with a level of control unattainable in bulk environments. The review entitled “*Nanoreactors: Small Spaces, Big Implications in Chemistry”* describes some very interesting examples of the use of nanoreactors for modulation of chemical reactions.^878^ CNTs have been the subject of particular interest for their use as nanoreactors due to their intrinsic properties and structural features. Their network of *sp* ^*2*^-carbon atoms provides significant advantages over other molecular or supramolecular nanocontainers, such as high chemical, EC, and thermal stability (up to 2800 °C in vacuum) and mechanical robustness (tensile strength higher than steel). Furthermore, CNTs can offer diverse electrical properties (semiconductors/conductors) and a wide range of inner diameters (from a few to tens of nm). Indeed, it has been clearly shown that the performance of reactions within the cavities of CNTs can drastically change the behavior of molecules or ions during chemical reactions, thus affecting the final products of these reactions.

The application of CNTs as nanoreactors started a few years later, after the demonstration of their ability to encapsulate C_60_ (formation of nanopeapods) in 1998.^146^ In 2000, Luzzi et al. reported for the first time the ability of fullerene guest molecules to oligomerize, coalesce, and merge into corrugated tubular structures inside the hosting SWCNTs by thermal treatment or electron beam.^173^ Additionally, there was evidence that fullerenes, treated under similar experimental conditions outside CNTs, were not able to form oligomeric and tubular structures. A wide variety of unusual molecular structures has been synthesized using different derivatives of C_60_ as monomers confined inside SWCNT nanocontainers (Figure 38).^879^

**Figure 38:**
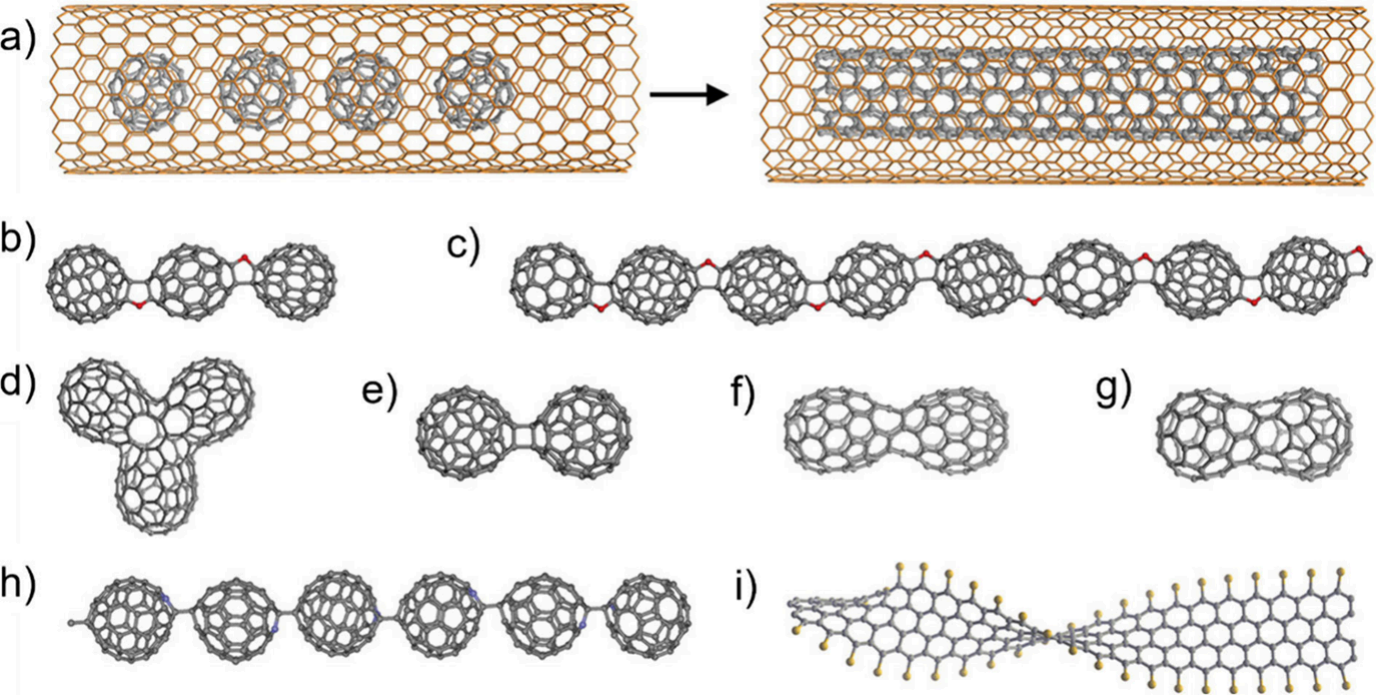
A variety of unusual molecular nanostructures can be synthesized inside SWCNTs. a) C_60_ molecules transform into an internal SWCNT; b) oligomers; c) polymers (C_60_O)_*n*_; d) “trefoil”; e–g) variety of dimer molecules formed from three or two fullerene cages, respectively; h) polymer (C_59_N)_*n*_; i) sulfur-terminated graphene nanoribbons.^879^ Reproduced with permission from ref 879. Copyright 2011, American Chemical Society.

Several other examples of the application of CNTs as nanoreactors have also been reported. Annealing or e-beam exposure of coronene-filled SWCNTs, with diameters close to the size of the guest molecule, results in their polymerization, forming internal CNTs.^880,881^ It was further observed that the same experimental treatment in coronene-filled MWCNTs yields polymerized coronene products, but no internal NTs are formed (Figure 39).^881^ Kharlamova et al. reported that the controlled filling of SWCNTs (diameter of 1.7 nm) with Ni­(Cp)_2_ can also form DWCNTs after the appropriate thermal treatment is carried out.^882^ More recently, Botos et al. reported the multistep synthesis of inorganic nanoribbons within the nanometric cavities of SWCNTs.^883^ Being electron donors, CNTs act as promoters for the initial step of the reaction, which involves the interaction of iodine anions with M­(CO)_6_ (M = Mo or W), leading to the formation of [M_6_I_14_]^2–^ anionic nanoclusters.

**Figure 39:**
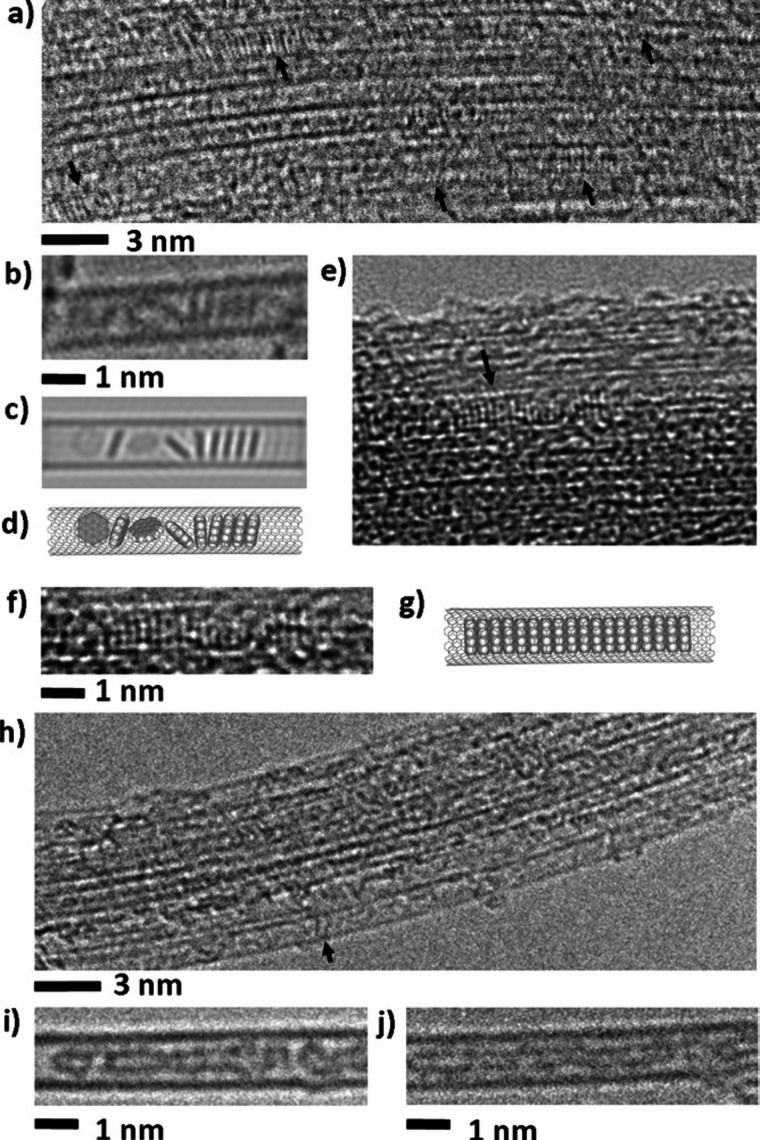
a–b) TEM images of purified SWCNTs filled with coronene at 50 °C using scCO_2_. Coronene stacks located within the NT bundles are indicated by black arrows. d) Structural diagram and c) simulated TEM image of coronene molecules stacked inside the CNT are in good agreement with the experimental data. e–f) TEM images of purified SWCNTs filled with coronene in the gas phase at 385 °C and 450 °C. h) Stacks of coronene molecules extend through the CNT interior and g) structural diagram corresponding to the experimental image. i–j) Coronene molecules quickly transform into amorphous carbon or internal nanotubes under the e-beam.^881^ Reproduced with permission from ref 881. Copyright 2013, John Wiley and Sons.

Confinement effects were also observed for the template synthesis of sulfur-terminated GNRs from tetrathiafulvalene (TTF) molecular precursor by heat treatment or electron beam irradiation.^65,94,535^ HRTEM images in Figure 40 show the influence of the diameter of the SWCNTs on the structure of the final products obtained from TTF molecules.^65^

**Figure 40:**
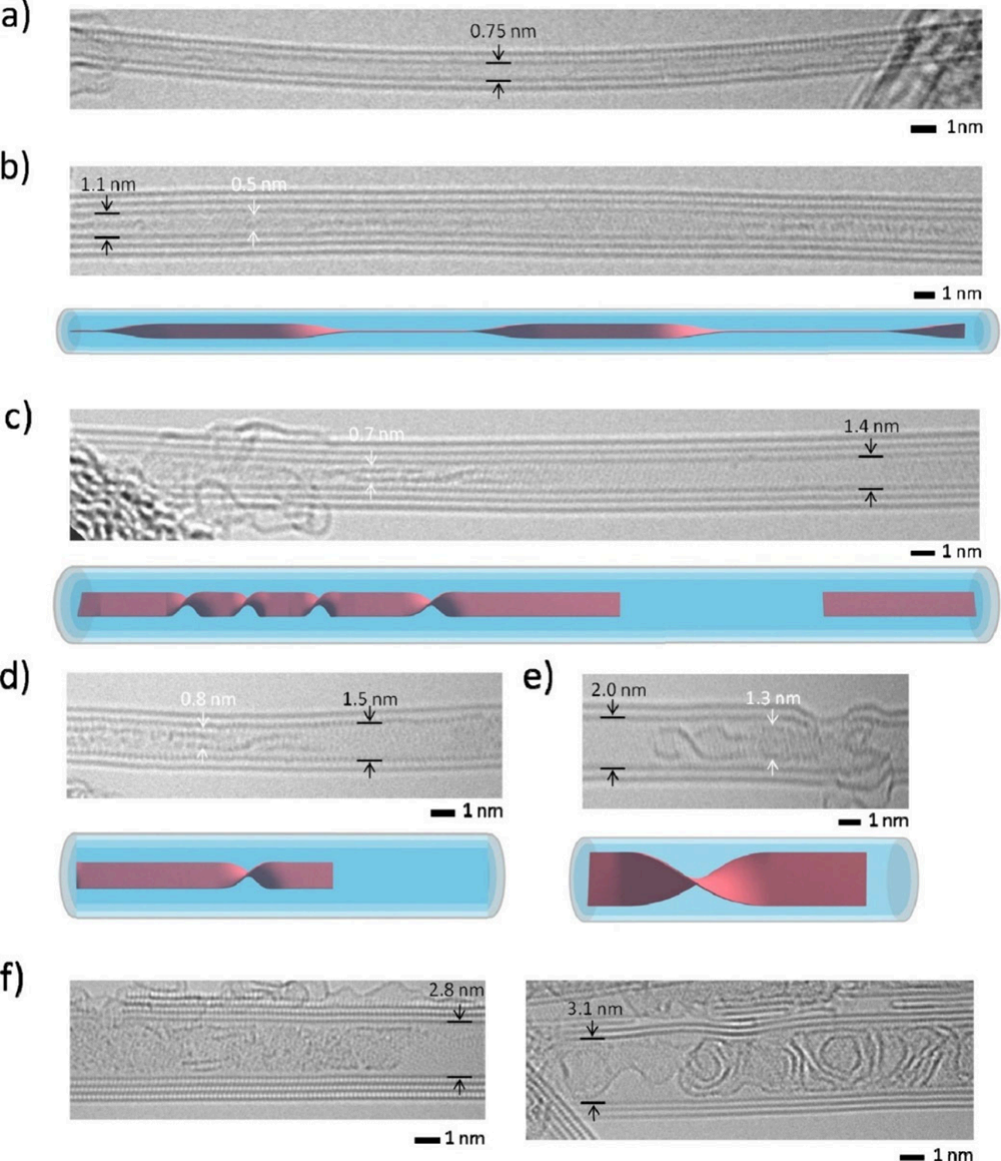
AC-HRTEM images (80 kV) showing the effects of the CNT internal diameter on the structure of the products formed inside CNTs. a) Narrow CNT (internal d_NT_ = 0.75 nm) containing a chain of atoms rather than a S-GNR. b–e) CNTs with internal diameters in the range of d_NT_ = 1–2 nm consistently contain S-GNRs, which exhibit helical twists (indicated by the schematic representation under each image; the nanoribbon and CNT are depicted in red and blue, respectively). f) Wide CNTs (left internal d_NT_ = 2.8 nm and right internal d_NT_ = 3.1 nm) contain structurally poorly defined, semiamorphous structures (left image) and carbon “onions” (right image) as the host CNTs are too wide to efficiently template nanoribbon formation.^65^ Reproduced with permission from ref 65. Copyright 2012, American Chemical Society.

Confinement not only promotes enhanced interactions between the guest reactant molecules encapsulated within CNTs, but also favors the preferential formation of specific isomers owing to spatial restrictions and electronic factors. Miners et al. studied aromatic halogenation^884^ and more recently, 1,3-dipolar cycloaddition reactions^885^ performed within CNTs, which play an active role in both the selectivity and kinetics of the processes. In the first case, the confinement of *N*-phenylacetamide and pyridinium dichlorobromate (halide source) within SWCNTs favored the para-selective bromination of the aromatic ring. The polarizing character of CNTs lowers the activation energy due to the stabilization of the dipole moment of the reaction intermediate, significantly increasing the reaction rate. Similar results were observed when performing 1,3-dipolar cycloadditions of alkyne species to benzyl azide. 1,4-triazole was the major product. In this case, the SWCNTs’ cavity induces shape selectivity, favoring the formation of the more linear isomer.

As mentioned above, SWCNTs have been used as platforms for the encapsulation and polymerization of white phosphorus.^15^ In addition, owing to the confinement effects provided by the SWCNTs, they also play an important role as a shield against the highly exothermic reaction of white phosphorus with atmospheric oxygen. The formation of oxidized phosphorus species on the tip of the SWCNTs after the first air exposure prevented further oxidation of the remaining entrapped white phosphorus. (Figure 6). The encapsulation, polymerization, and stabilization of P_4_ in SWCNTs at RT provide an exciting opportunity to isolate and perform fundamental studies on highly reactive intermediate species of phosphorus.

The use of CNTs as nanoreactors is not limited to the organic synthesis of DWCNTs or other derivatives, where the composition and structure of the products are predetermined by the reaction conditions. CNTs have also been extensively explored as nanoreactors for the controlled synthesis of new inorganic nanostructures.^886^ However, the synthesis of well-defined inorganic nanostructures is usually more challenging because of the possibility of yielding non-stoichiometric or polymorphic products. Botos et al. suggested an innovative concept for the use of SWCNTs as electrically active nanoreactors for the multistep inorganic synthesis of molybdenum iodide [Mo_6_I_12_]_*n*_ or molybdenum/tungsten disulfide [MS_2_]_*n*_.^883^ SWCNTs were used as electron donors to the encapsulated I_2_ in order to form I^–^, which then reacted with metal hexacarbonyls (M­(CO)_6_, M = Mo or W) yielding anionic nanoclusters [M_6_I_14_]^2–^ arranged in a perfect octahedral geometry. In the third step of the synthesis, the anionic nanoclusters could react with each other to form a new polymeric phase of [Mo_6_I_12_]_*n*_ or with H_2_S gas to form nanoribbons of molybdenum/tungsten disulfide [MS_2_]_*n*_ (Figure 41), respectively.

**Figure 41:**
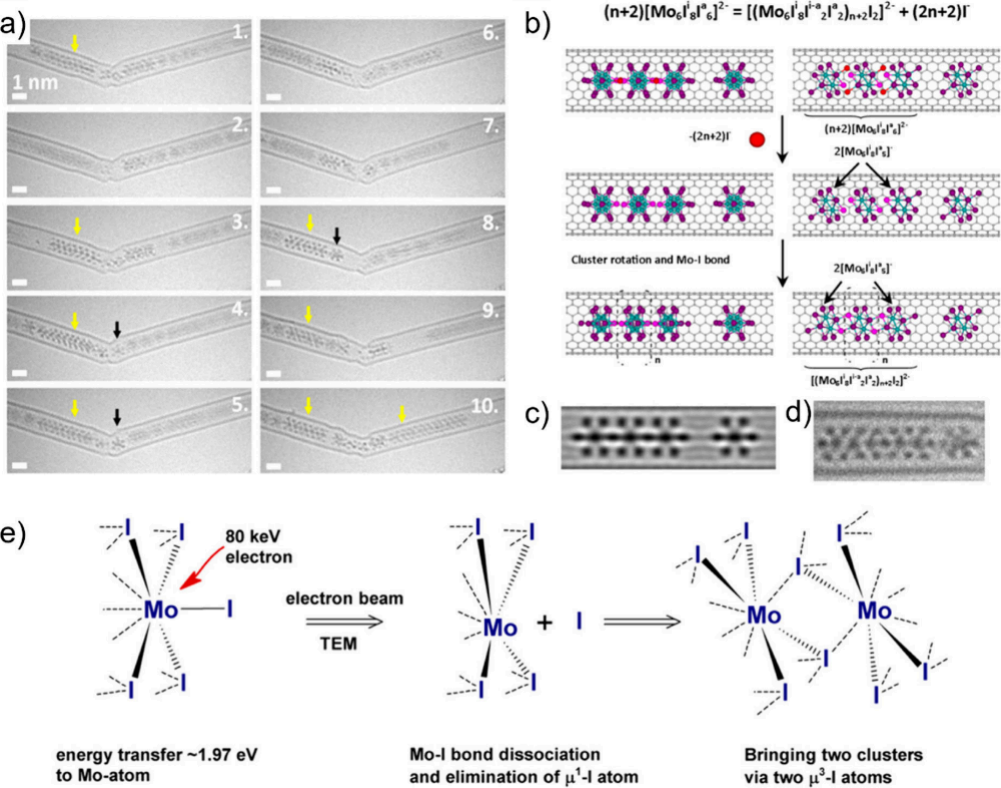
a) AC-HRTEM at 80 kV time series images showing the transformation of n·[Mo_6_I_14_]^2–^@SWCNT_2n_^+^ to [Mo_6_I_12_]_*n*_@SWCNT (yellow arrows) under 80 keV e-beam. b) Structural diagram of a reaction of iodide elimination and formation of the polymeric form of [Mo_6_I_12_]_*n*_. d) High magnified 80 kV AC-HRTEM images of [Mo_6_I_12_]_*n*_@SWCNT and c) corresponding image simulation. e) Schematic diagram that illustrates the kinetic energy transfer from the incident 80 keV electron to the Mo atom (up to 1.97 eV according to eq 1), which can trigger the dissociation of the Mo–I bond with μ1 -I followed by μ3 -I linking of the neighboring clusters and thus leading to the polymeric [Mo_6_I_12_]_*n*_ structure observed in AC-HRTEM.^883^ Reproduced with permission from ref 883. Copyright 2016, American Chemical Society.

The authors explored a combination of HRTEM imaging, EDX, and Raman spectroscopy to follow the multistep synthesis inside the SWCNTs and to prove the influence of the electron transfer mechanism on the stabilization of the anionic MX nanoclusters and their further transformation to MS_2_ nanoribbons. The formation of various ultrathin nanoribbons of TMCs confined within CNTs has been reported. Examples include the synthesis of TaS_2_,^18^ WS_2_@DWCNTs,^887^ HfTe_2_@MWCNTs^888^ and HfTe_3_@MWCNTs^69^ via CVT. More recently, Norman et al. proposed the multistep synthesis of ReS_2_ by encapsulation of a dirhenium decarbonyl complex within the CNTs, followed by its conversion into rhenium iodide and finally the formation of ReS_2_@SWCNTs by treatment under H_2_S.^889^

The controlled growth of gold NWs in the internal cavity of MWCNTs through isolated gold NPs, using a real-time manipulator installed in a TEM, provides another interesting example of the application of CNTs as nanoreactors. The authors reported the successful growth of a 45 nm-long gold NW in the hollow internal cavity of a MWCNT. They showed that the gold NW length could be precisely controlled (with less than 1 nm) by the number of serial contacts with gold NPs located on a supportive DWCNT. The main mechanism for the controlled growth of gold NWs inside CNTs was identified to depend on the nucleation of the encapsulated NW by thermomigration and the continuous coalescence of metal NPs by nanocapillary forces.^890^ Recently, the same group of authors observed that the origin of the nanocapillary force in the cavities of CNTs depends on the nature of the loaded material.^891^ For CNTs filled with Au NPs, the origin of the nanocapillary forces is dominated by the coalescence of NPs. In contrast, this phenomenon was not observed when Pt NPs were filled. The authors presume that the strong interaction between the carbon layers of CNT and Pt NPs suppresses adatom diffusion, which consequently prevents mass transport by coalescence. Indeed, it has been reported that a strong thermal gradient (thermomigration) is the origin of the nanocapillary forces for the controlled growth of Pt NWs inside CNTs. Haft et al. also demonstrated the ability for the controlled growth of NWs of group IV-elements in their metallic state (Sn, Ge and Pb) inside MWCNTs. The MWCNTs were previously filled with the respective molecular precursors and then thermally reduced in a tube furnace using a hydrogen–argon-mixture. The authors identified that the experimental parameters like reaction time, solvent, reduction *T* or concentration of filling agents in solution, allow to control the content and shape of different metal-filled CNTs.^292^

CNTs were also explored to confine and stabilize copper azide-based compounds to avoid detonation due to electrostatic charges during handling.^892^ In this study, CNTs were previously filled with copper oxide NPs. The guest NPs were then reduced using hydrogen. The resulting sample was subsequently treated with hydrazoic acid gas to produce the final copper-azide-filling material. The authors suggest that these new hybrids can be explored as nanodetonators and green primary explosives. This study provides relevant background insights into the physics of detonation at the nanoscale. The TEM images in Figure 42 show that copper azide NPs were formed inside the CNTs and exploded after initiation. Even after initiation, the reaction products were retained inside the CNTs walls, generating 10–20 nm hollow shells. Additionally, no signs of MWCNTs’ wall rupture were observed after violent initiation. The authors suggested that the propagation of the detonation wave along the 1D channel of MWCNTs might help maintain their structural integrity.

**Figure 42:**
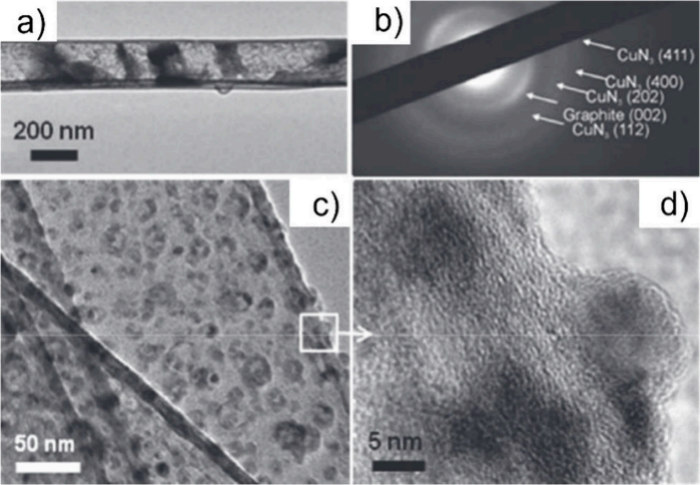
CNTs as stabilizing agents of azide-based compounds. TEM micrograph of a) CuN_3_/Cu­(N_3_)_2_ filled CNT, and b) SAED pattern of the CuN_3_ filled CNTs. These hybrid materials were proposed as potential nanodetonators and green primary explosives. c) TEM micrograph of a sample of copper azide-filled CNTs after the detonation was initiated, and d) HRTEM image of detonation products at the CNT wall.^892^ Reproduced with permission from ref 892. Copyright 2010, John Wiley and Sons.

### 8.5 Catalysis

The confinement of chemical reactions within the internal cavity of a nanoreactor allows for the effective control of the active sites of the catalyst and provides optimal conditions for determining the yields and selectivity of the reaction products.^893^ The advantages of using nanoreactors are evident at different levels of the catalytic chemical reaction, from reactants to products, including catalyst activity. It is expected that the interactions between the cavity and the reactant might increase the local concentration of species and favor the preferential orientation of molecules, thus leading to an improvement in the reaction rate.^894^ Furthermore, the confinement imposed by the cavity of the nanoreactor can benefit the spatial orientation of certain types of molecules, thus favoring the synthesis of specific isomers or oligomers, thereby improving the selectivity of the catalytic reaction.^895^ The confinement of the catalyst inside the nanoreactor walls can enhance the stability of its active sites. In some cases, the catalyst can establish electronic interactions with the host, inducing variations in the electronic states that can reduce the activation energy of the catalytic reaction and therefore improve the reaction kinetics.^896^

A large variety of engineered nanomaterials have already been explored as effective nanoreactors for different catalytic processes, including cavitands, zeolites, metal–organic frameworks, cyclodextrins, imprinted polymers, silicates, concurbituris, enzymes, and CNTs. As described previously, CNTs can be used to carry out reactions as simple vessels or nanoreactors for catalytic reactions. The main difference between these two applications lies in the nature of the reactions that occur within the cavities of the CNTs. The application of CNTs in catalytic reactions has several advantages, such as high thermal and chemical stability, mechanical robustness, and high electronic conductivity.^896^ The use of SWCNTs with very narrow diameters (0.7–2 nm) provides a high surface area, and the confinement effects are more pronounced between the reactant and catalyst.^61^ However, in the case of MWCNTs, their larger diameters might reduce the confinement effects, thus affecting the efficiency of the catalytic mechanism.

A recent review reported the potential and new developments of CNT-based nanoreactors for confining various M/M_*x*_O_*y*_ catalysts.^896^ CNTs have been used for the encapsulation of active systems to promote hydrogenation,^897–899^ hydrogen production,^900,901^ reduction^902^ and oxidation reactions. Generally, the confinement of the metallic catalyst has an important impact on the different levels of the catalytic reaction. It has been described that the hosting CNTs can change the electronic state of the catalyst, consequently enhancing the catalytic activity. This phenomenon can be advantageous for certain types of catalytic processes, depending on the reaction mechanism and specific interactions with the catalyst and reaction products.^139^ Other advantages include an improvement in the catalyst stability and the increase of the reaction selectivity.^896^ Despite significant progress in the field, a major drawback in the use of CNTs is the lack of uniform approaches or methodologies to measure the activity or selectivity of confined catalysts.^893^

Encapsulation within CNTs can also contribute to decreasing the poisoning of the catalyst, which usually occurs when the active sites are blocked by side products deposited onto their exposed surface (poisoning effects). These include, for instance, stable sulfate species resulting from the reaction of NH_3_ and O_2_ in SO_2_ rich environments during the selective catalytic reduction (SCR) of NOx.^903^ In the presence of CNTs, the reactivity of the system increases, and the decomposition of ammonium sulfates (NH_4_HSO_4_) is favored. In this case, SO_2_ contributes to the catalytic mechanism because the emerging NH_4_HSO_4_ species rapidly react with NO molecules at low *T*,^904^ and consequently, an improved catalytic activity is observed.^905^ One particular example of the advantages of using CNTs to improve the catalytic performance through encapsulation is the Fischer–Tropsch (FT) reaction, which stands out from other synthesis processes currently available for the production of liquid fuels. In the FT reaction, syngas (a mixture of CO and H_2_) is used as an intermediate, starting from feedstocks such as coal or biomass, to obtain long-chain hydrocarbons, thereby potentially reducing the reliance on non-renewable energy sources such as crude oil. The reaction is usually catalyzed by transition metals such as Co and Fe. Despite their high selectivity toward the production of heavy hydrocarbons, the process still faces drawbacks, including catalyst poisoning, deactivation, and formation of undesired side products such as methane. Encapsulating Co and Fe oxides within CNTs allows enhanced dispersion of the metallic particles, thus limiting sintering, which is one of the main reasons for the deactivation of the catalyst.^906–908^ Confining the catalyst also avoids poisoning due to interaction with sulfur species from the carbon sources employed during the FT process. Chen et al. showed how changes in the redox properties of iron catalysts confined inside MWCNTs can affect catalytic activity.^906^ The authors observed that within the confined CNT space, iron tends to form more reductive species preferentially, such as iron carbide (instead of FeO), which improved the activity of the catalyst. The formation of long carbon chains (C5^+^) when the catalyst was confined within the interior of the CNTs was favored, yielding to twice (33%) the amount compared to the system in which Fe_*x*_O_*y*_ NPs were deposited onto the external walls of the support.^906^ Furthermore, a higher selectivity for hydrocarbons in Fe-filled CNTs was reported. In fact, it has been suggested that the reduction in the amount of encapsulated catalyst can be remarkably improved by H_2_ or CO, thus contributing to the formation of more available active sites of carbides that improve the catalytic reaction. An additional advantage of CNTs in the FT process is their high thermal conductivity, which contributes to the heat dissipation during the exothermic reaction.^909^ The confinement of the reaction intermediates inside the CNTs channels prolongs the extent of the catalytic reaction, thus favoring the formation of longer hydrocarbon chains.

Catalytic hydrogenation using different metal NPs confined within CNT-based nanoreactors (namely, Pd, Ni, Pt, Au, Ru, Fe and Co) has been reported for different precursors, such as benzene and its derivatives, α-ketoester, cinnamaldehyde (CAL), cellobiose, and CO (syn gas).^896,898^

Oval and short rod like Pd NPs were encapsulated within MWCNTs for the hydrogenation of quinoline to 1,2,3,4-tetrahydroquinoline (py-THQ), usually employed as precursor for the formation of organic compounds of interest as dyes or drugs. Currently, obtaining this aromatic heterocyclic compound is challenging because the available synthetic approaches can also lead to the reduction of the benzene ring, and therefore, to the formation of other N-containing cyclic compounds such as decahydroquinoline (DHQ) or 5,6,7,8-tetrahydroquinoline (bz-THQ). In this case, the Pd@CNTs catalyst led to ultraselective formation of py-THQ, with a 100% conversion, which results in a significant improvement compared to the reaction in the presence of palladium decorated CNTs (Pd-CNTs) or palladium NPs supported on active carbons (Pd-AC), with conversions of 63.8% and 52.1%, respectively. This behavior has been explained in terms of the charge transfer between the hosting CNTs and the guest NPs, which become electron-deficient after encapsulation, contributing to the activation of H_2_ and promoting the adsorption of quinoline through the interaction of the heterocyclic N with the Pd NPs. This favors the reduction of this fraction of the molecule instead of the hydrogenation of benzene.^910^ Filling the NPs within the hosting CNTs prevents aggregation and deactivation of the catalyst, as observed in reactions promoted by Pd-AC and Pd-CNTs, in which the NPs underwent oxidation during air contact. Metal and metal oxides confined within CNTs have also demonstrated enhanced behavior in efficiently promoting oxidation reactions at low *T*. Reports describe significant improvement in the preferential oxidation (PROX) of CO in a H_2_-rich stream using Ru,^911–913^ CuO-CeO_2_,^914^ and Cu_*x*_Ce_1–*x*_O^915^ encapsulated within tubular nanocarbons. In a recent study, Pt clusters confined inside DWCNTs were developed for the oxidative catalysis of toluene. The restricted size of Pt nanoclusters confined within DWCNTs and their high stability (conferred to Pt catalytic active sites) were claimed as the main reasons for the improved catalytic activity observed.^916^

The encapsulation of active compounds within CNTs also finds application in the elimination of water contaminants. Relevant examples include the confinement of Fenton-like compounds such as Co_3_O_4_, which are used in heterogeneous catalysis. This approach overcomes the main drawbacks associated with the utilization of short-lived radical species and low mass transfer from the generation sites on the catalyst to the pollutants, limiting the consumption of the catalyst by the aqueous matrix. An unprecedented catalytic activity was observed, with high reaction rates and selectivity in water decontamination, associated with the activity of singlet oxygen (^1^O_2_) species.^917^ Further studies documented the degradation of moxifloxacin (MOX),^918^ and sulfamethoxazole (SMX)^919^ via PMS activation. In these cases, the authors encapsulated both Mn/Co and CoFe_2_O_4_ spinels within CNTs, achieving removal rates of 89.7% in 30 min for MOX and 98.46% in 60 min for SMX. Liu et al. reported the successful catalytic activity of Co_3_O_4_@CNTs for the degradation of norfloxacin (NX) in a peroxymonosulfate (PMS) system.^920^ The catalytic performance of the evaluated system was notably superior (Co_3_O_4_@CNTs, 94.8%) to that of other tested catalysts, namely, Co_3_O_4_/AC, Co_3_O_4_/Diatomite, and Co_3_O_4_/SBA-15, with degradation efficiencies between 25.9% and 34.4%. The authors evaluated the factors that played a main role in NX conversion, including pH, catalyst and PMS dosage, and *T*. The NX removal efficiency was directly related to the amount of catalyst added to the system, reaching its maximum with Co_3_O_4_@CNT concentration of 150 mg L^–1^. This efficiency can be affected by the amount of PMX, which was a limiting factor at low concentrations, but could also saturate the active sites when present in high dosages. The oxidation of NX was significantly affected by pH variations. The optimum conversion was set at a pH between 3 and 9.4. Within this range, PMS (in the form of HSO_5_^–^) easily produced reactive ^1^O_2_ species, and the interaction of the reactants was favored. The catalyst particles acquired a slightly positive character owing to the strong interaction with the support walls, which also provided stability and prevented the leaching of metal ions. This also enhanced the electrostatic interaction between the catalyst and HSO_5_^–^ fraction. Moreover, under acidic conditions, NX mostly adopts its protonated form, NX-H^+^, for further oxidation.

Table 11 summarizes the main reports on the applications of filled CNTs in catalysis.

**Table 11:** Main Approaches for Applying Filled CNTs in Catalysis

Catalysis approach	Primary Role of CNTs	Typical filler	Applications	References
**Reactions at** ***T* < 300 °C**	• Support.	Transition metals (namely, Pd, Ni, Pt, Au, Ag, Ru, Fe and Co, Cu).	Hydrogenation.	^896–899^,^910^,^921–923^
	• Electron highway.	MNi (M: Co,Cu,Fe), Pd.	Hydrogen production.	^900^,^901^
	• Thermal conductor.	Fe, Co.	Hydrocarbon production: FT reaction.	^906–908^
	• Confinement.	CeO_2_, MnOx.	NOx reduction.	^904^,^924^
		Ru, Cu_x_Ce_1_O, CuO-CeO_2_ binary oxides.	PROX of CO.	^911–915^
		Fenton-like compounds (CuFe_2-x_O_4_, Co_3_O_4_).	Degradation of organic pollutants (pharmaceuticals).	^917–920^
		Pd–Cu.	Water denitration.	^902^
		Pt.	Oxidation of toluene.	^916^
**Thermocatalysis**	• Support.	Pd.	Hydrogenation.	^925^
	• Thermal conductor.	Ni, Ce–Sr–Co, MoC_2_, Fe–Co–Cu.	Syngas production reactions: methane reforming.	^926–930^
	• Confinement Sintering mitigation.	Ce–Mn oxides, CeO_2_.	Oxidative dehydrogenation.	^931^,^932^
		Transition metals (Fe, Ni, Co).	CO_2_ conversion.	^323^,^896^,^926^,^933–935^
		Metal oxides.	FT synthesis.	
			Various oxidation reactions.	
		Rh-based catalyst.	Ethanol production.	^936^
			SCR of NO.	^904^
**Electrocatalysis**	• Conductive support.	Bi-NRs, Ni.	CO_2_RR.	^937–939^
	• Electron highway.	FeCo, Fe_3_C, Co, CoNi, CoFe/CoFe_2_O_4_, CoFe-NiFe, Co–Ni, PdMo.	ORR/OER, UOR.	^88^,^179^,^249^,^261^,^263^,^325^,^339^,^940–944^
	• Confinement.	Ni, Fe@Fe_2_P, Cobalt and β-Mo_2_C, Cr-doped FeNi-P, IrCo, Ru, PtCo.	HER.	^237^,^938^,^945–951^
		FeCo.	Hydrolysis.	^320^
**Photocatalysis**	• Electron acceptor/donor.	Semiconductor NPs (TiO_2_, CdS).	Degradation of organic pollutants (dyes, pharmaceuticals).	^952^
	• Light harvester.	TiO_2_.	Bacterial disinfection.	^953^
	• Support.	Co.	Water splitting (H_2_ production).	^954^
		TiO_2_, Au, FeNi.	Various oxidation reactions.	^955–957^
		PTH.	Hydrodehalogenation reactions.	^958^
**Magnetocatalysis**	• Magnetic particle encapsulation.	Ferromagnetic NPs (Fe, Co, Ni), their oxides.	Specialized chemical synthesis where magnetic separation/heating is beneficial.	^326^,^959–963^
	• Support.			
	• Heating.			

One particular advantage of CNTs is their susceptibility to functionalization, either by attaching bearing functionalities onto their external surface or by inducing structural modifications through the introduction of defects, such as replacing carbon atoms from their honeycomb lattice with heteroatoms such as nitrogen or boron, thus leading to the formation of nitrogen-doped CNTs (NCNTs) and B-doped CNTs (BCNTs).^964^ Substitutional doping is one of the most widely used strategies to modify the electronic structure of CNTs. Heteroatoms break the symmetry of the graphitic electron band structure, shifting the Fermi level either toward the conduction (electron attractor, p-doping) or the valence band (electron donor, n-doping), thereby introducing semiconducting behavior or enhancing the metallic character of the NT.^80,965^ These modifications may contribute positively to the catalytic reaction because the presence of the heteroatom enhances the chemical reactivity and surface polarity, which promote better absorption of the reactants. The encapsulated catalyst can establish stronger chemical interactions with heteroatom-containing groups and enhance the electron transfer properties, thus leading to higher stability of the system and minimizing the probability of leakage. A synergistic effect was also observed between the doped hosts and the catalyst because doped sites actively participated in the reaction mechanism. They act as additional active sites involved in the formation of reaction intermediates that contribute to the decrease in the activation energies (*E*) of the reactions. Consequently, by controlling the doping degree of the host, fine-tuning and potential improvement of the catalytic performance can be achieved, including aspects such as activity,^966^ selectivity, and stability.^326^ Multiple examples have been reported on using metallic catalysts confined within heteroatom-doped CNTs to promote the Oxygen Reduction Reaction (ORR), Oxygen Evolution Reaction (OER), CO_2_ reduction, and various organic reactions where electron transfer or specific binding is crucial.

Improved behavior was observed after testing Ni–Co–NPs@NCNTs and Fe–Co@NCNTs as catalysts for the degradation of organic pollutants.^323,326^ The superior catalytic performance was attributed to factors such as the presence of N atoms within the conjugated system (doping), synergistic effect between the filled NPs and the host, and protective effect of their encapsulation, which prevents metal leaching into the aqueous medium. N-doped CNTs filled with Fe/Fe_3_C were used for the degradation of the antibiotic drug tetracycline hydrochloride (TC), which, along with other similar drugs, represents a challenge owing to its increasing presence in aquifers and the environmental impact it entails.^337^ The reaction proceeded via the activation of PMS, an efficient agent for the advanced oxidation of the pollutant. After 30 min, the catalyst removed up to 95.2% of TC owing to the generation of free radicals and ^1^O_2_, increased electron transfer efficiency, and activation of PMS, facilitated by Fe-based particles and graphitic N from the hosting tubes.

A significant improvement in the catalytic hydrogenation of a series of biomass-derived compounds promoted by Co encapsulated within N-doped CNTs was reported.^967^ The formation of Co–N_*x*_ active sites provided stability and high reactivity to the supported catalyst, enabling the selective hydrogenation of furfuryl alcohol, benzaldehyde, hydroxymethylfurfural, vanillin, and CAL, leading to a 100% efficiency conversion into the respective alcohol. Superior selectivity toward the conversion of ketones, carboxylic acids, and nitroarenes was also observed as well. Lin et al. investigated the green synthesis of benzimidazoles catalyzed by N-doped CNTs confined Co NPs. The proposed approach was extended to the production of the fungicide fuberidazole, demonstrating the versatility of the supported catalyst. Moreover, the material was easily recovered by applying a magnetic field, which facilitated its recyclability. By DFT calculations, the authors determined the energy involved in the different steps of the hydrogenation reaction, systematically demonstrating the favorability of the reaction when the catalyst was confined within the N-doped host.^968^

Catalyst-assisted processes can be classified in terms of other forces contributing to the acceleration of the chemical reaction, such as heat (thermocatalysis), light (photocatalysis), electric current (electrocatalysis), or magnetic field (magnetocatalysis). While the catalyst promotes the reaction by lowering the activation energy of the process, the applied driving forces optimize the reaction rate by providing sufficient kinetic energy to the molecules to activate the reaction mechanism. The combined action usually results in more efficient, selective, and sustainable processes.

#### 8.5.1 Thermocatalysis

This approach is one of the most widely used techniques because of its ease of scalability, making it suitable for implementation in industrial processes, including syngas production reactions,^926–930^ hydrogenation, and dehydrogenation reactions.^931,932,969^ The application of heat (typically 300–800 °C) increases the vibration and mobility of the species involved in the reaction and, therefore, the probability of collision. CNTs confer enhanced properties to the thermocatalysts encapsulated within their interior. Confinement protects the material from sintering, agglomeration and oxidation, while their high thermal stability and thermal conductivity improve heat transfer and favor its homogeneous distribution along the container. Moreover, once the material is encapsulated, leaching into the reaction medium is minimized, which increases the reaction yield and avoids catalyst deactivation.^970,971^

Thermocatalysts based on CNTs encapsulating foreign materials have been prepared using both *in situ* and *ex situ* synthetic approaches. In 2007, Pan et al. encapsulated a Rh-based catalyst inside MWCNTs to improve the catalytic production of ethanol by using a mixture of CO and H_2_ (syngas).^936^ Afterward, Castillejos et al. reported the effect of the confinement of bimetallic PtRu NPs within MWCNTs for the selective hydrogenation of CAL (Figure 43).^933^

**Figure 43:**
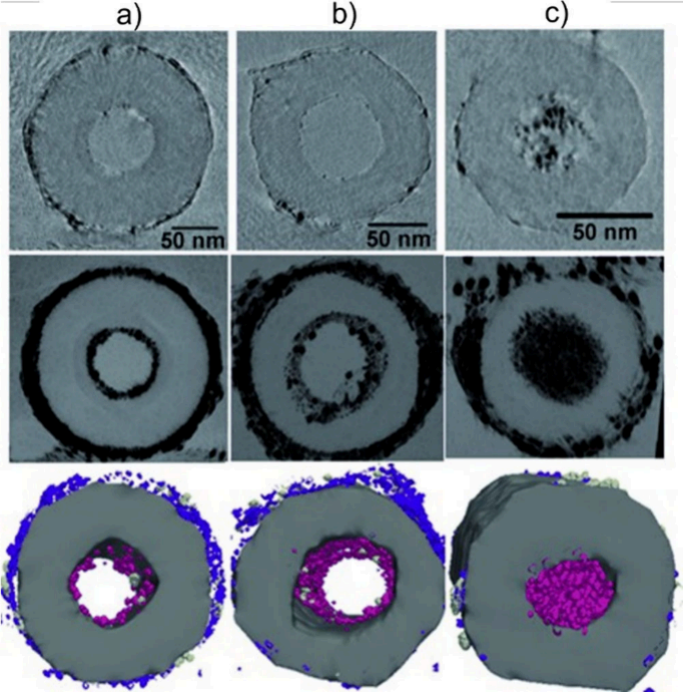
3D TEM analyses used to determine the spatial location of PtRu NPs confined within MWCNTs. The top part of the image includes examples of transverse sections extracted from the reconstruction. The contribution of the PtRu NPs (dark areas) is highlighted by presenting the minimum intensity projections of all sections on the same plane (middle). Bottom: Modeling of the reconstruction of PtRu-loaded CNTs prepared by a) the decomposition of Pt and Ru in the presence of CNTs functionalized with O-bearing moieties (5 wt % PtRu) and impregnation of b) pristine CNTs (11 wt % PtRu) and c) L functionalized CNTs (23 wt % PtRu) with RuPt NPs using THF as solvent. The large, round-shaped NPs on transverse sections and global projections are AuNPs deposited before 3D TEM analysis to facilitate the treatment of the tilt series for the reconstruction: violet PtRu NPs outside, red PtRu NPs inside, and yellow Au NPs.^933^ Reproduced with permission from ref 933. Copyright 2009, John Wiley & Sons.

The authors employed two approaches for loading CNTs with bimetallic NPs using pristine CNTs, CNTs functionalized with O-bearing moieties, and CNTs functionalized with an amide-containing long alkyl chain. The first method consisted in a liquid-phase impregnation process using THF as the solvent, which led to 5–23 wt % PtRu-CNTs. In the second case, the thermal decomposition of Ru and Pt precursors was carried out in the presence of 4-(3-phenylpropyl) pyridine (L) as a ligand and CNTs. The spatial location of the loaded NPs was evaluated using both 2D and 3D TEM, which suggested that the encapsulation of NPs within the inner cavities of the CNTs was favored in the case of alkylamide-functionalized hosting tubes (Figure 43 c)). An improved selectivity from 35% for bimetallic NPs to 95% for confined PtRu/MWCNTs toward the thermocatalytical obtaining of cinnamyl alcohol (COL) was observed. The authors suggested that CAL adsorption perpendicular to the Pt surface inside CNTs favors the selectivity for COL. Furthermore, a higher concentration of active hydride species was observed for the confined Ru NPs, which could improve the catalytic reaction.

Recently, Wang et al. reported improved hydrogenation of CO_2_ to produce methanol using Pd NPs confined within MWCNTs.^925^ A greater conversion was observed (1.3 times), along with the selectivity to methanol production (3.6 times) for the confined Pd catalyst, when compared with the non-encapsulated catalyst. These results were attributed to the high electronic density inside the CNTs, which can stabilize more Pd^δ+^ catalytic active sites and consequently improve the catalytic CO_2_ hydrogenation to produce methanol. Many other examples of catalytic oxidation reactions using different metallic NPs confined into CNT-based nanoreactors, such as, Ni, Au, Ru, MnO_2_, Co, Cu, TiO_2_, Au, and Pt, have also been reported.^969^ The application of these hybrid materials was investigated for different precursors such as, CH_4_, CO, benzyl alcohol, styrene, propylene, silane, toluene, hydroquinone and methanol among others.^323,896,934^

MWCNTs were also used to confine Ni NPs during the catalytic oxidation of CH_4_. The authors reported that the higher catalytic activity for confined Ni NPs was mainly due to the high electron density and the improved stability of the catalyst provided by the confinement within CNTs.^926^ Alloyed FeNi-filled CNTs showed superior catalytic oxidation of cyclohexane, using molecular oxygen as the oxidant, when compared to Ni-filled CNTs. The confinement of the electron-donating metals within CNTs could favor electron transfer.^935^

#### 8.5.2 Electrocatalysis

Applying an external electrical potential can increase the rate of catalyst-driven reactions. These reactions, which involve charge transfer (Faraday reactions), may occur on the surface of a material acting as an electrode within an EC cell. Because of their physical, mechanical, and chemical properties, CNTs offer a compelling set of advantages when used as electrodes in these systems.^972^ In their metallic and semimetallic phases, they are highly conductive, thus favoring electron transfer from the electricity source through the electrolyte-electrode interface to finally reach the surface of the catalyst, which is the driving force enabling the reaction to occur. Their morphology and inner cavities facilitate the mass transport of the reactants, while their high surface area allows encapsulation of a wide amount of both catalyst and reactants, thus increasing the active site density and, therefore, the conversion yield. CNTs are mechanically and chemically stable but are also susceptible to exohedrally functionalization, which provides an opportunity to tune their surface chemistry and enhance their chemical affinity with the electrolyte or promote the absorption of the reactant. Functionalization may also create active sites onto the CNTs walls that contribute to the formation of intermediates.^973^ Metal- and alloy-filled CNTs have been explored in many types of electrocatalytic reactions, with several reports on their use in the catalytic synthesis and decomposition of ammonia,^261^ EC oxidation of hydrazine, EC reduction of methanol, oxygen and CO_2_ ^320,896,937,943^ or as catalysts for Hydrogen Evolution Reaction (HER),^237,325,945–948^ among others.^972,974^

CNTs exhibit properties inherent to their C-conjugated nanostructures, which contribute to performance enhancement and promote the evolution of certain reaction mechanisms. This is the case for their proven activity for the ORR, which, for practical purposes, requires the modification of the electronic structure of the C network or the introduction of other elements into the C matrix.^249,940^ Gao et al. fabricated efficient ORR electrocatalysts for fuel cells and metal–air batteries based on N-doped MWCNTs filled with Fe_3_C NWs.^940^ The hybrid material exhibited excellent ORR catalytic activity and half-wave potential (0.88 V), long-term durability, and excellent methanol tolerance, mainly induced by the strain effects on the geometry and electronic structure of the carbon atoms adjacent to graphitic-N. More recently, N-doped CNTs encapsulating Co NPs,^263^ FeCo NPs,^179^ Co-based bifunctional catalysts,^941^ CoNi NPs^339^ or CoFe-NiFe biphase alloy nanoheterojunctions^942^ have been used for ORR and OER, and represent potentially useful alternatives for developing rechargeable magnesium or zinc–air batteries.^339,941^ In the case of CoFe/CoFe_2_O_4_@NCNTs, the reaction proceeds via the Fe–N_*x*_ and Co–N_*x*_ sites and the presence of oxygen vacancies, generated by the electron transfer at the CoFe/CoFe_2_O_4_ interface, contributes to the capture and release of oxygenated species.^941^ Other examples include the encapsulation of Co–Ni NPs within nitrogenated CNTs, leading to catalysts with excellent ORR/Urea Oxidation reaction (UOR) activity. These were further employed to build urea-assisted rechargeable batteries with up to 61% energy efficiency.^88^ Hybrids formed by Co–Ni being part of carbon nanofibers-based 3D arrays were also synthesized via electrodeposition. The material was then annealed between 400 and 750 °C and showed superior potential toward the evaluated reactions, compared to materials available to date.^339^

Zhang et al. evaluated the role of nanoconfinement effects in the electrocatalytic performance of Bi NRs encapsulated within N-doped CNTs for the CO_2_ Reduction Reaction (CO_2_RR).^937^ In addition to the advantages inherent to the encapsulation of the material, such as aggregation prevention and protection against Bi oxidation, the high surface area and enhanced electronic conductivity provided system stability against the cathodic polarization. The confinement of the reactants increased the reaction rate, leading to a Faradaic efficiency of up to 90.9% for formate production, with a high selectivity toward the CO_2_RR against the competing side HER.

#### 8.5.3 Photocatalysis

Photocatalysts typically consist of semiconducting materials with electrons in the valence band that, in the presence of photons with sufficient energy, undergo transitions toward the conductive level, thus creating highly reactive electron (e^–^) – hole (e^+^) pairs. These charge carriers induce a redox reaction with several molecules adsorbed on their surface, enabling, among others the degradation of organic pollutants in both liquid and gas-phase environments or the sustainable production of energy.^970^ Besides a source of light, photocatalysis usually requires heat as additional driving force, which contributes to the electron transfer processes to initiate and accelerate the reaction (photothermal catalysis).^975,976^

The encapsulation of photoactive materials within CNTs provides enhanced properties to the catalytic system.^977^ A variety of reports mostly involve improved oxidation reactions either for the degradation of organic pollutants or the selective formation of alcohols,^956^ ketones, using metals and metal oxides confined within CNTs. Their high surface area allows the loading of their interior with a large amount of catalyst, thus increasing the active sites to enhance the catalytic activity. Moreover, the carbon-based honeycomb lattice walls have high electron storage capacity, which increases the ability of the system to accept photon-excited electrons and therefore prolongs the electron–hole recombination times.^977^ Confined titania showed improved performance for both bacterial disinfection^953^ and the catalytic oxidation of propylene by H_2_O_2_.^955^ By encapsulating TiO_2_ clusters into DWCNTs, up to an 8-fold increase in the catalytic oxidation of propylene was observed compared to titania-decorated DWCNTs. The authors attributed these results to the small size of the titania clusters formed inside the DWCNTs and the strong electron transfer between the catalyst and nanoreactor. Cui et al. evaluated the catalytic activity of CdS filled CNTs in the degradation of methylene blue (MB).^952^ The material showed enhanced adsorption of the organic dye and improved catalytic performance (2.5 times) compared with pure CdS.

Organic dyes have emerged as alternatives to transition metals and their oxides, which usually require high-energy (UV) radiation to promote photocatalytic reactions. Nevertheless, these molecules are often sensitive to high-temperatures and radiation exposure and need to be anchored to stable platforms to prolong their lifetime. CNTs are suitable supports in this regard, as it has been reported by González-Muñoz et al., who enhanced the photocatalytic activity of 10-phenylphenothiazine (PTH) by confining the material within SWCNTs. The authors obtained an efficient and easily recoverable hybrid formed by 8% of PTH encapsulated within the carbon tubular nanostructure, which demonstrated a high activity for hydrogenation reactions catalyzed by visible light.^958^

#### 8.5.4 Magnetocatalysis

When catalysts formed by magnetic elements are encapsulated inside CNTs, a magnetic field, which often converts to heat, can be used as a driving force to promote catalytic reactions or in a further step during the catalytic process. External magnetic stimuli (which can be easily tuned) can be used to raise the *T* of the system and activate the sites involved in the catalytic reaction, thus improving the efficiency of the system. The most straightforward application of this approach involves using a magnetic field for the separation and recovery of CNTs filled with magnetic compounds or NPs containing ferromagnetic or superparamagnetic elements, such as Fe, Ni, and Co, once the reaction has proceeded.^960,961^ This enables the efficient removal of the catalyst, thus reducing environmental impacts owing to the efficient recycling of the material.^326,978^ Similar to other catalytic approaches, a synergistic effect and positive contribution are observed when magnetic catalysts are encapsulated within CNTs. The hosting tubes protect the magnetic particles from oxidation or degradation and dissipate heat and electron transfer. Moreover, they can be functionalized and targeted to obtain biocompatible materials for hyperthermia and drug delivery.

### 8.6 Sensors

This section reviews recent advances in sensor technologies based on filled CNTs, highlighting how endohedral functionalization enables enhanced sensitivity, selectivity, and operational stability across different sensing modalities (Table 12). While CNTs are already recognized as outstanding materials for next-generation sensors due to their exceptional physical, chemical, electrical, mechanical, and optical properties, the incorporation of functional species inside their hollow cavities introduces additional degrees of freedom for sensor design.

**Table 12:** Filled CNTs for Sensor Applications

Sensor Type	Type of NT	Filler Material	Target Analyte	Detection Mechanism	Key Performance parameters	Advantages	References
Magnetic Sensor	MWCNTs	Fe	Magnetic stray fields (MFM)	Magnetic force microscopy.	High capability to determine magnetic stray field gradients independent of domain size.	Novel monopole-like magnetic probe.	^984^
EC Sensor	MWCNTs	Ag-filled MWCNTs	Free cyanide ions (CN^–^)	EC (Nernstian).	Range: 21 nM–0.1 M; LDL: 13 nM; Response <2 min	High reproducibility, wide linear range, stable performance.	^985^
	MWCNTs	Ni–Co alloy NWs	Glucose	EC (redox-based, non-enzymatic).	Sensitivity: 0.695 mA mM^–1^ cm^–2^; LDL: 1.2 μM; Linear range: 5 μM–10 mM.	High selectivity, reliability in human serum samples.	^311^
	MWCNTs	Prussian Blue NPs	Glucose	EC (enzyme-like catalysis).	High peroxidase-like activity; efficient glucose quantification.	Enhanced catalytic and sensing activity.	^986^
	MWCNTs	NiSe_2_	Dopamine	EC oxidation of dopamine.	High sensitivity of 19.62 μA μM^–1^ cm^–2^; broad linear range of 5 nM–640 μM.	Strong signal amplification, high catalytic activity, improved selectivity, structural stability.	^987^
Gas Sensor	MWCNTs	V_2_O_5_	CH_4_	Electrical (charge transfer).	Sensitivity: 1.5%; Improvement over unfilled CNT (138 s/234 s/0.5%).	RT operation; enhanced response due to hybridization (V–O–C orbital).	^446^
	SWCNTs	Ni(acac)_2_	NO_2_ gas	Electrical (charge transfer).	Stable, reversible, enhanced sensitivity.	Improved charge transfer from filler to adsorbed molecules.	^988^
	SWCNTs	S	NO_2_	Electrical (interface interactions).	Increased sensitivity and stability.	Enhanced interface interaction with S fillers.	^989^
	SWCNTs	S and P-based compounds	NO_2_	Changes in electrical conductivity/resistance due to interaction of NO_2_ with filled SWCNTs; enhanced charge-transfer effects from S/P fillers.	Real-time detection; improved sensitivity (specific values require article data).	High sensitivity, real-time response, enhanced selectivity from S/P filling, stable operation in air.	^90^
	DWCNT	SnO_2_	Tridecane	Changes in electrical resistance/conductivity at RT; adsorption-induced charge transfer enhanced by filler material.	RT operation; improved sensitivity (exact values require article data).	Low-power operation (RT), enhanced sensitivity to VOCs, improved selectivity.	^990^
Optical Sensor	SWCNTs	FeCp_2_	General environment/chemical sensing	Optical (PL modulation).	Up to 3× NIR PL enhancement; Chirality-selective.	Charge transfer neutralizes *p*-type doping; structure-dependent.	^568^
	Semiconducting and metallic DWCNTs	Single Versus Double Wall CNTs	Molecular identity/conformation	Optical (PL spectral shifts via dielectric modulation).	Shifts correlate with exciton binding energy changes.	Sensitive to dielectric environment and guest molecule.	^994^
	SWCNT	DNA-wrapped SWCNT + MB	TMV RNA; Biotin-binding proteins	FRET.	Up to 150% fluorescence increase on hybridization.	Real-time, reversible sensing; specific and tunable.	^995^

CNTs exhibit a high specific surface area and remarkable charge-transport properties, making them extremely sensitive to subtle changes in their surrounding environment.^979^ As a result, they efficiently transduce molecular recognition events into measurable electrical or optical signals. Most CNT-based sensors reported to date rely on EC transduction mechanisms,^980^ with the overarching goal of achieving high selectivity, sensitivity, and reproducibility.^981^

In practical applications, CNTs are often employed as hybrid materials, combined with NPs, biomolecules, or polymers to enhance analyte recognition. Numerous reviews have documented CNT-based hybrid transducers for the detection of several analytes, mainly gases and biomolecules.^696,980,982^ Despite this progress, endohedral functionalization, where active species are confined within the CNT interior, remains far less explored than conventional exohedral approaches.^696^ Recent studies on fullerene- and nanocapsule-based hybrids have underscored the importance of confinement effects in bioanalytical sensing,^983^ pointing to the largely untapped potential of endohedral strategies for creating new classes of CNT-based sensor platforms.

#### 8.6.1 Magnetic Sensors

Iron-filled MWCNTs were reported as novel monopole-like probes for quantitative magnetic force microscopy (MFM).^984^ These Fe-filled CNT (FeCNT) sensors demonstrated an exceptional ability to determine magnetic stray-field gradients independently of domain size. Unlike conventional MFM probes, which often possess complex and poorly defined magnetic structures, individual FeCNTs behave as nanomagnets with well-defined and stable magnetic properties. By combining the mechanical resilience and nanoscale dimensions of CNTs with the strong ferromagnetic response of encapsulated iron, FeCNT-based probes enable a simplified magnetic description. In practice, the CNT can be treated as an extended magnetic dipole, with the tip region closest to the sample surface effectively acting as a magnetic monopole during imaging. This well-defined magnetic behavior represents a clear advantage for the quantitative interpretation of MFM data and illustrates the unique opportunities offered by endohedral filling in magnetic sensing applications.

#### 8.6.2 Electrochemical Sensors

EC sensing represents the most widely explored application of filled CNTs. A representative example is the use of silver-filled MWCNTs as active sensing elements for the determination of free cyanide ions (CN^–^) in aqueous solutions.^985^ The corresponding Ag-filled MWCNT-based electrode exhibited a near-Nernstian response of 59.8 ± 0.3 mV decade^–1^, indicating predictable potentiometric behavior over a wide linear detection range (21.0 nM–0.1 M) and a low detection limit (LDL) of 13.0 nM. The sensor responded rapidly (<2 min), maintained stable performance for up to three months, and showed excellent selectivity against common interfering anions, demonstrating the practical advantages of endohedrally filled CNT electrodes.

Beyond ion sensing, filled CNTs have also been successfully applied to biomolecule detection. For instance, Ni–Co alloy NW–filled MWCNTs were employed as non-enzymatic EC probes for glucose detection.^311^ MWCNT/Ni–Co-modified GCE exhibited enhanced redox activity and high peak currents, attributed to the synergistic effects of the bimetallic nanostructures, large surface area, and high electrical conductivity of the CNT framework (Figure 44). Similar strategies have been reported for other filled-MWCNT-based glucose sensors, which achieved high sensitivity (0.695 mA mM^–1^ cm^–1^), LDL (1.2 μM), and wide linear ranges (5 μM–10 mM), along with excellent selectivity in human serum samples.^986^

**Figure 44:**
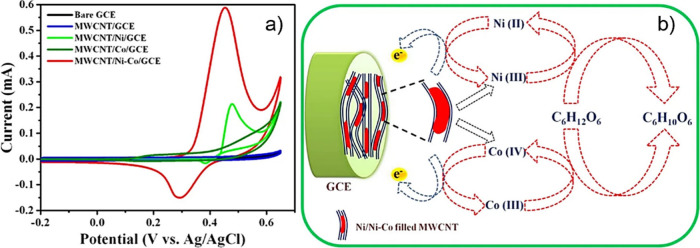
Evaluation of Ni–Co alloy NWs-filled MWCNTs as non-enzymatic EC sensor probes for the detection of glucose. a) CVs of studied GCE in 0.1 M NaOH in the presence of 2 mM glucose at a scan rate of 20 mV s^–1^ and b) mechanism involved in the electrooxidation of glucose at MWCNT/Ni–Co.^311^ Reproduced with permission from ref 311. Copyright 2016, Springer Nature.

Extending these concepts to neurotransmitter detection, Singh et al. reported a highly efficient non-enzymatic dopamine sensor based on metal chalcogenide–filled CNTs.^987^ In this case, nickel selenide (NiSe_2_) nanostructures were formed inside CNTs via a one-step, environmentally friendly CVD process. The resulting NiSe_2_-filled CNT composites exhibited markedly enhanced electrocatalytic activity toward dopamine oxidation, with high sensitivity (19.62 μA μM^–1^ cm^–2^), a very low detection limit, and a broad linear range from 5 nM to 640 μM. These improvements were attributed to synergistic interactions between the electrocatalytic NiSe_2_ phase and the highly conductive CNT network, which together facilitate efficient charge transfer. High selectivity in the presence of interfering species further highlights the promise of chalcogenide–filled CNTs for non-invasive biochemical sensing.

#### 8.6.3 Gas Sensors

Gas sensing is another area where filled CNTs have demonstrated significant advantages, particularly through the modulation of charge-transfer interactions and electronic structure.^90^ For example, vanadium oxide (V_2_O_5_) solution-filled MWCNTs were proposed for methane-gas detection.^446^ Compared with unfilled CNTs, these hybrids showed substantially faster response and recovery times at RT, along with a 3-fold increase in sensitivity. At room temperature, these hybrids exhibited a substantial improvement in response time, decreasing from 138 s for unfilled MWCNTs to 16 s for V_2_O_5_-filled ones. The recovery time was similarly improved from 234 to 120 s, and the sensitivity increased from 0.5% to 1.5%. The improvement in the sensitivity to methane gas was attributed to the increased density of states around the Fermi level of the hybrid material, which resulted from the hybridization between the V-3d and O-2*sp* orbitals with the C-2*sp* orbitals. Moreover, the improved response and recovery times of V_2_O_5_-filled MWCNT-based sensor were attributed to the weak physisorption interactions with the gas compared to those of the non-encapsulated metal oxide.

Considerable attention has also been devoted to NO_2_ sensing using filled SWCNTs, where achieving both high sensitivity and rapid recovery at ambient conditions remains challenging. Early studies examined metallicity-sorted SWCNTs filled with nickel precursors that were converted into Ni nanoclusters.^965^ By tailoring both the electronic character of the SWCNTs and the nature of the filling, reversible NO_2_ adsorption was achieved at RT. Photoemission spectroscopy revealed that semiconducting SWCNTs filled with Ni nanoclusters exhibited Fermi-level pinning within the bandgap, resulting in enhanced sensitivity and rapid recovery, whereas metallic SWCNTs displayed slower and incomplete desorption due to stronger chemisorption.^988^

Interface-driven sensitivity was further elucidated by studies comparing sulfur-coated and sulfur-filled SWCNTs. In sulfur-filled SWCNTs, the encapsulated sulfur acts as a charge reservoir, enhancing charge transfer to adsorbed NO_2_ molecules and significantly increasing sensitivity. The combination of sulfur filling and external sulfur coating yielded sensors with excellent NO_2_ sensitivity (1 ppb–10 ppm), improved recovery, strong selectivity, and long-term stability under humid conditions.^989^ Other examples include encapsulating sulfur, phosphorus and their binary compounds within SWCNTs for NO_2_ sensing.^90^ Thin-film sensors fabricated from these hybrids exhibited dramatically improved NO_2_ detection at 150 °C, with detection limits as low as 2–7 ppb and response times below 1 min (Figure 45 a)). In these cases, the authors attributed the improvement in the device sensitivity, and thus the sensor response, to the supplemental charge transfer from the core through the NT wall to the adsorbed molecules. DFT calculations indicated that the NO_2_ adsorption energies decreased by 0.12–0.32 eV relative to those of the unfilled SWCNTs, explaining the faster recovery while preserving strong sensor responses. These sensors also demonstrated operational stability for over 12 months and excellent selectivity in the presence of H_2_O, CO, CO_2_, and NH_3_, confirming that the encapsulated P–S phases create chemically favorable environments for NO_2_ adsorption (Figure 45 c–f)).

**Figure 45:**
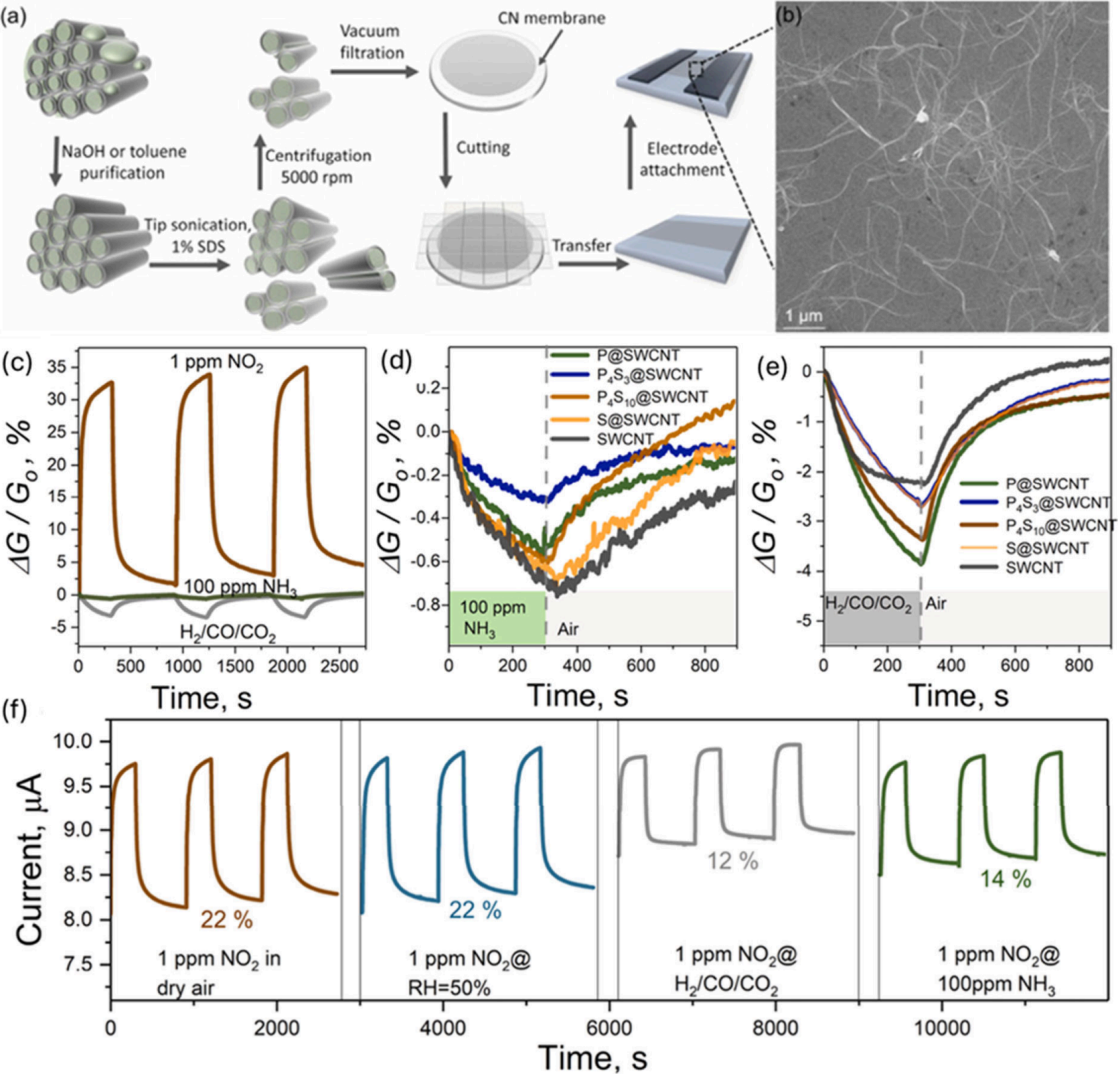
a) Schematic illustration of the fabrication process of the SWCNT-based sensors. b) SEM image of the SWCNT film c) Response of the P_4_S_10_@SWCNT sensor at 150 °C to synthetic air containing 1 ppm of NO_2_, 100 ppm of NH_3_, and 3.75 vol % H_2_/CO/CO_2_. d) Comparison of sensor response of empty and filled SWCNT toward e) 100 ppm of NH_3_ and c) H_2_/CO/CO_2_ exposure. f) Response of the P_4_S_10_@SWCNT sensor to 1 ppm of NO_2_ in synthetic air and when water, carbon oxides, or ammonia were present in the gas mixture. Reproduced with permission from ref 90. Copyright 2024, Elsevier.^90^

Finally, filled DWCNTs have been explored for volatile organic compound (VOC) detection.^990^ SnO_2_-filled DWCNTs exhibited significantly enhanced sensitivity and faster response toward tridecane compared with pristine CNTs. Specifically, the hybrid sensors showed a 63% higher response and a faster detection time of approximately 20 s, compared to approximately 75 s for the unfilled reference. These results highlight the versatility of endohedral filling strategies for gas sensing under ambient conditions.

#### 8.6.4 Optical Sensors

Optical sensing based on filled CNTs has emerged as a rapidly advancing strategy that leverages the interplay between the electronic structure of the NT and the optical response of encapsulated or interacting species.^991^ In these cases, sensing relies on modulating PL or energy-transfer processes originating from the CNT itself, guest molecules, or nearby fluorophores, in response to physical or chemical changes within or around the CNT (Table 12).^992^ A key mechanism involves PL quenching or enhancement in semiconducting SWCNTs.^189^ Guest species confined within the CNT’s cavity can facilitate charge or energy transfer, resulting in PL quenching, or stabilize excitonic states, enhancing emission.^993^ These responses are especially sensitive to redox-active, magnetic, or polarizable fillers. Pichler et al. showed that encapsulation of FeCp_2_ molecules inside SWCNTs leads to a significant and chirality-selective enhancement of their NIR PL.^568^ This enhancement, observed to be up to 3-fold for CNTs with chiralities such as (8,6) and (9,5), is attributed to electron charge transfer from the FeCp_2_ to the CNT, which neutralizes *p*-type doping effects caused by surfactants and oxidative processing. The degree of enhancement was found to be dependent on the CNT diameter, with a maximum for CNTs with a 0.9 nm diameter, suggesting structural influences on the charge interaction. First-principles calculations support the proposed mechanism, highlighting the ability of internal molecular filling to modulate the optical properties of SWCNTs for potential applications in sensing and optoelectronics. Another important sensing mechanism involves dielectric screening-induced spectral shifts. Encapsulated materials modify the local dielectric constant, altering the exciton binding energies and leading to measurable shifts in the PL spectra. These shifts can indicate the identity or conformation of guest molecules, as well as the solvent or environmental conditions.^994^ Additionally, fluorescent guest molecules, such as organic dyes, porphyrins, or lanthanide complexes, retain or exhibit altered emission when confined inside CNTs. Spatial confinement and π-electron interactions influence the quantum yield, emission wavelength, and lifetime of these probes, enabling the detection of chemical transformations, ion binding, and photoreactivity. Finally, Förster Resonance Energy Transfer (FRET) can occur between encapsulated fluorophores and CNTs. When spectral overlap and spatial proximity conditions are satisfied, dynamic energy transfer enables reversible, real-time sensing of molecular binding events and environmental changes, making this approach particularly useful in biosensing.^992^ Recently, Landry et al. developed a DNA-wrapped SWCNT sensor system enhanced by the redox dye MB, which enables controlled fluorescence quenching via FRET.^995^ Upon hybridization with complementary DNA, MB is displaced beyond the ∼6.8 nm FRET radius, resulting in up to a 150% increase in NIR fluorescence. This nanostructured sensor was successfully used to detect tobacco mosaic virus (TMV) RNA in infected plants, with clear signal differentiation from healthy controls. Additionally, the nanoplatform was explored to detect biotin-binding proteins demonstrating its versatility for both nucleic acid and protein targets. These results demonstrate a tunable, specific, and broadly applicable SWCNT-based nanosensor platform for biomedical and environmental applications.

### 8.7 Nanoelectronics

This section discusses how endohedral filling of CNTs enables precise electronic structure engineering and unlocks new functionalities in nanoelectronic and optoelectronic devices. By confining electron donors, acceptors, or functional nanostructures inside SWCNTs, it becomes possible to modulate charge transfer, doping type, and carrier dynamics in a stable and controllable manner, overcoming many limitations associated with external functionalization.

One of the most extensively studied nanoelectronic applications of filled SWCNTs is the creation of air-stable p–n junctions, which serve as fundamental building blocks for electronic devices and circuits. Such junctions are typically fabricated through partial or piecewise filling of SWCNTs with electron donors and acceptors. Systematic studies have shown that filling SWCNTs with halides of 3*d*-, 4*d*-, 5*d*- and 4*f*-metals, as well as Ga chalcogenides, which have larger work function than the pristine CNTs, results in *p*-doping of SWCNTs accompanied by charge transfer from the CNT walls to the incorporated substances and a lowering of the Fermi level of SWCNTs by ∼0.3–0.4 eV.^996^ The magnitude of this Fermi level shift depends on both the halogen species and the metal atom involved. For halides of 3*d*- and 4*d* metals, the shift increases in the order I–Br–Cl, while within the *3d*-metal series it decreases from Mn to Zn. In many cases, filling of SWCNTs with MX of 3*d*-, 4*d*-, and 4*f*- group metals leads to the formation of chemical bonds between the CNT walls and the confined metal atoms driven by hybridization between π-orbitals of carbon and *d*-orbitals of the metal. In contrast, no such bonding is observed for SWCNTs filled with chalcogenides of Ga, Bi, or Sb, indicating a weaker electronic coupling.

Complementary *n*-type doping can be achieved by encapsulating compounds with smaller work functions than pristine SWCNTs. For example, filling with RbI induces charge transfer from the encapsulated salt to the CNT walls, shifting the Fermi level upward by approximately 0.2 eV.^997^ Similar, though weaker, *n*-doping effects have been reported for MCp_*x*_-filled SWCNTs, with Fermi-level shifts on the order of 0.1 eV.^216,284,576,998^ By contrast, filling with TMCs of Sb and Bi, whose work functions are similar to those of pristine SWCNTs, does not significantly alter the electronic structure. These doping effects have been comprehensively characterized using Raman spectroscopy, NEXAFS, photoemission spectroscopy, XPS, ultraviolet photoelectron spectroscopy (UPS), and OAS, providing consistent evidence for controlled electronic modulation via endohedral filling.

Building on these doping strategies, several experimental demonstrations of p–n junctions based on filled SWCNTs have been reported. Early examples include partially Fe-filled SWCNTs fabricated via solution methods, which exhibited rectifying behavior characteristic of p–n diodes.^999^ More advanced approaches employed controlled plasma irradiation to achieve piecewise cofilling of SWCNTs with electron donors (Cs) and acceptors (I or C_60_), enabling precise spatial control over the doping profile.^1000^

A representative device based on Cs/I cofilled SWCNTs is shown in Figure 46, which presents the source–drain current as a function of the applied backgate voltage (*I*_DS_–*V*_G_) of the FET.^1000^ The device was operated at ambient *T* and was first irradiated for 2 h with I^–^ ions. Subsequently, different irradiation times with Cs^+^ ions were systematically investigated. After the initial I^–^ ion irradiation, when the Cs^+^ irradiation time was zero, the characteristic *p*-type *I*_DS_–*V*_G_ was evident (Figure 46 a)). After an additional 5 h of exposure to Cs^+^ irradiation, the *I*_DS_–*V*_G_ characteristics were reversed from *p*-type to *n*-type features (Figure 46 (c)). This complete reversal demonstrates the potential to precisely tune the doping level via the applied Cs^+^ irradiation time. In particular, in a balanced intermediate regime where the Cs^+^ and I^–^ ion dosages were almost the same, a unique hump structure in the *I*_DS_–*V*_G_ dependence, which is characteristic of a *p-n* junction, was observed (Figure 46 b)).^1000^ The current rectification ratio is shown as a function of Cs^+^ irradiation time in Figure 46 d). It is defined as the absolute the ratio of the maximum to minimum source–drain current (|*I*_DSmax_/*I*_DSmin_|) at a given *V*_G_. Clear current-rectifying features with ratios between 10^3^ and 10^4^ could be achieved exclusively when roughly equal amounts of cesium and iodine were irradiated (Figure 46 d)). The high rectifying ratio is also concomitant with the *p*-*n* junction characteristic in Figure 46 b). It is worth mentioning that despite the volatility of cesium and iodine, the devices made from Cs/I@SWCNT were air-stable (Figure 46 e)). This is readily explained by the fact that the ions are trapped inside SWCNTs and are therefore stable and well-shielded from the environment.^1000^ In a related study, SWCNTs were filled with only Cs by advanced plasma irradiation to achieve different levels of *n*-type doping. This resulted in direct control of the transport properties of Cs@SWCNT.^1001^

**Figure 46:**
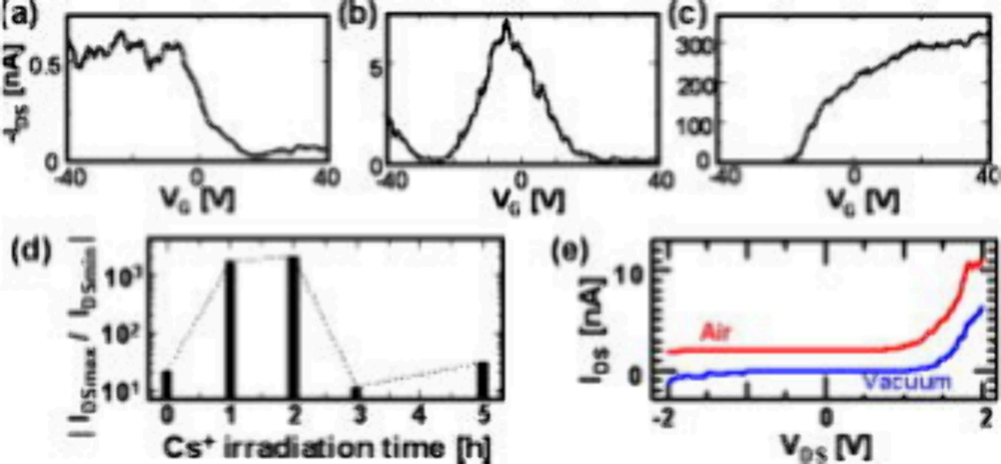
a–c) *I*_DS_–*V*_G_ characteristics of Cs/I@SWCNT. The Cs^+^ irradiation times are 0 h (*V*_DS_ = −0.5 V) a), 2 h (*V*_DS_ = −2 V) b), and 5 h (*V*_DS_ = −1 V) c). The I^–^ irradiation time was always 2 h. d) Current rectification ratio |*I*_DS max_/*I*_DS min_| versus Cs^+^ irradiation time. e) *I*_DS_–*V*_DS_ characteristics of Cs/I@SWCNT under vacuum and ambient conditions (*V*_G_ = 40 V). The data in ambient conditions are offset for clarity.^1000^ Reproduced with permission from ref 1000. Copyright 2009, AIP Publishing.

Beyond conventional *p–n* junctions, endohedral filling also enables more complex electronic functionalities, including ambipolar transport and bandgap engineering. Fullerene- and endohedral fullerene-filled SWCNTs have been explored as active channels in ambipolar field-effect transistors (FETs), where both electron and hole conduction can be accessed.^493,1002,1003^ In particular, Dy@C_82_-filled SWCNTs exhibited *T*-dependent switching of conductivity type, transitioning from *p*-type behavior at RT to *n*-type conduction at lower *T* (∼265 K).^501,1004^ These findings illustrate how confined molecular species can modulate carrier populations and transport mechanisms in a reversible and controllable manner.

Filled SWCNTs also offer new opportunities for controlling ultrafast carrier dynamics, which are critical for optoelectronic and energy-conversion applications. Burdanova et al. explored ultrafast electron transport in films composed of both pristine and P-filled SWCNTs through the measurement of their transient photoconductivity using an optical pump–terahertz probe technique (Figure 47 a–c)).^338^ This approach allows the tracking of the immediate response of electrons after photoexcitation on femtosecond time scales. Under femtosecond irradiation, the electrons in the CNT films reached *T* as high as 800 K (Figure 47 e–f)). Both the filled and unfilled SWCNT films exhibited a transition between positive and negative photoconductivity, which arises from the interplay between metallic CNTs, which typically contribute to negative photoconductivity, and semiconducting CNTs, which tend to contribute to positive photoconductivity. This crossover highlights the complex mixture of optical responses within these films. Importantly, P encapsulated inside SWCNTs produces a stable shift in the Fermi level of the SWCNTs. This internal doping mechanism significantly enhances hot carrier generation processes, increasing the carrier multiplication efficiency up to 1.5, surpassing even the already strong intrinsic efficiency observed in empty semiconducting SWCNTs. Because the P atoms are protected within the NT interior, the doping effect remains stable over time, offering a reliable and tunable method for modifying the electronic properties. These findings demonstrate that P-filled SWCNTs are highly promising for optoelectronic applications that require efficient carrier generation, such as photodetectors and solar cells.

**Figure 47:**
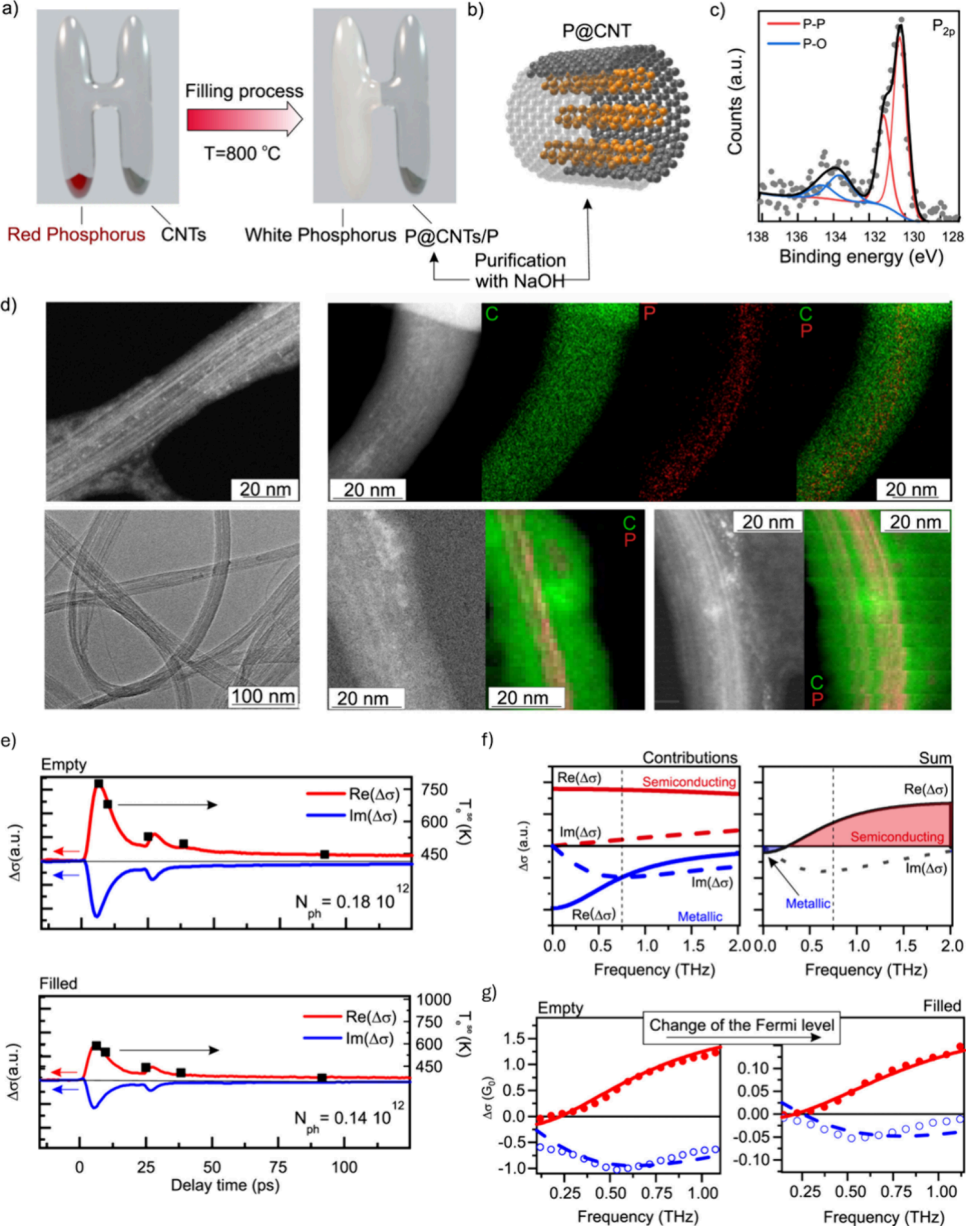
a) Schematic illustration of the synthesis process of composite nanomaterials from SWCNTs and red P. b) The structure of filled CNT after purification of surface P. Orange atoms indicate double chains inside SWCNTs. Hydrogen atoms are not shown. c) XPS P_2p_ spectra of P@SWCNTs confirming the filling of CNTs with C–P. d) Left to right (top): HRTEM images of a bundles of filled SWCNTs and EDX maps of C (green) and P (red) atoms and their combination. Left to right (bottom): TEM image of CNTs network with EDX maps of bundles, showing formation of chains inside SWCNTs. e) The measured real and imaginary parts of the photoconductivity at a frequency of 1 THz as a function of pump–probe delay time. Dots correspond to the electronic *T* of semiconducting SWCNTs which well fit the observed photoconductivity response for both metallic and semiconducting CNTs. f) The modeled contributions of metallic and semiconducting SWCNTs to total photoconductivity shown in the right. g) The photoinduced conductivity spectra of empty and filled CNTs (dots) at 3 ps pump–probe delay time and the corresponding fit (lines) obtained from the semiclassical Boltzmann model.^338^ Reproduced with permission from ref 338. Copyright 2024, Elsevier.

Recently, the encapsulation of topological insulator materials inside SWCNTs has opened additional avenues for nanoelectronic applications. Sloan et al. reported the filling of SWCNTs with bismuth selenide (Bi_2_Se_3_) and bismuth telluride (Bi_2_Te_3_), with detailed structural characterization performed using HRTEM and ADF-STEM.^377^ In Bi_2_Se_3_-filled SWCNTs with diameters of approximately 2.3 nm, the encapsulated material formed an alloy close to Bi_0.7_Se_0.3_ rather than the expected bulk stoichiometry. In contrast, Bi_2_Te_3_-filled SWCNTs exhibit a variety of microstructures, most of which are derived from the rhombohedral R-3mH phase of Bi_2_Te_3_ crystals. The observed stoichiometries range from nearly bulk-like Bi_2_Te_3_ to more Te-rich phases, such as Bi_6_Te_7_ and BiTe, with the specific composition being strongly influenced by the diameter of the CNTs. Depending on the proportions of these ordered and disordered phases, the filled CNTs may introduce a broad spectrum of electronic states into the composite material, although the overall variation in the band gap is expected to remain relatively small.

Despite this structural diversity, Raman spectroscopy revealed only minor perturbations of the SWCNT electronic structure, indicating that the small band gaps of Bi_2_Se_3_ and Bi_2_Te_3_ do not strongly disrupt the CNT states. These findings suggest that chalcogenide-filled SWCNTs offer potential pathways toward hybrid nanoelectronic and thermoelectric devices.

Table 13 highlights current reports on filled CNTs for nanoelectronic applications.

**Table 13:** Filled CNTs Reported for Nanoelectronic Applications

Nanolectronic applications	Type of NT	Filler Material	Filling Method/structure	Key findings	Reference
*n*-type doped SWCNT channel for FETs	SWCNT	RbI	Vapor-phase filling.	RbI acts as an e^•^ donor, shifting SWCNT Fermi level.	^997^
Tunable FETs and spintronic systems	SWCNT	MCp_*x*_ (FeCp_2_, CoCp_2_, NiCp_2_)	Capillary or vapor-phase encapsulation.	Systematic study of charge transfer and tunable properties depending on MCp_*x*_ type.	^998^
*p*–*n* junction diode	SWCNT	Fe NPs	In situ encapsulation.	Fe-filled SWCNTs form air-stable *p*–*n* junction diodes.	^999^
Nanoscale *p*–*n* junction/diode	SWCNT	Cs/I and Cs/C_60_	Plasma ion irradiation process.	Fabrication of *p*–*n* junctions using donor- and acceptor-filled SWCNTs.	^1000^
Ambipolar field-effect transistor (FET)	SWCNT	Gd@C_82_ metallofullerenes	Peapod structure.	Exhibited ambipolar FET behavior; Gd@C_82_ alters carrier mobility.	^1002^
Quantum wire/1D hybrid nanostructure for memory and quantum transport	SWCNT	(Gd@C_82_)n (metallofullerene chain)	Peapod structure.	Showed modified electronic density and conduction mechanism via hybridization effects.	^1003^
*T*-sensitive FET/sensor	SWCNT	C_60_, fullerenes and metallofullerene	Peapod structure.	Conductance shows *T*-dependent semiconducting-to-metallic transition.	^1004^
Nanoelectronic and thermoelectric applications	SWCNT	Bismuth chalcogenides	Melting filling (capillary-driven filling of SWCNTs from the melt).	Demonstrates formation of solid solution and homologous 1D nanostructures inside SWCNTs, relevant for tuning electronic and transport properties.	^377^
Hot-carrier optoelectronics, photodetectors, energy-conversion nanodevices	SWCNT with a diameter ranging from 1.6 to 3.0 nm	P	Vapor-phase filling.	Demonstrates the possibility of stable hot carrier multiplication in filled SWCNTs, indicating enhanced carrier dynamics relevant for high-efficiency nanoelectronic and optoelectronic devices.	^338^

### 8.8 Spintronics

Endohedral metallofullerene molecules, such as Dy_*n*_Sc_3‑n_N@C_80_ (*n* = 1, 2), represent single molecule magnets (SMMs) that function as isolated magnets owing to their large magnetic anisotropies and slow relaxation of magnetization. They are considered promising materials for applications in molecular spintronics.^1005^ By encapsulating SMMs inside SWCNTs, the magnetic properties of SMMs are combined with the electronic properties of the SWCNTs. SMMs form a quasi 1D arrangement inside SWCNTs while being protected from the environment by the CNT walls. Upon encapsulation, the neighboring intermolecular dipole–dipole interactions can be reduced, leading to enhanced SMM properties. The interaction of SMMs with SWCNTs affects their electronic and spintronic properties, resulting in giant magnetoresistance.^92,1005–1008^ Nakanishi et al. encapsulated an endohedral metallofullerene DySc_2_N@C_80_ inside purified SWCNTs with a narrow diameter distribution of 1.4 ± 0.1 nm to control and enhance the SMM properties. Figure 48 a) shows the structural models of the DySc_2_N@C_80_ molecule and filled SWCNTs. A HRTEM image of an individual CNT filled with SMMs is included in Figure 48 b).^1006^

**Figure 48:**
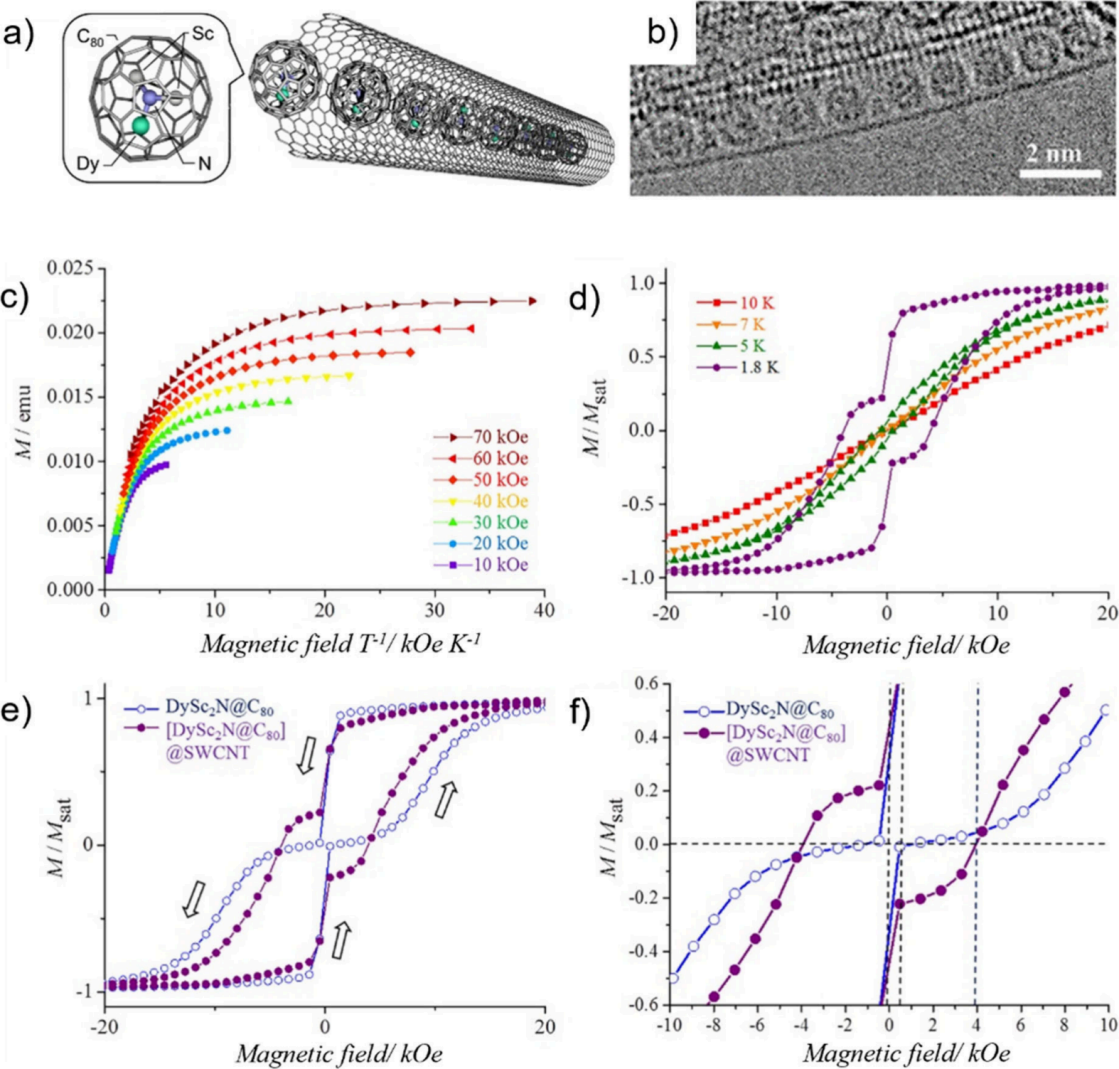
a) Structural models of the DySc_2_N@C_80_ molecule and filled SWCNTs. b) HRTEM image of an individual filled CNT showing the molecules within the SWCNT interior space. c) Magnetization versus magnetic field/*T* plots for DySc_2_N@C_80_@SWCNT in an applied magnetic field (H) ranging from 10 to 70 kOe. d) Magnetization divided by the saturation magnetization (M/M_sat_) plotted versus H for DySc_2_N@C_80_@SWCNT at *T* of 1.8, 5, 7, and 10 K. e) M/M_sat_ plotted versus H for DySc_2_N@C_80_ and DySc_2_N@C_80_@SWCNT at 1.8 K. Arrows show the direction of the measurements. f) Relaxation of the magnetization (Δ*m*/*m*_sat_) for DySc_2_N@C_80_@SWCNT at 2 K, with Δ*m*(*t*) = *m*(*t*) – *m*(*t* → ∞). Solid line corresponds to the best fit.^1006^ Reproduced with permission from ref 1006. Copyright 2018, American Chemical Society.

Static magnetic measurements were performed on free DySc_2_N@C_80_ and DySc_2_N@C_80_@SWCNT to determine the effects of the encapsulation of SMMs in SWCNTs on their magnetic properties. The magnetization was measured as a function of the magnetic field (*H*) and *T*. Figure 48 c) shows the magnetization data for DySc_2_N@C_80_@SWCNT (magnetization versus *H*/*T*) at different values of H ranging from 10 to 70 kOe.^1006^ The saturated magnetization values depend on the applied *H*. These values are equal to those for free DySc_2_N@C_80_, which indicates that the Dy ion in DySc_2_N@C_80_ still has uniaxial magnetic anisotropy and/or a low-lying excited state. Figure 48 d) shows magnetization divided by the saturation magnetization (*M*/*M*_sat_) plotted versus *H* plots for DySc_2_N@C_80_@SWCNT at *T* of 1.8, 5, 7, and 10 K. Clear magnetization loops were detected at *T* below 5 K, similar to those for DySc_2_N@C_80_. This suggests that the SMM properties of DySc_2_N@C_80_ are maintained when the molecules are encapsulated in SWCNTs. A comparison of the magnetization loops at 1.8 K for free DySc_2_N@C_80_ and DySc_2_N@C_80_@SWCNT shows that the stepwise hysteresis, a characteristic of SMM, is present in both samples (Figure 48 e)). However, upon inserting the molecules into SWCNTs, the coercivity increases from 0.5 to 4 kOe.^1006^

The slow relaxation of the magnetization of SMMs is a crucial parameter in spintronic applications. For free DySc_2_N@C_80_ endofullerene, a relaxation time of approximately 40 min for an undiluted sample was reported.^1006^ To compare the relaxation times for free DySc_2_N@C_80_ and DySc_2_N@C_80_@SWCNT, the time-dependent relaxation of the magnetization for DySc_2_N@C_80_@SWCNT was measured at different *T*.^1006^ The curve at 2 K in a zero field is shown in Figure 48 f). The relaxation occurred via a triple-exponential decay for DySc_2_N@C_80_@SWCNT, whereas it was double-exponential for DySc_2_N@C_80_. The fitting of the relaxation data showed that the relaxation time of the magnetization of DySc_2_N@C_80_@SWCNT increased to ∼94 min. This improvement in the SMM properties is caused by the suppression of quantum tunneling of magnetization due to dilution upon encapsulation within the interior space of SWCNTs.^1006^

Another class of SMMs is polynuclear metal complexes that can retain their magnetization behavior even in the absence of an external magnetic field.^1009^ Although the most famous members of this family are manganese-based magnets, such as Mn_12_Ac [Mn_12_O_12_(OAc)_16_(H_2_O)_4_],^1010^ Mn_6_,^1011^ or Mn_4_,^1012^ most of the current SMMs contain lanthanide ions.^1013^ These systems possess a unique set of properties, such as magnetic bistability, quantum coherence, and quantum tunneling of magnetization, which make them ideal candidates for a range of applications in molecular electronics, spintronics, and data storage devices. To develop devices based on these nanoscale units, integration at the macroscopic scale is still necessary. Therefore, CNTs can serve not only as a protective layer preventing degradation of the magnets, but also as encapsulation of SMMs within CNTs, which can be used to modulate their electronic properties through local arrangement and orientation of the magnets.

Reports on the encapsulation of SMMs within the internal cavities of CNTs are scarce. Khlobystov and co-workers^1005^ demonstrated that the filling of CNTs with SMMs was possible. The authors encapsulated Mn_12_Ac in MWCNTs using scCO_2_ as the carrier fluid. The presence of Mn atoms was confirmed by EDX, while TEM showed the presence of free-standing SMM molecules along the internal cavity of the CNT (Figure 49 b)). Magnetic characterization of the filled CNTs revealed that although the properties of the filling material were retained, the hosting platform offered an alternative route for the relaxation of magnetization.

**Figure 49:**
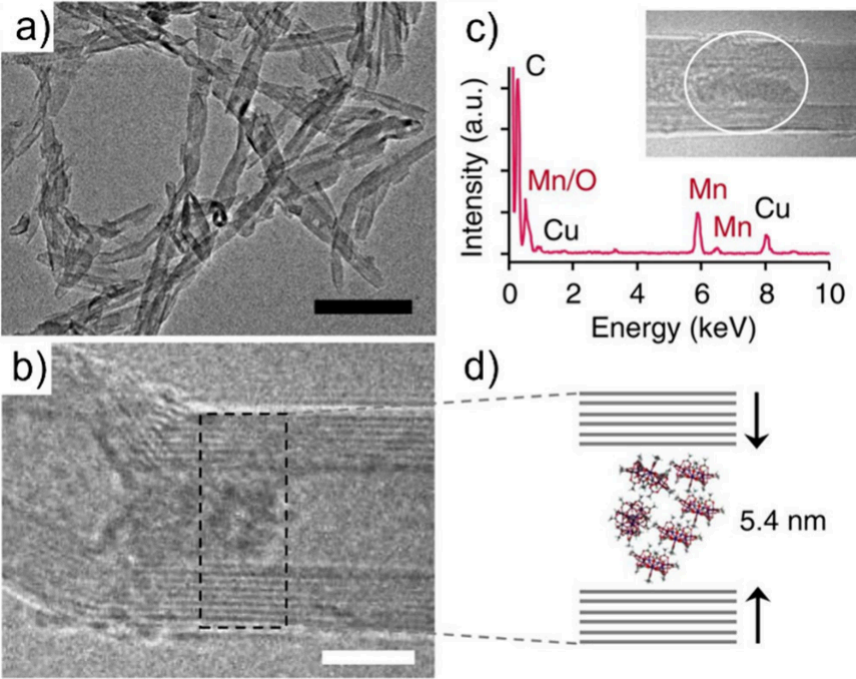
a–b) TEM images of MWCNT filled with Mn_12_Ac; (black scale bar corresponds to 100 nm; white scale bar to 5 nm. c) EDX spectrum of the selected area in the TEM image (inset, circled) confirms the successful encapsulation of Mn-containing molecules within the MWCNT. d) Schematic representation of the packing of Mn_12_Ac molecules in a MWCNT.^1005^ Reproduced with permission from ref 1005. Copyright 2011, Springer Nature.

POM, which have been demonstrated to be useful as molecular magnets, also exhibit catalytic activity to promote oxidation reactions and show promise as antiviral agents. A Lindqvist anion (W^VI^[W_6_O_19_]^2–^), a POM formed by a combination of six WO_6_ octahedra, has been successfully encapsulated within SWCNTs and DWCNTs.^1014^ Other attempts to encapsulate SMMs into SWCNTs resulted in the formation of hybrid structures with the magnet moiety located on the outer surface of the CNT, leaving them exposed to degradation under external ambient conditions.^1015–1017^

### 8.9 Magnetic Storage

SWCNTs filled with magnetic NPs can be used in magnetic storage devices.^1018^ In most cases, residues originating from the metal catalyst (usually Fe or Ni used for the synthesis of CNTs) pose a drawback and need to be removed from the sample. Nevertheless, considering the biocompatibility of Fe and taking advantage of its residual presence in a sample of SWCNTs,^1019^ Okotrub et al. filled sulfur within SWCNTs to induce the formation of FeS (1.7 nm < *d* < 1.9 nm). After encapsulation of sulfur, the sample was irradiated with a high-intensity polychromatic photon beam that promoted the migration of sulfur from the interior to the exterior, also inducing sulfur melting and increasing the concentration of sulfides. The authors also investigated the magnetic properties of nanohybrids, which were prepared using high *T* and high temperature-high pressure conditions^1020^ (Figure 50).

**Figure 50:**
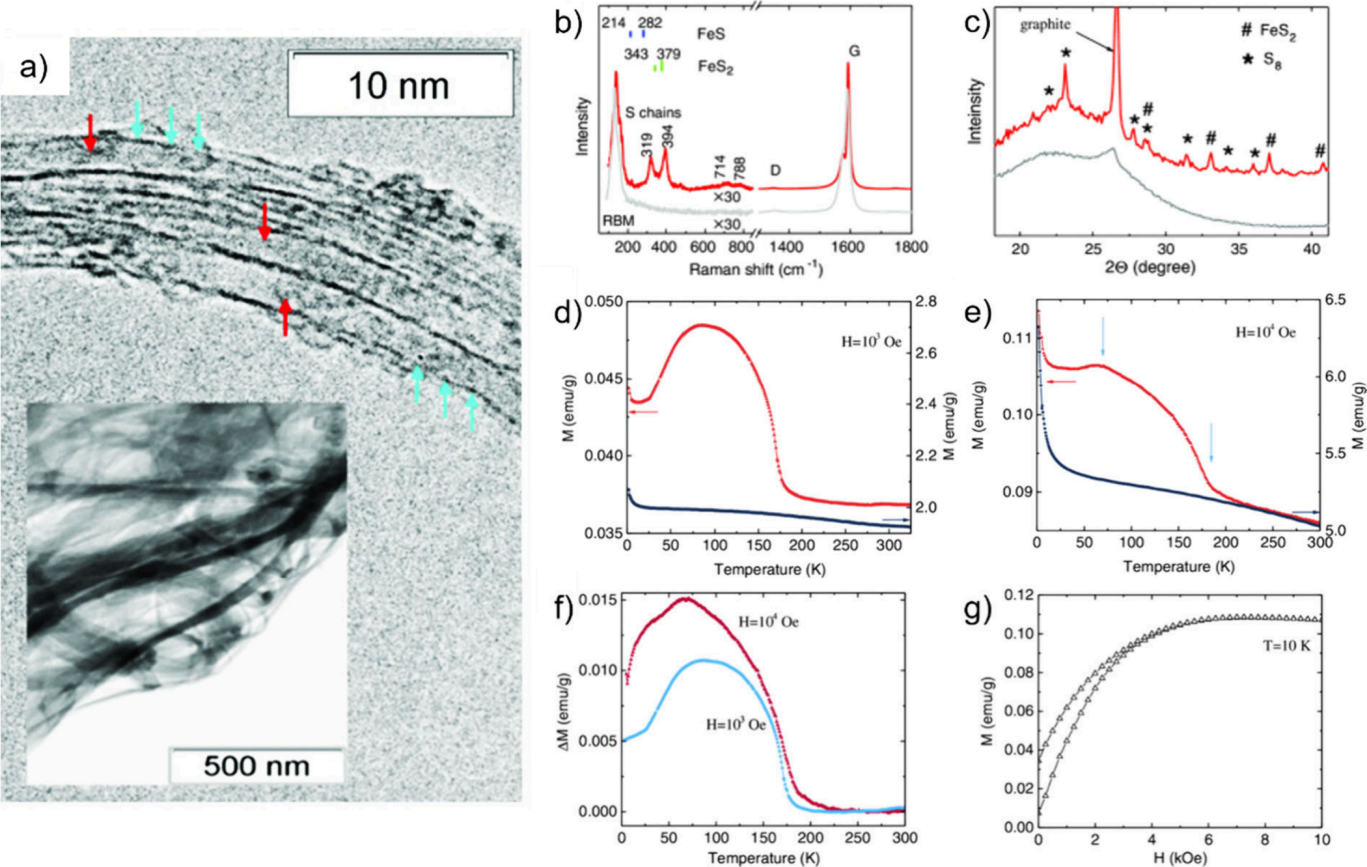
Morphology, structural, and magnetic characterization of SWCNTs filled with sulfur and iron sulfide (as prepared by a high *T* method). a) HRTEM image of SWCNTs filled with sulfur and iron sulfide. b) Raman spectra. c) XRD. ZFC magnetization data for pristine SWCNTs (gray) and sulfur-filled (red) SWCNTs at d) H = 1 kOe and e) H = 10 kOe. f) sulfur-related magnetization contribution. g) Hysteresis M­(H) curve measured at 10 K.^1020^ Reproduced with permission from ref 1020. Copyright 2020, John Wiley and Sons.

The structural characteristics and properties of sulfur-filled SWCNTs were strongly affected by the synthesis approach. While the high *T* treatments led to the encapsulation of both monoelemental sulfur and mostly iron sulfide NPs (pyrite), applying high pressure induced the release of the former Fe species present within the interior of the CNTs, and only sulfur was detected after synthesis (Figure 50 b–c)). As a consequence, more drastic variations in magnetization were observed for the first sample (high-*T* protocol), which presented a strong ferromagnetic-type behavior (below 175 K) and antiferromagnetic ordering (below 75 K), induced by the confinement of the iron sulfide NPs (Figure 50 d–g))

## 9 Disposal, Recyclability and Environmental Impact of Filled CNTs

A major concern when working with filled CNTs is their potential toxicity and environmental impact, which must be carefully considered. These risks can originate from both the encapsulated fillers and the CNTs themselves, with the latter having raised significant concern. In fact, in the early days, CNTs were reported as toxic, and were linked to negative biological effects and classified as possibly carcinogenic, but this toxicity was mainly caused by the NPs employed as catalyst (in non-purified samples) or from the presence of very long CNTs or bundles, which were even compared to asbestos. Several studies have shown that such toxicity can be largely mitigated by using purified, short and well-dispersed CNTs.

The environmental impact of filled CNTs requires a context-dependent regulatory approach. A complete life-cycle analysis it is indeed necessary, that includes the raw materials used for their preparation, synthesis conditions, composition of the new heterostructure, potential uses and disposal strategy. The main environmental concerns associated with the methods of synthesis of filled CNTs stemmed from the high energy demand and GHG emissions of *in situ* synthetic approaches, such as CVD or AD, often requiring high *T* conditions, expensive compressed gas and toxic chemicals and catalysts. To address these aspects, the use of sustainable carbon sources is becoming increasingly attractive, such as vegetable oils and biowaste.^1021^

An end-of-life planning including a predetermined disposal method will contribute to significantly mitigate negative environmental and toxicological effects of filled CNTs. The entities encapsulated within the CNTs might have their own inherent toxicity. Therefore, disposal and recycling strategies should consider the presence of hazardous materials and their potential release or transformation during recycling and disposal. A significant concern lies in the potential environmental persistence and accumulation of filled CNTs. In this respect, it would be advisable to induce structural defects on the walls of the filled CNTs before their disposal which would accelerate their degradation. Table 14 includes examples of filler materials and the specific considerations made to establish an adequate harm-risk profile and disposal strategy.

**Table 14:** Most Common Fillers and Their Disposal Alternatives

Filler Material	Primary Environmental Hazard (Upon Release)	Key Mechanisms of Toxicity	Potential Disposal/End-of-Life Alternatives
Heavy Metals (Cd, Pb, Hg)	Non-biodegradability & Bioaccumulation	• Direct toxicity	Chemical Stabilization/Solidification: Encapsulate the waste in cement, glass, or polymer matrices before landfilling to prevent leaching.
		• Accumulation in the food chain.	High-Temperature Vitrification: Converting the material into a stable, non-leachable glass-like substance.
Transition Metals (Fe, Ni, Co)	Oxidative Stress & Cellular Damage	Catalysis of harmful ROS (Fenton reaction); DNA/cell damage.	Pyrolysis/Thermal Treatment: High-temperature inert gas treatment to recover metal catalysts in a less reactive form or to destroy the carbon shell, reducing the hazard from the long CNT structure. Metallurgical Recycling.
Metal Oxides	Ecotoxicity to Aquatic Organisms	Particle-specific toxicity; slow dissolution to release toxic metal ions.	Acid/Base Leaching followed by Recovery: Dissolving the metal oxide filler in a controlled environment and recovering the metal ions for reuse (resource efficiency). Secure Landfilling (if metal recovery is not feasible).
Metal Salts	Osmotic Stress & Dissolution Toxicity	High dissolution rate releasing toxic ionic components rapidly into water.	Washing and Regeneration: For adsorbent applications, use controlled washing (acid/base) to regenerate the CNT and collect the concentrated salt solution for treatment or recovery. Controlled Neutralization.
Organic Drugs/Pesticides	Pharmacological Activity/Ecotoxicity	The released substance maintains its original biological effects (e.g., endocrine disruption).	Incineration: High-temperature thermal destruction to completely break down the organic filler into CO_2_, water, and inert ash, thus eliminating the biological activity. Solvent Extraction for recovery (if high-value).
CNT Shell Itself (Long/Rigid Structures)	Physical Hazard & Biopersistence	Respiratory hazard (similar to asbestos); non-specific toxicity.	Chemical Oxidation/Wet Oxidation: Using strong oxidizing agents (HNO_3_, H_2_O_2_) to shorten and break down the long, rigid CNTs into less hazardous, shorter carbon species that are easier to degrade.

Paradoxically, the cavities of CNTs can serve in environmental remediation. They are for instance highly effective adsorbents used to remove pollutants, such as heavy metals from contaminated water or act as high surface area reservoirs either of GHGs or H_2_ (finding application in clean energy generation). For these applications, the end-of-life hazard risk is crucial. Determining the stability of the encapsulated molecules is primarily required to avoid the platform to become a secondary pollution source that can contaminate soil or groundwater.

Regarding recycling and reuse, current practices for CNTs in general, often involve landfilling or incinerating materials containing CNTs at their end-of-life.^1022^ Toxicological considerations at the end of life are vital for determining whether filled CNTs can be safely reused, repurposed, or recycled, or how to ensure safe disposal if these pathways are not feasible.

The form in which filled CNTs are used is important to determine their disposal and potential recyclability. While some applications require the use of filled CNTs by themselves or with only slight surface modifications, in other cases the integration into matrices or complex devices may be required. When CNTs are integrated into polymeric matrices, a primary consideration is whether they are permanently embedded or can be readily removed from the composite, as the mode of integration determines the subsequent separation strategy. Depending on the forces responsible for matrix stability, chemical treatments or the application of mechanical forces may enable processing, extraction, and recycling of CNTs. For applications in which CNTs function as integral components of mixed-material fibers or complex devices, recyclability is less straightforward. In such cases, end-of-life strategies typically involve dissolution of the matrix and reprocessing of the recovered raw materials using selected solvents. Siqueira et al. demonstrated that CNT fibers can be fully recycled while maintaining their mechanical, electrical, or structural properties.^1023^ The authors prepared single-source fibers of both SWCNTs and DWCNTs by independently dispersing the CNTs in chlorosulfonic acid (CSA), followed by fiber spinning. The solution-spun CNT fibers were straightforwardly recycled following the CSA-mixing reprocessing shown in Figure 51. Irrespective of their constituent source materials, the recycled fibers maintained their morphology, structure, alignment, and properties.

**Figure 51:**
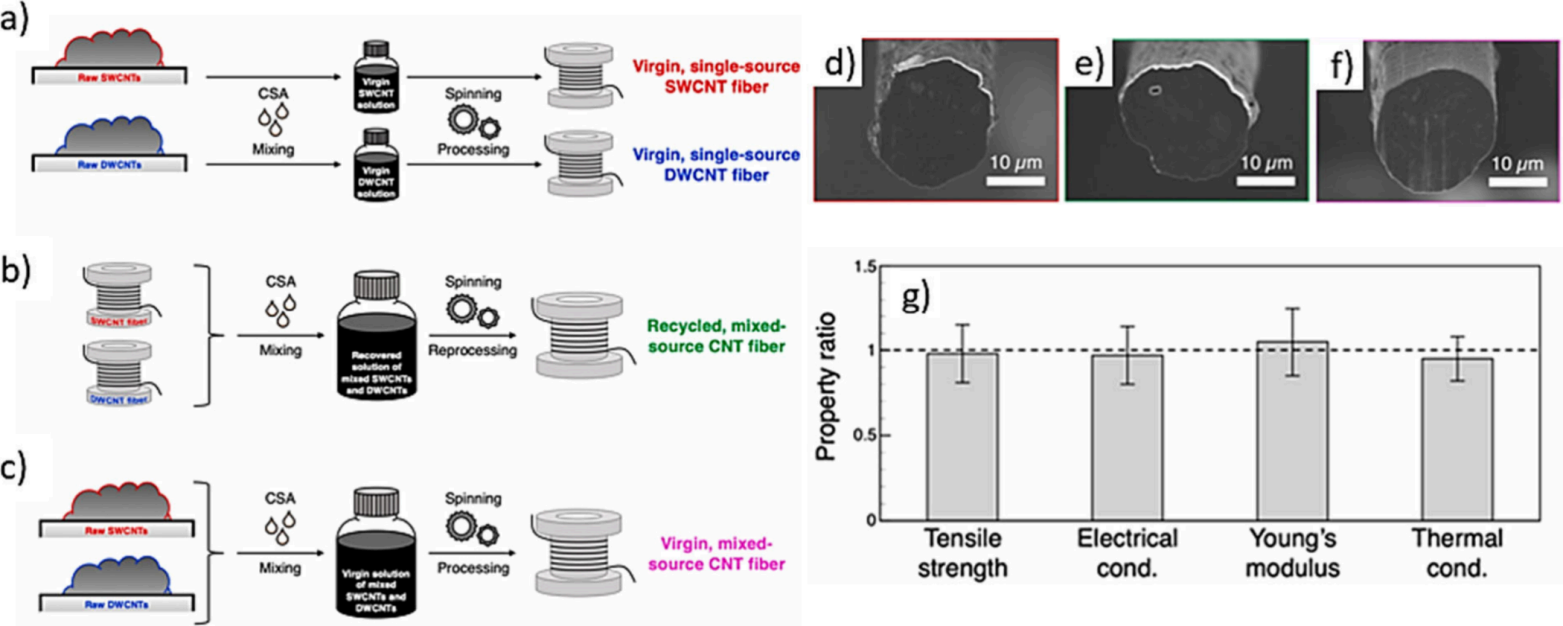
Schematic representation of recycling process of solution-spun CNTs fibers. a) Both SWCNTs and DWCNTs were independently mixed in CSA solutions and processed to obtain single source fibers. b) For recycling, the virgin carbon fibers were redispersed and processed using the protocol described above. c) Virgin mixed source CNTs were prepared for comparison. SEM micrographs of d) virgin SWCNT fiber, e) recycled mix-source fiber, and f) virgin mix-source fiber confirmed that after the recycling process CNT fiber maintain their morphology intact. g) Ratio of properties of the recycled, mixed-source CNT fiber to properties of the virgin mixed-source CNT fiber^1023^ Reproduced with permission from ref 1023. Copyright 2025, Elsevier.

Battery applications involve mixing CNTs with binders and active materials such as lithium metal oxides. In this case, recovery of the metal component alongside the C has been accomplished by hydrometallurgy or pyrolysis. On the other side, the disposal of filled CNTs that have been previously integrated into electronic devices faces important challenges owing to the complexity of these systems. Specific strategies, beyond standard recycling, associated with removal of components and chemicals simultaneously preventing the release of hazardous materials are required. The ideal end-of-life strategy may include presorting, using specialized recycling techniques, and the practice of safety protocols to reduce the toxicity risks.

Creating a more sustainable and circular economy by upcycling waste derived from the use of advanced materials in current technologies is a key goal. Converting waste into valuable products emerges as a new business opportunity, in which industrial processes become more profitable owing to the virtual elimination of procedures associated with the disposal of materials, and therefore of the costs derived of their elimination, and the reduction of other indirect expenses, such as government taxes stablished due to the environmental impact of such activities. Moreover, waste valorization can decrease the dependence of the technology evolution to the use of primary and often scarce resources. Despite the promising potential of this approach, there are main challenges associated with scalability, cost-effectiveness, purity, and standardizing production processes. A comprehensive framework for responsible and sustainable development in the field is therefore required.^1024^ Owing to the high stability of CNTs under external stimuli and conditions of physical and chemical stress, using these nanostructures for the production of new functional materials emerges as a sustainable alternative to traditional recycled raw materials, which typically degrade upon processing.

## 10 Conclusions, Main Challenges and Future Remarks

The possibility of using the cavities of CNTs to load materials into their interior was explored shortly after the initial reports on the structure of CNTs in the early 90s. Since then, the number of materials successfully encapsulated within CNTs has grown exponentially, leading to new combinations and complex architectures. Scientists have demonstrated the fundamental interest in these systems and explored their applications in several fields.

The encapsulation of a wide variety of compounds, ranging from organic and inorganic solid materials to liquids and gases, has led to novel hybrid structures with new or enhanced properties, some of which arise from QC effects. CNTs have proven highly effective in the stabilization of several compounds, protecting them from degradation. The inner cavities of CNTs also allow controlled reactions to be performed at the nanoscale. Not surprisingly, filled CNTs have found applications in biomedicine, catalysis, energy storage, gas storage, gas separation, nanoelectronics, spintronics, magnetic storage, sensing technologies and as nanoreactors.

A myriad of synthetic approaches has led to the encapsulation of foreign materials within CNTs. The final properties of the resulting hybrids will be not only determined by the filling material but also by the employed filling method, CNT type and functionalization. Figure 52 summarizes the main degrees of freedom associated with the encapsulation process.

**Figure 52:**
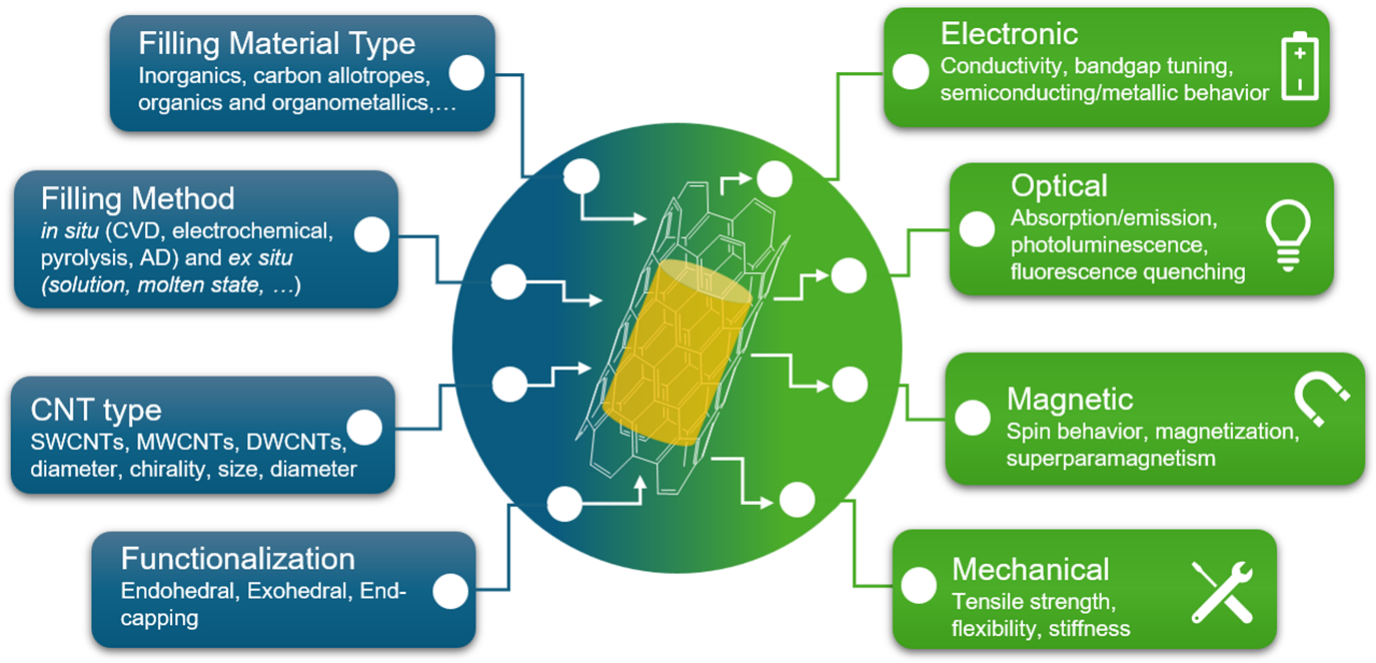
Schematic representation illustrating how the chosen synthetic approach for encapsulating foreign materials within CNT’s cavities governs the final properties of the resulting hybrids.

Despite the extensive amount of research devoted to filled CNTs, several challenges still lie ahead. A major challenge, not limited to filled CNTs but to CNT research in general, is the heterogeneity of the CNT samples. Differences in diameter, length, chirality, defect density, and the presence of impurities can significantly influence the physicochemical properties of the material. Batch-to-batch variations are often observed even when CNTs are obtained from the same source. Therefore, to advance in this area of research, it is important that authors report the source and characteristics of the CNTs used in their studies. This includes information on purification procedures and any pretreatments applied before filling. Furthermore, several reports on filled CNTs also lack details on the experimental conditions employed for the filling and post-processing steps, such as the removal of external non-encapsulated compounds. Without these experimental details, it is difficult for other research groups to reproduce the reported studies and build upon previous work. Developing new synthetic strategies that minimize the introduction of defects and impurities within CNTs will also contribute to improving the performance of the resulting hybrid. Significant efforts must also be directed toward mild processing and external functionalization strategies that preserve the integrity, at least to a certain extent, of the CNT walls and tips to avoid leakage of the encapsulated compounds. This is a key aspect, particularly when using SWCNTs and covalent functionalization strategies since only one graphene layer separates the encapsulated compounds from the external environment. Post-filling functionalization allows for an increase in the dispersibility and ease of processing of filled CNT samples.

Characterization techniques have played a pivotal role in advancing this field of research. Initial studies on the structural determination of inorganic crystals confined within SWCNTs required the image reconstruction of several HRTEM images. Currently, high-end atomic resolution can be directly achieved using aberration-corrected microscopy, which can be combined with analytical techniques in the same equipment. Understanding the encapsulation process at the atomic and molecular levels, as well as the interactions established at the CNT-guest interface once the filler reaches the inner surface of the CNTs, would certainly contribute to controlling the morphology and structure of the encapsulated system. Therefore, an in-depth characterization of interfacial phenomena is required. There are still numerous challenges in terms of characterization tools that can provide detailed insights into the structure, composition, and properties at the nanoscale level. Although high-resolution electron microscopy and spectroscopy techniques have been widely employed and provide crucial insights, *in situ* characterization remains largely unexplored. Efforts should focus on the real-time monitoring of the encapsulation process and *in situ* analysis of the physical properties and behavior of filled CNTs under operational conditions, namely, during catalysis, energy, or gas storage. These can, for instance, be tuned via external stimuli using specialized sample holders. Advancements in synchrotron-based methods and cryo-TEM are also expected to significantly impact in this area of research.

Efforts should be made by the research community to standardize the protocols employed to determine the filling yield. Although electron microscopy remains the most commonly used technique, it provides local information and is user dependent. Bulk analytical techniques, such as TGA, have been proposed but are not widely employed. When using TEM to assess the filling yield, we suggest reporting the percentage of the CNT length that is filled versus the observed empty cavity, rather than considering an individual CNT as filled, even if only a small fraction of the CNT contains the encapsulated compound.

Poor control over the filling yield, and heterogeneity in terms of the number of filled CNTs present in the sample after encapsulation are also important issues. Tuning the reaction conditions may contribute to minimizing the coexistence of different nanostructures within the cavities of CNTs, which morphology, crystal structure and interactions with the hosting CNTs determine the properties and hence further applications of the obtained hybrid. While the width of the guest species is limited by the diameter of the host, the guest can occupy different areas along the CNT length, forming aggregates, NPs, NTs, or discontinuous crystallites, which print differences even in different areas of the same tube. Achieving high filling yields, for instance, will contribute to the homogeneity of the sample and, therefore, to the uniformity of the properties of the bulk material. Sample homogeneity can be greatly improved by using high-purity CNTs with well-defined diameters, lengths, and even chiralities as starting materials. An alternative strategy, which has been barely been explored would consist in the separation of already filled CNTs. Both strategies result in high production costs and can only be employed for fundamental research or applications where a small amount of filled CNTs are required.

Although the number of reports on filled CNTs is extensive, encapsulating highly reactive or sensitive species is one of the most challenging aspects in terms of preparation approaches. The simultaneous or sequential encapsulation of multiple materials to create more complex hybrid systems will also pave the way for the controlled nanoengineering of functional materials with tailored and unprecedented properties.

Computational tools have played a pivotal role and will continue to do so. Remarkably, the first report highlighting the possibility of filling CNTs was a theoretical study published in 1992. Advanced theoretical modeling and simulations are crucial for understanding host–guest interactions, guiding experimental design and material selection, and elucidating and even predicting novel and unexpected properties. Efforts should also be devoted to take advantage from ML in the area of filled CNTs. It will allow to accelerate the rational design of novel hybrid structures by predicting structure–property relationships. By learning from vast data sets of simulations and experiments, ML can rapidly screen candidate materials, identify non-intuitive synthetic strategies, and predict complex behaviors, thereby opening new opportunities in this field of research.

A large variety of filled CNTs have been and are being studied for the different research areas discussed in this review, each of them having their own advantages and disadvantages. The areas in which we believe that filled CNTs will have a major impact are those that benefit from (i) a synergistic effect between the filler and the CNTs and (ii) the unique structure of CNTs, i.e., a very small cavity surrounded by graphene layers. These graphene layers provide a perfect shelter to protect materials in their interior. Therefore, from a fundamental science perspective, CNTs will continue to serve as excellent vessels to investigate growth of otherwise unattainable materials, reactions down to the atomic scale and quantum phenomena. The encapsulation of vdW solids in the interior of CNTs also requires further research. Only few reports are available on this type of structures and a myriad of 1D tubular heterostructures can be created that combine the properties of both 1D and 2D materials. In all this areas, the toxicity and environmental impact of filled CNTs are minimal because a very small amount of material is required. At a larger scale, the application that we think might have a larger impact is the energy field where the graphitic structure offers protection of the electroactive materials thus improving their performance. In this case, a proper life-cycle analysis is essential. It is also worth stressing that when filled CNTs are incorporated into composite materials their potential toxicity is largely alleviated because they no longer behave as individual objects.

Finally, for filled CNTs to move closer to the market, it is necessary to develop easily scalable, cost-effective and safe and sustainable by design processes for their production. In this context, employing environmentally friendly processes that are energy-efficient and rely on non-hazardous chemicals is highly desirable throughout the synthesis and purification of CNTs, as well as during encapsulation and post-treatment steps. The economic feasibility for large scale applications, such as the above-mentioned energy field, must also be assessed. This will depend not only the source of CNTs, which price largely depends on the purity and homogeneity of each batch, but also on the filling material. Cost-calculations should be made on specific systems since will largely vary depending on the chemicals and processes employed. For instance, the price of CNTs, which would only be one of the components, ranges from 50 $/kg up to 20.000 $/kg.^1025^

Policies should actively promote the principles of a circular economy for CNTs. This includes eco-design for recyclability, optimizing production for energy efficiency, and developing chemical recycling of filled CNT waste that would otherwise be incinerated or sent to landfills. This approach aims to recover valuable materials and reduce the need for raw material extraction, positioning filled CNTs as more sustainable components of the economy. A life-cycle assessment is fundamental for understanding safety, environmental, and economic impacts of filled CNTs. This life-cycle assessment will be largely dependent on the envisaged application.

With ongoing progress in the synthesis of novel filled CNTs and the development of cutting-edge characterization techniques and computational tools, filled CNTs hold immense potential in both fundamental and applied sciences. To summarize, Figure 53 shows an schematic representation that includes key aspects related to filled CNTs. It emphasizes areas of interest, highlights modeling and characterization as key players in their development, areas of application, future research directions, and the main challenges in the field.

**Figure 53:**
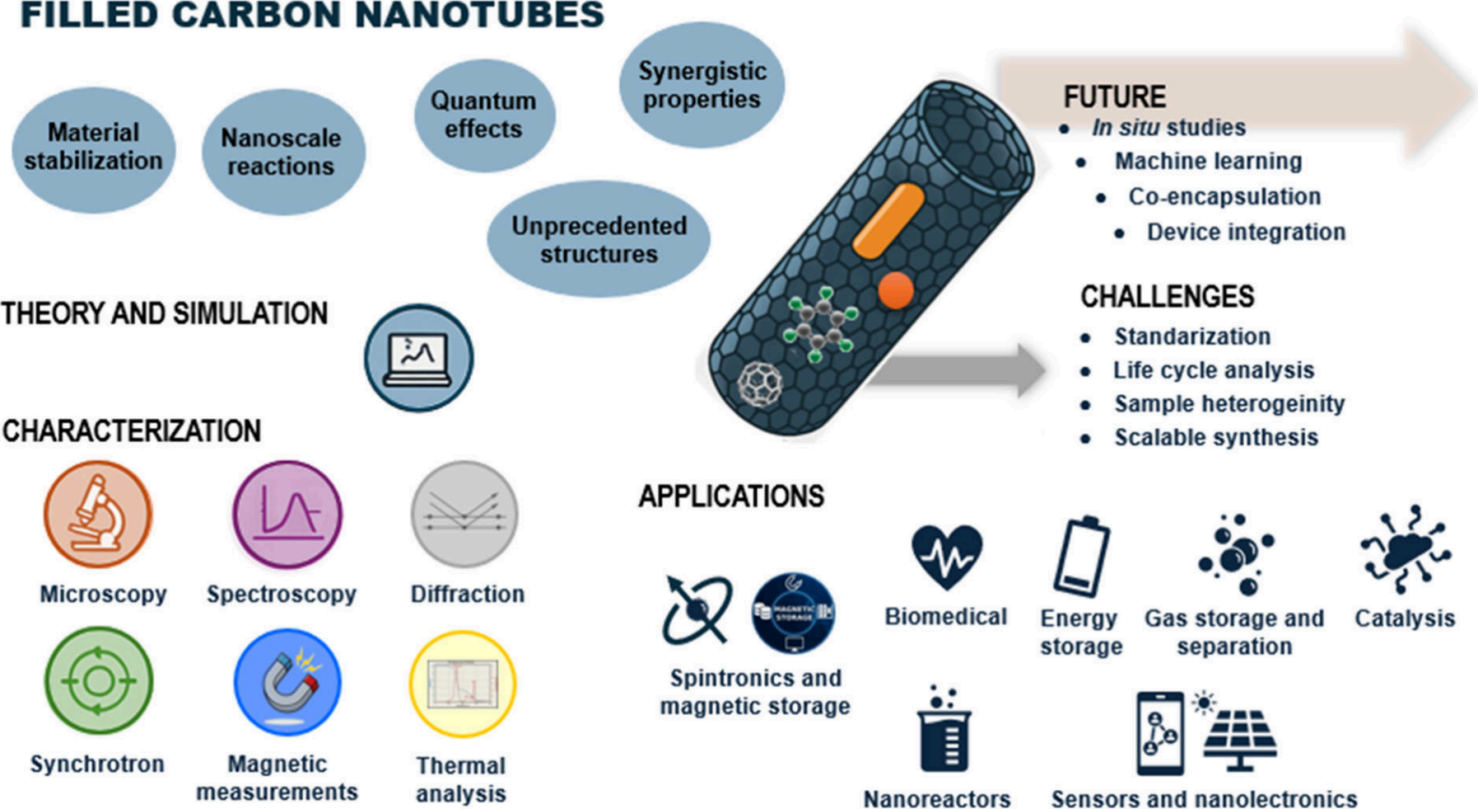
Schematic representation of the key aspects related to filled CNTs, including characterization, applications, future directions and main challenges.

## Abbreviations

1D: One-dimensional
2D: Two-dimensional
3D: Three-dimensional
4T: Quaterthiophene
5T: Quinquethiophene
6T: α-sexithiophene
AC: Active carbon
acac: Acetylacetonate
acFeCp_2_: Acetylferrocene
AD: Arc Discharge
AI: Artificial Intelligence
AIBN: Azobis­(isobutyronitrile)
AIMD: ab initio Molecular Dynamics
AIP: Adiabatic Ionization Potential
APTS: 3-aminopropyl-triethoxysilane
BCNT: B-doped Carbon Nanotube
bz-THQ: 5,6,7,8-tetrahydroquinoline
(C_5_H_5_)_2_, Cp: Cyclopentadienyl
C_60_: Buckminsterfullerene
CAL: Cinnamaldehyde
Car: β-carotene
CB: Carboplatin
CC: Coupled Cluster
CDDP: Cisplatin (cis-diamminedichloroplatinum)
CI: Configuration Interaction
CNC: Carbon Nanocage
CNT: Carbon Nanotube
CO_2_RR: CO_2_ Reduction Reaction
COL: Cinnamyl Alcohol
C–P: Phosphorus (P) Chains
CoPc: Cobalt Phthalocyanine
CV: Cyclic Voltammogram
CVD: Chemical Vapor Deposition
CVT: Chemical Vapor Transport
ΔG: Gibbs free energy
DFT: Density Functional Theory
DHQ: Decahydroquinoline
DMSO: Dimethyl sulfoxide
DOX: Doxorubicin
DPSQ: 2,4-bis­[4-(N,N-diphenyl amino)-2,6-dihydroxyphenyl] squaraine
DTA: Differential Thermal Analysis
DWCNT: Double Walled Carbon Nanotube
E: Activation Energies
e^–^: Electrons
EC: Electrochemical
ED: Electron Diffraction
EELS: Electron Energy Loss Spectroscopy
EGFR: Epidermal Growth Factor Receptor
EM: Electron microscopy
EPR: Electron Paramagnetic Resonance
EDX: Energy-Dispersive X-ray Spectroscopy
Fcc: Face-Centered Cubic
FeCp_2_: Ferrocene
FEM: Finite Element Method
FePc: Iron­(II) Phthalocyanine
FESEM: Field Emission Scanning Electron Microscopy
FET: Field-Effect Transistors
FIR: Far Infrared
FRET: Förster Resonance Energy Transfer
FT: Fischer–Tropsch
FTIR: Fourier Transform Infrared Spectroscopy
GCE: Glassy Carbon Electrodes
GdNT: Gadonanotube
GC: Grand Canonical
GCMC: Grand Canonical Monte Carlo
GEM: Gemcitabine
GHG: Greenhouse Gas
GNR: Graphene Nanoribbon
HAADF: High-Angle Annular Dark-Field
HER: Hydrogen Evolution Reaction
HerPc_2_: Erbium biphthalocyanine
HF: Hartree–Fock
Hfac: Hexafluoroacetylacetone
HiPco: High-Pressure CO
HRSTEM: High resolution Scanning Transmission Electron Microscopy
HRTEM: High-Resolution Transmission Electron Microscopy
ICP: Inductively Coupled Plasma Spectroscopy
ICP-MS: Inductively Coupled Plasma Mass Spectrometry
IL: Ionic Liquid
IR: Infrared
KH560: 3-glycidyloxypropyl trimethoxy
LA: Laser Ablation
LDL: Low detection limit
LIB: Lithium-Ion Battery
LiNCT: Lithium Neutron Capture Therapy
L–OHP: Oxaliplatin
LV: Laser Vaporization
M: Metal
mAb: Cetuximab
MB: Methylene Blue
MC: Metal carbide
MCp_*x*_: Metallocene
MD: Molecular Dynamics
MFM: Magnetic Force Microscopy
ML: Machine Learning
MM: Molecular Mechanics
MMM: Mix matrix membrane
MOF: Metal–Organic Framework
MOX: Moxifloxacin
MPH: Molten-Phase
MPn: Møller–Plesset Perturbation
MRBI: Magnetic Resonance Bioimaging
MRI: Magnetic Resonance Imaging
MSC: Mesenchymal Stem Cell
MWCNT: Multiwalled Carbon Nanotube
MX: Metal halide
M_*x*_O_*y*_: Metal Oxide
NCSI: Negative Spherical Aberration Imaging
NCNT: Nitrogen-doped Carbon Nanotube
ND: Neutron Diffraction
NEXAFS: Near Edge X-ray Absorption Fine Structure Spectroscopy
NIB: Sodium Ion Battery
NiCp_2_: Nickelocene
NIR: Near Infrared
NMR: Nuclear Magnetic Resonance
NP: Nanoparticle
NR: Nanorod
NT: Nanotube
NW: Nanowire
NX: Norfloxacin
OAS: Optical Absorption Spectroscopy
OER: Oxygen Evolution Reaction
ORR: Oxygen Reduction Reaction
PANi: Polyaniline
Pc: Phthalocyanine
PBI: N,N′-bis­(3-pentyl)­perylene-3,4,9,10-bis­(dicarboximide)
PCL: Poly-*ε*-caprolactone
Pd-AC: Palladium NPs supported on active carbon
Pd-CNT: Palladium decorated CNTs
PE: Polyethylene
PEG–PCL: Polyethylene glycol-poly-*ε*-caprolactone
PL–PEG: Phospholipid-polyethylene glycol
PEO: Poly­(ethylene oxide)
PET: Positron Emission Tomography
PL: Photoluminescence
PLE: Photoluminescence Excitation
PLGA: Poly­(lactic-*co*-glycolic acid)
PLV: Pulsed laser vaporization
PMS: Peroxymonosulfate
POM: Polyoxometalate
PP: Polyphosphides
PPy: Polypyrrole
PROX: Preferential Oxidation
PTH: 10-phenylphenothiazine
PVA: Poly­(vinyl alcohol)
PVC: Polyvinyl Chloride
PS: Polystyrene
PVT: Physical Vapor Transport
Py: Pyrrole
Py-THQ: 1,2,3,4-tetrahydroquinoline
QC: Quantum Confinement
QCM: Quartz Crystal Microbalance
QCM-D: QCM with Dissipation Monitoring
QMC: Quantum Monte Carlo
RIXS: Resonant Inelastic X-ray Scattering
r-P: Ring-shaped Phosphorus
RT: Room Temperature
RUB: Robeson Upper Bound
SAED: Selected Area Electron Diffraction
SAXS: Small-Angle X-ray Scattering
SCO: Spin Crossover
scCO_2_: Supercritical CO_2_
SC-P: Square columnar Phosphorus
SCR: Selective Catalytic Reduction
SEM: Scanning Electron Microscopy
siRNA: Small Interfering RNA
SLNT: Single Layered Nanotube
SMM: Single Molecule Magnet
SMX: Sulfamethoxazole
SNAP: S-nitroso-N-acetyl-d-penicillamine
SR-EELS: Spatially Resolved Electron Energy Loss Spectroscopy
STEM: Scanning Transmission Electron Microscopy
SWCNT: Single Walled Carbon Nanotube
T: Temperature
Tc: Critical Temperature
TC: Tetracycline hydrochloride
TCNQ: Tetracyano-p-quinodimethane
TDAE: Tetrakis (dimethylamino)­ethylene
TEM: Transmission Electron Microscopy
TGA: Thermogravimetric Analysis
Thd: Tetramethylheptanedione
TMC: Transition Metal Chalcogenide
TMV: Tobacco Mosaic Virus
TPD: Temperature-Programmed Desorption
TTF: Tetrathiafulvalene
UHV: Ultrahigh vacuum
UOR: Urea Oxidation Reaction
UPS: Ultraviolet Photoelectron Spectroscopy
UV: Ultraviolet
UV–vis-NIR: Ultraviolet–Visible-Near-Infrared
vdW: van der Waals
WAXS: Wide-Angle X-ray Scattering
XANES: X-ray Absorption Near Edge Structure
XPS: X-ray Photoelectron Spectroscopy
XAS: X-ray Absorption Spectroscopy
XMCD: X-ray Magnetic Circular Dichroism
XRD: X-ray Diffraction
XRS: X-ray Scattering
ZnPc: Zinc Phthalocyanine
ZR: Zigzag Nanoribbons
